# METTL3 promotes homologous recombination repair and modulates chemotherapeutic response in breast cancer by regulating the EGF/RAD51 axis

**DOI:** 10.7554/eLife.75231

**Published:** 2022-05-03

**Authors:** Enjie Li, Mingyue Xia, Yu Du, Kaili Long, Feng Ji, Feiyan Pan, Lingfeng He, Zhigang Hu, Zhigang Guo

**Affiliations:** 1 Jiangsu Key Laboratory for Molecular and Medical Biotechnology, College of Life Sciences, Nanjing Normal University Nanjing China; University of Iowa United States; Harvard Medical School United States

**Keywords:** METTL3, homologous recombination repair, chemotherapeutic response, EGF, RAD51, Mouse

## Abstract

Methyltransferase-like 3 (METTL3) and N^6^-methyladenosine (m^6^A) are involved in many types of biological and pathological processes, including DNA repair. However, the function and mechanism of METTL3 in DNA repair and chemotherapeutic response remain largely unknown. In present study, we identified that METTL3 participates in the regulation of homologous recombination repair (HR), which further influences chemotherapeutic response in both MCF-7 and MDA-MB-231 breast cancer (BC) cells. Knockdown of METTL3 sensitized these BC cells to Adriamycin (ADR; also named as doxorubicin) treatment and increased accumulation of DNA damage. Mechanically, we demonstrated that inhibition of METTL3 impaired HR efficiency and increased ADR-induced DNA damage by regulating m6A modification of EGF/RAD51 axis. METTL3 promoted EGF expression through m6A modification, which further upregulated RAD51 expression, resulting in enhanced HR activity. We further demonstrated that the m6A ‘reader,’ YTHDC1, bound to the m6A modified EGF transcript and promoted EGF synthesis, which enhanced HR and cell survival during ADR treatment in BC. Our findings reveal a pivotal mechanism of METTL3-mediated HR and chemotherapeutic drug response, which may contribute to cancer therapy.

## Introduction

m^6^A modification of RNA has been reported to participate in regulating numerous cellular processes in eukaryotes (Wang et al., 2014; Wang et al., 2015; Xiang et al., 2017). METTL3 is a key member of the m^6^A methyltransferase complex, which also includes co-factors METTL14 and the Wilms tumor 1 associated protein (WTAP) (Liu et al., 2014; Wang et al., 2015). This RNA modification can be recognized by a set of m^6^A-binding proteins, including YTH domain containing family protein (YTHDF1/2/3), YTH domain containing 1/2 (YTHDC1/2), and insulin like growth factor 2 mRNA binding protein (IGF2BP1/2/3), which serve as m^6^A ‘readers’ and mediate specific functions of m^6^A-modified RNA (Deng et al., 2018; Wang et al., 2015). Furthermore, fat mass and obesity-associated protein (FTO) and RNA demethylase, ALKBH5, work as m^6^A ‘erasers’ to remove m^6^A modifications from RNA (Deng et al., 2018). Recent studies have indicated that the m^6^A modification and METTL3 play important roles in the progression and chemotherapy response of various cancers, including BC (Cai et al., 2018; Deng et al., 2018; Pan et al., 2021).

Chemotherapy is used in early-stage BC and locally advanced BC to provide an improved chance for breast-conserving surgery, reduce the risk of recurrence, and increase survival rates (Fisusi and Akala, 2019). The use of adjuvant chemotherapy remains an effective treatment for triple-negative BC and other types of invasive breast cancer (Hennigs et al., 2016). Several chemotherapeutic agents, including ADR, docetaxel, 5-fluorouracil, and cisplatin are used in combination chemotherapy for BC treatment (Fisusi and Akala, 2019). Since 1970s, ADR was considered as the most active chemotherapeutic agent for the treatment of BC, which was used in neoadjuvant chemotherapy or combination therapy (Chan et al., 1999; Fisher et al., 1998; Paridaens et al., 2000). Chemotherapeutic agents, such as ADR, induce apoptosis by causing DNA damage (He et al., 2016; Lu et al., 2020). Targeting the DNA damage response (DDR) may enhance the sensitization of cancer cells to chemotherapeutic drugs (Li et al., 2021; Lu et al., 2020). Moreover, elevated DNA repair activity contributes to the drug resistance of cancer cells treated with chemotherapy (Li et al., 2021; Lu et al., 2020).

Key proteins involved in DNA repair, such as BRCA1, RAD51, and RAD52 in HR, xeroderma pigmentosum group C (XPC) in nucleotide excision repair (NER), and flap endonuclease 1 (FEN1) in base excision repair (BER), influence the susceptibility of various cancers and may be suitable targets in cancer mono- and combination therapy (Ali et al., 2017; Grundy et al., 2020; Hengel et al., 2016; Huang et al., 2016; Li et al., 2021; Lu et al., 2020; Malik et al., 2020; Miki et al., 1994). In addition, mutations in several DDR proteins, such as BRCA1, BRCA2, PALB2, and RAD51 also contribute to hereditary breast and ovarian cancer (Hengel et al., 2017; Li et al., 2021; Lok et al., 2013). Mutations in these proteins incapacitates HR and results in synthetic lethality with inhibition of RAD52 or PARP1, suggesting a novel strategy for treating patients with BRCA1/2/PALB2-mutant tumors (Bryant et al., 2005; Farmer et al., 2005; Lok et al., 2013). However, targeting key DDR proteins to improve sensitization of cancer cells and circumvent cancer cell resistance remain significant challenges to achieving satisfactory therapeutic effects (Li et al., 2021; Pan et al., 2021). Therefore, exploring novel treatment strategies and mechanisms that affect DDR in cancer cells may contribute to improved treatment for these diseases.

Currently, METTL3 and m^6^A modification have been implicated in DDR (Xiang et al., 2017; Yu et al., 2021b; Zhang et al., 2020). However, the molecular mechanism of METTL3 and m^6^A modification in DDR, especially in the context of chemotherapeutic drug-induced DDR, remains unexplored. Here, we report that METTL3 is involved in the regulation of HR by regulating the EGF/RAD51 axis. Knockdown of METTL3 sensitizes both MCF-7 (estrogen receptor (ER)-positive BC cell) and MDA-MB-231 (MB-231, triple-negative BC cell) BC cells to ADR, impairs HR, and induces significant DNA damage. Moreover, YTHDC1 was identified as the reader that binds and protects m^6^A-modified EGF mRNA and regulates DNA repair and the response of BC cells to ADR. Overall, our findings provide insight into the function and mechanism of METTL3 in HR and the chemotherapeutic drug response in BC, and demonstrate the potential of targeting METTL3 as an antitumor treatment.

## Results

### METTL3 regulates chemotherapeutic response of BC cells

**Figure 1.**
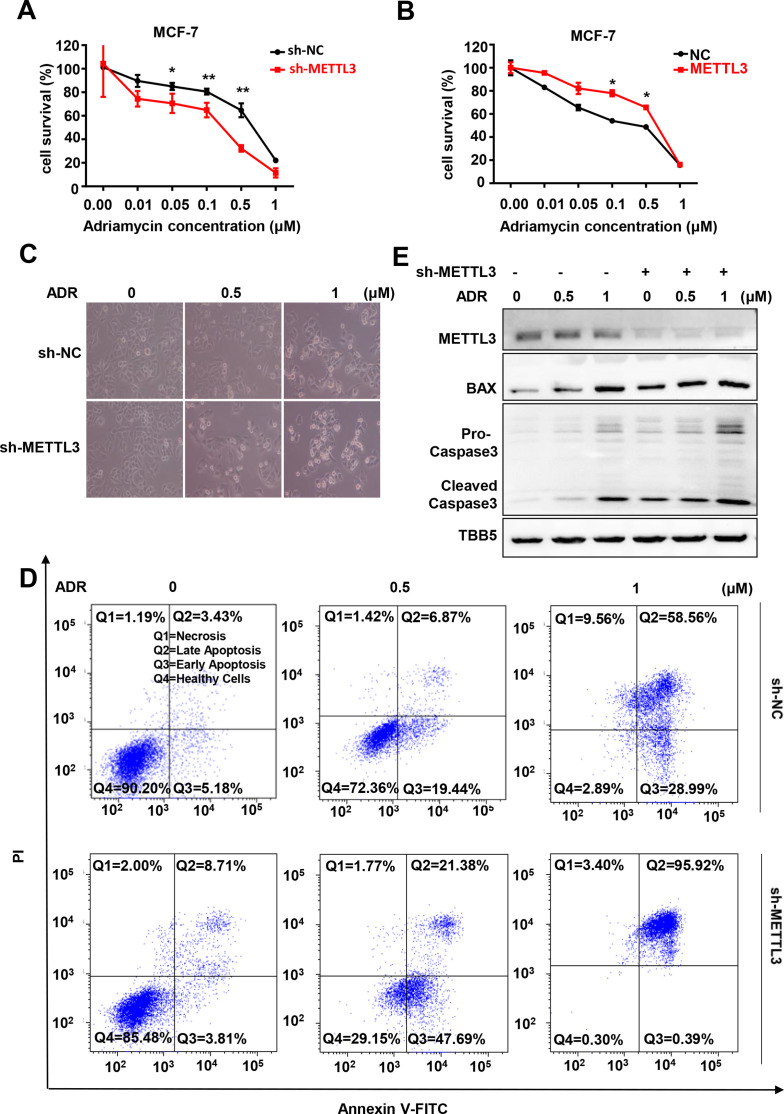
Knockdown of Methyltransferase-like 3 (METTL3) sensitizes both MCF-7 and MB-231 cells to Adriamycin (ADR). (**A, B**) MTT assays were performed to determine the effect of METTL3 on ADR cytotoxicity in MCF-7 or MDA-MB231 cells. Data are expressed as the mean ± standard deviation (SD), n=3 per group. (**C**) Morphological analysis of MCF-7 with different drug treatments. (**D**) Annexin V/PI staining and flow cytometry assay of control or METTL3-KD MCF-7 cells with different drug treatments. (**E**) Western blot (WB) analysis of METTL3, BAX, and Caspase 3 in control or METTL3-KD MCF-7 cells treated with various concentrations of ADR. All statistical data are presented as the mean ± SD. * p<0.05; ** p<0.01; *** p<0.001 (Student’s *t*-test). Figure 1—source data 1.Source data for Figure 1E.

METTL3 has been reported to be involved in the progression of several types of cancers, including BC (Deng et al., 2018; Wang et al., 2020a). We wonder whether METTL3 regulates chemotherapeutic response of BC cells. First, we identified the elevated METTL3 and m^6^A levels in five types of BC cells, including MCF-7, MB-231, T47D, SKBR3, and BT474 cells (Figure 1—figure supplement 1A, B). Then, we investigated the role of METTL3 in regulating the sensitivity of both MCF-7 and MB-231 cells (ER-positive and triple-negative cells, respectively) to chemotherapeutic drugs with stable METTL3-OV or -KD cell lines (Figure 1—figure supplement 1C, D). Cell viability assays were performed using METTL3-KD and METTL3-OV MCF-7 stable cell lines treated with five first-line chemotherapeutic drugs including 5-FU, cisplatin (DDP), ADR, paclitaxel, and carboplatin. The results indicated that modification of METTL3 expression markedly attenuated the sensitivity of MCF-7 cells to ADR compared with the other drugs (Figure 1A and Figure 1—figure supplement 1E-I; Andreetta et al., 2010). Cell viability assays using METTL3-OV and -KD MB-231 stable cells verified the effect of METTL3 on ADR sensitivity (Figure 1B and Figure 1—figure supplement 1E). These results were further verified in a morphological analysis, which showed that silencing METTL3 enhanced chemotherapeutic drug sensitivity (Figure 1C), whereas overexpression of METTL3 decreased sensitivity in MCF-7 cells (Figure 1—figure supplement 1J). Furthermore, flow cytometry analysis demonstrated that treatment with ADR induced higher apoptosis rates in both METTL3-KD MCF-7 and MB231 cells compared with control cells (Figure 1D and Figure 1—figure supplement 1K). Accordingly, elevated pro-apoptotic Bax and caspase 3 were detected in METTL3-KD BC cells treated with ADR (Figure 1E). We then detected the effect of METTL3 on ADR-sensitivity using a human non-tumorigenic breast epithelial cell line MCF-10A. Our data showed that overexpression of METTL3 attenuated the sensitivity of MCF-10A cells to ADR (Figure 1—figure supplement 1L-N). As expected, silence of METTL3 reduced global m^6^A levels in MCF-7 and MB-231 BC cells, whereas overexpression of METTL3 increased m^6^A levels in these cells compared with control cells (Figure 1—figure supplement 1O, P).

**Figure 1—figure supplement 1.**
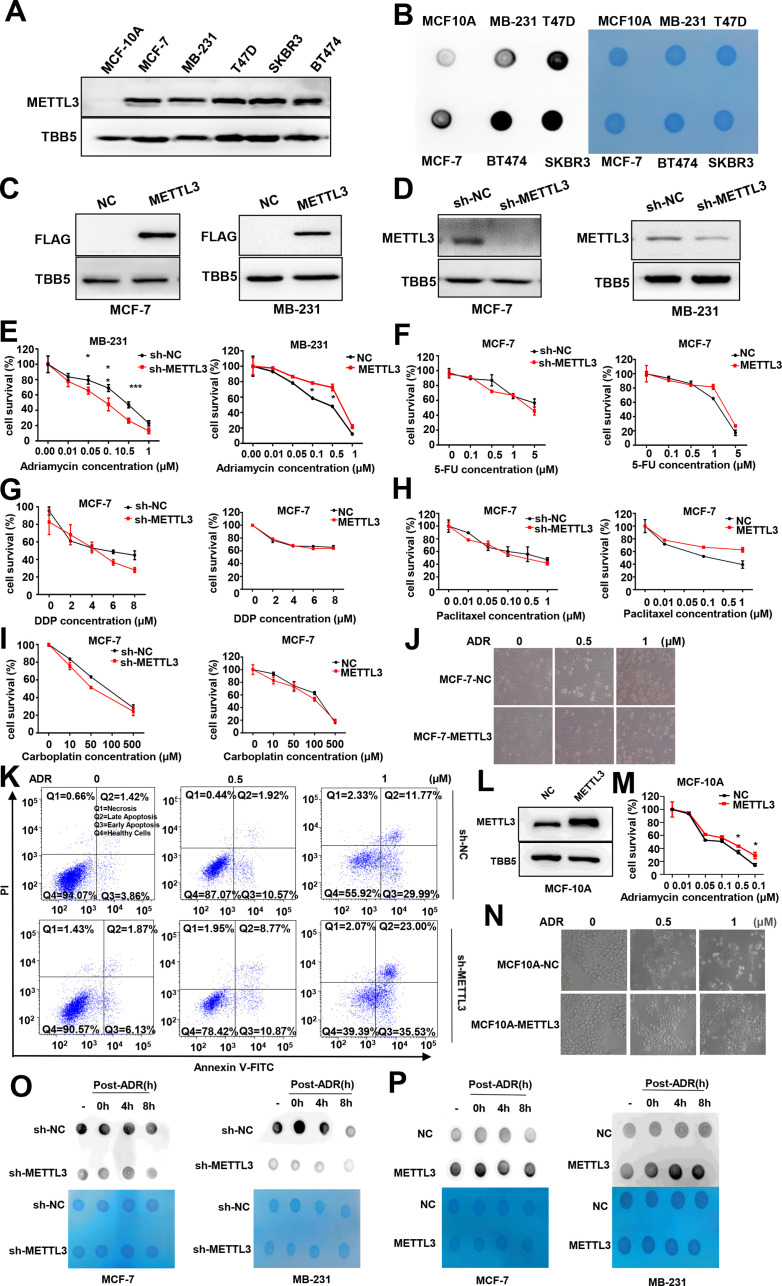
Knockdown of Methyltransferase-like 3 (METTL3) sensitizes MCF-7 and MB231 cells to Adriamycin (ADR). (**A**) Protein levels of METTL3 in breast cancer (BC) cells and normal breast cell (MCF-10A). (**B**) m^6^A modification in BC cells and normal breast cell. (**C, D**) Western blotting (WB) assay of METTL3 in the METTL3-OV stable cell lines (**C**) or METTL3-KD stable cell lines (**D**) in MCF-7 and MB231. (**E**) MTT assays using METTL3-KD and METTL3-OV MB-231 stable cell lines treated with ADR. Data are expressed as the mean ± standard deviation (SD), n=3 per group. (**F–I**) MTT assays using METTL3-KD and METTL3-OV MCF-7 stable cell lines treated with other four first-line chemotherapeutic drugs for BC treatment, including 5-FU (**F**), cisplatin (DDP) (**G**), paclitaxel (**H**), and carboplatin (**I**). Data are expressed as the mean ± standard deviation (SD), n=3 per group. (**J**) Morphological analysis of different treated MCF-7. (**K**) Flow cytometry analysis showed METTL3 deficiency increases ADR-induced cell apoptosis in MB231. (**L**) WB to detected METTL3 in METTL3-OV MCF-10A cells. (**M**) MTT assays in wildtype and METTL3-OV MCF-10A cells treated with ADR. Data are expressed as the mean ± standard deviation (SD), n=3 per group. (**N**) Morphological analysis of control and METTL3-OV MCF-10A cells treated with different dose of ADR. (**O, P**) Dot blot to detected the m^6^A level of poly(A)^+^ RNAs isolated from total RNA of METTL3-KD or METTL3-OV MCF-7, and MDA-MB231 cells with or without ADR treatment and recovery (0.5 μM ADR treatment for 1 hr and recovery for different time). Methylene blue staining served as a loading control. * p<0.05, ** p<0.01, *** p<0.001. Figure 1—figure supplement 1—source data 1.Source data for Figure 1—figure supplement 1A. Figure 1—figure supplement 1—source data 2.Source data for Figure 1—figure supplement 1B. Figure 1—figure supplement 1—source data 3.Source data for Figure 1—figure supplement 1C. Figure 1—figure supplement 1—source data 4.Source data for Figure 1—figure supplement 1D. Figure 1—figure supplement 1—source data 5.Source data for Figure 1—figure supplement 1L. Figure 1—figure supplement 1—source data 6.Source data for Figure 1—figure supplement 1O. Figure 1—figure supplement 1—source data 7.Source data for Figure 1—figure supplement 1P.

### METTL3 promotes HR

**Figure 2.**
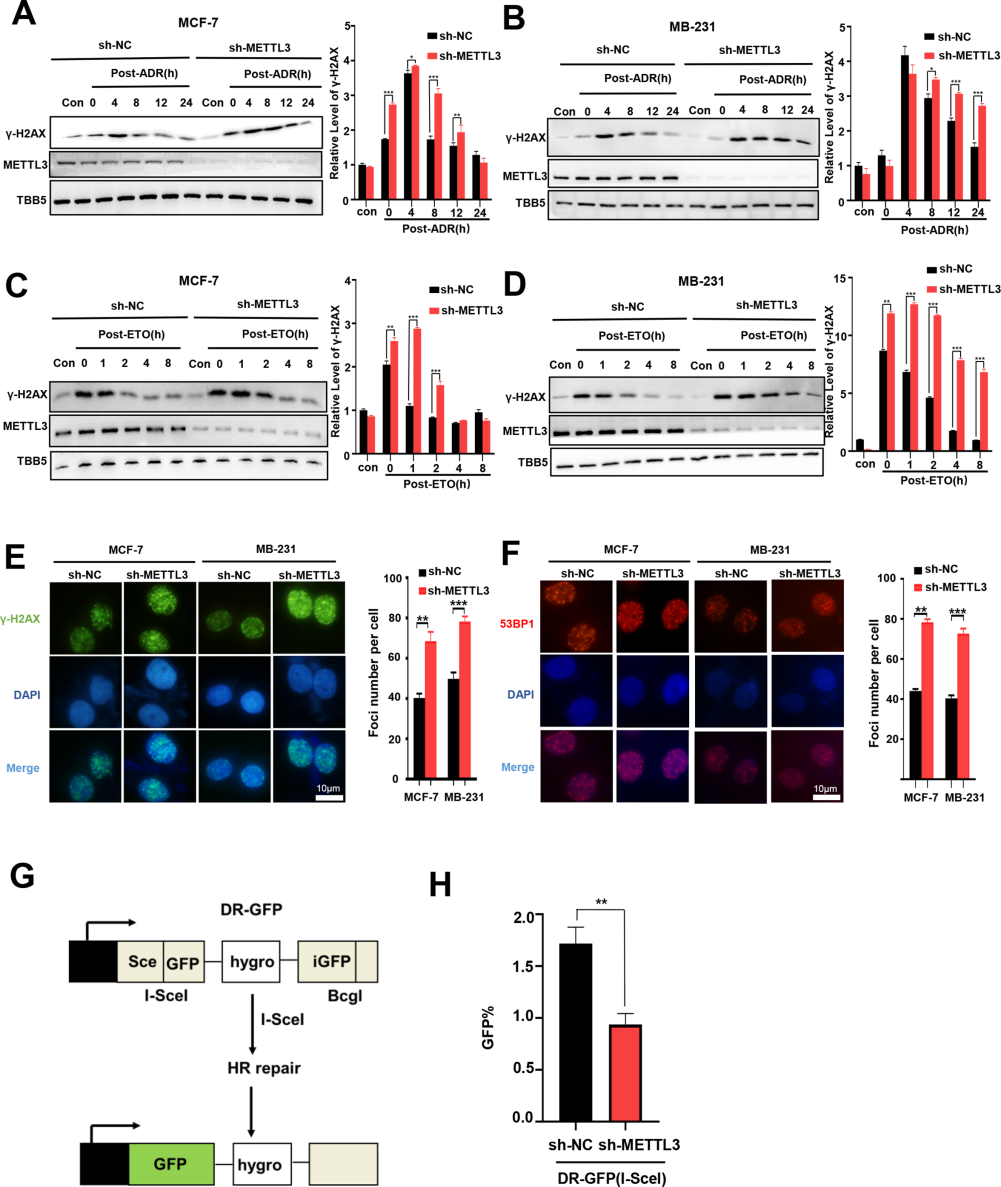
Knockdown of Methyltransferase-like 3 (METTL3) impairs homologous recombination repair (HR) efficacy. (**A, B**) Western blot (WB) assay to determine γ-H2AX levels in control and METTL3-KD MCF-7 cells (**A**) or MB-231 cells (**B**) with ADR (0.5 μM) treatment for 1 hr following different recovery times. (**C, D**) WB to determine γ-H2AX levels in control and METTL3-KD MCF-7 cells (**C**) or MB-231 cells (**D**) with ETO (10 μM) treatment for 1 hr following different recovery times. The Quantification of relative WB band are represented as the mean ± SD of three biological repeats. n=3 per group. (**E**) Immunofluorescence staining of γ-H2AX foci in different labeled cells with ADR treatment for 1 hr following 8 hr of recovery. (**F**) Immunofluorescence staining of 53BP1 foci in different cells treated same in (**E**). The quantification of average foci numbers per cells were showed in right panel, 50 cells were calculated in each group. (**G**) Schematic of GFP-based HR reporter system. (**H**) The GFP+ frequency of HR-mediated DSB repair in control and METTL3-KD U2OS cells (n=3 per group). ** p<0.01; *** p<0.001 (Student’s *t*-test). Figure 2—source data 1.Source data for Figure 2A. Figure 2—source data 2.Source data for Figure 2B. Figure 2—source data 3.Source data for Figure 2C. Figure 2—source data 4.Source data for Figure 2D.

ADR is normally described as a classic topoisomerase II poison that intercalates into DNA and forms DNA adducts, and subsequently induces DNA double strand breaks (DSBs) (Swift et al., 2006; Yang et al., 2014). We further addressed whether METTL3 was involved in DSB repair and affected ADR-induced DNA damage. To explore the role of METTL3 in the regulation of DNA repair, we treated METTL3-KD or –OV BC cells with ADR and subsequently released the cells into fresh medium lacking ADR and monitored the levels of γ-H2AX (an established marker of DNA damage) over time. Our data showed that knockdown of METTL3 maintained higher γ-H2AX levels in both MCF-7 and MB-231 cells compared with control cells (Figure 2A and B), whereas overexpression of METTL3 resulted in an earlier decline of γ-H2AX compared with control cells (Figure 2—figure supplement 1A, B). We further detected the effect of METTL3 on the regulation of DSB repair that was induced by etoposide (ETO; another inhibitor of topoisomerase II). Accordingly, our data showed that METTL3 promoted the repair of DSB induced by ETO in both MCF-7 and MB-231 cells (Figure 2C and D, Figure 2—figure supplement 1C, D). As showed in our data, the DNA damage was more rapid induced by etoposide than doxorubicin (Figure 2A–D), which was consistent with previous study in lung cancer cell lines (Binaschi et al., 1990). These results may due to a few differences among mechanisms of these two compounds poisoned DNA strands and cells, by which ADR induced cell death by trapping topoisomerase II, formation of ADR-DNA adducts, and generation of free radicals or ceramide production, whereas DNA damage secondary to topoisomerase II inhibition appears to be a major mechanism for etoposide-induced apoptosis (Yang et al., 2014; Yang et al., 2001). Consistently, an increased number of positive nuclei foci of γ-H2AX was detected in both METTL3-KD MCF-7 and MB-231 cells after ADR treatment and then released after 4 hr (Figure 2E), whereas experiments using METTL3-OV MCF-7 and MB-231 cells showed the opposite effects (Figure 2—figure supplement 1E). Since the phosphorylation and foci formation of γ-H2AX was the marker of both DNA damage and DNA replication stress, we also detected another DNA damage marker 53BP1 (tumor-suppressor p53-binding protein 1, a key regulator of DSB repair) foci (Gagou et al., 2010; Wang et al., 2002; Ward and Chen, 2001). Similar results were obtained for the foci of 53BP1 in both MCF-7 and MB-231 cells (Figure 2F and Figure 2—figure supplement 1F). DSBs are primarily repaired by either HR or non-homologous end joining (NHEJ) (Sonoda et al., 2006). Using two well-characterized green fluorescent protein (GFP)-based HR and NHEJ reporter systems, we determined the effect of METTL3 on HR and NHEJ efficiency (Mendez-Dorantes et al., 2020; Tsai et al., 2020). The results showed that overexpression of METTL3 significantly enhanced HR-mediated DSB repair, whereas knockdown of METTL3 decreased efficiency of HR (Figure 2G and H; and Figure 2—figure supplement 1G). No effect of METTL3 was observed on NHEJ-mediated DSB repair (Figure 2—figure supplement 1H, I). This is consistent with previous reports using the HR and NHEJ luciferase reporter system based on crisper-cas9-induced DSBs (Zhang et al., 2020).

**Figure 2—figure supplement 1.**
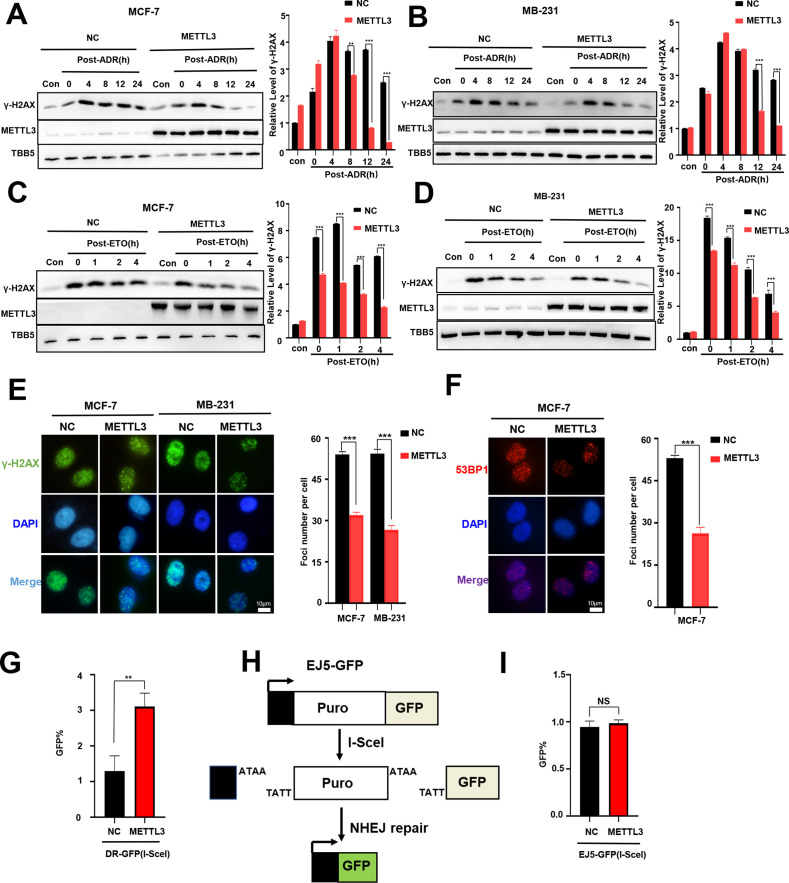
Methyltransferase-like 3 (METTL3) enhances homologous recombination repair (HR) activity. (**A and B**) Western blotting (WB) assay to determine γ-H2AX levels in control and METTL3-OV MCF-7 cells (**A**) or MB-231 cells (**B**) with ADR (0.5 μM) treatment for 1 hr following different time of recovery. (**C and D**) WB assay to determine γ-H2AX levels in control and METTL3-OV MCF-7 cells (**C**) or MB-231 cells (**D**) with ETO (10 μM) treatment for 1 hr following different time of recovery. The Quantification of relative WB band are represented as the mean ± SD of three biological repeats. (**E**) Immunofluorescence staining of γ-H2AX foci in METTL3-OV MCF-7 and MB-231 cells with ADR treatment for 1 hr following 4 hr recovery. (**F**) 53BP1 foci in METTL3-OV MCF-7 cells. The quantification of average foci numbers per cells were showed in right panel, 50 cells were calculated in each group. (**G**) The GFP+ frequency of HR-mediated DSB repair in control and METTL3-OV U2OS cells (n=3 per group). (**H**) Schematic of GFP-based NHEJ reporter system. (**I**) Relative quantification of the frequency of NHEJ-mediated DSBR in METTL3-ovexpressing U2OS cells. n=3 per group. ** p<0.01; *** p<0.001 (Student’s *t*-test). Figure 2—figure supplement 1—source data 1.Source data for Figure 1—figure supplement 1A. Figure 2—figure supplement 1—source data 2.Source data for Figure 2—figure supplement 1B. Figure 2—figure supplement 1—source data 3.Source data for Figure 2—figure supplement 1C. Figure 2—figure supplement 1—source data 4.Source data for Figure 2—figure supplement 1D.

### EGF is the target of METTL3 and is regulated by m^6^A modification

**Figure 3.**
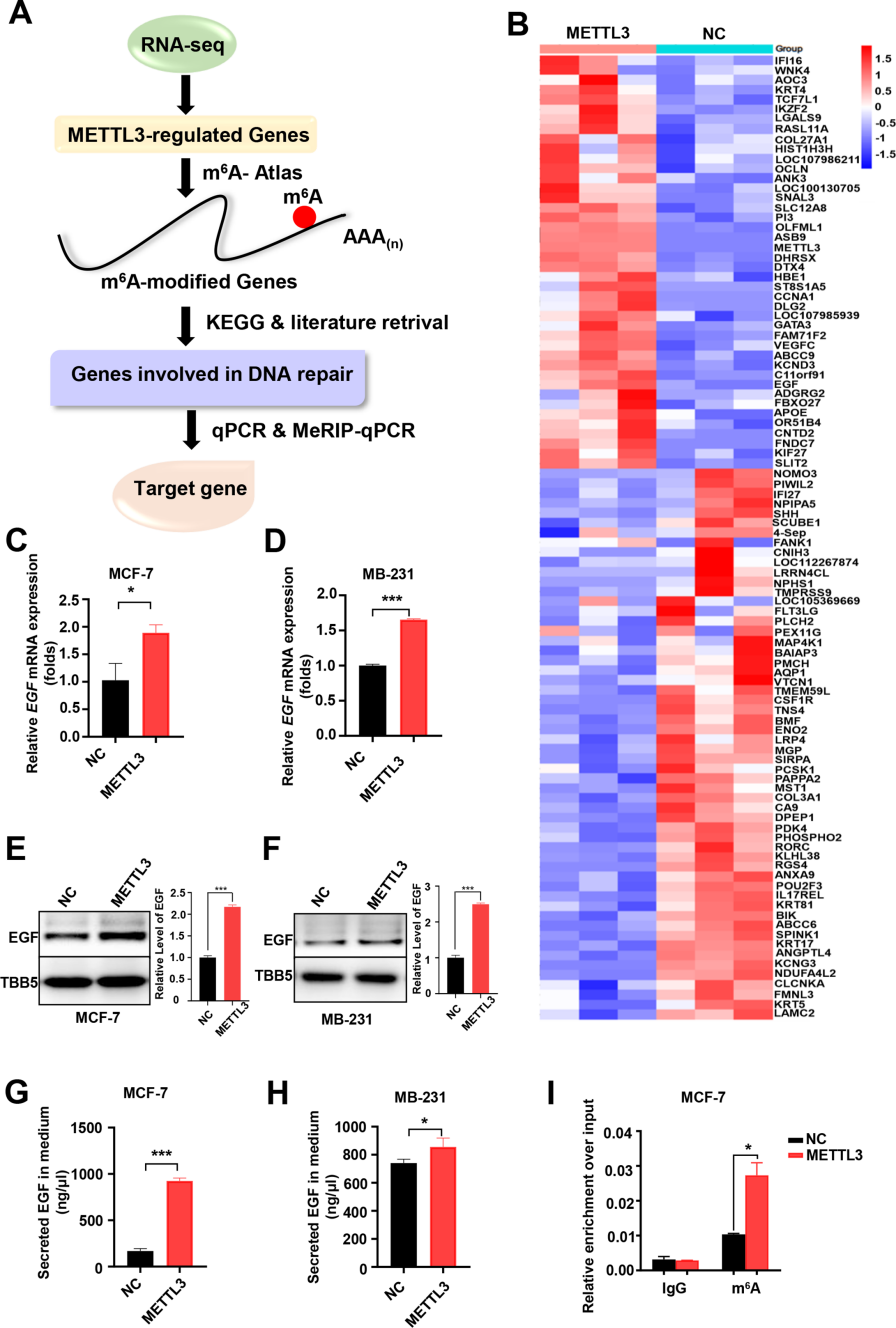
Screening and Identification of EGF as the targets of Methyltransferase-like 3 (METTL3) in breast cancer (BC). (**A**) Schematic of the screening progress of METTL3 targets in BC. (**B**) Heat map of RNA-seq to identify the genes regulated by METTL3 overexpression. The value presented Log_2_ (fold change). (**C, D**) qRT-PCR was performed in METTL3-overexpressing MCF-7 (**C**) and MB-231 cells (**D**) to detect EGF expression. Data are expressed as the mean ± standard deviation (SD), n=3 per group. (**E, F**) Western blot (WB) analysis of EGF expression in METTL3-overexpressing MCF-7 (**E**) and MB-231 cells (**F**). (**G, H**) ELISA assay measuring secreted EGF in the medium of METTL3-overexpressing MCF-7 (**G**) and MB-231 cells. (**H**) Data are expressed as the mean ± standard deviation (SD), n=3 per group. (**I**) MeRIP-qPCR analysis was used to assess the m^6^A levels of EGF mRNA in METTL3-overexpressing MCF-7 cells. The enrichment of m^6^A in each group was calculated by m^6^A-IP/input and IgG-IP/input. Data are expressed as the mean ± standard deviation (SD), n=3 per group. * p<0.05; *** p<0.001 (Student’s *t*-test). Figure 3—source data 1.Source data for Figure 3E. Figure 3—source data 2.Source data for Figure 3F.

A comprehensive assay combined with RNA-seq, MeRIP-qPCR, bioinformatics analysis, and literature retrieval were designed to explore the putative target(s) of METTL3-mediated m^6^A modification, which is involved in the regulation of both DNA repair and BC sensitivity to ADR (Figure 3A). A total of 98 genes showed significant changes (p<0.05; 42 up-regulated; and 56 down-regulated) in METTL3-ovexpressing MCF-7 cells compared with control MCF-7 cells (Figure 3B). Among these genes, 52 were shown to be modified by m^6^A in the exonic, 5’UTR, or 3’UTR of mRNA region in the m^6^A-Atlas, a comprehensive knowledgebase for unraveling the m^6^A epitranscriptome (Table 1; Tang et al., 2021). Furthermore, literature retrieval identified 8 (Table 2) out of 52 genes that were reported to be involved in the regulation of DNA repair, among which EGF was highlighted because of its role in cancer progression, and DNA repair (Myllynen et al., 2011; Wilson et al., 2009; Yacoub et al., 2003). Next, we verified the expression of EGF regulated by METTL3. The mRNA levels of EGF increased in both METTL3-OV MCF-7 and MDA-MB231 cells compared with control cells (Figure 3C and D), whereas knockdown of METTL3 down-regulated EGF expression (Figure 3—figure supplement 1A, B). WB analysis of whole cell lysates further verified the up-regulation of EGF by METTL3 (Figure 3E and F, and Figure 3—figure supplement 1C, D). Moreover, secreted EGF in the culture supernatants were examined by ELISA. The results indicated increased EGF levels in the medium of METTL3-OV MCF-7 and MB-231 cells (Figure 3G and H), whereas down-regulated EGF was detected in the medium of METTL3-KD cells (Figure 3—figure supplement 1E, F ). Overexpression of METTL3 also enhanced the expression of EGF in MCF-10A cells (Figure 3—figure supplement 1G, H). Furthermore, to validate the m^6^A modification in EGF mRNA, we performed a methylated RNA immunoprecipitation (meRIP)-qPCR assay using an m^6^A antibody followed by qPCR for the predicted region of the m^6^A sites. EGF mRNA exhibited the highest score by the sequence-based RNA adenosine methylation site predictor algorithm (Zhou et al., 2016). Using specific primers designed for the predicted m^6^A-harboring regions of EGF, the qPCR data showed that overexpression of METTL3 upregulated the m^6^A modification of EGF mRNA (Figure 3I and Figure 3—figure supplement 1I), whereas knockdown of METTL3 significantly attenuated its m^6^A levels (Figure 3—figure supplement 1J). Our data indicated that EGF was the target of METTL3 and regulated by METTL3-mediated m^6^A modification in both BC cell line and non-tumorigenic breast cell line.

**Figure 3—figure supplement 1.**
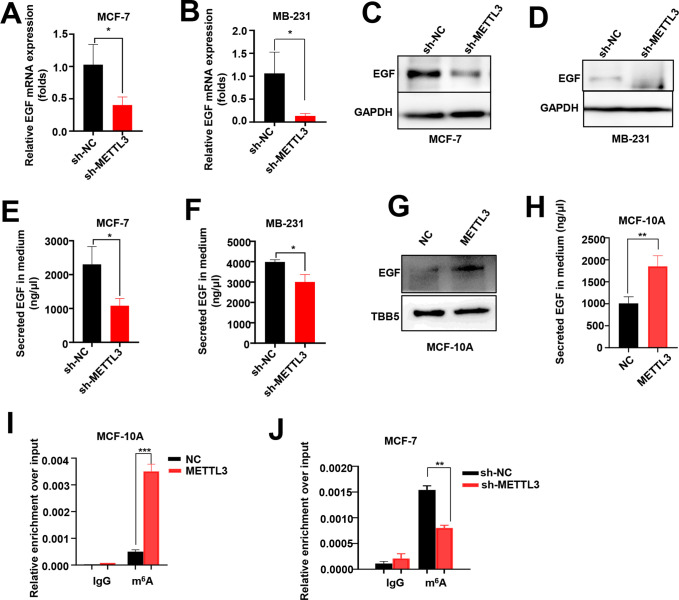
EGF is the targets of Methyltransferase-like 3 (METTL3) in MCF-7, MB-231, and MCF-10A cells. (**A, B**) RT-qPCR was performed in METTL3-KD MCF-7 (**A**) and MB-231 cells (**B**) to detect the EGF mRNA expression. Data are expressed as the mean ± standard deviation (SD), n=3 per group. (**C, D**) Western blotting (WB) analysis of the EGF expression in METTL3-KD MCF-7 (**C**) and MB-231 cells (**D**). (**E, F**) ELISA assay to test the secreted EGF in the medium of METTL3-KD MCF-7 (**E**) and MB-231 cells (**F**). Data are expressed as the mean ± standard deviation (SD), n=3 per group. (**G**) WB showed that EGF was upregulated in METTL3-OV MCF-10A cells. (**H**) Secreted EGF increased in METTL3-OV MCF-10A cells. Data are expressed as the mean ± SD, n=3 per group. (**I**) MeRIP-qPCR analysis showed the upregulation of m^6^A levels of EGF mRNA in METTL3-OV MCF-10A cells. (**J**) Decreased m^6^A levels of EGF mRNA in METTL3-KD MCF-7 cells. Data are expressed as the mean ± standard deviation (SD), n=3 per group. * p<0.05; ** p<0.01; *** p<0.001 (Student’s *t*-test). Figure 3—figure supplement 1—source data 1.Source data for Figure 3—figure supplement 1C. Figure 3—figure supplement 1—source data 2.Source data for Figure 3—figure supplement 1D. Figure 3—figure supplement 1—source data 3.Source data for Figure 3—figure supplement 1G.

**Table 1.** 52 genes were showed to be modified by m^6^A in the exonic in this study.

KCNG3	DLG2	SLC12A8	PHOSPHO2	AQP1	ABCC6
RGS4	C11orf91	GATA3	RORC	SIRPA	COL3A1
ASB9	HIST1H3H	IFI16	KRT17	BMF	
ST8SIA5	FBXO27	RASL11A	LAMC2	BIK	
METTL3	SLIT2	DTX4	TMPRSS9	SHH	
ABCC9	AOC3	ANK3	PLCH2	NOMO3	
APOE	COL27A1	KIF27	BAIAP3	NPIPA5	
EGF	OCLN	ENO2	FMNL3	MST1	
IKZF2	PI3	PCSK1	LRP4	PMCH	
DHRSX	VEGFC	TNS4	PDK4	CSF1R	

**Table 2.** Eight genes were showed to be modified by m^6^A and be involved in DNA repair in this study.

EGF	METTL3	DLG2	VEGFC
**GATA3**	**KRT17**	**IFI16**	**MST1**

### EGF regulates RAD51 expression and enhances HR activity

**Figure 4.**
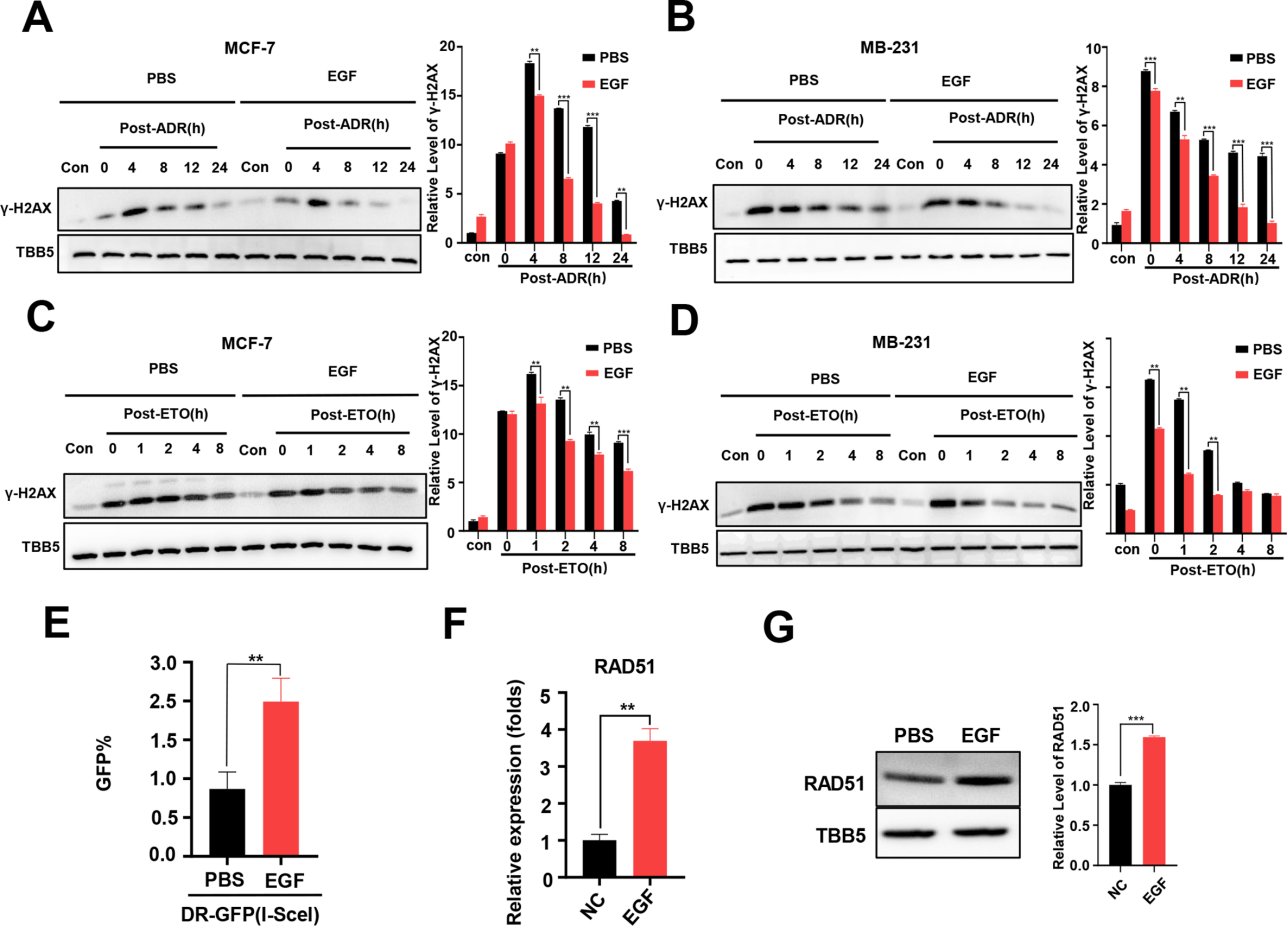
Additional EGF promotes RAD51 expression and enhances homologous recombination repair (HR) activity. (**A, B**) Western blot (WB) assay identified that EGF (10 ng/ml) enhanced DNA repair in MCF-7 (**A**) and MB-231 cells (**B**) treated with ADR (0.5 μM) (shown by lower γ-H2AX in EGF treated samples compare to PBS treated samples). (**C, D**) EGF (10 ng/ml) enhanced DNA repair in MCF-7 (**C**) and MB-231 cells (**D**) treated with ETO (10 μM). The Quantification of relative WB band are represented as the mean ± SD of three biological repeats. (**E**) The GFP+ frequency of HR reporter assay in the treatment with EGF (10 ng/ml for 4 hr) or vehicle. Data are expressed as the mean ± standard deviation (SD), n=3 per group. (**F, G**) EGF augmented RAD51 mRNA (**F**) and protein (**G**) expression in MCF-7 cells. Data are expressed as the mean ± standard deviation (SD), n=2 per group. ** p<0.01 (Student’s *t*-test). Figure 4—source data 1.Source data for Figure 4A. Figure 4—source data 2.Source data for Figure 4B. Figure 4—source data 3.Source data for Figure 4C. Figure 4—source data 4.Source data for Figure 4D. Figure 4—source data 5.Source data for Figure 4G.

The EGF/EGFR signaling pathway has been reported to regulate DSB repair in lung cancer cells following X-irradiation by promoting both the NHEJ and HR pathways (Kriegs et al., 2010; Myllynen et al., 2011). Thus, we wondered whether EGF/EGFR is involved in METTL3-mediated DSB repair in both MCF-7 and MB-231 cells treated with chemotherapeutic agents, such as ADR. First, we evaluated the effect of the EGF/EGFR signaling pathway on DSB repair in these BC cells. MCF-7 and MB-231 cells were treated with ADR or ETO, respectively, and then released for different times with EGF treatment. WB analysis showed that the γ-H2AX levels markedly decreased in both ADR- and ETO-treated cells following EGF treatment, indicating that EGF enhanced DSB repair (Figure 4A–D). Using a GFP-based HR reporter system, we detected the effect of the EGF/EGFR pathway on regulating HR activity. Our data showed that additional EGF enhanced HR activity in the reporter system (Figure 4E), whereas the EGFR inhibitors erlotinib and gefitinib could inhibited the HR activity with lower GFP-positive cells compared to vehicle treatment (Figure 4—figure supplement 1A, B). To explore the molecular mechanism of EGF-mediated HR, we determined whether EGF/EGFR regulated the expression of core genes involved in HR, including *BRCA1*, *BRCA2*, *CtIP*, and *RAD51*. Our data showed that both EGF and METTL3 exhibited a slight effect on the regulation of BRCA1, BRCA2, and CtIP expression (Figure 4—figure supplement 1C, D). In contrast, the expression of RAD51 was markedly regulated by the EGF/EGFR signaling pathway (Figure 4F and G, and Figure 4—figure supplement 1).

**Figure 4—figure supplement 1.**
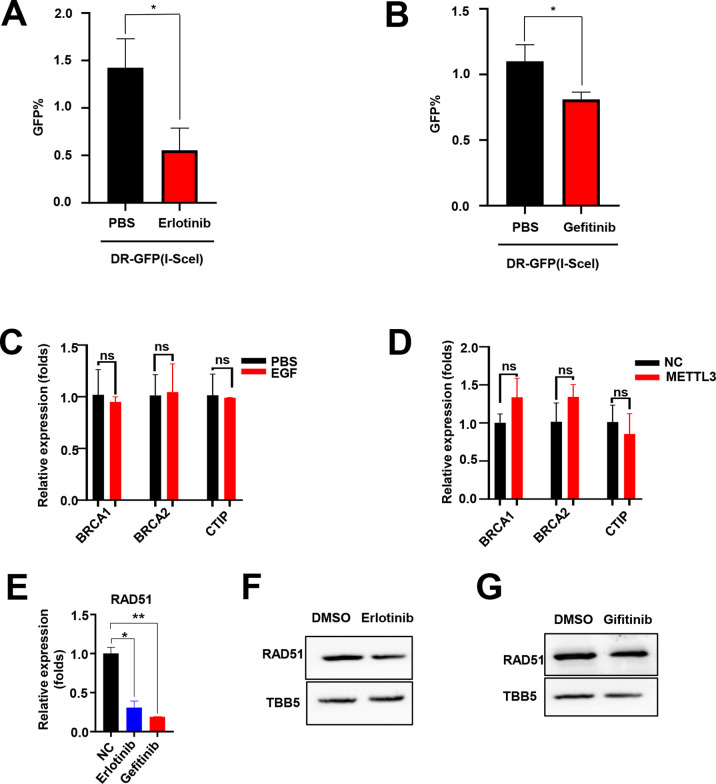
EGF promotes RAD51 expression and enhances homologous recombination repair (HR) efficiency. (**A and B**) EGFR inhibitor (Erlotinib and Gefitinib) reduced HR efficacy in GFP-based HR reporter system. n=3 per group. EGF (**C**) or METTL3 (**D**) regulated the mRNA levels of *BRCA1*, *BRCA2*, and *CtIP*. Data are expressed as the mean ± standard deviation (SD), n=3 per group. (**E**) Erlotinib and Gefitinib reduced the mRNA levels of *RAD51*. Data are expressed as the mean ± SD, n=3 per group. (**F–G**) Erlotinib (**F**) and Gefitinib (**G**) reduced the protein levels of RAD51 in MCF-7 cells. * p<0.05 (Student’s *t*-test). Figure 4—figure supplement 1—source data 1.Source data for Figure 4—figure supplement 1F. Figure 4—figure supplement 1—source data 2.Source data for Figure 4—figure supplement 1G.

### METTL3-modification of DNA repair is EGF/ RAD51 dependent

**Figure 5.**
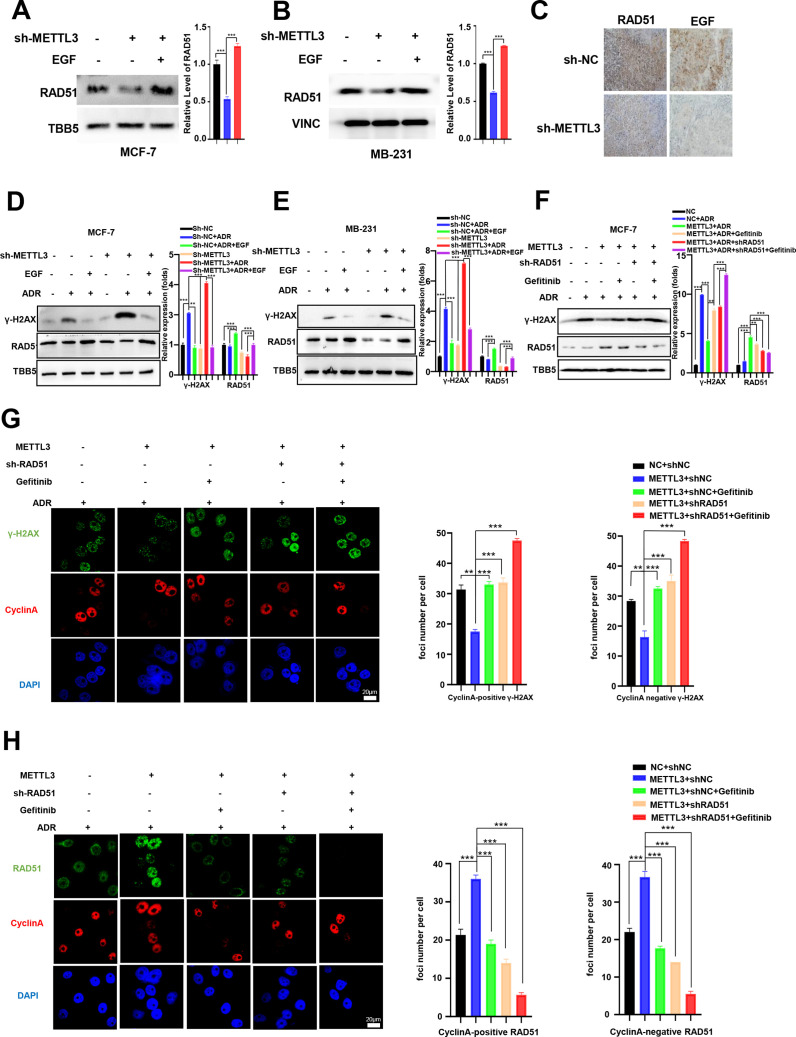
Methyltransferase-like 3 (METTL3) promotes DNA repair via EGF/Rad51 axis. (**A, B**) RAD51 protein levels in METTL3-KD MCF-7 (**A**) and MB-231 (**B**) cells treated with or without EGF. (**C**) Immunohistochemistry analysis of the expression of EGF and RAD51 in control and METTL3-KD tumor tissues. (**D, E**) WB analysis showing that treatment with 10 ng/ml EGF for 8 hr restores DNA repair activity in METTL3-KD MCF-7 (**D**) and MB-231 (**E**) cells. (**F**) WB analysis showing that knocking down RAD51 or EGFR inhibitor Gefitinib (10 nM for 8 hr) treatment in METTL3-OV cells decreases METTL3-enhanced DNA repair activity. The Quantification of relative WB band are represented as the mean ± SD of three biological repeats. (**G**) Immunofluorescence analysis of co-staining of γ-H2AX and cyclin A in METTL3-OV cells ±RAD51 shRNA or gefitinib (10 nM) during ADR treatment (0.5 μM for 1 hr and recovery for 8 hr without ADR). The quantitative assay is on the right. n=3 per group. (**H**) Immunofluorescence analysis of cyclin A and RAD51 foci in cells treated the same as in (**G**). The quantification of average foci numbers per cells were showed in right panel, 50 cells were calculated in each group. ** p<0.01; *** p<0.001 (Student’s *t*-test). Figure 5—source data 1.Source data for Figure 5A. Figure 5—source data 2.Source data for Figure 5B. Figure 5—source data 3.Source data for Figure 5D. Figure 5—source data 4.Source data for Figure 5E. Figure 5—source data 5.Source data for Figure 5F.

We next explored the effect of EGF/EGFR signaling on METTL3-mediated HR in MCF-7 and MB-231 cells. WB analysis showed that knockdown of METTL3 down-regulated RAD51 expression, which was recovered by EGF treatment in both MCF-7 and MB-231 cells (Figure 5A and B). In contrast, overexpression of METTL3 up-regulated RAD51 expression, which was repressed by the EGFR inhibitors, erlotinib and gefitinib (Figure 5—figure supplement 1A, B ). Both EGF and RAD51 levels were down-regulated in xenograft tissues derived from METTL3-KD MCF-7 cells (Figure 5C). Accordingly, EGF treatment impeded METTL3-KD-mediated repression of DNA repair in both MCF-7 and MB-231 cells (Figure 5D and E). The EGFR inhibitor, erlotinib, repressed DNA repair activities that were up-regulated by overexpression of METTL3 in both BC cells (Figure 5—figure supplement 1C, D). These data indicate that the EGF/EGFR signaling pathway regulates RAD51 expression and participates in METTL3-mediated HR.

**Figure 5—figure supplement 1.**
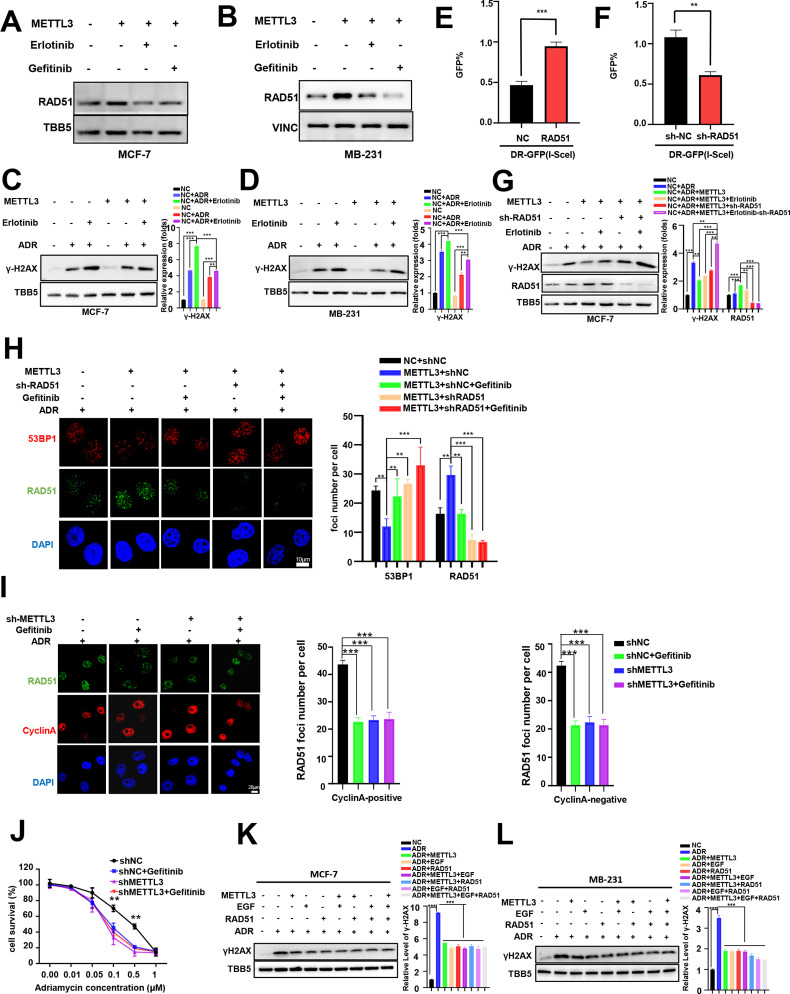
METTL3-mediated DNA repair is EGF/ Rad51 dependent. (**A, B**) Western blotting (WB) showed that overexpression of METTL3 upregulated RAD51 expression, which were abolished by EGFR inhibitors erlotinib (**A**) and gefitinib (**B**). (**C–D**) Erlotinib repressed DNA repair activities that were upregulated by overexpression of METTL3 in MCF-7 (**C**) and MB231 (**D**) cells. (**E, F**) Overexpression of RAD51 (**E**) or knockdown of RAD51 (**F**) enhanced or impaired GFP+ frequencies in GFP-base HR reporter system, respectively. (**G**) Overexpression of METTL3 promoted DNA repair efficacy (shown by γ-H2AX down-regulation) that were reversed by treatment with shRAD51 or Erlotinib. (**H**) Immunofluorescence analysis of 53BP1 foci was performed in detecting MCF-7 cells, which were treated as showed in the figures. Cells were stained with an anti-RAD51 and anti-53BP1 antibody, and nucleus was stained using DAPI and then visualized by a fluorescence microscope. (**I**) Immunofluorescence analysis showed that RAD51 foci decreased to similar levels in cells with single inhibition of METTL3 by shRNA or single inhibition of EGF/EGFR by gefitinib or double treatments with shMETTL3 and gefitinib. The quantification of average foci numbers per cells were showed in right panel, 50 cells were calculated in each group. (**J**) Cells survival assay in the condition as same as in (**I**). (**K, L**) WB showed that overexpression of METTL3/EGF/RAD51 all could alleviate DNA damage (shown by decreased γ-H2AX) in both MCF-7 (**K**) and MB-231 cells (**L**) with similar manner. The Quantification of relative WB band are represented as the mean ± SD of three biological repeats. p<0.01; *** p<0.001 (Student’s *t*-test). Figure 5—figure supplement 1—source data 1.Source data for Figure 5—figure supplement 1A. Figure 5—figure supplement 1—source data 2.Source data for Figure 5—figure supplement 1B. Figure 5—figure supplement 1—source data 3.Source data for Figure 5—figure supplement 1C. Figure 5—figure supplement 1—source data 4.Source data for Figure 2—figure supplement 1D. Figure 5—figure supplement 1—source data 5.Source data for Figure 5—figure supplement 1G. Figure 5—figure supplement 1—source data 6.Source data for Figure 5—figure supplement 1K. Figure 5—figure supplement 1—source data 7.Source data for Figure 2—figure supplement 1L.

We next investigated whether the effects of METTL3 on DNA repair were EGF/RAD51 dependent. We first confirmed the effect of RAD51 on HR activity in GFP-based HR reporter system as showed in Figure 2G. Our data showed that overexpression of RAD51 promoted HR activity, whereas knockdown of RAD51 repressed HR efficiency in the reporter system (Figure 5—figure supplement 1E, F), which were consistent with other current studies (Asan et al., 2019; Ouyang et al., 2021). Then, we knocked down of RAD51 in METTL3-OV MCF-7 cells. The METTL3-OV MCF-7 cells were transfected with shRAD51 (shRNA of RAD51) for 36 hr, then treated with ADR for 1 hr and released for 8 hr with or without gefitinib/erlotinib treatment. Overexpression of METTL3 resulted in elevated DNA repair activity (shown by γ-H2AX down-regulation) which was reversed by treatment with siRAD51 or gefitinib/erlotinib (Figure 5F, and Figure 5—figure supplement 1G). We further detected the γ-H2AX foci and RAD51 foci in different treated cells by immunofluorescence. We stained Cyclin A to represent S/G2 phase cells. Consistently, immunofluorescence analysis showed that the γ-H2AX foci were down-regulated by overexpression of METTL3, which were reversed by treatment with shRAD51 or gefitinib in both cyclin A positive cells and cyclin A negative cells (Figure 5G). The 53BP1 foci showed similar partners to γ-H2AX foci in these different treated cells (Figure 5—figure supplement 1H). The RAD51 foci were also augmented by overexpression of METTL3 and reversed with gefitinib treatment in both cyclin A positive cells and cyclin A negative cells (Figure 5H). Moreover, we detected RAD51 foci in cells with single inhibition of METTL3 by shRNA or single inhibition of EGF/EGFR by gefitinib or double treatments with shMETTL3 and gefitinib. We found that the RAD51 foci decreased to similar levels in cells in these three conditions compared to those in control cells, which indicated an epistatic effect of shMETTL3 and EGF/EGFR inhibition (Figure 5—figure supplement 1I). This epistatic effect was verified by cells survival assay (Figure 5—figure supplement 1J). Furthermore, we detected γ-H2AX levels in the ADR-treated MCF-7 and MB-231 cells with overexpression of METTL3 or EGF or RAD51. Our data showed that overexpression of METTL3/EGF/RAD51 all could alleviate DNA damage (shown by decreased γ-H2AX) in both MCF-7 and MB-231 cells with similar manner (Figure 5—figure supplement 1K, L). These results suggest that METTL3 augments HR in ADR-treated BC cells via the EGF/ RAD51 axis.

### YTHDC1 enhances the METTL3/m^6^A-regulated EGF/Rad51 axis

**Figure 6.**
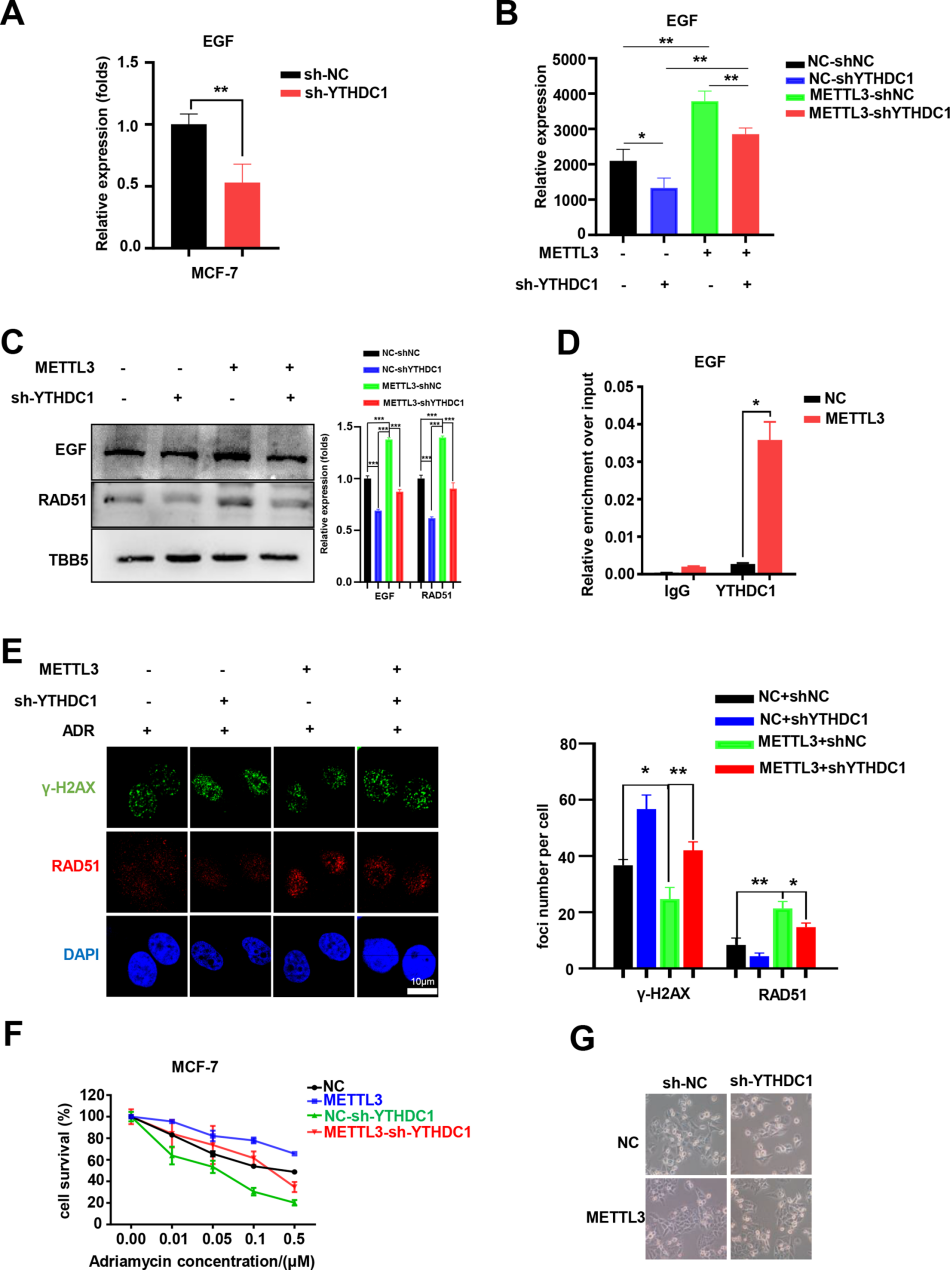
YTHDC1 is the ‘reader’ of the METTL3/m^6^A-regulated EGF/RAD51 axis. (**A**) The expression of EGF in YTHDC1-silenced MCF-7 cells was detected by qRT-PCR. Data are expressed as the mean ± standard deviation (SD), n=3 per group. (**B**) The mRNA levels of EGF in control or METTL3-OV cells with or without knocking down YTHDC1. Data are expressed as the mean ± standard deviation (SD), n=3 per group. (**C**) WB assay determining the effect of YTHDC1 knockdown on EGF and RAD51 expression in control and METTL3-OV MCF-7 cells. The Quantification of relative WB band are represented as the mean ± SD of three biological repeats. (**D**) RIP-qPCR assay showing the enrichment of the EGF transcript in METTL3-OV cells. Data are expressed as the mean ± SD, n=3 per group. (**E**) Immunofluorescence analysis of γ-H2AX and RAD51 foci in METTL3-OV cells with knocked-down YTHDC1. The quantification of average foci numbers per cell are shown in the right panel, 50 cells were calculated in each group. (**F**) MTT assays were performed to detect the effect of YTHDC1 knockdown on ADR sensitivity in control and METTL3-OV MCF-7 cells. Data are expressed as the mean ± standard deviation (SD), n=3 per group. (**G**) Morphological analysis of control or METTL3-OV MCF-7 cells with or without YTHDC1 knockdown. Cells were treated with 0.5 μM ADR for 24 hr. * p<0.05; ** p<0.01; *** p<0.001 (Student’s *t*-test). Figure 6—source data 1.Source data for Figure 6C.

There are two major families of m^6^A ‘readers’ that play a specific role in controlling the fate of methylated mRNA including the YTH family and the IGF2BP family (Deng et al., 2018; Wang et al., 2015; Xiao et al., 2016). To elucidate the specific m^6^A readers of EGF mRNA and to determine the m^6^A-dependent mechanism of EGF regulation, we performed qPCR assays to screen EGF-related m^6^A readers. Interestingly, knockdown of YTHDC1, but not other members of the YTH family or the IGF2BP family, down-regulated both EGF and RAD51 in MCF-7 cells (Figure 6A and Figure 6—figure supplement 1A-C). Furthermore, knockdown of YTHDC1 reversed the METTL3-mediated up-regulation of EGF and RAD51 (Figure 6B and C). The potential binding motif of YTHDC1 in EGF mRNA is UGG(m^6^A)CU, which is the preferentially binding motif of YTHDC1 (Xu et al., 2014). Using RIP-qPCR, we found that the direct interaction between YTHDC1 and EGF transcripts was enhanced in METTL3-OV cells compared with that in control cells (Figure 6D), whereas the interaction between YTHDC1 and EGF transcripts was down-regulated in the condition of shMETTL3 (Figure 6—figure supplement 1D). Furthermore, the YTHDC1 deficiency impaired the outcome of down-regulation of γ-H2AX foci in METTL3-OV MCF-7 cells (Figure 6E). An MTT assay revealed that YTHDC1 depletion rendered MCF-7 cells more sensitive to ADR and reversed METTL3-induced ADR resistance in METTL3-overexpressing cells (Figure 6F). These results were verified by morphological analysis (Figure 6G). Moreover, cell survival assay identified that double knockdown of METTL3 and YTHDC1 enhanced the sensitivity of MCF-7 to ADR, which is similar to those of single knockdown of METTL3 or YTHDC1 (Figure 6—figure supplement 1E). This result indicated an epistatic effect between shMETTL3 and shYTHDC1 on ADR response in MCF-7 cells. Taken together, our results suggest that YTHDC1 functions as an m^6^A ‘reader’ to enhance EGF mRNA stability and augment HR and cell survival in ADR-treated BC cells.

**Figure 6—figure supplement 1.**
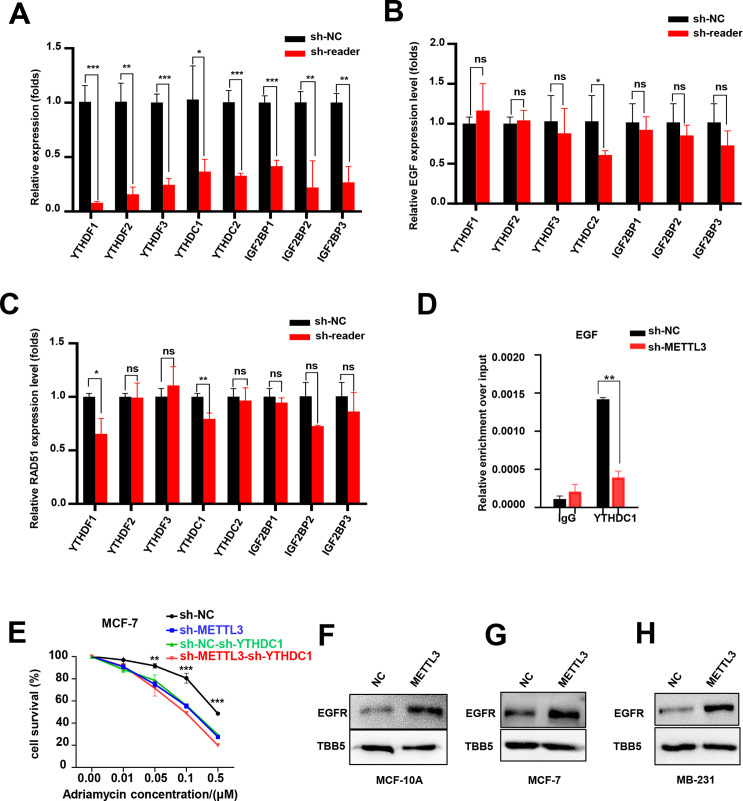
YTHDC1 was the reader of METTL3/m6A-regulated EGF. (**A**) mRNA levels of different readers in m^6^A readers -silencing MCF-7 cells was detected using RT-qPCR. (**B, C**) EGF and RAD51 mRNA levels in m^6^A readers -silencing MCF-7 cells using RT-qPCR. Data are expressed as the mean ± standard deviation (SD), n=3 per group. (**D**) RIP-qPCR assay showing the enrichment of the EGF transcript in METTL3-OV cells. Data are expressed as the mean ± standard deviation (SD), n=3 per group. (**E**) Cell survival assay in the cells in the condition of shMETTL3, shYTHDC1 or double KD of METTL3, and YTHDC1. (**F–H**) WB analysis showed that overexpression of METTL3 promoted EGFR expression in MCF-10A, MCF-7, and MB-231 cells. * p<0.05; ** p<0.01; *** p<0.001 (Student’s *t*-test). Figure 6—figure supplement 1—source data 1.Source data for Figure 6—figure supplement 1F. Figure 6—figure supplement 1—source data 2.Source data for Figure 6—figure supplement 1G. Figure 6—figure supplement 1—source data 3.Source data for Figure 6—figure supplement 1H.

## Discussion

**Figure 7.**
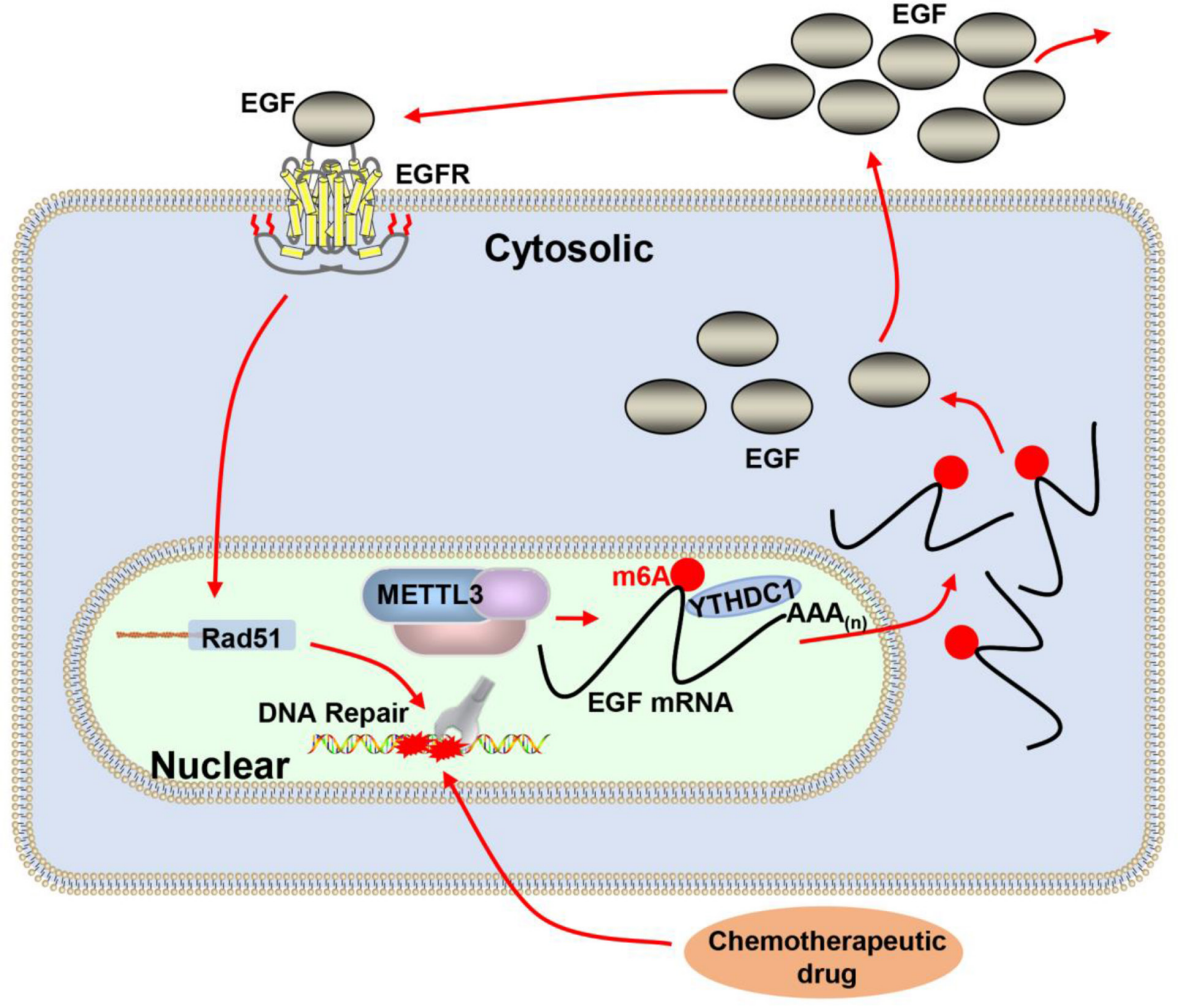
Proposed schematic diagram of the proposed mechanism elucidated in this study. Methyltransferase-like 3 (METTL3) augments EGF transcript m^6^A modification, which was recognized by the reader YTHDC1, resulting increase of EGF expression. Elevated EGF promoted RAD51 expression, enhanced homologous recombination repair (HR) efficacy, and modified chemotherapeutic response of cancer cells.

A deficiency of DNA repair proteins is associated with carcinogenesis and elevated DNA repair activity contributes to drug resistance in cancer (Li et al., 2021; Lu et al., 2020). MELLT3 and its regulated m^6^A modification have been reported to be involved in DNA repair (Xiang et al., 2017; Zhang et al., 2020). However, the potential mechanism of METTL3 in DNA repair and chemotherapeutic response is poorly defined. In the present study, we demonstrated that m^6^A RNA methylation levels and METTL3 expression were elevated in five different kinds of BC cells. Biochemical and cell biological analysis revealed that inhibition of METTL3 sensitizes both ER-positive and triple-negative BC cells to ADR treatment with elevated DNA damage. Knockdown of METTL3 impaired HR activity and increased ADR-induced DNA damage through modification of the EGF/RAD51 axis. METTL3 promoted HR through m^6^A-dependent upregulation of EGF expression, which further augmented RAD51 expression. The m^6^A ‘reader,’ YTHDC1, bound to the m^6^A-modified EGF transcript, protected EGF mRNA, and enhanced EGF expression (Figure 7). This result was consistent with other studies showing that YTHDC1 is recruited to sites of DNA damage, bound m^6^A RNA, and increased the activity of DSB repair (Yu et al., 2021a; Zhang et al., 2020).

Recently, RNA m^6^A modification and the core RNA methyltransferase, METTL3, were reported to play an important role in cancer chemotherapy (Deng et al., 2018). METTL3 was implicated in ADR resistance in MCF-7 cells by regulating the miR-221–3 p/HIPK2/Che-1 axis (Pan et al., 2021). Our data further demonstrated that METTL3 knockdown markedly sensitized both MCF-7 and MB-231 cells to ADR. ADR is one of the most effective antitumor agents for BC treatment, although it is limited by severe side effects. Various mechanisms have been proposed to explain ADR-induced cell death, including trapping topoisomerase II, formation of ADR-DNA adducts, and generation of free radicals that increase oxidative stress, which induce DNA damage and result in cell death (Yang et al., 2014). We also showed that a deficiency in METTL3 inhibits DNA repair and increases the accumulation of DNA damage in ADR-treated MCF-7 and MB-231 cells, which further leads to cell death.

A large number of studies have implicated dysfunctional DNA repair proteins, such as BRCA1, RAD51, FEN1, and Polβ, in BC initiation and progression (Li et al., 2021; Lu et al., 2020; Martin et al., 2007; Thacker, 2005; Wang et al., 2019; Wang et al., 2020b; Xia et al., 2021). Elevated DNA repair activity contributes to drug resistance and limits the efficacy of chemotherapeutic agents (Long et al., 2021; Lu et al., 2020). Thus, targeting the DDR key proteins such as BRCA1 and BRCA2 is a potential effective therapeutic strategy for mono- or combination therapy. In 2005, the synthetic lethality between PARP inhibition and BRCA1 or BRCA2 mutation was reported by two independent groups, suggesting a novel strategy for treating patients with BRCA-mutant tumors (Bryant et al., 2005; Farmer et al., 2005; Xia et al., 2021). Inhibition of PAPR1 causes failure of DNA single-stranded breaks repair, finally leading to DSBs in cells. HR-competent cells can repair DSBs for cell survival, whereas in cells with defects caused by mutations in BRCA1 or BRCA2 or other HR-associated proteins, the DNA damage remains unrepaired, causing cell death (Xia et al., 2021). In 2014, PARP inhibitor Olaparib was primary approved by the FDA to treat certain patients with ovarian cancer. The PARP inhibitors are now indicated for the treatment of patients with germline breast cancer susceptibility gene (BRCA) mutated, human epidermal growth factor receptor 2 (HER2)-negative metastatic BC, who have been previously treated with chemotherapy (Arora et al., 2021; Xia et al., 2021). Although PARPi has shown great efficacy, their widespread use is restricted by various factors, including drug resistance and the limited population. In recent years, there have been a number of studies and clinical trials evaluating the use of cytotoxic chemotherapy such as temozolomide, platinum salts, and topoisomerase inhibitors in combination with PARPi, and the evidence suggests that combination therapy may be of considerable use in many types of cancer (Palleschi et al., 2021). RNA m^6^A modification has been reported to be involved in the regulation of DNA repair. Xiang et al., 2017 reported that METTL3-mediated m^6^A RNA accumulates at UV-damaged sites in DNA, which further recruits DNA polymerase κ (Pol κ) to damaged sites to facilitate NER and cell survival (Xiang et al., 2017). Zhang et al., reported METTL3-m^6^A-YTHDC1 mediated HR in U2OS cells exposed to X-rays or treatment with Zeocin (Zhang et al., 2020). Another recent study found that METTL3-METTL14 is active in vitro on double-stranded DNA containing a cyclopyrimidine dimer, and the m^6^A reader, YTHDC1, is recruited to sites of DNA damage (Yu et al., 2021a). Our data demonstrated that silencing METTL3 inhibited the repair of DSBs induced by ADR. Nevertheless, the molecular mechanism of DNA repair regulation by METTL3/m^6^A remains undefined.

There are two major pathways to repair DSBs including error-free HR and error-prone NHEJ (Sonoda et al., 2006). Using a GFP-based reporter system, we found that METTL3 regulated HR, but not NHEJ in DSB repair, which is consistent with the study of Zhang et al., 2020. However, Gene Ontology (GO) analysis of METTL3-associated gene expression profiles based on RNA-seq in MCF-7 cells did not identify enriched ‘DNA damage’ or ‘DNA repair’ categories (data not shown). This data is supported by Xiang et al., showing that GO analysis of METTL3-dependent, UV-induced methylated transcripts only identified ‘DNA damage’ as weakly enriched (Xiang et al., 2017). Zhang et al., also reported that METTL3 does not change active RNA polymerase II binding to DNA lesions and has no effect on RNA transcription at DSBs. Another study showed that METTL3 catalyzes m^6^A modification of FEN1 mRNA, which is recognized and stabilized by IGF2BP2 in hepatocellular carcinoma cells (Pu et al., 2020). However, our RNA-seq data showed no change in FEN1 expression with METTL3 overexpression in MCF-7 cells, suggesting that METTL3-mediated FEN1 expression may be cell specific.

EGF/EGFR signaling is important to many biological processes, such as cell proliferation, cell division, and tissue development. Human cancer tissues express high levels of growth factors and their receptors, such as EGF/EGFR, which exhibit autocrine or paracrine regulation of cancer growth (Knowlden et al., 2003; Mendelsohn and Baselga, 2000; Singh and Harris, 2005). Aberrant activation of EGF/EGFR signaling contributes to cancer proliferation, epithelial-mesenchymal transition, and metastasis (Mendelsohn and Baselga, 2000). Therefore, targeting EGFR by antibodies or small molecule tyrosine kinase inhibitors has been used successfully to treat various malignancies, including BC (Barzegar et al., 2017; Ueno and Zhang, 2011). Based on RNA-seq, MeRIP-qPCR, and the screening strategy, we determined that EGF is regulated in a METTL3-m^6^A-YTHDC1-dependent manner in MCF-7 and MB-231 cells. Overexpression of METTL3 markedly increased EGF expression and secretion, whereas knockdown of METTL3 resulted in the opposite effect. Moreover, we detected the expression of EGFR in METTL3-OV MCF-7, MB-231, and MCF-10A cells. We found that overexpression of METTL3 enhanced the expression of EGFR in all these cells (Figure 6—figure supplement 1F-H), which is consistent to previous study (Lin et al., 2016). These data indicated METTL3 might have more global effect on EGR-RAD51 axis. We further demonstrated that EGF/EGFR axis plays an important role in METTL3-mediated HR in both ER-positive and triple-negative cells during ADR treatment. Our results are consistent with previous reports showing that EGF/EGFR signaling plays a role in DSB repair (Myllynen et al., 2011). Moreover, dysregulated EGF/EGFR signaling modulates the expression of several genes involved in DNA repair including ERCC1, XRCC1, RAD51, and RAD50 (Kryeziu et al., 2013; Yacoub et al., 2003). Accordingly, we found that EGF/EGFR signaling regulated RAD51 expression in both MCF-7 and MB-231 cells and the regulation of RAD51 expression by METTL3 was EGF-dependent. Furthermore, knockdown of RAD51 or inhibition of EGFR by gefitinib/erlotinib impaired the effect of METTL3 on the up-regulation of DNA repair activity. Although other studies and our RNA-seq data suggest that there may be other factors involved in the regulation of METTL3-mediated DNA repair and response to chemotherapy (Cai et al., 2018; Pan et al., 2021; Wang et al., 2020a). Our experiments demonstrate a role for EGF in the regulation of HR activity and ADR sensitivity in BC.

The m^6^A modification of EGF mRNA has been detected in human kidney and embryonic stem cells by different groups using m^6^A-REF-seq (GSE125240) and MAZTER-seq (GSE122961), respectively (Garcia-Campos et al., 2019; Zhang et al., 2019). Our MeRIP-qPCR assay identified that m^6^A modification of EGF mRNA was augmented in METTL3-expressing MCF-10A, MCF-7, and MB-231 cells compared with that in control cells. The m^6^A-modified EGF mRNA was recognized and bound by YTHDC1, which further promoted EGF synthesis and secretion. We demonstrated that the regulation of EGF expression by METTL3 was m^6^A-YTHDC1-dependent and knockdown of YTHDC1 impeded the up-regulation of EGF in METTL3-overexpressing cells, and restored the sensitivity of MCF-7 cells to ADR. Moreover, knockdown of YTHDC1 impeded METTL3-enhanced RAD51 expression and inhibited recruitment of RAD51 to damaged sites. Our data combined with the studies of Zhang et al., and Yu et al., demonstrate that YTHDC1 plays an important role in DNA repair (Yu et al., 2021a; Zhang et al., 2020). Our results further suggest the involvement of YTHDC1 in the regulation of EGF mRNA stability. Although YTHDC1 is primary known as a nuclear m^6^A reader, which regulates mRNA splicing through the recruitment and modulation of pre-mRNA splicing factors such as SRSF3 (Xiao et al., 2016). Our data suggest that YTHDC1 may contribute to the stability of cytosolic mRNA, including EGF, through m^6^A. Our hypothesis is supported by previous studies suggesting that YTHDC1 can shuttle between the nucleus and the cytoplasm (Rafalska et al., 2004), to process mature mRNAs, including MAT2A (Shima et al., 2017). Moreover, Roundtree et al., showed that YTHDC1 mediates the export of methylated mRNA from the nucleus to the cytoplasm, resulting in nuclear clearance of mRNAs and accompanying their resulting cytoplasmic abundance (Roundtree et al., 2017). These results suggest multiple processes through which YTHDC1 regulates the processing of mature mRNA, whereas the detailed molecular mechanism warrants further study.

Collectively, we showed an effect of METTL3 on HR via the m^6^A-YTHDC1-dependent regulation of the EGF/RAD51 axis, and demonstrated a role for METTL3 in the response of BC chemotherapy. Our results suggest that the development of METTL3 inhibitors or targeting its pathway may lead to promising treatments for cancer patients.

## Materials and methods

**Key resources table:** 

Reagent type (species) or resource	Designation	Source or reference	Identifiers	Additional information
Genetic reagent (*Mus. musculus*)	BALB/c-Nude (BALB/cNj-Foxn1^nu^/Gpt)	GemPharmatech Co., Ltd., Nanjing, China	Strain NO.D000521	
Cell line (*Homo sapiens*)	MCF-7	National Collection of Authenticated Cell Cultures, Chinese Academy of Science	CSTR:19375.09. 3101HUMSCSP531	
Cell line (*Homo sapiens*)	MDA-MB-231	National Collection of Authenticated Cell Cultures, Chinese Academy of Science	CSTR:19375.09. 3101HUMSCSP5043	
Antibody	anti-METTL3 (Mouse monoclonal)	ABclonal	Cat# A19079 RRID: Addgene_101892	WB (1:1000)
Antibody	anti-γ-H2AX (Mouse monoclonal)	Cell Signaling Technology	Cat# 80,312 S	WB (1:1000) IF(1:300)
Antibody	anti-BAX (Rabbit polyclonal)	ABclonal	Cat# A11550 RRID: AB_516294	WB (1:1000)
Antibody	anti-caspase3 (Rabbit polyclonal)	Proteintech	Cat# 19677-I-AP RRID: AB_590739	WB (1:1000)
Antibody	anti-EGF (Rabbit polyclonal)	Proteintech	Cat# 27141–1-AP RRID: AB_1066833	WB (1:1000)
Antibody	anti-RAD51 (Rabbit polyclonal)	Proteintech	Cat# 14961–1-AP RRID: AB_10706869	WB (1:1000) IF (1:300)
Antibody	anti-FLAG (Mouse monoclonal)	bioworld	Cat# AP0007MH **RRID：AB_1537400**	WB (1:1000)
Antibody	anti-EGFR (Rabbit polyclonal)	ABclonal	Cat# A11577 RRID: AB_442085	WB (1:1000)
Antibody	anti-TBB5 (Mouse monoclonal)	Abgent	Cat# AM1031A RRID: AB_1554765	WB (1:1000)
Antibody	anti-Cyclin A (Mouse monoclonal)	proteintech	Cat# 66391–1-Ig	IF(1:300)
Sequence-based reagent	METTL3_F	This paper	qPCR primers	AAGCTGCACTTCAGACGAAT
Sequence-based reagent	METTL3_R	This paper	qPCR primers	GGAATCACCTCCGACACTC
Sequence-based reagent	EGF_F	This paper	qPCR primers	TGGATGTGCTTGATAAGCGG
Sequence-based reagent	EGF_R	This paper	qPCR primers	ACCATGTCCTTTCCAGTGTGT
Sequence-based reagent	RAD51_F	This paper	qPCR primers	CAACCCATTTCACGGTTAGAGC
Sequence-based reagent	RAD51_R	This paper	qPCR primers	TTCTTTGGCGCATAGGCAACA
Commercial assay or kit	Human EGF ELISA Kit	SenBeiJia Biological Technology Co.	Cat# SBJ-H0212	
Chemical compound, drug	Doxorubicin (Adriamycin) HCl	Selleck	Cat# S1208 CAS No. 25316-40-9	
Chemical compound, drug	Paclitaxel	Selleck	Cat# S1150 CAS No. 33069-62-4	
Chemical compound, drug	Cisplatin	Selleck	Cat# S1166 CAS No. 15663-27-1	
Chemical compound, drug	5-Fluorouracil, 5-FU	Selleck	Cat# S1209 CAS No. 51-21-8	
Chemical compound, drug	Recombinant Human EGF	Beyotime	Cat# P5552	
Chemical compound, drug	Erlotinib	Beyotime	Cat# SC0168	
Chemical compound, drug	Gefitinib	Beyotime	Cat# SC0186	
Software, algorithm	GraphPad Prism software	GraphPad Prism (https://graphpad.com)	RRID: SCR_015807	Version 8.0.0

### Plasmid construction

The oligonucleotide 5′-CAGGAGATCCTAGAGCTATTA-3′ was used for construction of METTL3-KD lentivirus vectors as previously described (Xiang et al., 2017). The lentivirus vectors were constructed and purified by the Corues Biotechnology Company (Nanjing, China). For knockdown of RAD51, YTHDC1 and other ‘readers,’ and the silencing plasmids containing shRNA sequences were constructed based on psilencer3.0-H1. The shRNA sequences are listed in Table 3. All plasmids were verified by sequencing.

**Table 3.** Sequences of the shRNA used in this study.

Gene name	shRNA sequences
sh-METTL3	5’-CAGGAGATCCTAGAGCTATTA-3’
sh-RAD51	5’-GACTGCCAGGATAAAGCTT-3’
sh-YTHDC1	5’-CCAGAGAGTGAACAAGATAAA-3’
sh-YTHDF1	5’-GGGGGTTGAGTGTTGCATCTT-3’
sh-YTHDF2	5’-AAGGCTAAGCAGGTGTTGAAA-3’
sh-YTHDF3	5’-TAAGTCAAAGAAGACGTATTACTC-3’
sh-YTHDC2	5’-GCCCACAGATTGGCTTATTTA-3’
sh-IGF2BP1	5’-TGCTATTCTTCCTAATCTATATC-3’
sh-IGF2BP2	5’-GTGAAGCTGGAAGCGCATATCTC-3’
sh-IGF2BP3	5’-CGGTGAATGAACTTCAGAATTCTC-3’

### Cell culture and the development of stable cell lines

MCF-7 and MB-231 were purchased from the National Collection of Authenticated Cell Cultures, Chinese Academy of Science. All cells were authenticated by STR profiling and tested for mycoplasma contamination. Cells were cultured in the recommended medium supplemented with 10% fetal bovine serum (FBS, Invigentech), 1% penicillin, and 1% streptomycin, and incubated in an incubator with 5% CO_2_ at 37℃. For METTL3-overexpressing (OV) or –knockdown (KD) MCF-7 and MB-231 stable cells, the cells were infected with specific lentivirus vectors for 48 hr and then selected with puromycin for two weeks. All cell lines were confirmed to be negative for mycoplasma contamination.

### ^m6^A dot blotting

Total RNA was isolated using the Trizol method and mRNAs were isolated with the GenElute mRNA Miniprep Kit (Sigma). The concentration and purity of the mRNA were measured using a NanoDrop 2000. The mRNAs were denatured by heating to 95°C for 5 min, followed by chilling on ice. Next, the mRNAs (50∼100 ng) were spotted directly onto a positively-charged nylon membrane (GE Healthcare, USA) and air-dried at room temperature for 5 min. The membrane was then ultraviolet (UV) crosslinked using a Ultraviolet Crosslinker, washed with PBST for 5 min, blocked with 5% nonfat milk in TBST, and then incubated with anti-m^6^A antibody (A17924, ABclonal) overnight at 4°C. HRP-conjugated anti-rabbit IgG secondary antibody was added to the membrane for 1.5 hr at room temperature with gentle shaking, followed by development with enhanced chemiluminescence. Last, 0.02% methylene blue staining was used to verify that equal amounts of mRNA were spotted onto the membrane.

### Drug sensitivity assay

Cells were seeded into 96-well plates at 3000 cells per well for at least three parallel experiments. 24 hr later, cells were exposed to ADR at increasing concentrations (0, 0.01, 0.05, 0.1, 0.5, and 1 μΜ) for 48 hr; 5-FU at increasing concentrations (0, 0.1, 0.5, 1, 5 μΜ) for 48 hr; carboplatin at increasing concentrations (0, 10, 50, 100, 500 μΜ) for 48 hr; paclitaxel at increasing concentrations (0, 0.01, 0.1, 1 nΜ) for 48 hr; or DDP at increasing concentrations (0, 2, 4, 6, 8 μΜ) for 48 hr. Chemotherapeutic drug-treated cells were incubated with 10 μL 3-(4,5)-dimethylthiazol (-z-y1)–3,5-diphenyltetrazolium bromide (MTT) solution (5 mg/mL, Sigma-Aldrich, St Louis, MO, USA) for 4 hr. The media was replaced with 100 μL dimethyl sulfoxide (DMSO, Sigma-Aldrich) to dissolve the formazan crystals within 10 min. The absorbance of the formazan was read at 450 nm. The relative values were calculated based on the mean of control cells in the absence of ADR, which was showed as 100%. At least three replicates were performed for each drug treatment.

### RNA-seq and analysis

RNA-Seq was performed by oeBiotech Inc (Shanghai, China). For RNA sequencing, purified RNA from METTL3 overexpressing or control cells was used for library construction with the Illumina TruSeq RNA Sample Prep Kit (FC-122–1001) and then sequenced with an Illumina HiSeq 2000. Raw reads were aligned to the human genome, GRCh37/hg19, by Bowtie2. Differentially expressed genes (DEGs) between METTL3-OV and the control samples were identified using the limma-voom method. A heatmap clustered by k-means was used to show DEGs or transcripts. The raw sequencing data were deposited in the Gene Expression Omnibus database (accession to cite for these SRA data: PRJNA743152).

### RNA immunoprecipitation (RIP)

RNA immunoprecipitation (RIP) assays were conducted using the EZ-Magna RIP RNA-Binding Protein Immunoprecipitation Kit Merck Chemicals (Shanghai Co., Ltd). The anti-YTHDC1 antibody for RIP was purchased from Cell Signaling Technology, Inc (# 81,504 S).

### m^6^A-RNA immunoprecipitation (MeRIP) and MeRIP-qPCR

m^6^A enrichment followed by qRT-PCR was used to quantify the changes in m^6^A methylation of the target gene using the Magna MeRIP m^6^A Kit (Millipore, MA) following the manufacturer’s instructions. Briefly, 5 μg of fragmented mRNA extracted from MCF-7 stable cells was incubated with 5 μg of m^6^A antibody (A17924, ABclonal). Methylated mRNA was eluted by free m^6^A from the beads and purified with the GenElute mRNA Miniprep Kit (MRN70, Sigma). One tenth of the fragmented RNA was saved as an input control for standardization. The relevant enrichment of m^6^A from METTL3 in each sample was analyzed by RT-qPCR.

### Immunofluorescence

For immunofluorescence assays, cells were washed with PBS for three times then fixed with 4% formaldehyde for 10 min at room temperature. After permeabilization with 0.1% Triton X-100 for 10 min, cells were blocked with 3% BSA for 1 hr. Then, cells were incubated with indicated primary antibodies overnight at 4 °C. Following washed with PBST for three times, cells were incubated with fluorescent secondary antibodies for 2 hr at room temperature. Subsequently, cells were stained with DAPI and visualized under a fluorescence microscope (Nikon 80I 10–1500×).

### Tumor analysis

All animal experiments were performed according to the procedures approved by the Laboratory Animal Care Committee at Nanjing Normal University (Permit number IACUC—20210251) and followed National Institutes of Health guide for the care and use of Laboratory animals. Six-to-seven weeks old female nude mice were purchased from GemPharmatech Co., Ltd. (Nanjing, China), and were maintained under specific pathogen-free conditions for subcutaneous inoculation. Cells were trypsinized and resuspended in DMEM at a consistence of 1 × 10^7^ cells/ml. A total of 1 × 10^6^ cells were injected into flank of mice. 27 days after injection, tumors were removed for paraffin-embedded sections.

### Immunohistochemistry

Immunohistochemical staining was performed as previously described (Lu et al., 2020). Briefly, tumor tissues were fixed in 4% polysorbate. Paraffin-embedded sections from tissue specimens were deparaffinized and heated at 100 °C in 10 mM citrate buffer (pH 6.0) for 15 min for antigen retrieval. Slides were incubated with primary antibody at 4 °C overnight, followed by incubation with secondary antibody at room temperature and visualized using a DAB Kit (Bioworld). Then, it was redacted with hematoxylin. The expression levels of target proteins in tissue were examed according to the semiquantitative immunoreactivity score (IRS).

### Apoptosis assay

METTL3-KD or control MCF-7 or MB-231 cells were treated with doxorubicin for 24 hr and replaced with fresh media. After other 24 hr recovery, about 1 × 10^5^ cells per well were collected and stained with both Annexin V and propidium iodide (PI). Apoptosis was analyzed by flow cytometry using the BD FACSverse.

### ELISA

The cell culture media were centrifuged at the speed of 5000 r.p.m for 5 min and supernatant were collected for EGF measurement using commercial kits (SenBeiJia Biological Technology Co., Nanjing, China) according to manufacturer’s instructions.

### Statistical analysis

Statistical analysis was performed with GraphPad Prism 8.0. Statistical significance was determined using a two-tailed Student’s t-test or analysis of variance in the case of comparisons among multiple groups. p<0.05 was considered statistically significant.

## Data Availability

The raw sequencing data were deposited in the Gene Expression Omnibus database (accession to cite for these SRA data: PRJNA743152). The following dataset was generated: EnjieL ZhigangH 2021METTL3 Overexpressing MCF-7NCBI BioProjectPRJNA743152

## Additional files

Transparent reporting form

## References

[bib1] Ali R, Rakha EA, Madhusudan S, Bryant HE (2017). DNA damage repair in breast cancer and its therapeutic implications. Pathology.

[bib2] Andreetta C, Minisini AM, Miscoria M, Puglisi F (2010). First-line chemotherapy with or without biologic agents for metastatic breast cancer. Critical Reviews in Oncology/Hematology.

[bib3] Arora S, Balasubramaniam S, Zhang H, Berman T, Narayan P, Suzman D, Bloomquist E, Tang S, Gong Y, Sridhara R, Turcu FR, Chatterjee D, Saritas-Yildirim B, Ghosh S, Philip R, Pathak A, Gao JJ, Amiri-Kordestani L, Pazdur R, Beaver JA (2021). FDA Approval Summary: Olaparib Monotherapy or in Combination with Bevacizumab for the Maintenance Treatment of Patients with Advanced Ovarian Cancer. The Oncologist.

[bib4] Asan A, Skoko JJ, Woodcock CSC, Wingert BM, Woodcock SR, Normolle D, Huang Y, Stark JM, Camacho CJ, Freeman BA, Neumann CA (2019). Electrophilic fatty acids impair RAD51 function and potentiate the effects of DNA-damaging agents on growth of triple-negative breast cells. The Journal of Biological Chemistry.

[bib5] Barzegar M, Ma S, Zhang C, Chen X, Gu Y, Shang C, Jiang X, Yang J, Nathan CA, Yang S, Huang S (2017). SKLB188 inhibits the growth of head and neck squamous cell carcinoma by suppressing EGFR signalling. British Journal of Cancer.

[bib6] Binaschi M, Capranico G, De Isabella P, Mariani M, Supino R, Tinelli S, Zunino F (1990). Comparison of DNA cleavage induced by etoposide and doxorubicin in two human small-cell lung cancer lines with different sensitivities to topoisomerase II inhibitors. International Journal of Cancer.

[bib7] Bryant HE, Schultz N, Thomas HD, Parker KM, Flower D, Lopez E, Kyle S, Meuth M, Curtin NJ, Helleday T (2005). Specific killing of BRCA2-deficient tumours with inhibitors of poly(ADP-ribose) polymerase. Nature.

[bib8] Cai X, Wang X, Cao C, Gao Y, Zhang S, Yang Z, Liu Y, Zhang X, Zhang W, Ye L (2018). HBXIP-elevated methyltransferase METTL3 promotes the progression of breast cancer via inhibiting tumor suppressor let-7g. Cancer Letters.

[bib9] Chan S, Friedrichs K, Noel D, Pintér T, Van Belle S, Vorobiof D, Duarte R, Gil Gil M, Bodrogi I, Murray E, Yelle L, von Minckwitz G, Korec S, Simmonds P, Buzzi F, González Mancha R, Richardson G, Walpole E, Ronzoni M, Murawsky M, Alakl M, Riva A, Crown J, 303 Study Group (1999). Prospective randomized trial of docetaxel versus doxorubicin in patients with metastatic breast cancer. Journal of Clinical Oncology.

[bib10] Deng X, Su R, Weng H, Huang H, Li Z, Chen J (2018). RNA N^6^-methyladenosine modification in cancers: current status and perspectives. Cell Research.

[bib11] Farmer H, McCabe N, Lord CJ, Tutt ANJ, Johnson DA, Richardson TB, Santarosa M, Dillon KJ, Hickson I, Knights C, Martin NMB, Jackson SP, Smith GCM, Ashworth A (2005). Targeting the DNA repair defect in BRCA mutant cells as a therapeutic strategy. Nature.

[bib12] Fisher B, Bryant J, Wolmark N, Mamounas E, Brown A, Fisher ER, Wickerham DL, Begovic M, DeCillis A, Robidoux A, Margolese RG, Cruz AB, Hoehn JL, Lees AW, Dimitrov NV, Bear HD (1998). Effect of preoperative chemotherapy on the outcome of women with operable breast cancer. Journal of Clinical Oncology.

[bib13] Fisusi FA, Akala EO (2019). Drug Combinations in Breast Cancer Therapy. Pharmaceutical Nanotechnology.

[bib14] Gagou ME, Zuazua-Villar P, Meuth M (2010). Enhanced H2AX phosphorylation, DNA replication fork arrest, and cell death in the absence of Chk1. Molecular Biology of the Cell.

[bib15] Garcia-Campos MA, Edelheit S, Toth U, Safra M, Shachar R, Viukov S, Winkler R, Nir R, Lasman L, Brandis A, Hanna JH, Rossmanith W, Schwartz S (2019). Deciphering the “m^6^A Code” via Antibody-Independent Quantitative Profiling. Cell.

[bib16] Grundy MK, Buckanovich RJ, Bernstein KA (2020). Regulation and pharmacological targeting of RAD51 in cancer. NAR Cancer.

[bib17] He L, Zhang Y, Sun H, Jiang F, Yang H, Wu H, Zhou T, Hu S, Kathera CS, Wang X, Chen H, Li H, Shen B, Zhu Y, Guo Z (2016). Targeting DNA Flap Endonuclease 1 to Impede Breast Cancer Progression. EBioMedicine.

[bib18] Hengel SR, Malacaria E, Folly da Silva Constantino L, Bain FE, Diaz A, Koch BG, Yu L, Wu M, Pichierri P, Spies MA, Spies M (2016). Small-molecule inhibitors identify the RAD52-ssDNA interaction as critical for recovery from replication stress and for survival of BRCA2 deficient cells. eLife.

[bib19] Hengel S.R., Spies MA, Spies M (2017). Small-Molecule Inhibitors Targeting DNA Repair and DNA Repair Deficiency in Research and Cancer Therapy. Cell Chemical Biology.

[bib20] Hennigs A, Riedel F, Marmé F, Sinn P, Lindel K, Gondos A, Smetanay K, Golatta M, Sohn C, Schuetz F, Heil J, Schneeweiss A (2016). Changes in chemotherapy usage and outcome of early breast cancer patients in the last decade. Breast Cancer Research and Treatment.

[bib21] Huang F, Goyal N, Sullivan K, Hanamshet K, Patel M, Mazina OM, Wang CX, An WF, Spoonamore J, Metkar S, Emmitte KA, Cocklin S, Skorski T, Mazin AV (2016). Targeting BRCA1- and BRCA2-deficient cells with RAD52 small molecule inhibitors. Nucleic Acids Research.

[bib22] Knowlden JM, Hutcheson IR, Jones HE, Madden T, Gee JMW, Harper ME, Barrow D, Wakeling AE, Nicholson RI (2003). Elevated levels of epidermal growth factor receptor/c-erbB2 heterodimers mediate an autocrine growth regulatory pathway in tamoxifen-resistant MCF-7 cells. Endocrinology.

[bib23] Kriegs M, Kasten-Pisula U, Rieckmann T, Holst K, Saker J, Dahm-Daphi J, Dikomey E (2010). The epidermal growth factor receptor modulates DNA double-strand break repair by regulating non-homologous end-joining. DNA Repair.

[bib24] Kryeziu K, Jungwirth U, Hoda MA, Ferk F, Knasmüller S, Karnthaler-Benbakka C, Kowol CR, Berger W, Heffeter P (2013). Synergistic anticancer activity of arsenic trioxide with erlotinib is based on inhibition of EGFR-mediated DNA double-strand break repair. Molecular Cancer Therapeutics.

[bib25] Li L, Kumar AK, Hu Z, Guo Z (2021). Small Molecule Inhibitors Targeting Key Proteins in the DNA Damage Response for Cancer Therapy. Current Medicinal Chemistry.

[bib26] Lin S, Choe J, Du P, Triboulet R, Gregory RI (2016). The m(6)A Methyltransferase METTL3 Promotes Translation in Human Cancer Cells. Molecular Cell.

[bib27] Liu J, Yue Y, Han D, Wang X, Fu Y, Zhang L, Jia G, Yu M, Lu Z, Deng X, Dai Q, Chen W, He C (2014). A METTL3-METTL14 complex mediates mammalian nuclear RNA N6-adenosine methylation. Nature Chemical Biology.

[bib28] Lok BH, Carley AC, Tchang B, Powell SN (2013). RAD52 inactivation is synthetically lethal with deficiencies in BRCA1 and PALB2 in addition to BRCA2 through RAD51-mediated homologous recombination. Oncogene.

[bib29] Long K, Gu L, Li L, Zhang Z, Li E, Zhang Y, He L, Pan F, Guo Z, Hu Z (2021). Small-molecule inhibition of APE1 induces apoptosis, pyroptosis, and necroptosis in non-small cell lung cancer. Cell Death & Disease.

[bib30] Lu X, Liu R, Wang M, Kumar AK, Pan F, He L, Hu Z, Guo Z (2020). MicroRNA-140 impedes DNA repair by targeting FEN1 and enhances chemotherapeutic response in breast cancer. Oncogene.

[bib31] Malik SS, Zia A, Rashid S, Mubarik S, Masood N, Hussain M, Yasmin A, Bano R (2020). XPC as breast cancer susceptibility gene: evidence from genetic profiling, statistical inferences and protein structural analysis. Breast Cancer (Tokyo, Japan).

[bib32] Martin RW, Orelli BJ, Yamazoe M, Minn AJ, Takeda S, Bishop DK (2007). RAD51 up-regulation bypasses BRCA1 function and is a common feature of BRCA1-deficient breast tumors. Cancer Research.

[bib33] Mendelsohn J, Baselga J (2000). The EGF receptor family as targets for cancer therapy. Oncogene.

[bib34] Mendez-Dorantes C, Tsai LJ, Jahanshir E, Lopezcolorado FW, Stark JM (2020). BLM has Contrary Effects on Repeat-Mediated Deletions, based on the Distance of DNA DSBs to a Repeat and Repeat Divergence. Cell Reports.

[bib35] Miki Y, Swensen J, Shattuck-Eidens D, Futreal PA, Harshman K, Tavtigian S, Liu Q, Cochran C, Bennett LM, Ding W (1994). A strong candidate for the breast and ovarian cancer susceptibility gene BRCA1. Science (New York, N.Y.).

[bib36] Myllynen L, Rieckmann T, Dahm-Daphi J, Kasten-Pisula U, Petersen C, Dikomey E, Kriegs M (2011). In tumor cells regulation of DNA double strand break repair through EGF receptor involves both NHEJ and HR and is independent of p53 and K-Ras status. Radiotherapy and Oncology.

[bib37] Ouyang J, Yadav T, Zhang JM, Yang HB, Rheinbay E, Guo HS, Haber DA, Lan L, Zou L (2021). RNA transcripts stimulate homologous recombination by forming DR-loops. Nature.

[bib38] Palleschi M, Tedaldi G, Sirico M, Virga A, Ulivi P, De Giorgi U (2021). Moving beyond PARP Inhibition: Current State and Future Perspectives in Breast Cancer. International Journal of Molecular Sciences.

[bib39] Pan X, Hong X, Li S, Meng P, Xiao F (2021). METTL3 promotes adriamycin resistance in MCF-7 breast cancer cells by accelerating pri-microRNA-221-3p maturation in a m6A-dependent manner. Experimental & Molecular Medicine.

[bib40] Paridaens R, Biganzoli L, Bruning P, Klijn JG, Gamucci T, Houston S, Coleman R, Schachter J, Van Vreckem A, Sylvester R, Awada A, Wildiers J, Piccart M (2000). Paclitaxel versus doxorubicin as first-line single-agent chemotherapy for metastatic breast cancer: A European Organization for Research and Treatment of Cancer Randomized Study with cross-over. Journal of Clinical Oncology.

[bib41] Pu J, Wang J, Qin Z, Wang A, Zhang Y, Wu X, Wu Y, Li W, Xu Z, Lu Y, Tang Q, Wei H (2020). IGF2BP2 Promotes Liver Cancer Growth Through an m6A-FEN1-Dependent Mechanism. Frontiers in Oncology.

[bib42] Rafalska I, Zhang Z, Benderska N, Wolff H, Hartmann AM, Brack-Werner R, Stamm S (2004). The intranuclear localization and function of YT521-B is regulated by tyrosine phosphorylation. Human Molecular Genetics.

[bib43] Roundtree IA, Luo G-Z, Zhang Z, Wang X, Zhou T, Cui Y, Sha J, Huang X, Guerrero L, Xie P, He E, Shen B, He C (2017). YTHDC1 mediates nuclear export of N^6^-methyladenosine methylated mRNAs. eLife.

[bib44] Shima H, Matsumoto M, Ishigami Y, Ebina M, Muto A, Sato Y, Kumagai S, Ochiai K, Suzuki T, Igarashi K (2017). S-Adenosylmethionine Synthesis Is Regulated by Selective N^6^-Adenosine Methylation and mRNA Degradation Involving METTL16 and YTHDC1. Cell Reports.

[bib45] Singh AB, Harris RC (2005). Autocrine, paracrine and juxtacrine signaling by EGFR ligands. Cellular Signalling.

[bib46] Sonoda E, Hochegger H, Saberi A, Taniguchi Y, Takeda S (2006). Differential usage of non-homologous end-joining and homologous recombination in double strand break repair. DNA Repair.

[bib47] Swift LP, Rephaeli A, Nudelman A, Phillips DR, Cutts SM (2006). Doxorubicin-DNA adducts induce a non-topoisomerase II-mediated form of cell death. Cancer Research.

[bib48] Tang Y, Chen K, Song B, Ma J, Wu X, Xu Q, Wei Z, Su J, Liu G, Rong R, Lu Z, de Magalhães JP, Rigden DJ, Meng J (2021). m6A-Atlas: a comprehensive knowledgebase for unraveling the N6-methyladenosine (m6A) epitranscriptome. Nucleic Acids Research.

[bib49] Thacker J (2005). The RAD51 gene family, genetic instability and cancer. Cancer Letters.

[bib50] Tsai LJ, Lopezcolorado FW, Bhargava R, Mendez-Dorantes C, Jahanshir E, Stark JM (2020). RNF8 has both KU-dependent and independent roles in chromosomal break repair. Nucleic Acids Research.

[bib51] Ueno NT, Zhang D (2011). Targeting EGFR in Triple Negative Breast Cancer. Journal of Cancer.

[bib52] Wang B, Matsuoka S, Carpenter PB, Elledge SJ (2002). 53BP1, a mediator of the DNA damage checkpoint. Science (New York, N.Y.).

[bib53] Wang Xiao, Lu Z, Gomez A, Hon GC, Yue Y, Han D, Fu Y, Parisien M, Dai Q, Jia G, Ren B, Pan T, He C (2014). N6-methyladenosine-dependent regulation of messenger RNA stability. Nature.

[bib54] Wang X., Zhao BS, Roundtree IA, Lu Z, Han D, Ma H, Weng X, Chen K, Shi H, He C (2015). N(6)-methyladenosine Modulates Messenger RNA Translation Efficiency. Cell.

[bib55] Wang M, Li E, Lin L, Kumar AK, Pan F, He L, Zhang J, Hu Z, Guo Z (2019). Enhanced Activity of Variant DNA Polymerase β (D160G) Contributes to Cisplatin Therapy by Impeding the Efficiency of NER. Molecular Cancer Research.

[bib56] Wang M, Long K, Li E, Li L, Li B, Ci S, He L, Pan F, Hu Z, Guo Z (2020a). DNA polymerase beta modulates cancer progression via enhancing CDH13 expression by promoter demethylation. Oncogene.

[bib57] Wang H, Xu B, Shi J (2020b). N6-methyladenosine METTL3 promotes the breast cancer progression via targeting Bcl-2. Gene.

[bib58] Ward IM, Chen JJ (2001). Histone H2AX is phosphorylated in an ATR-dependent manner in response to replicational stress. The Journal of Biological Chemistry.

[bib59] Wilson KJ, Gilmore JL, Foley J, Lemmon MA, Riese DJ (2009). Functional selectivity of EGF family peptide growth factors: implications for cancer. Pharmacology & Therapeutics.

[bib60] Xia M, Guo Z, Hu Z (2021). The Role of PARP Inhibitors in the Treatment of Prostate Cancer: Recent Advances in Clinical Trials. Biomolecules.

[bib61] Xiang Y, Laurent B, Hsu C-H, Nachtergaele S, Lu Z, Sheng W, Xu C, Chen H, Ouyang J, Wang S, Ling D, Hsu P-H, Zou L, Jambhekar A, He C, Shi Y (2017). RNA m^6^A methylation regulates the ultraviolet-induced DNA damage response. Nature.

[bib62] Xiao W, Adhikari S, Dahal U, Chen Y-S, Hao Y-J, Sun B-F, Sun H-Y, Li A, Ping X-L, Lai W-Y, Wang X, Ma H-L, Huang C-M, Yang Y, Huang N, Jiang G-B, Wang H-L, Zhou Q, Wang X-J, Zhao Y-L, Yang Y-G (2016). Nuclear m(6)A Reader YTHDC1 Regulates mRNA Splicing. Molecular Cell.

[bib63] Xu C, Wang X, Liu K, Roundtree IA, Tempel W, Li Y, Lu Z, He C, Min J (2014). Structural basis for selective binding of m6A RNA by the YTHDC1 YTH domain. Nature Chemical Biology.

[bib64] Yacoub A, McKinstry R, Hinman D, Chung T, Dent P, Hagan MP (2003). Epidermal growth factor and ionizing radiation up-regulate the DNA repair genes XRCC1 and ERCC1 in DU145 and LNCaP prostate carcinoma through MAPK signaling. Radiation Research.

[bib65] Yang XH, Sladek TL, Liu X, Butler BR, Froelich CJ, Thor AD (2001). Reconstitution of caspase 3 sensitizes MCF-7 breast cancer cells to doxorubicin- and etoposide-induced apoptosis. Cancer Research.

[bib66] Yang F, Teves SS, Kemp CJ, Henikoff S (2014). Doxorubicin, DNA torsion, and chromatin dynamics. Biochimica et Biophysica Acta.

[bib67] Yu D, Horton JR, Yang J, Hajian T, Vedadi M, Sagum CA, Bedford MT, Blumenthal RM, Zhang X, Cheng X (2021a). Human MettL3-MettL14 RNA adenine methyltransferase complex is active on double-stranded DNA containing lesions. Nucleic Acids Research.

[bib68] Yu F, Wei J, Cui X, Yu C, Ni W, Bungert J, Wu L, He C, Qian Z (2021b). Post-translational modification of RNA m6A demethylase ALKBH5 regulates ROS-induced DNA damage response. Nucleic Acids Research.

[bib69] Zhang Z, Chen LQ, Zhao YL, Yang CG, Roundtree IA, Zhang Z, Ren J, Xie W, He C, Luo GZ (2019). Single-base mapping of m(6)A by an antibody-independent method. Science Advances.

[bib70] Zhang C, Chen L, Peng D, Jiang A, He Y, Zeng Y, Xie C, Zhou H, Luo X, Liu H, Chen L, Ren J, Wang W, Zhao Y (2020). METTL3 and N6-Methyladenosine Promote Homologous Recombination-Mediated Repair of DSBs by Modulating DNA-RNA Hybrid Accumulation. Molecular Cell.

[bib71] Zhou Y, Zeng P, Li YH, Zhang Z, Cui Q (2016). SRAMP: prediction of mammalian N6-methyladenosine (m6A) sites based on sequence-derived features. Nucleic Acids Research.

