# Supplementary figures and images for: METTL3 promotes homologous recombination repair and modulates chemotherapeutic response in breast cancer by regulating the EGF/RAD51 axis (part 1 of 2)
Source: eLife. 2022 May 3;11:e75231. doi: 10.7554/eLife.75231 (PMC9094751; doi:10.7554/eLife.75231)

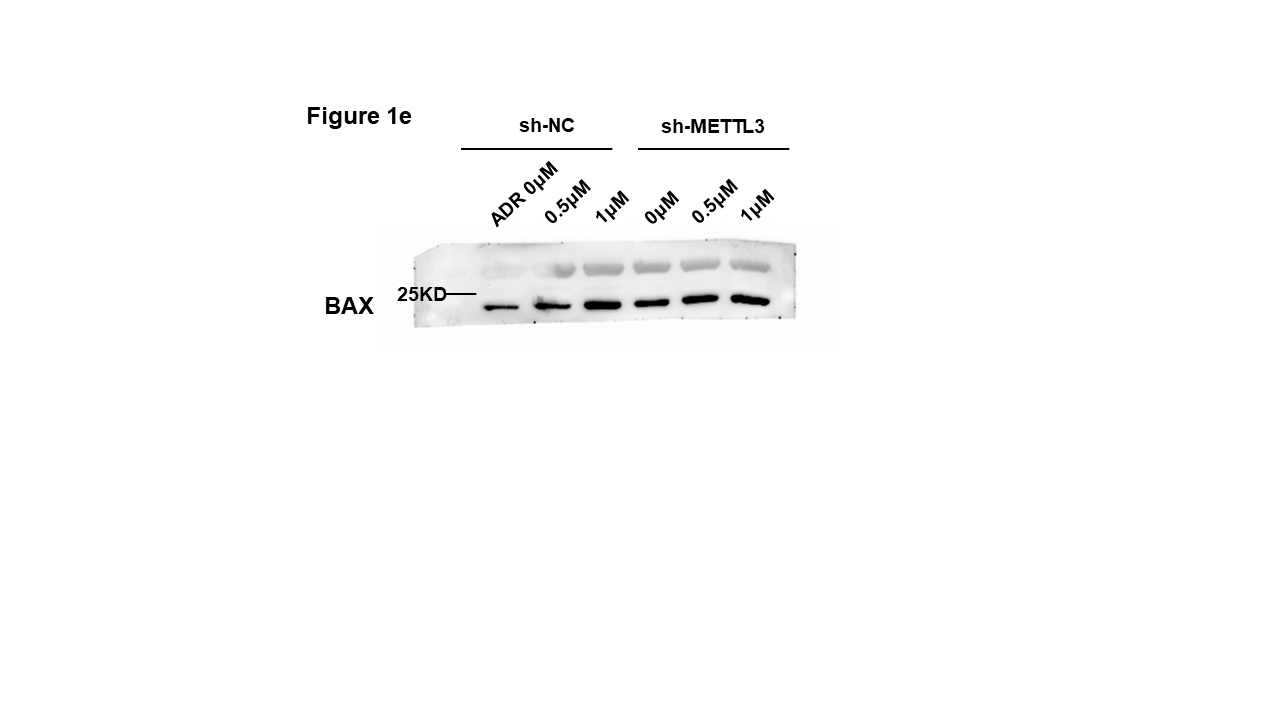

Supplement: Figure 1—source data 1. [file elife-75231-fig1-data1.zip › Figure 1e/Figure 1e BAX.TIF]

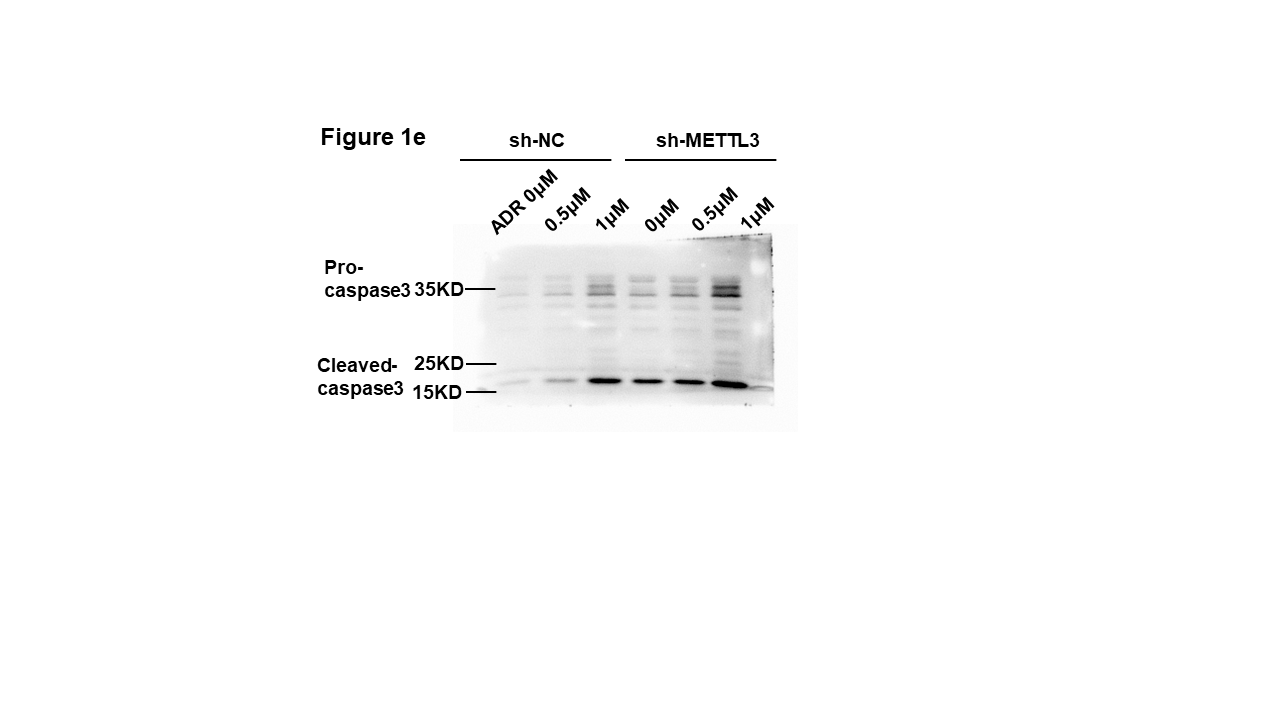

Supplement: Figure 1—source data 1. [file elife-75231-fig1-data1.zip › Figure 1e/Figure 1e caspase3.TIF]

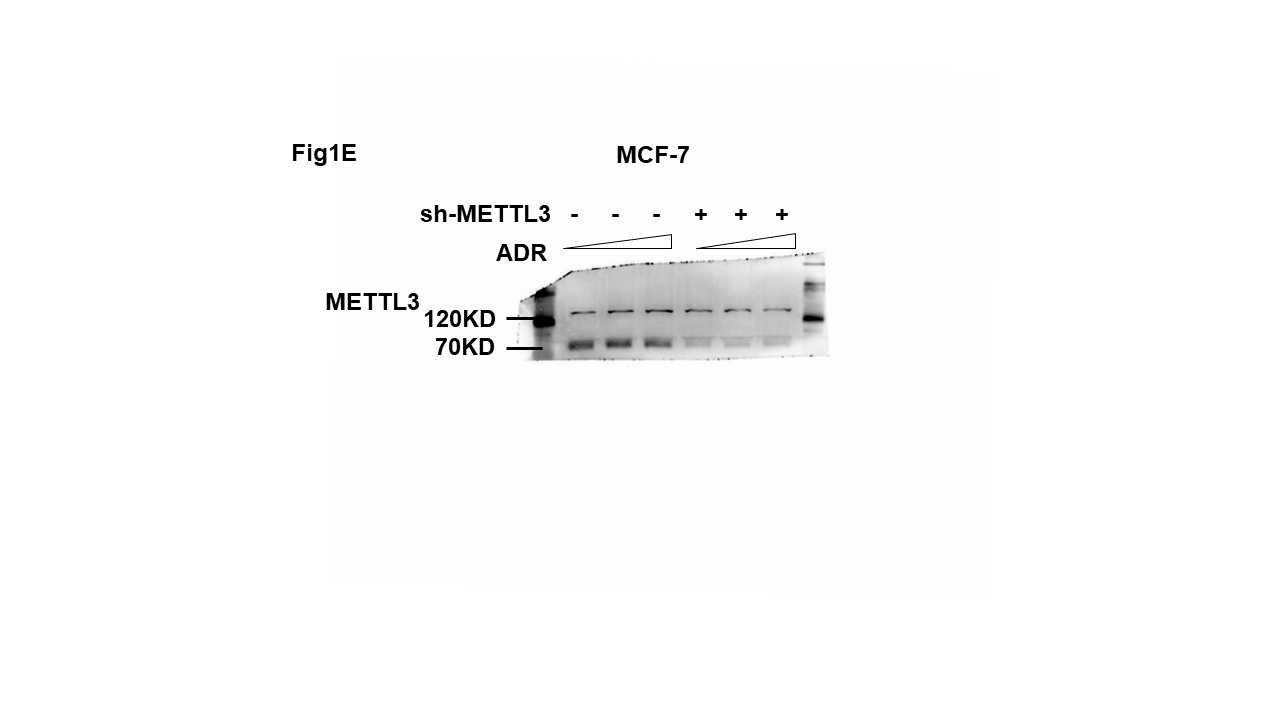

Supplement: Figure 1—source data 1. [file elife-75231-fig1-data1.zip › Figure 1e/Figure 1e METTL3.tif]

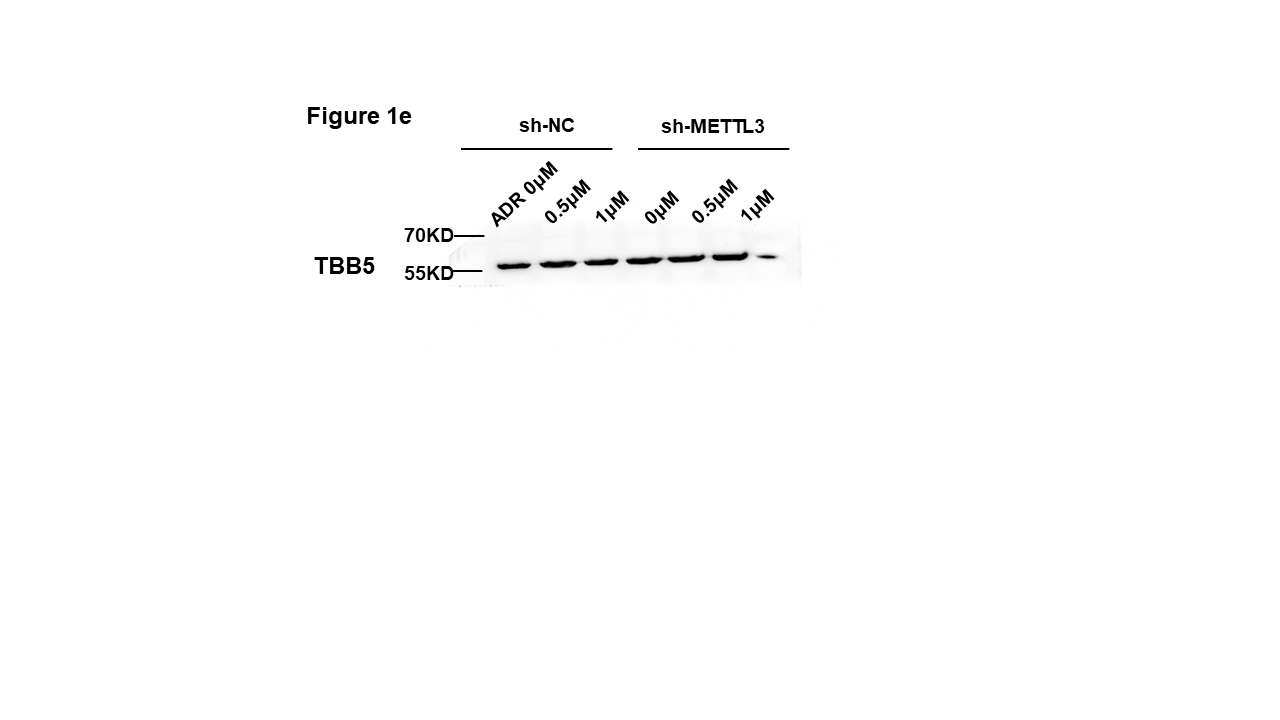

Supplement: Figure 1—source data 1. [file elife-75231-fig1-data1.zip › Figure 1e/Figure 1e TBB5.TIF]

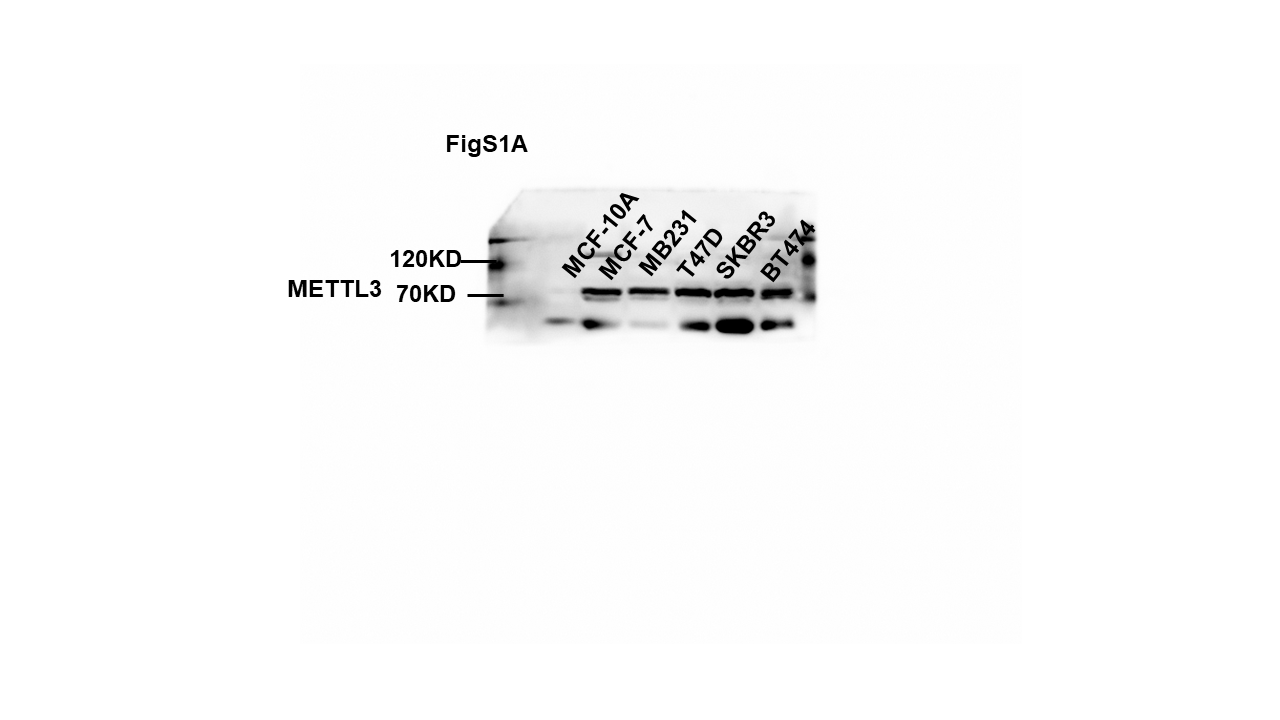

Supplement: Figure 1—figure supplement 1—source data 1. [file elife-75231-fig1-figsupp1-data1.zip › Figure S1A/FigureS1A METTL3.TIF]

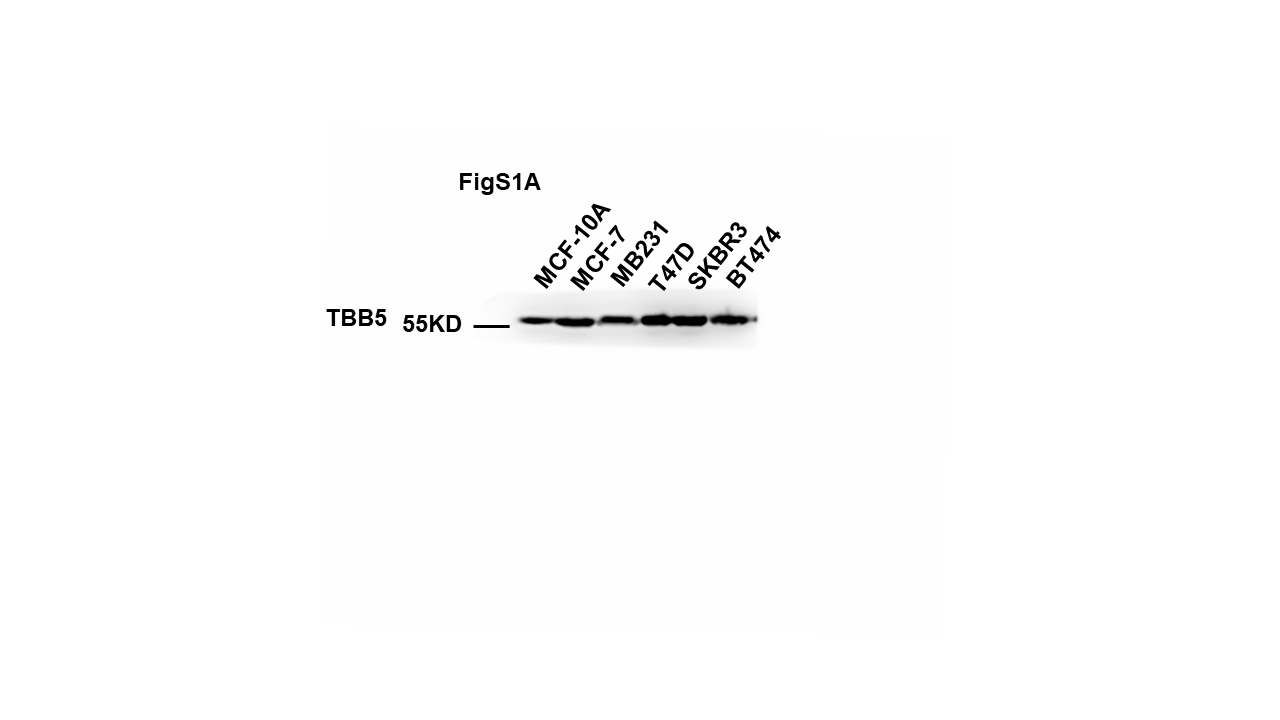

Supplement: Figure 1—figure supplement 1—source data 1. [file elife-75231-fig1-figsupp1-data1.zip › Figure S1A/FigureS1A TBB5.TIF]

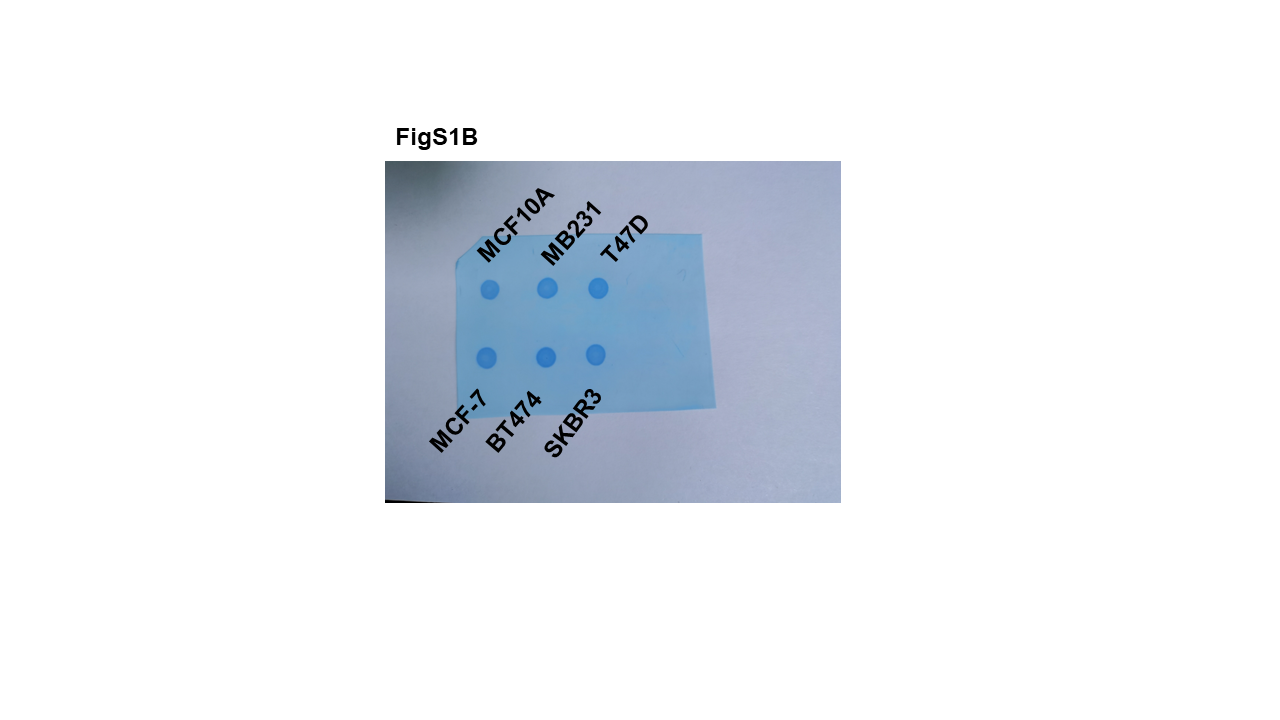

Supplement: Figure 1—figure supplement 1—source data 2. [file elife-75231-fig1-figsupp1-data2.zip › Figure S1B/FigureS1B (2).TIF]

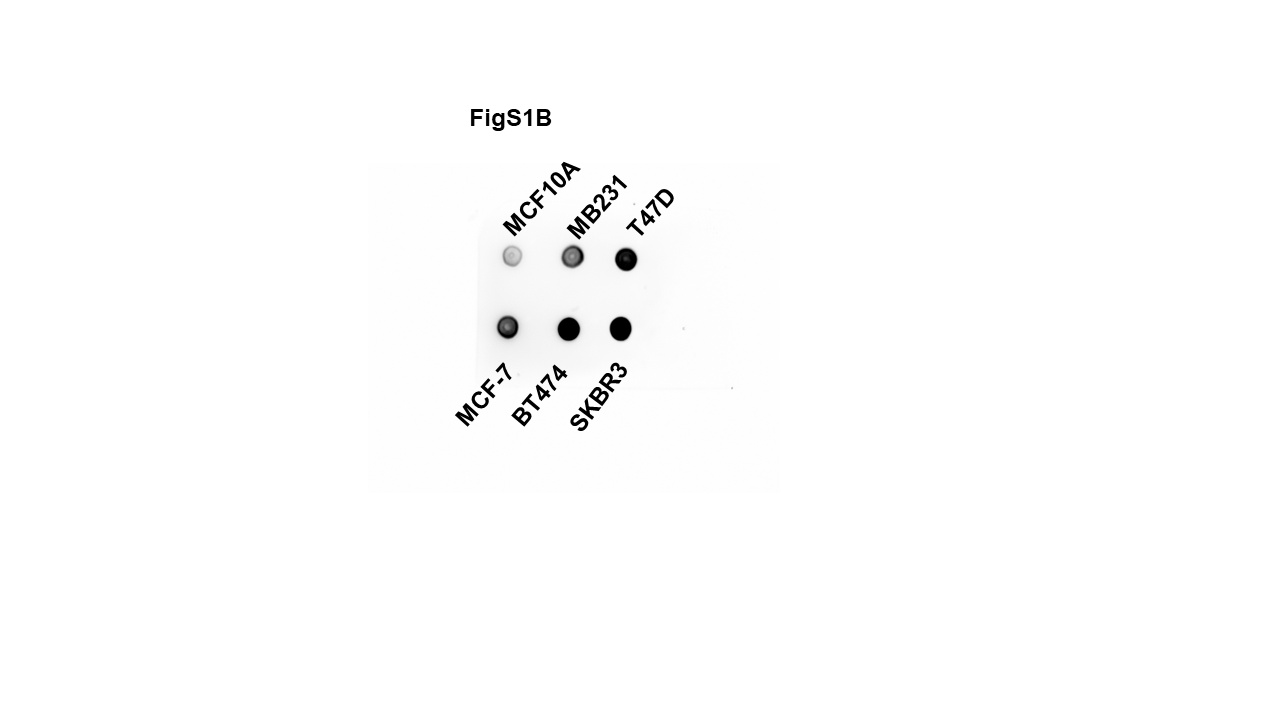

Supplement: Figure 1—figure supplement 1—source data 2. [file elife-75231-fig1-figsupp1-data2.zip › Figure S1B/FigureS1B.TIF]

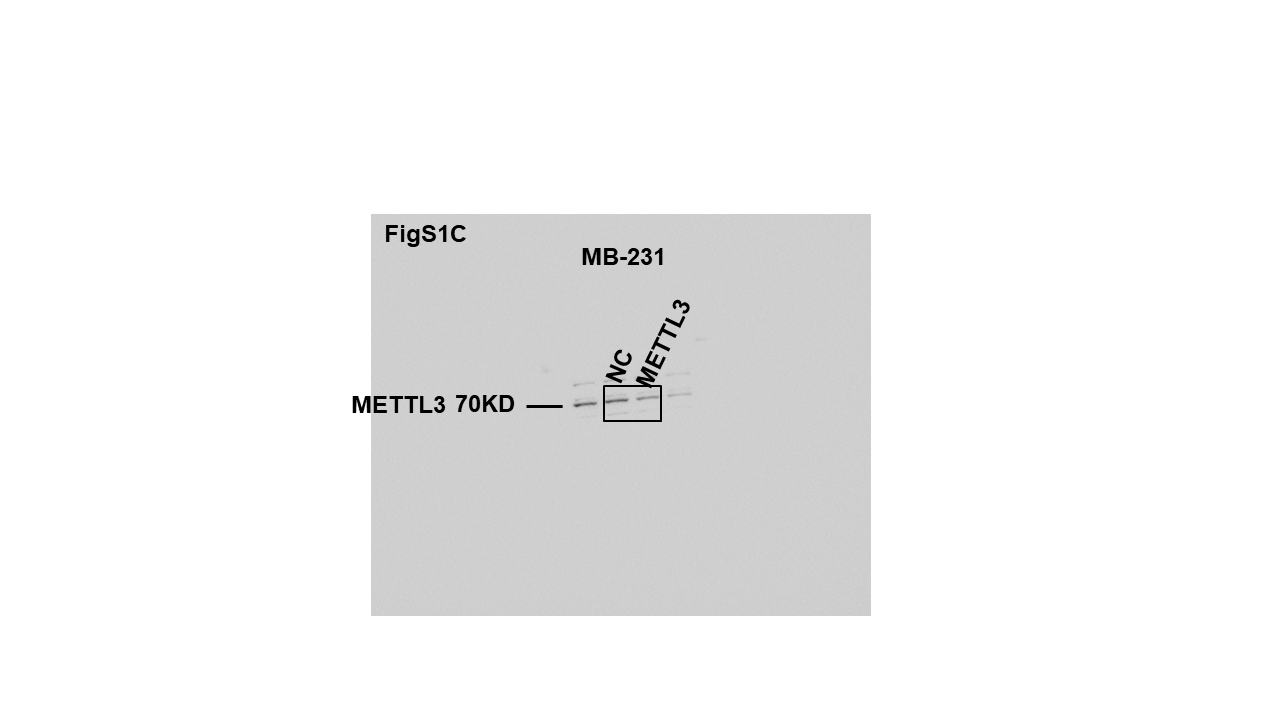

Supplement: Figure 1—figure supplement 1—source data 3. [file elife-75231-fig1-figsupp1-data3.zip › Figure S1C/FigureS1C MB231 METTL3.TIF]

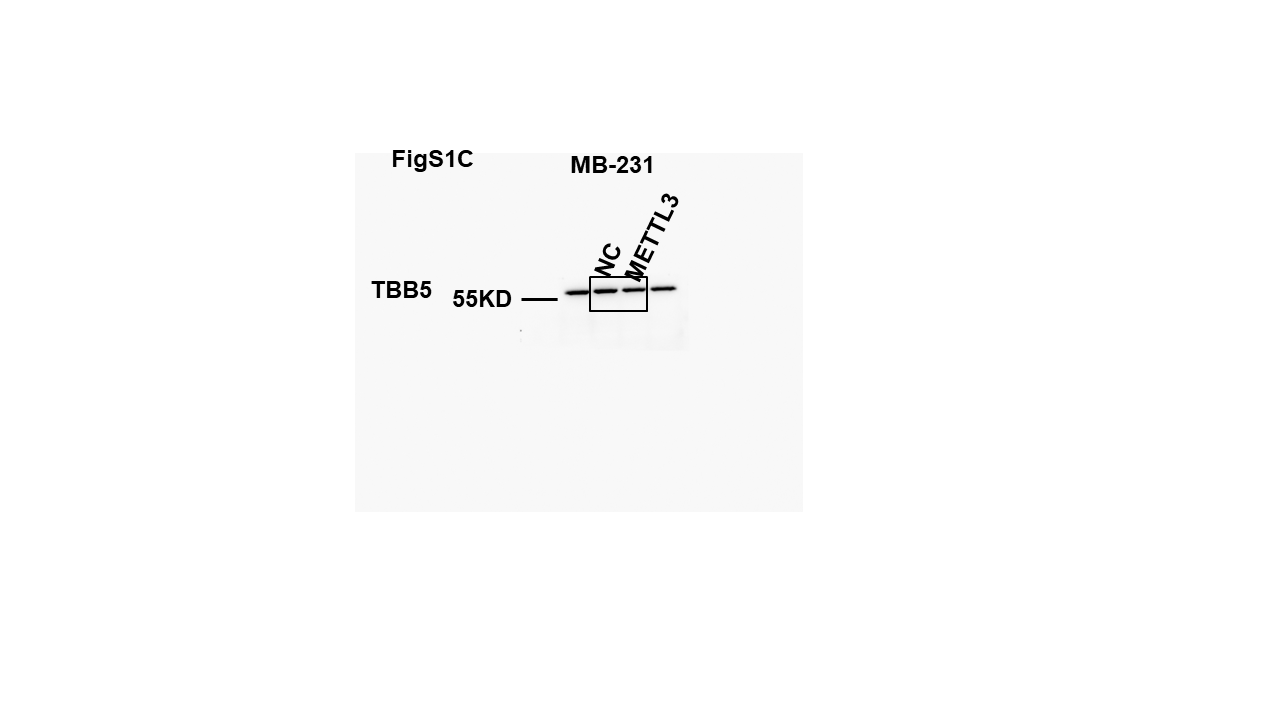

Supplement: Figure 1—figure supplement 1—source data 3. [file elife-75231-fig1-figsupp1-data3.zip › Figure S1C/FigureS1C MB231 TBB5.TIF]

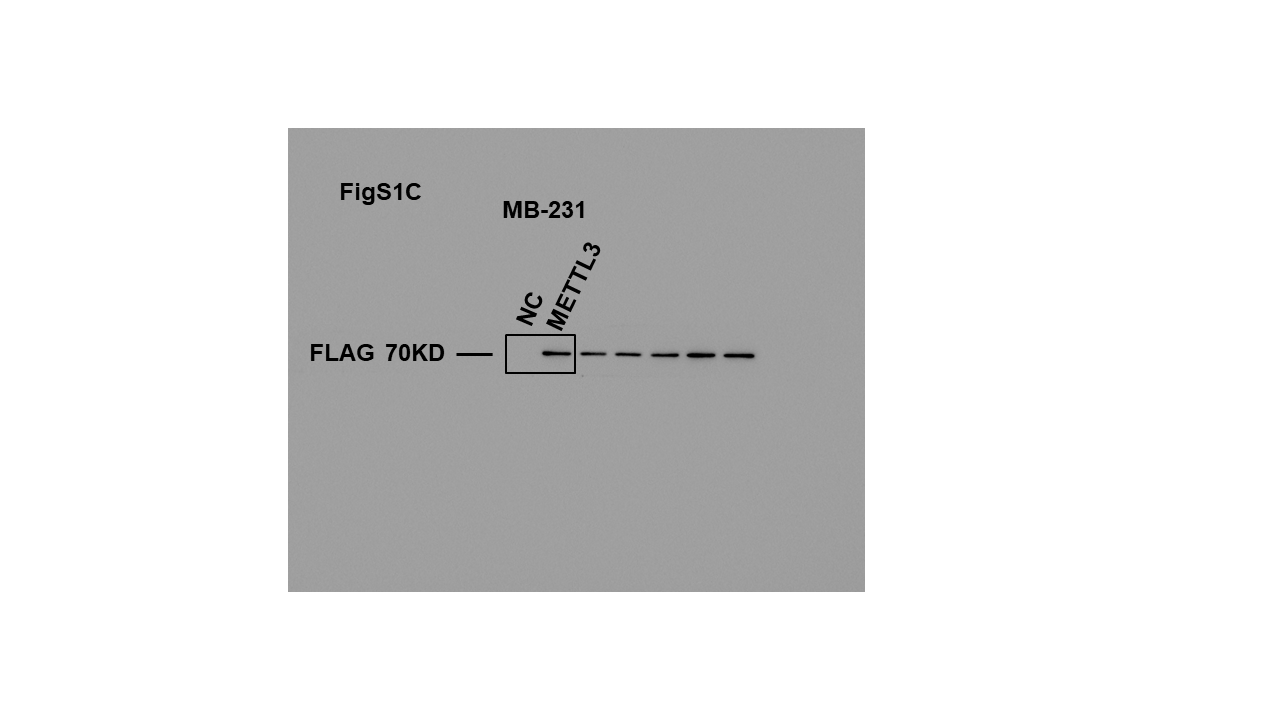

Supplement: Figure 1—figure supplement 1—source data 3. [file elife-75231-fig1-figsupp1-data3.zip › Figure S1C/FigureS1C MB231 FLAG.TIF]

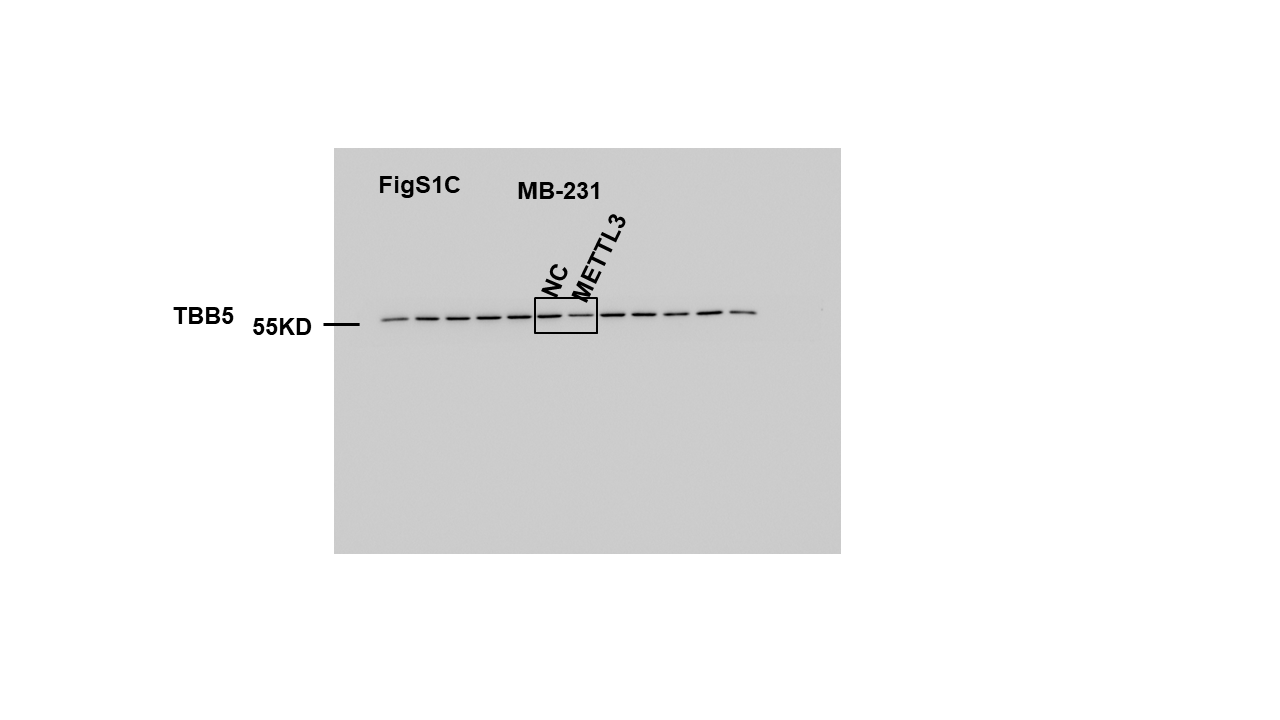

Supplement: Figure 1—figure supplement 1—source data 3. [file elife-75231-fig1-figsupp1-data3.zip › Figure S1C/FigureS1C MB231 TBB5.TIF]

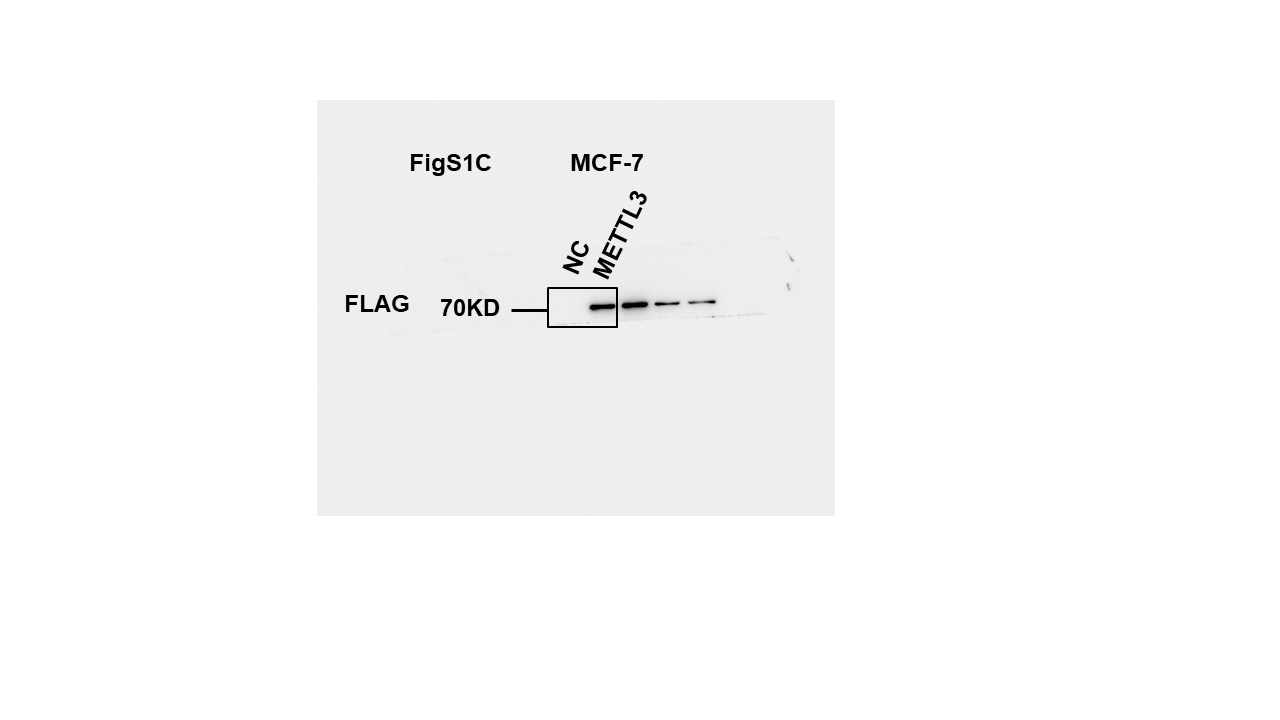

Supplement: Figure 1—figure supplement 1—source data 3. [file elife-75231-fig1-figsupp1-data3.zip › Figure S1C/FigureS1C MCF-7 FLAG.TIF]

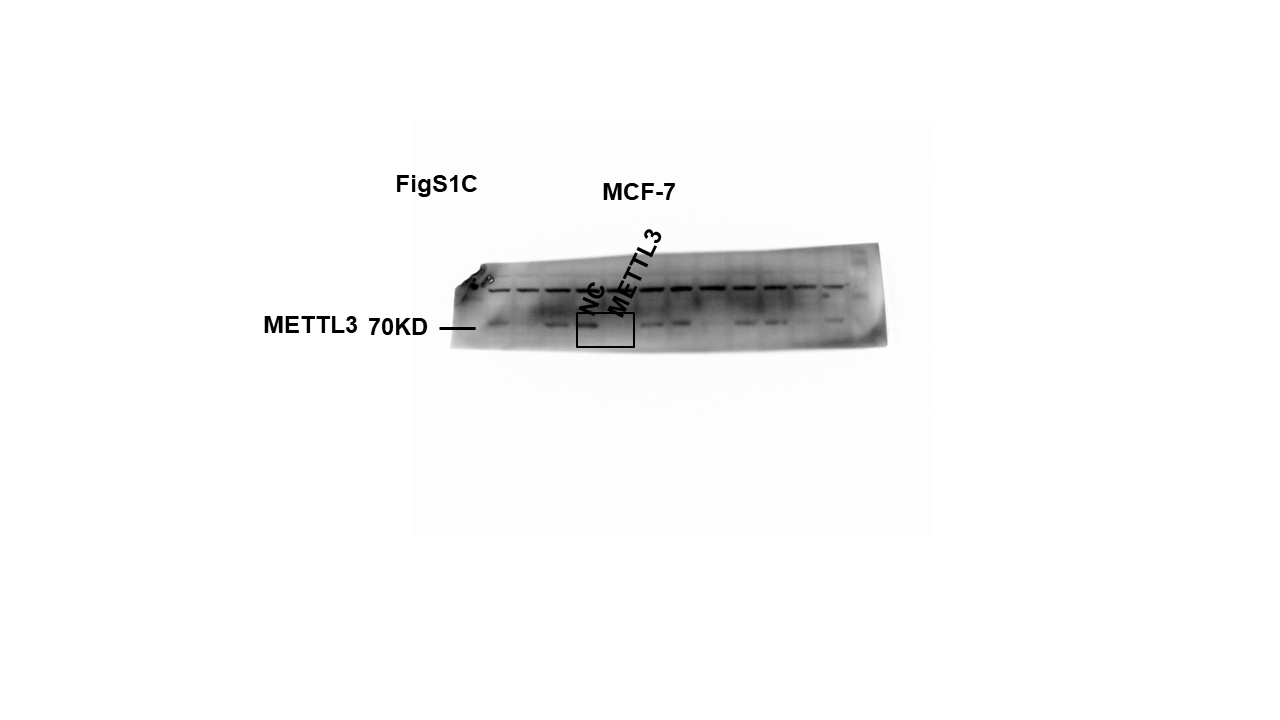

Supplement: Figure 1—figure supplement 1—source data 3. [file elife-75231-fig1-figsupp1-data3.zip › Figure S1C/FigureS1C MCF-7 METTL3.TIF]

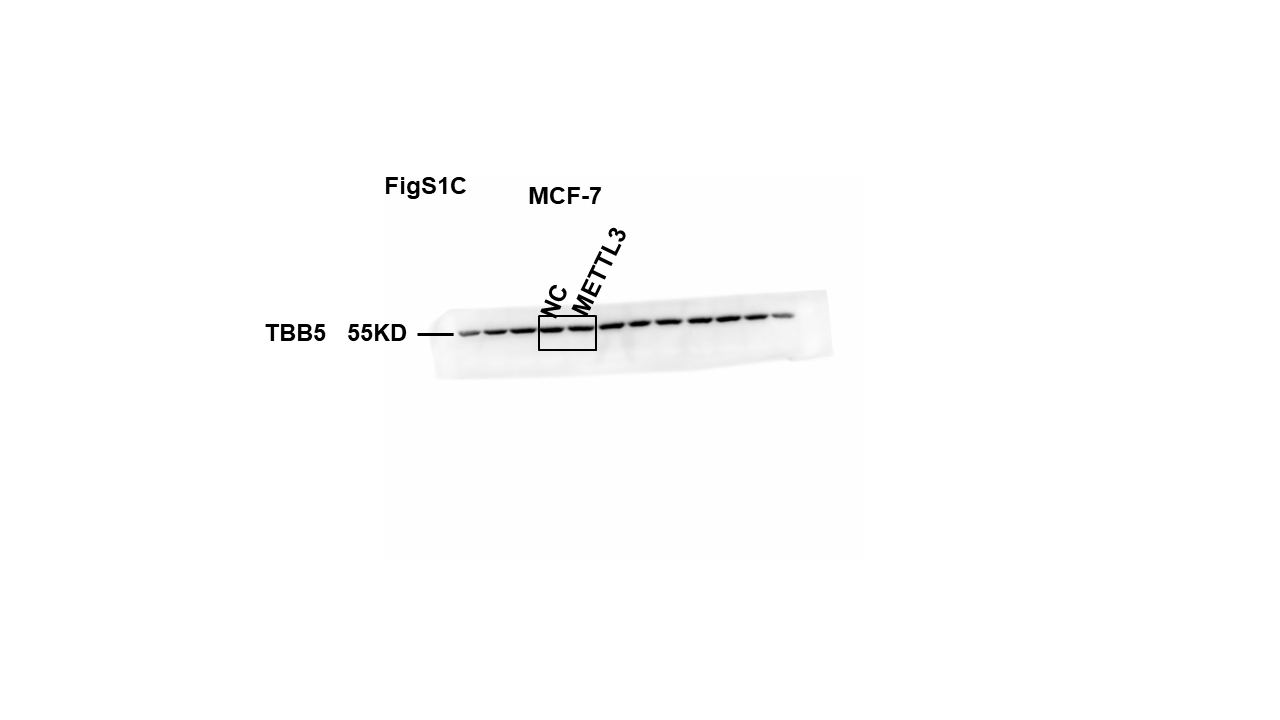

Supplement: Figure 1—figure supplement 1—source data 3. [file elife-75231-fig1-figsupp1-data3.zip › Figure S1C/FigureS1CMCF-7 TBB5 (2).TIF]

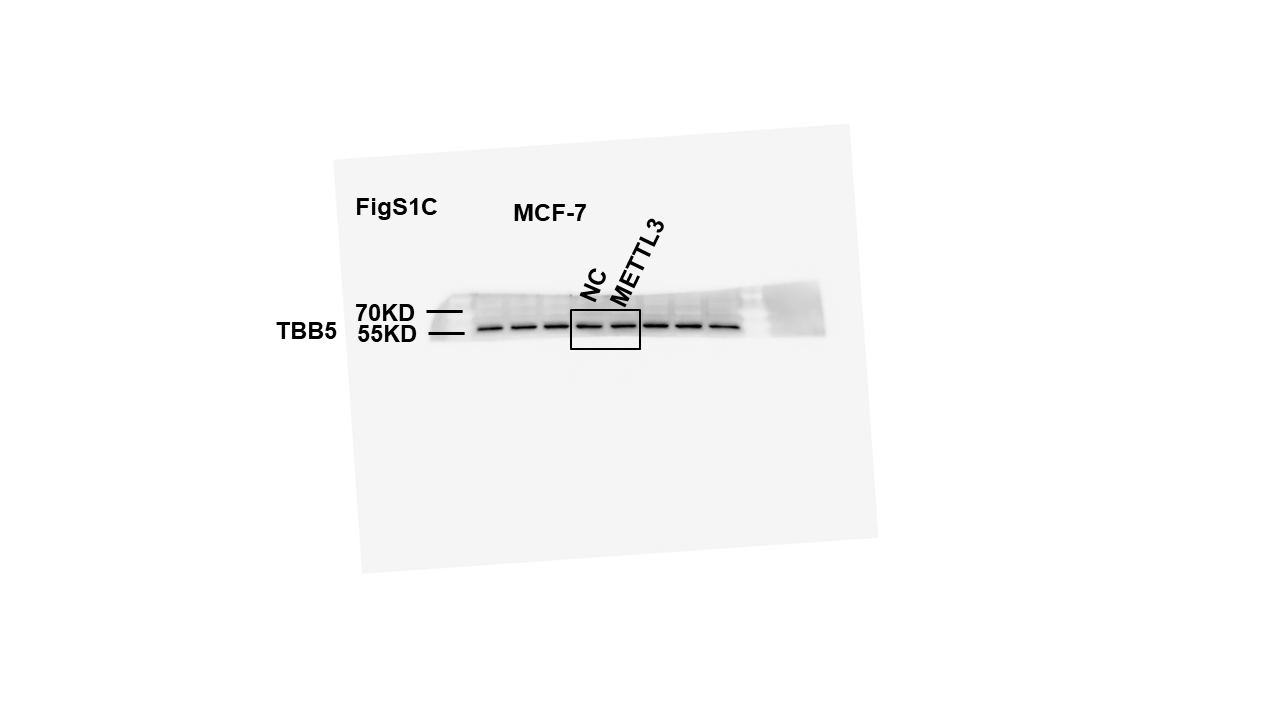

Supplement: Figure 1—figure supplement 1—source data 3. [file elife-75231-fig1-figsupp1-data3.zip › Figure S1C/FigureS1CMCF-7 TBB5.TIF]

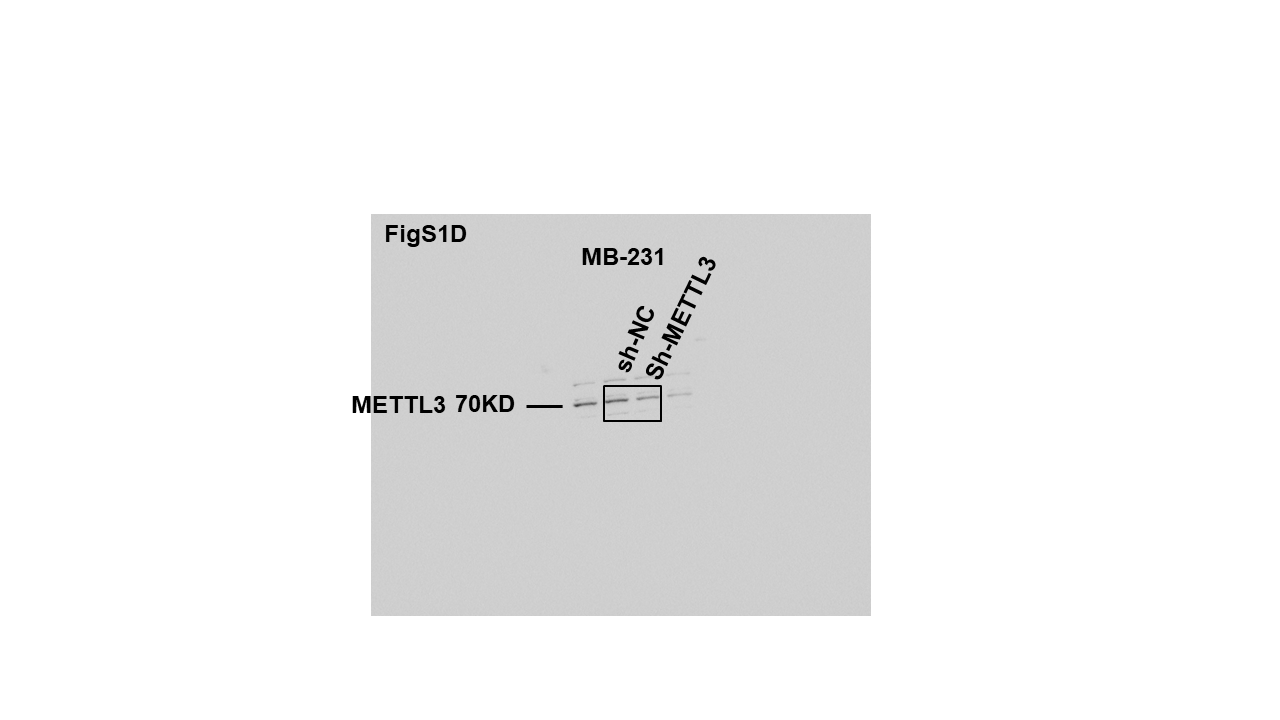

Supplement: Figure 1—figure supplement 1—source data 4. [file elife-75231-fig1-figsupp1-data4.zip › Figure S1D/Figure S1D MB231 METTL3.tif]

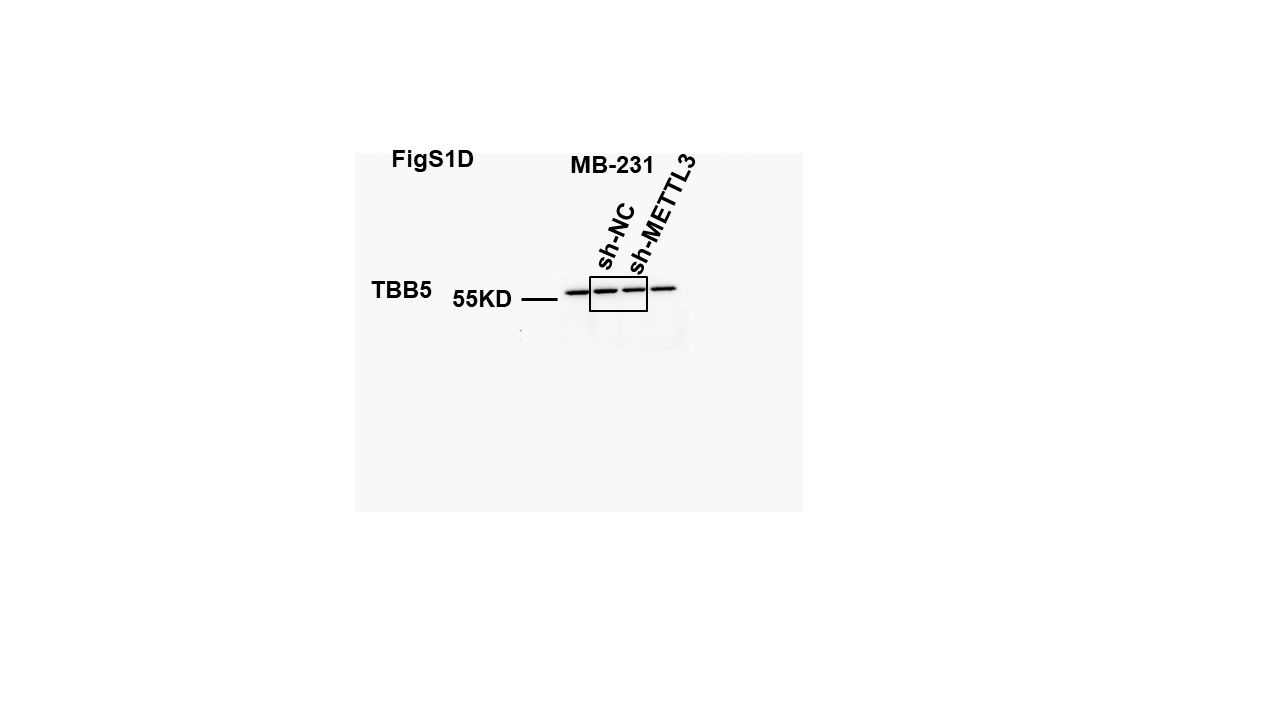

Supplement: Figure 1—figure supplement 1—source data 4. [file elife-75231-fig1-figsupp1-data4.zip › Figure S1D/Figure S1D MB231 TBB5.tif]

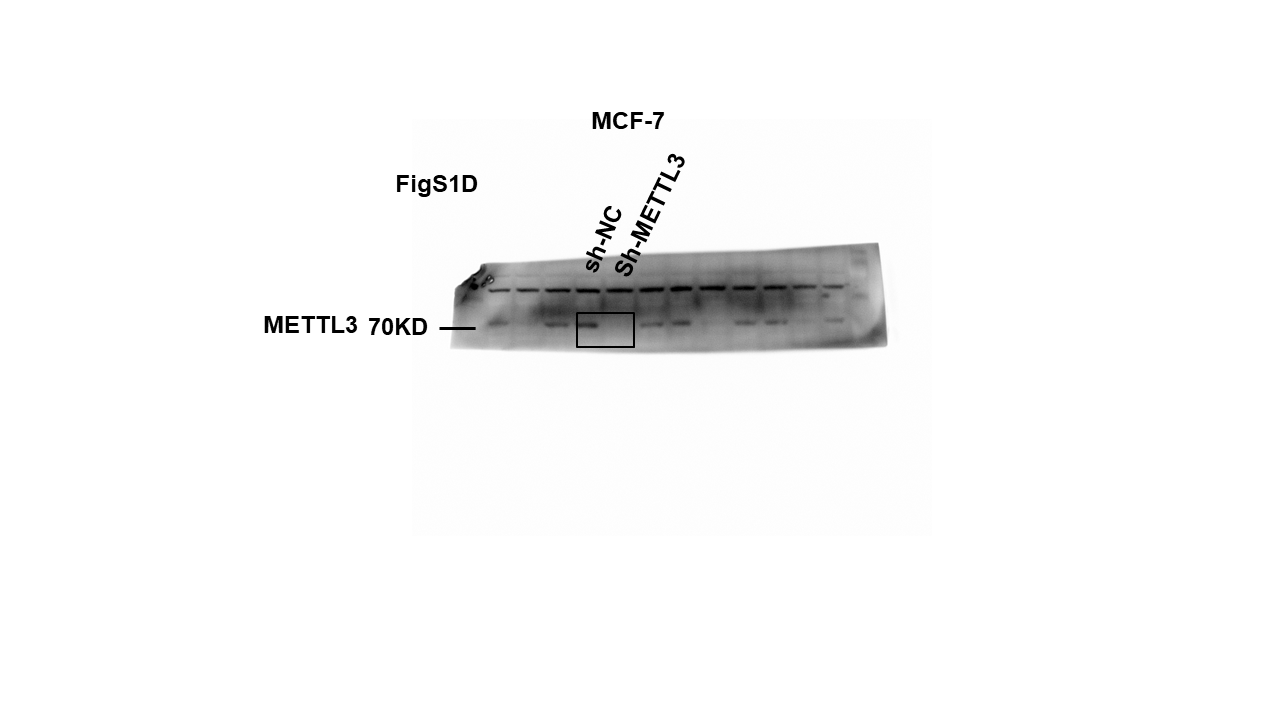

Supplement: Figure 1—figure supplement 1—source data 4. [file elife-75231-fig1-figsupp1-data4.zip › Figure S1D/Figure S1D MCF-7 METTL3.tif]

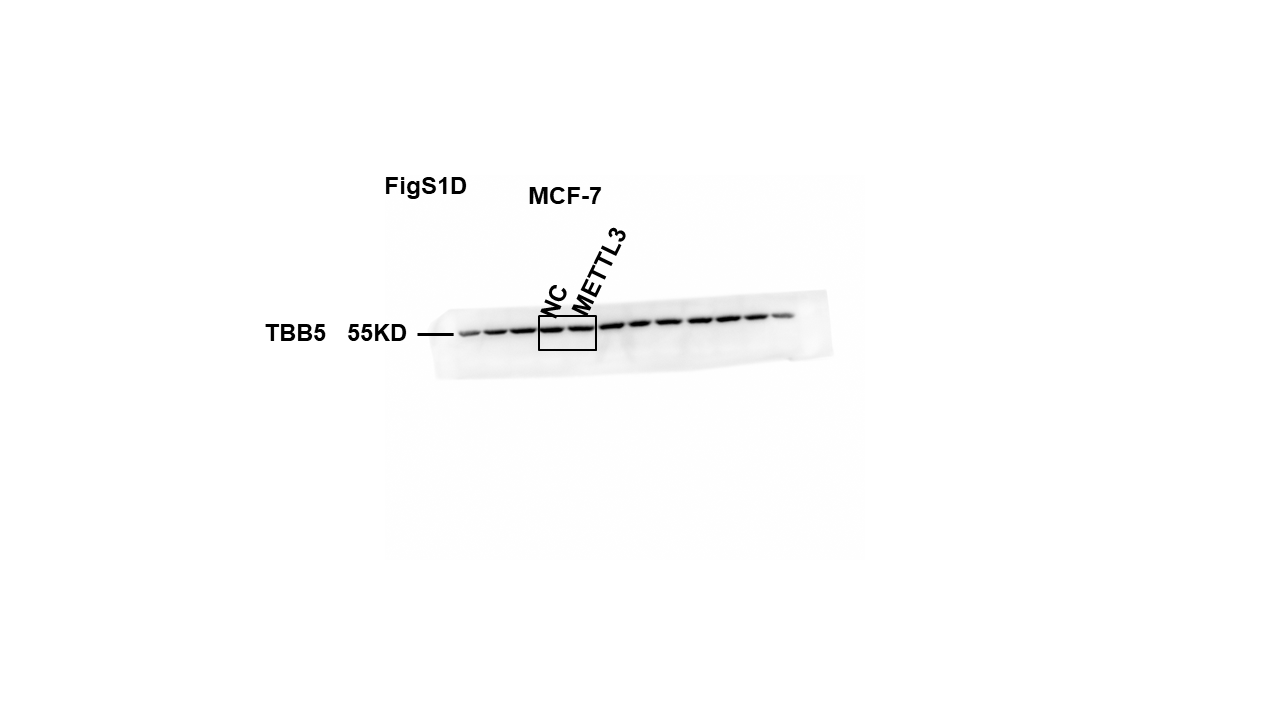

Supplement: Figure 1—figure supplement 1—source data 4. [file elife-75231-fig1-figsupp1-data4.zip › Figure S1D/Figure S1D MCF-7 TBB5.tif]

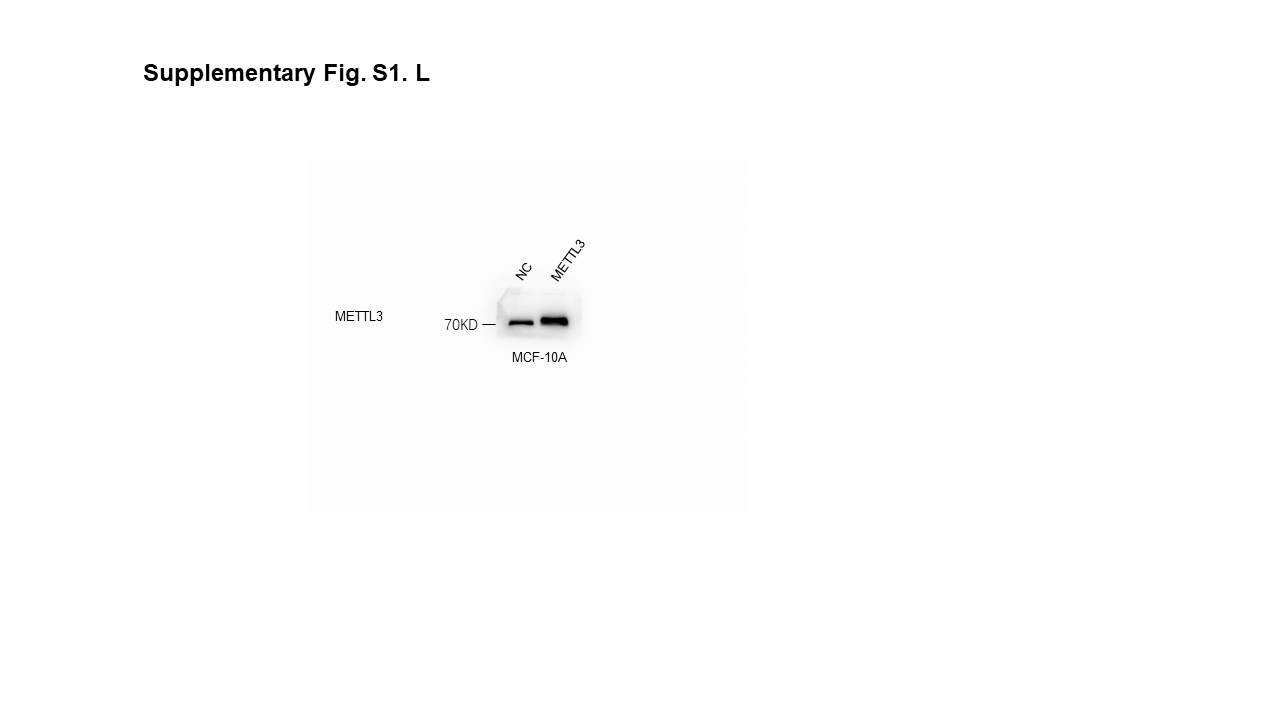

Supplement: Figure 1—figure supplement 1—source data 5. [file elife-75231-fig1-figsupp1-data5.zip › Figure S1L/Figure S1L METTL3.TIF]

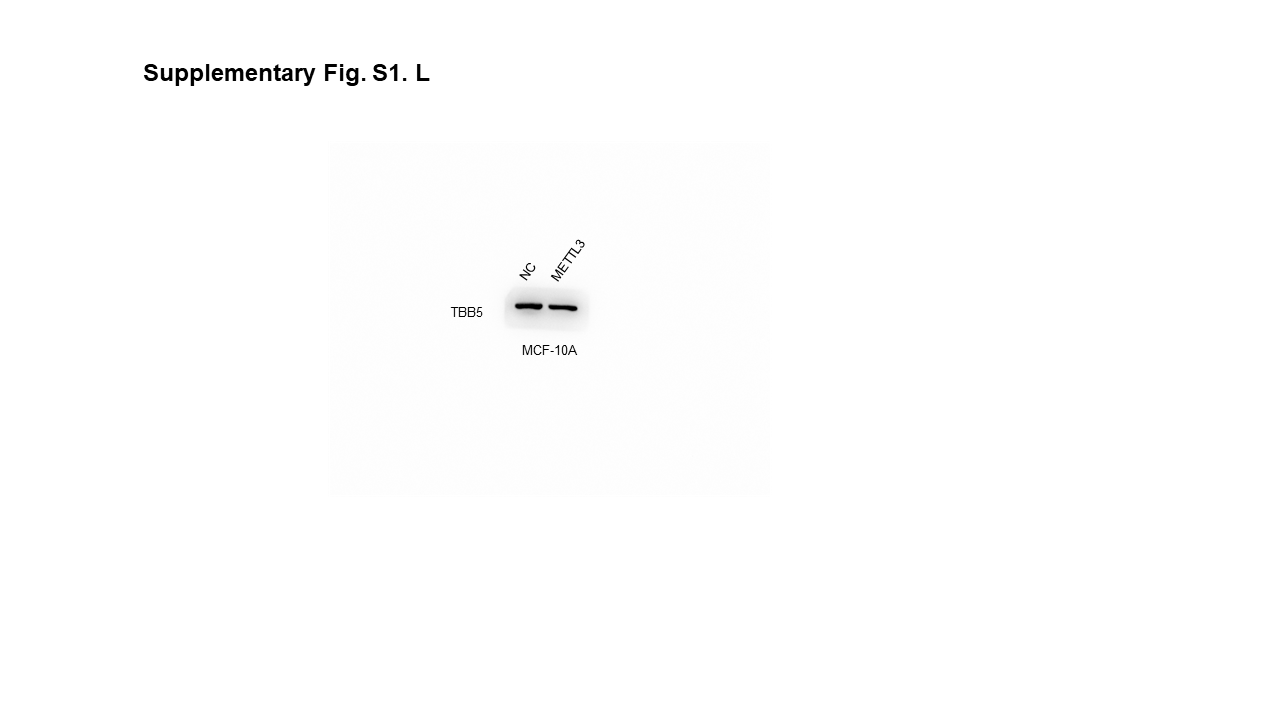

Supplement: Figure 1—figure supplement 1—source data 5. [file elife-75231-fig1-figsupp1-data5.zip › Figure S1L/Figure S1L TBB2.TIF]

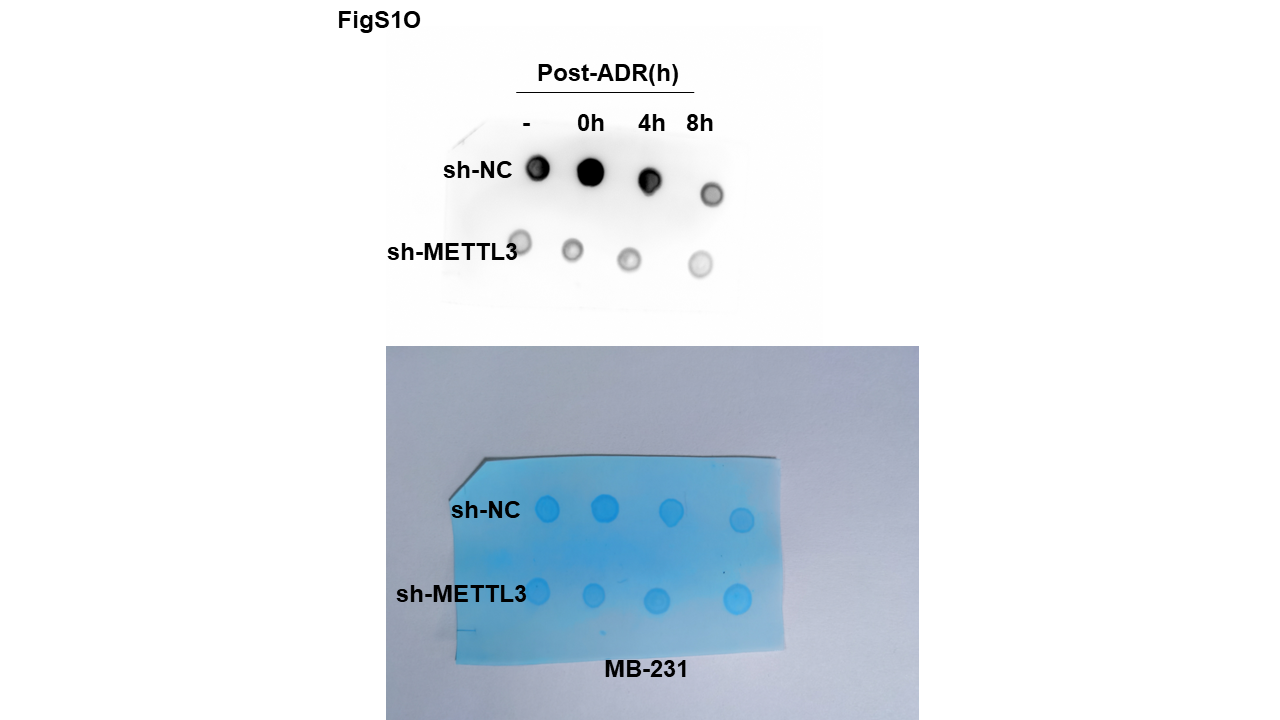

Supplement: Figure 1—figure supplement 1—source data 6. [file elife-75231-fig1-figsupp1-data6.zip › Figure S1O/Figure S1O MB231.tif]

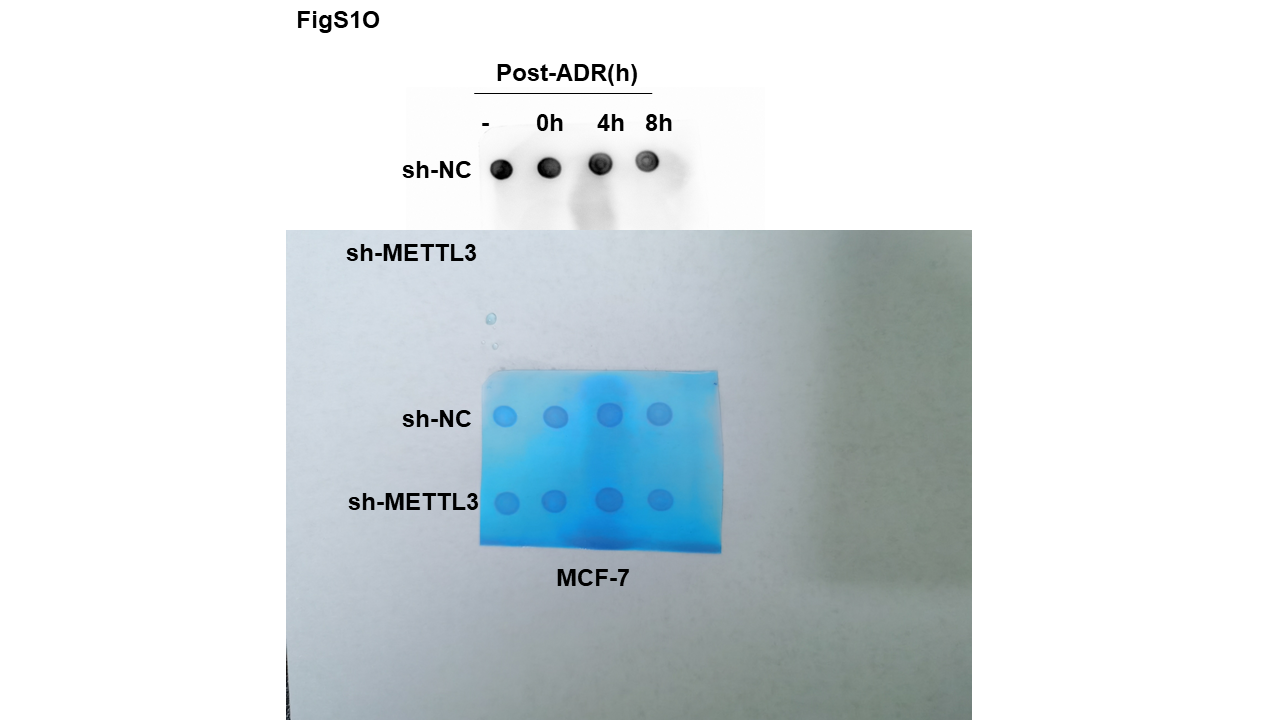

Supplement: Figure 1—figure supplement 1—source data 6. [file elife-75231-fig1-figsupp1-data6.zip › Figure S1O/Figure S1O MCF-7.tif]

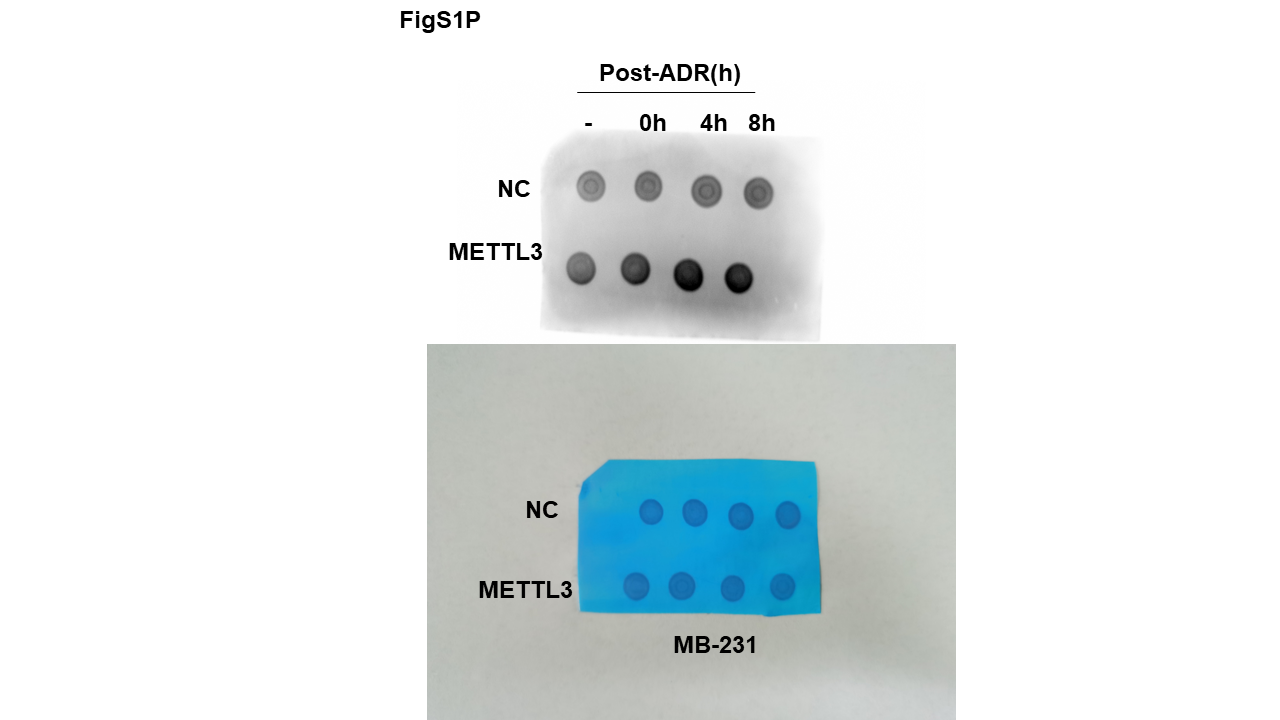

Supplement: Figure 1—figure supplement 1—source data 7. [file elife-75231-fig1-figsupp1-data7.zip › Figure S1P/Figure S1P MB231.tif]

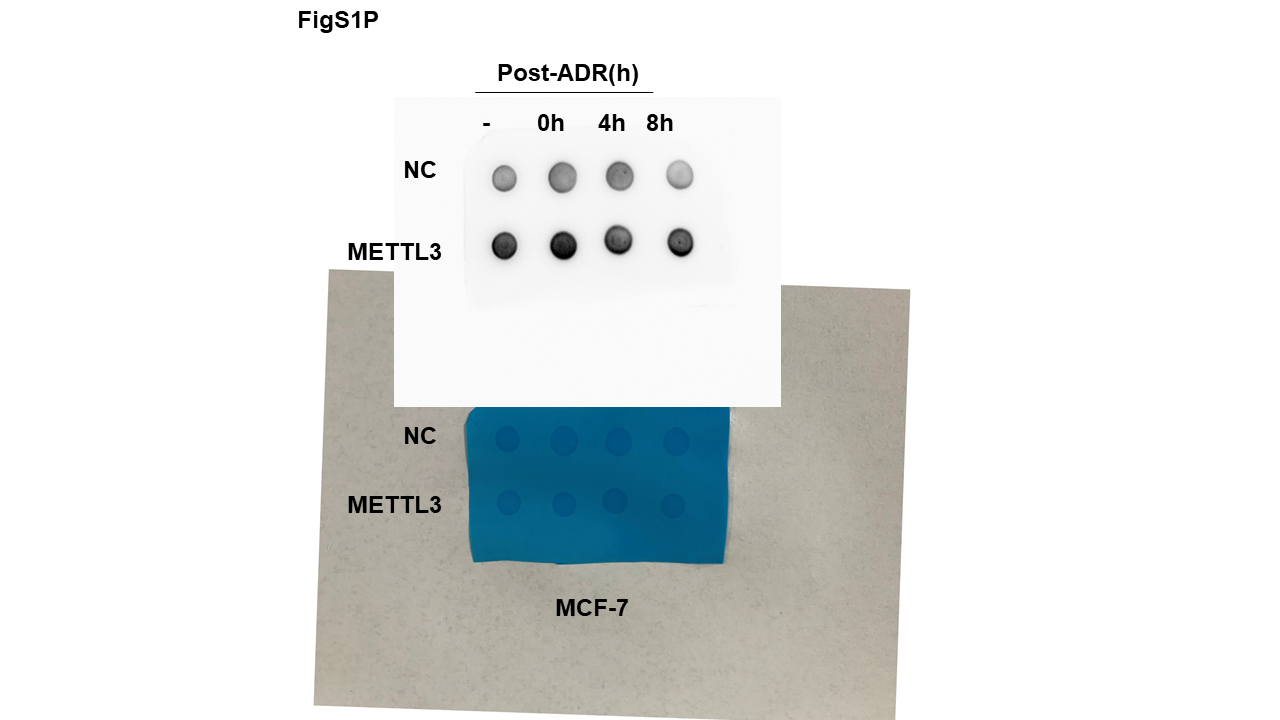

Supplement: Figure 1—figure supplement 1—source data 7. [file elife-75231-fig1-figsupp1-data7.zip › Figure S1P/Figure S1P MCF-7.tif]

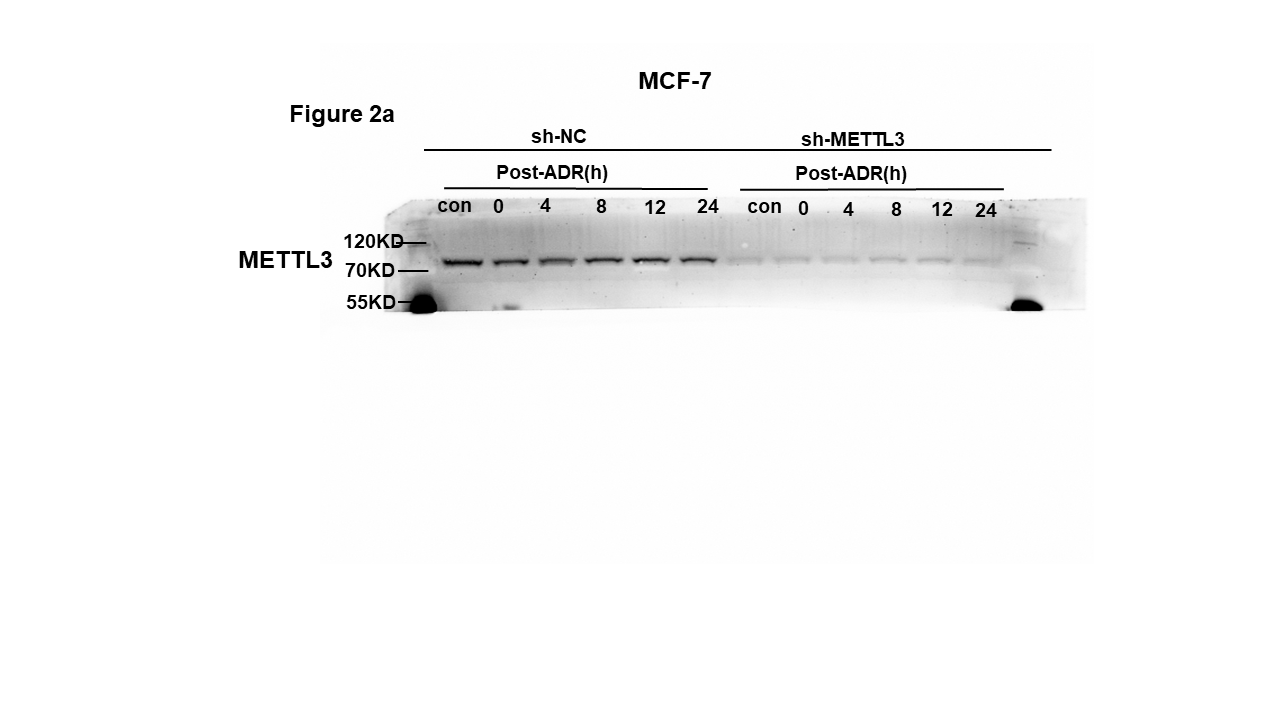

Supplement: Figure 2—source data 1. [file elife-75231-fig2-data1.zip › Figure 2a/Figure 2a METTL3.TIF]

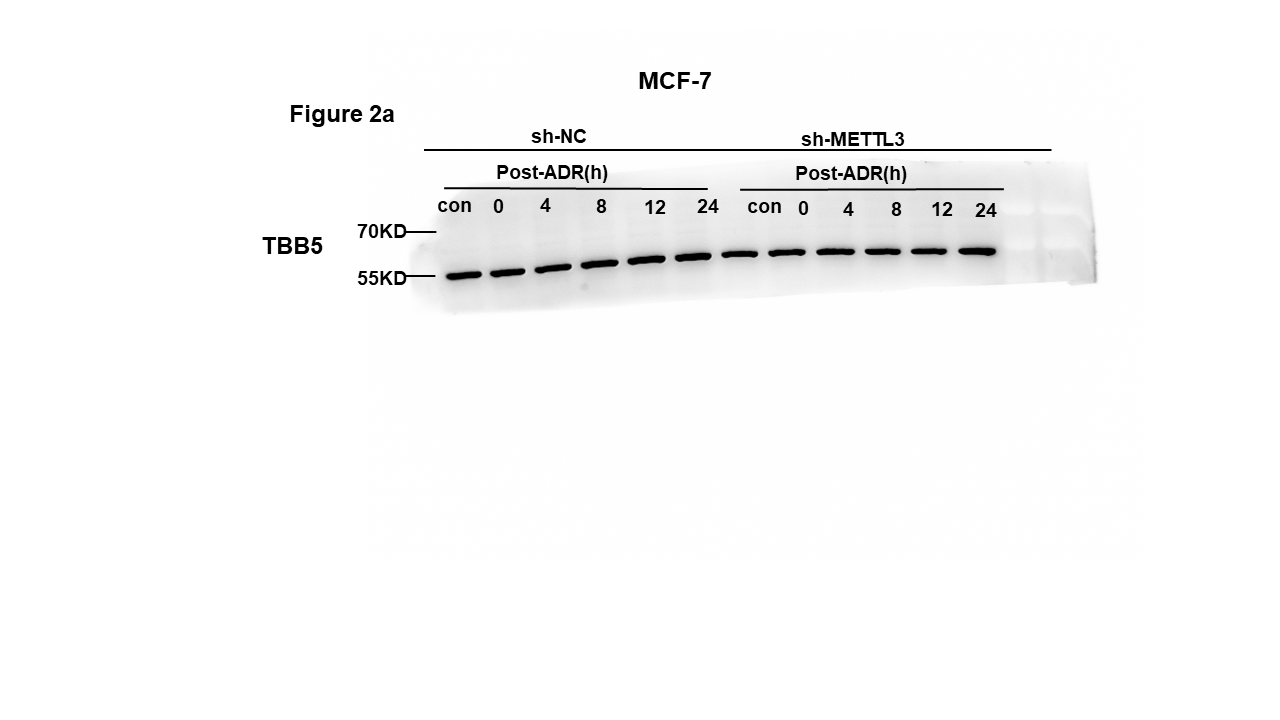

Supplement: Figure 2—source data 1. [file elife-75231-fig2-data1.zip › Figure 2a/Figure 2a TBB5.TIF]

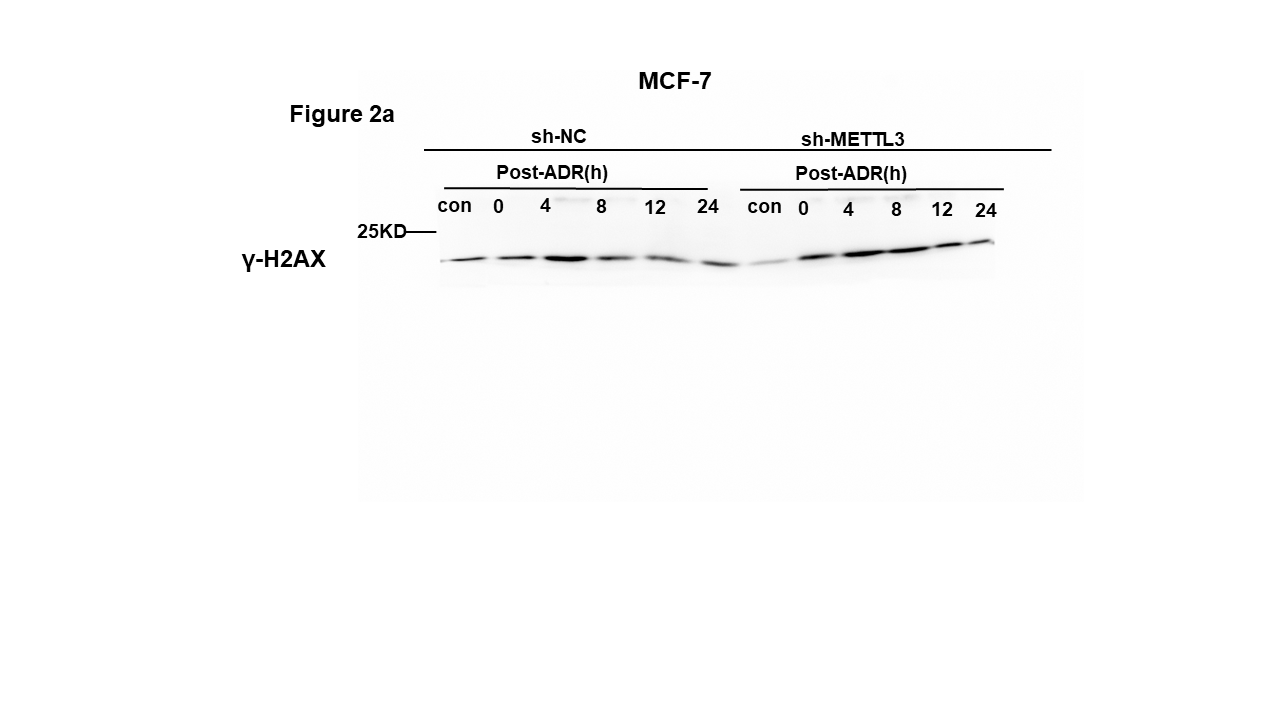

Supplement: Figure 2—source data 1. [file elife-75231-fig2-data1.zip › Figure 2a/Figure 2a γ-H2AX.TIF]

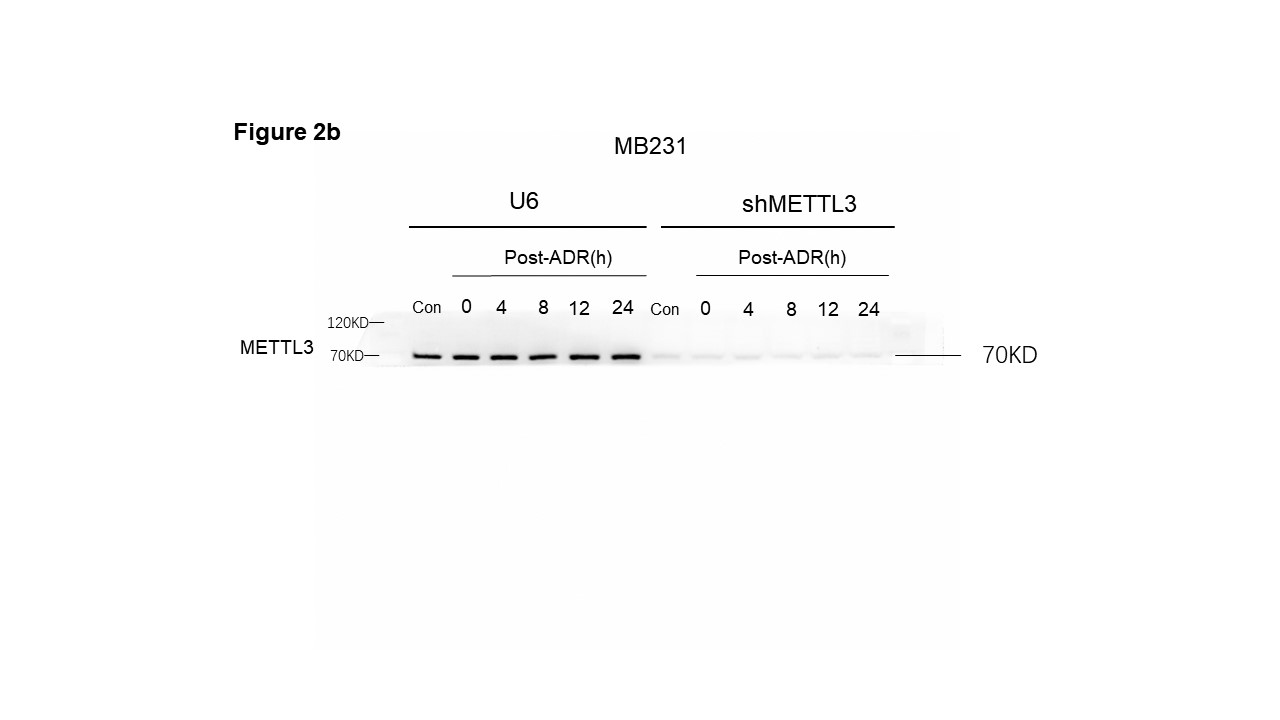

Supplement: Figure 2—source data 2. [file elife-75231-fig2-data2.zip › Figure 2b/Figure 2b mettl3.JPG]

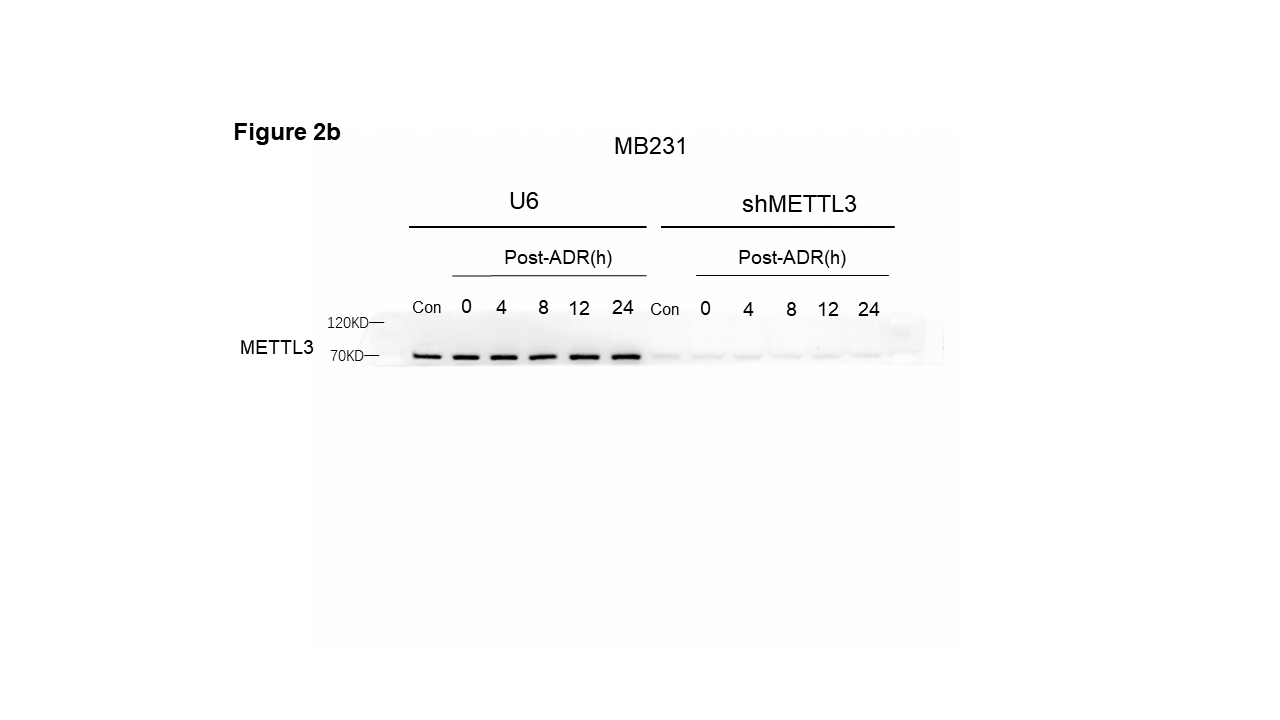

Supplement: Figure 2—source data 2. [file elife-75231-fig2-data2.zip › Figure 2b/Figure 2b mettl3.TIF]

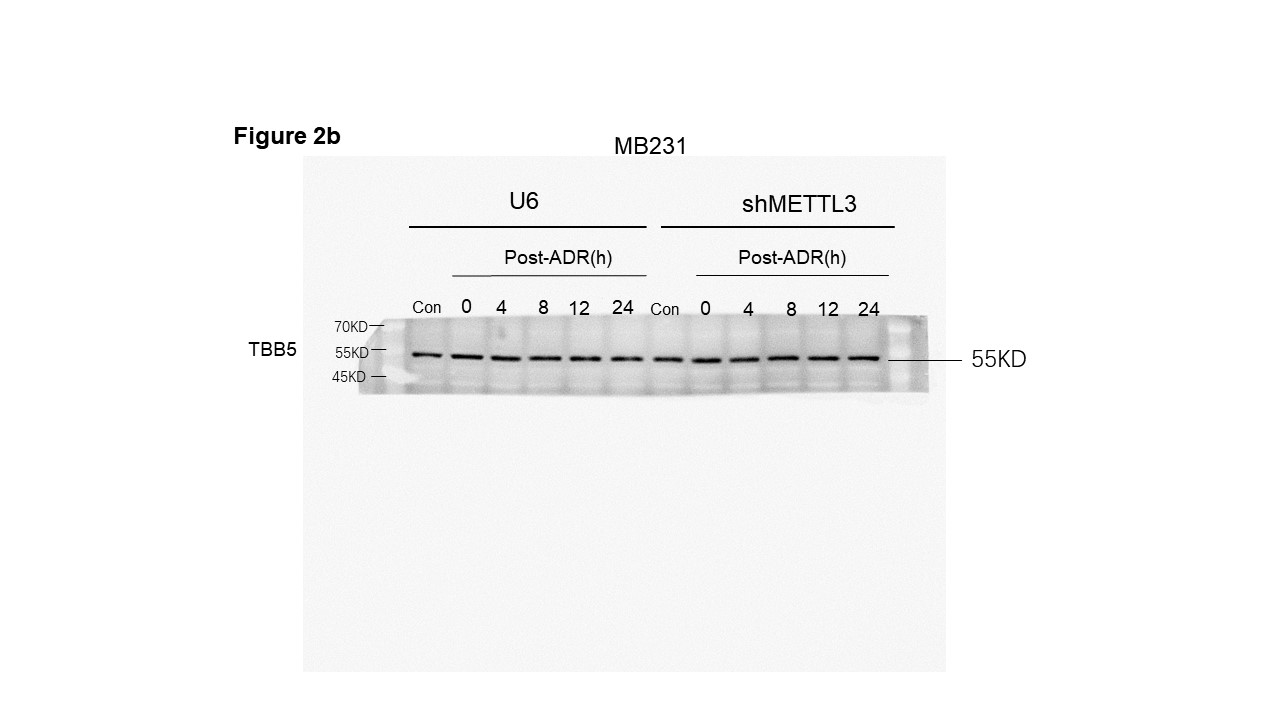

Supplement: Figure 2—source data 2. [file elife-75231-fig2-data2.zip › Figure 2b/Figure 2b tbb5.JPG]

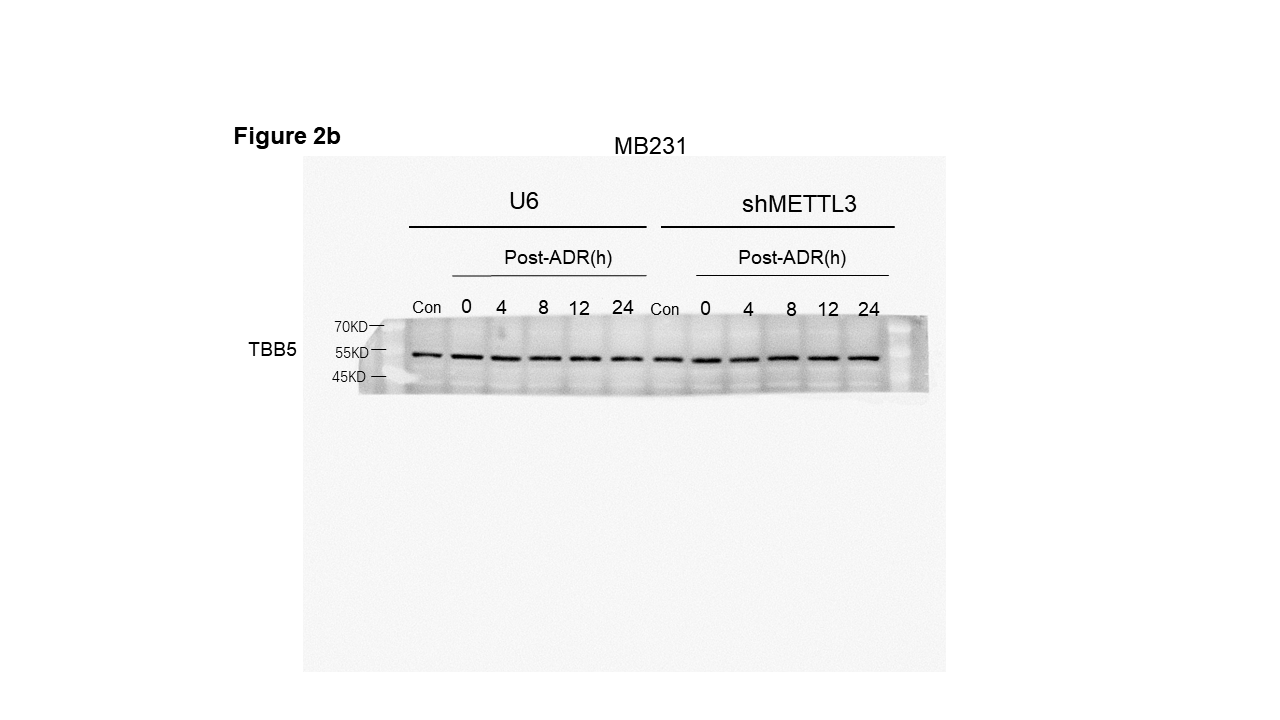

Supplement: Figure 2—source data 2. [file elife-75231-fig2-data2.zip › Figure 2b/Figure 2b tbb5.TIF]

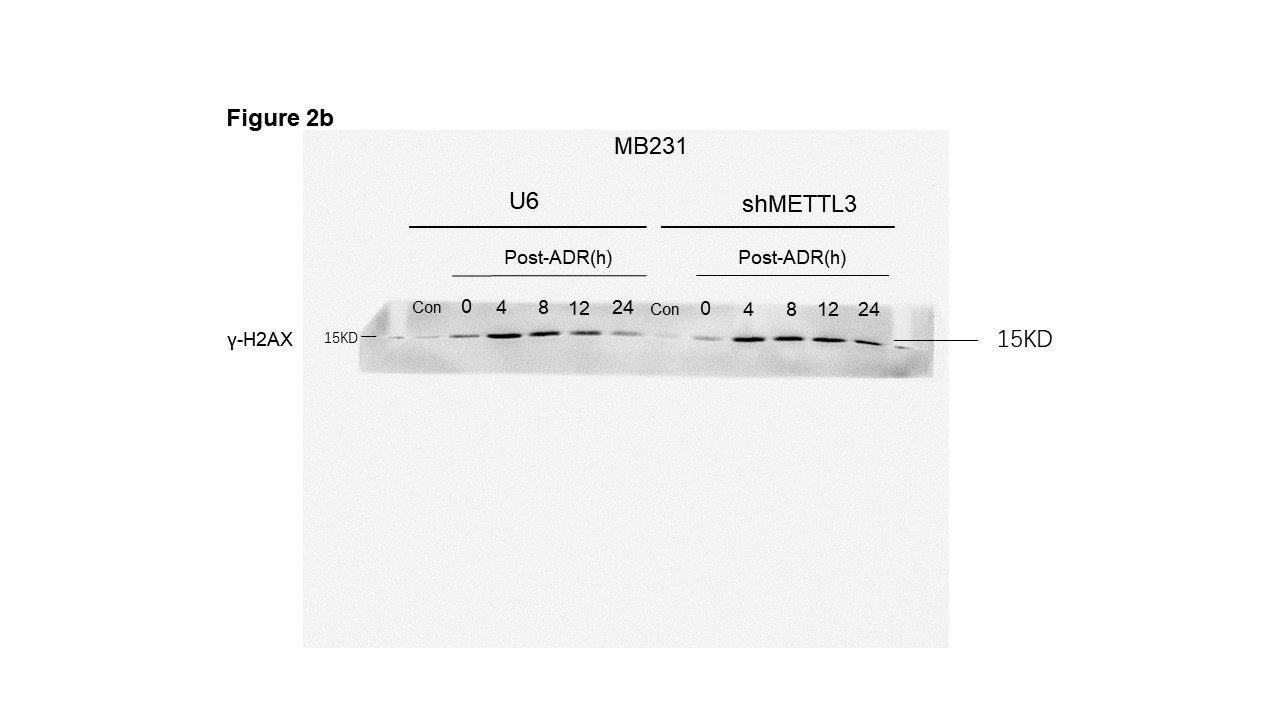

Supplement: Figure 2—source data 2. [file elife-75231-fig2-data2.zip › Figure 2b/Figure 2b γh2ax.JPG]

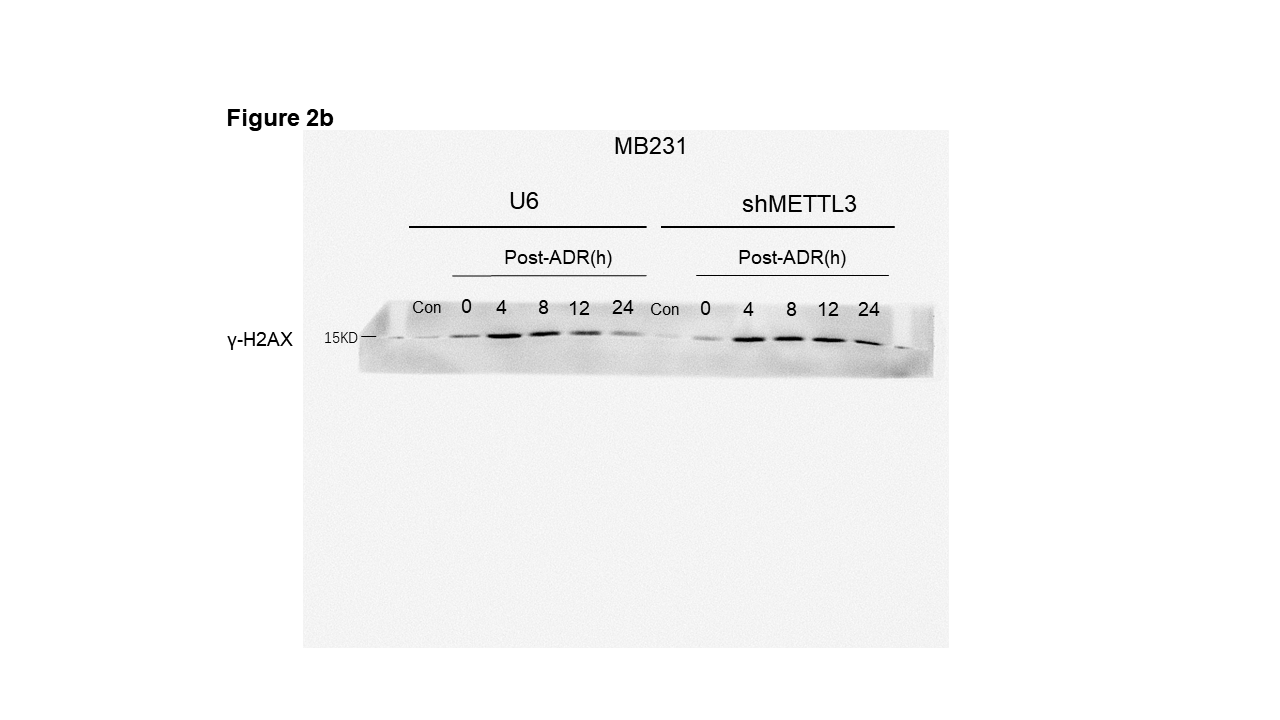

Supplement: Figure 2—source data 2. [file elife-75231-fig2-data2.zip › Figure 2b/Figure 2b γh2ax.TIF]

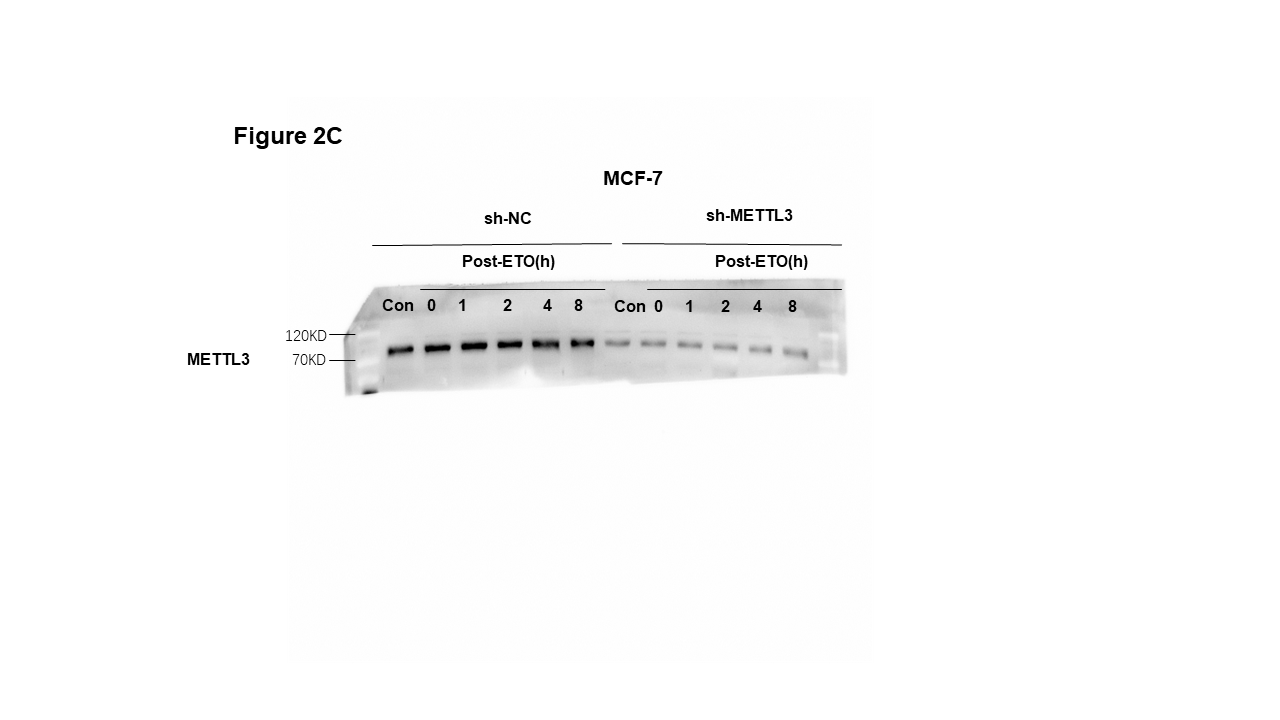

Supplement: Figure 2—source data 3. [file elife-75231-fig2-data3.zip › Figure 2C/Figure 2C METTL3.TIF]

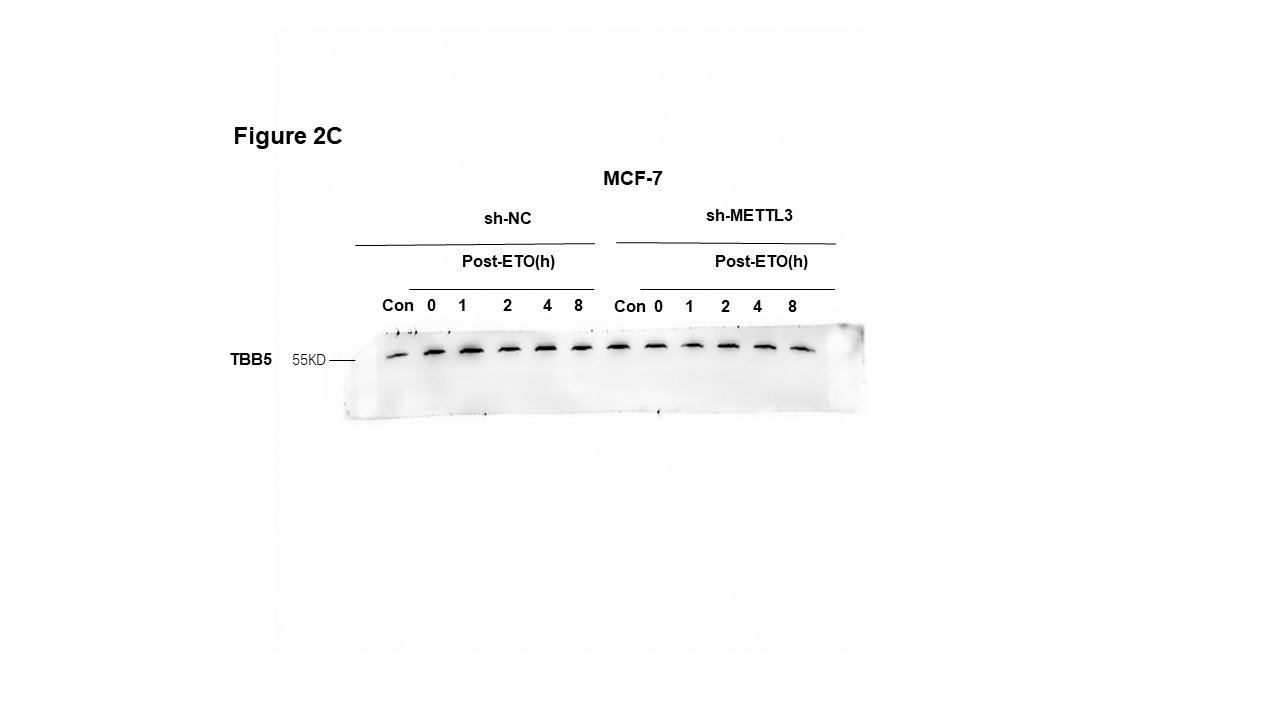

Supplement: Figure 2—source data 3. [file elife-75231-fig2-data3.zip › Figure 2C/Figure 2C TBB5.TIF]

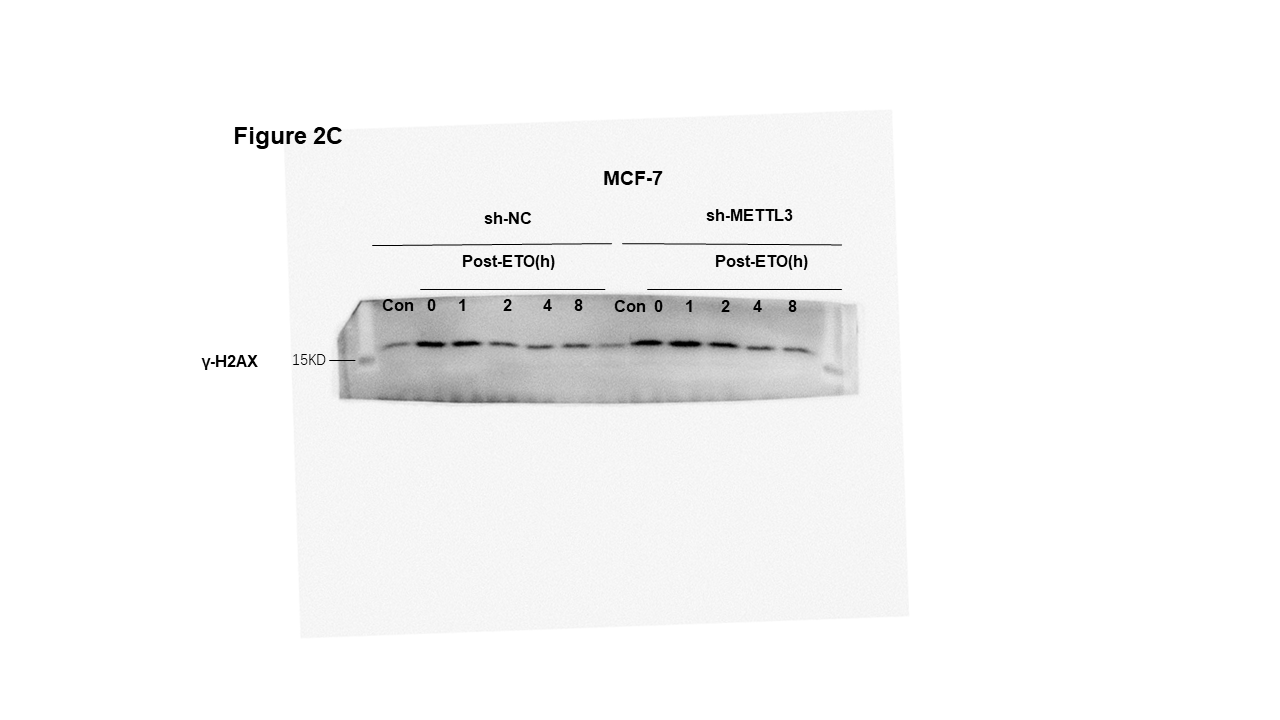

Supplement: Figure 2—source data 3. [file elife-75231-fig2-data3.zip › Figure 2C/Figure 2C a├-H2AX.TIF]

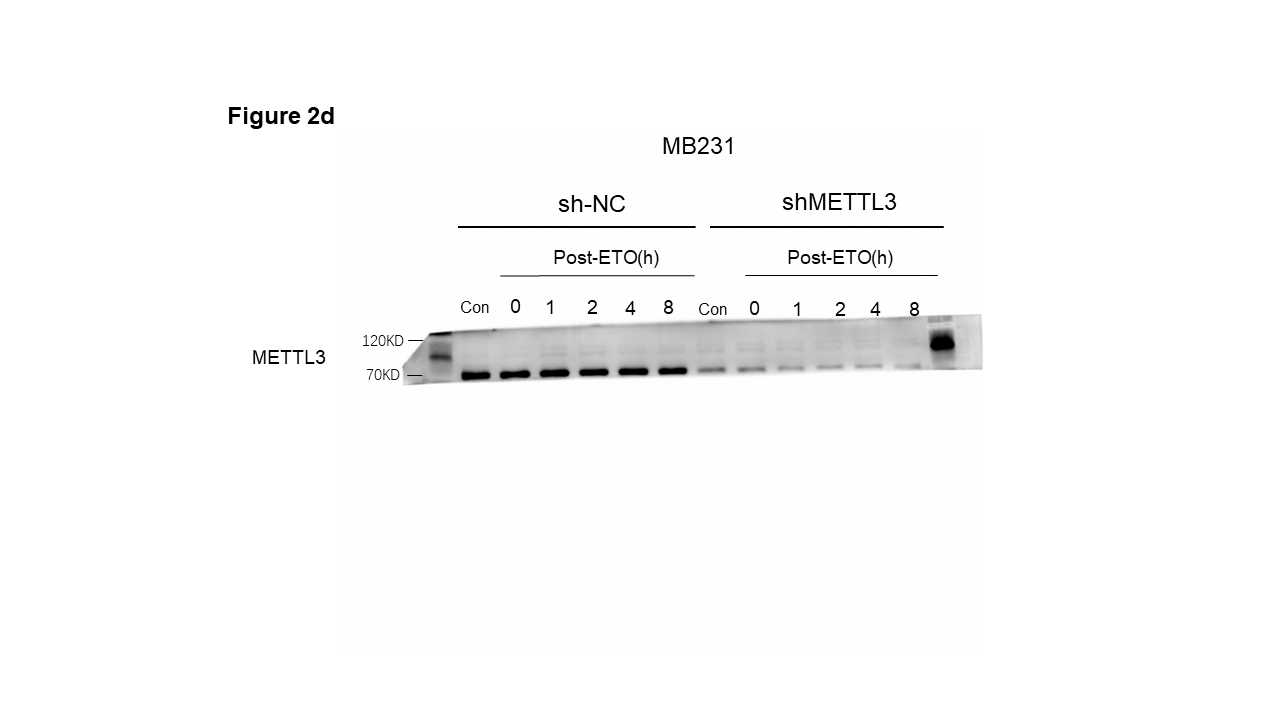

Supplement: Figure 2—source data 4. [file elife-75231-fig2-data4.zip › Figure 2D/Figure 2D METTL3.TIF]

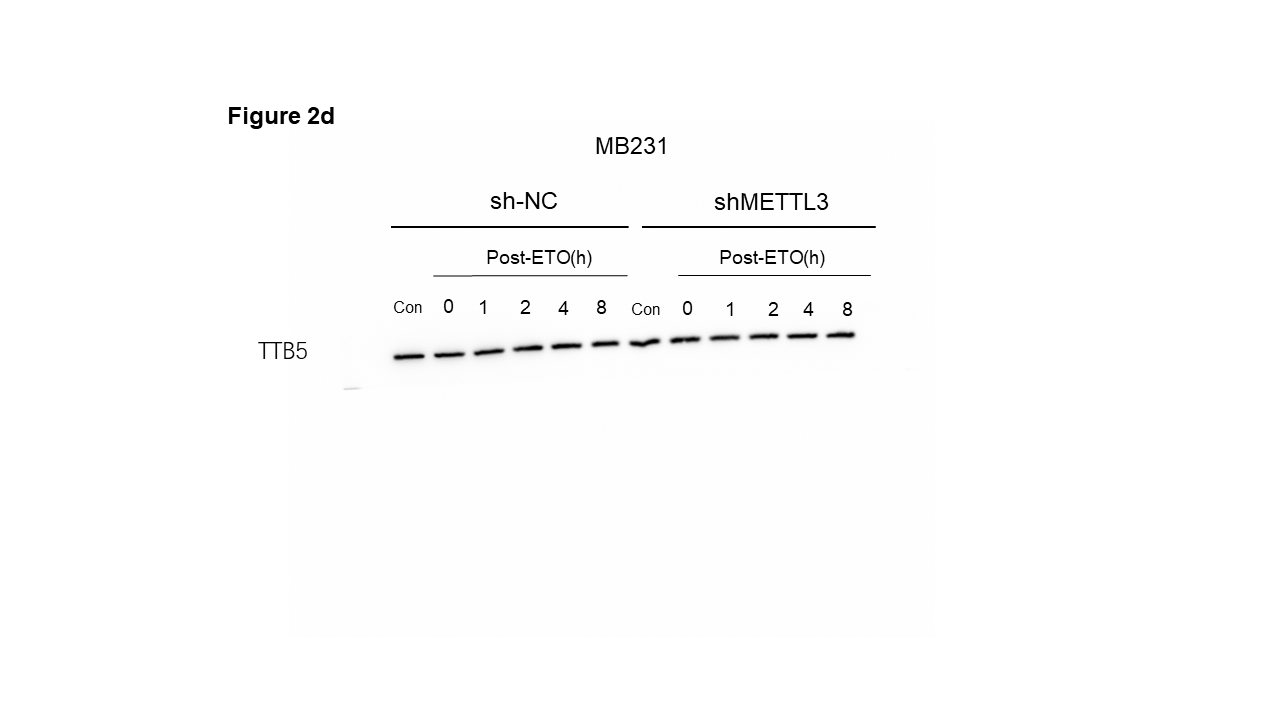

Supplement: Figure 2—source data 4. [file elife-75231-fig2-data4.zip › Figure 2D/Figure 2D TBB5.TIF]

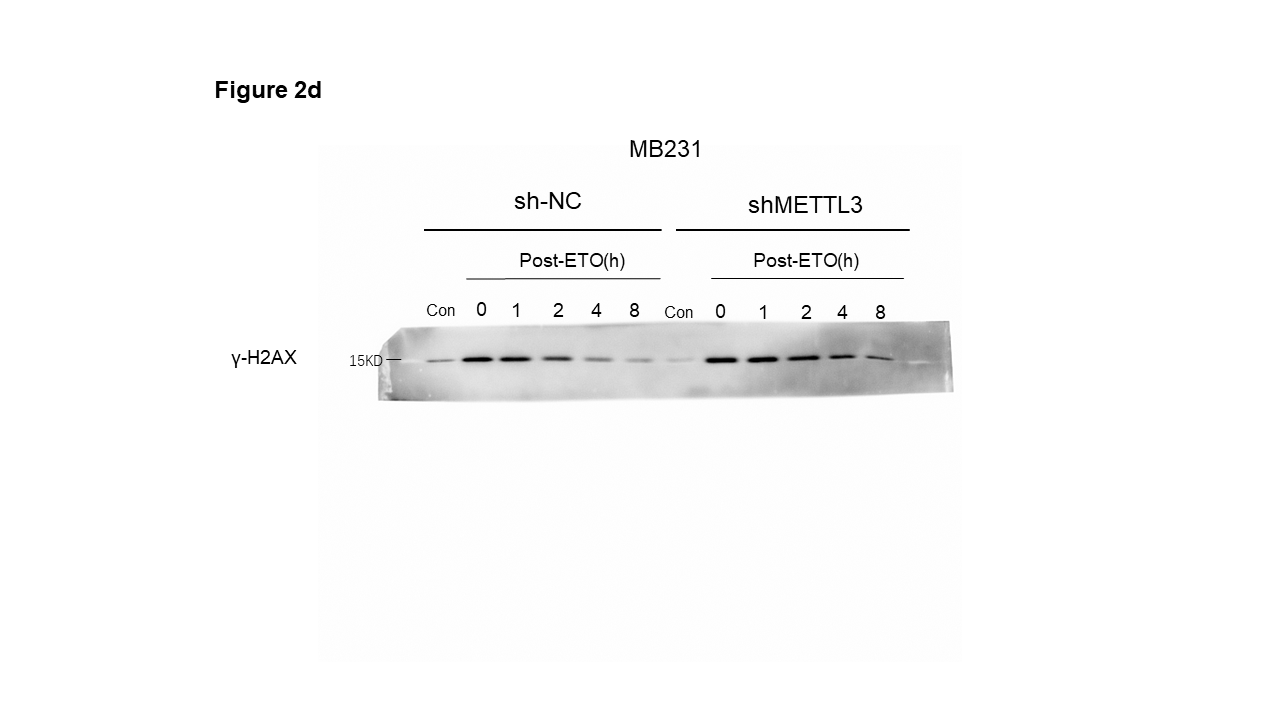

Supplement: Figure 2—source data 4. [file elife-75231-fig2-data4.zip › Figure 2D/Figure 2D a├-H2AX.TIF]

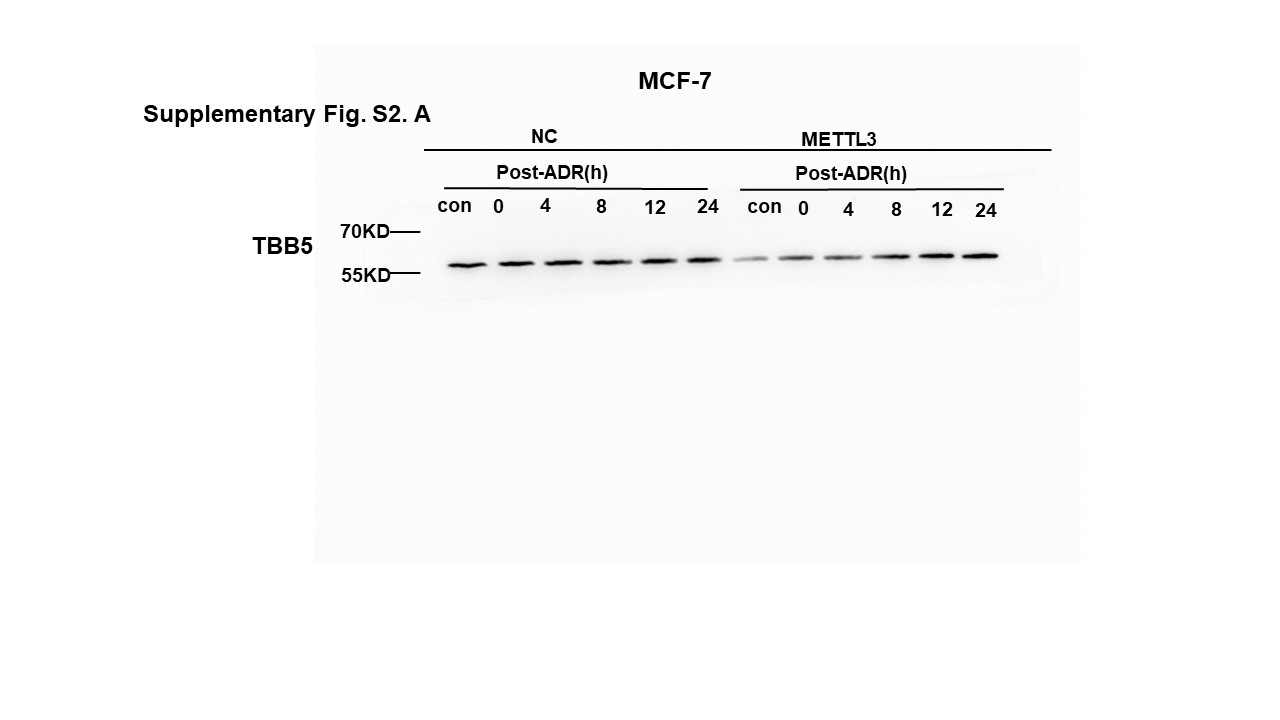

Supplement: Figure 2—figure supplement 1—source data 1. [file elife-75231-fig2-figsupp1-data1.zip › Figure S2A/Supplementary Fig.S2. A TBB5.jpg.TIF]

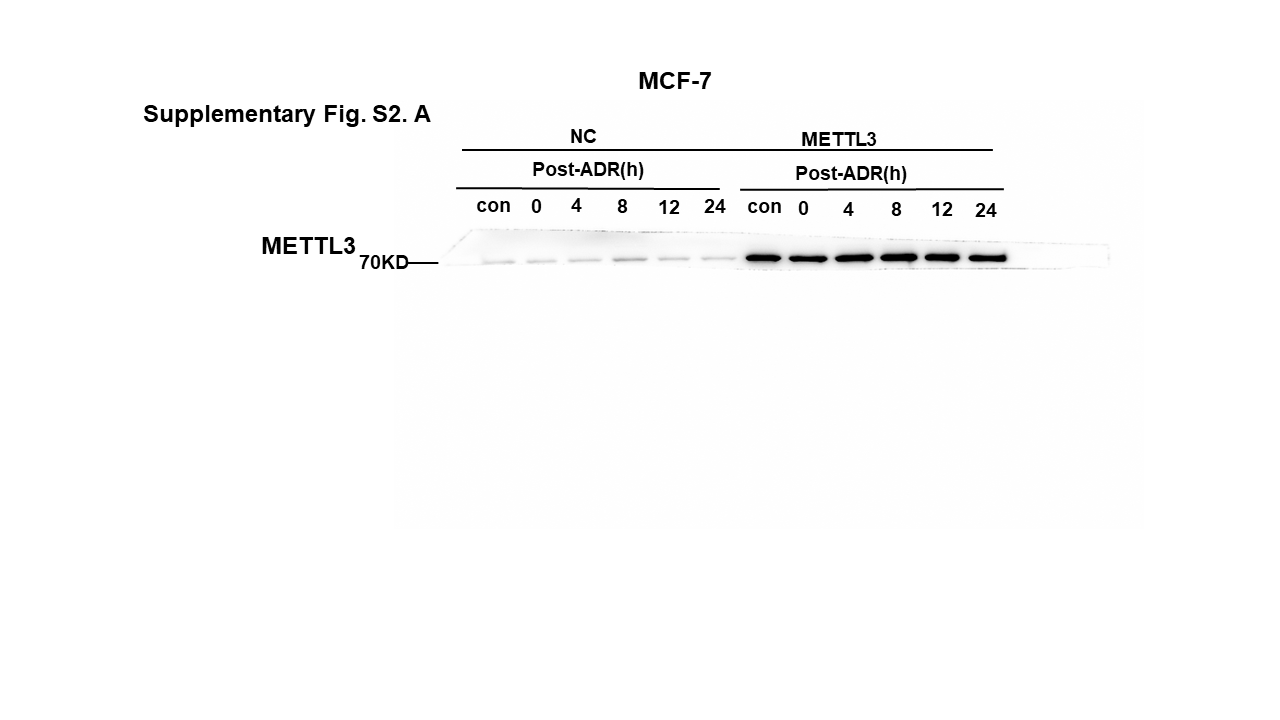

Supplement: Figure 2—figure supplement 1—source data 1. [file elife-75231-fig2-figsupp1-data1.zip › Figure S2A/Supplementary Fig.S2. A METTL3.jpg.TIF]

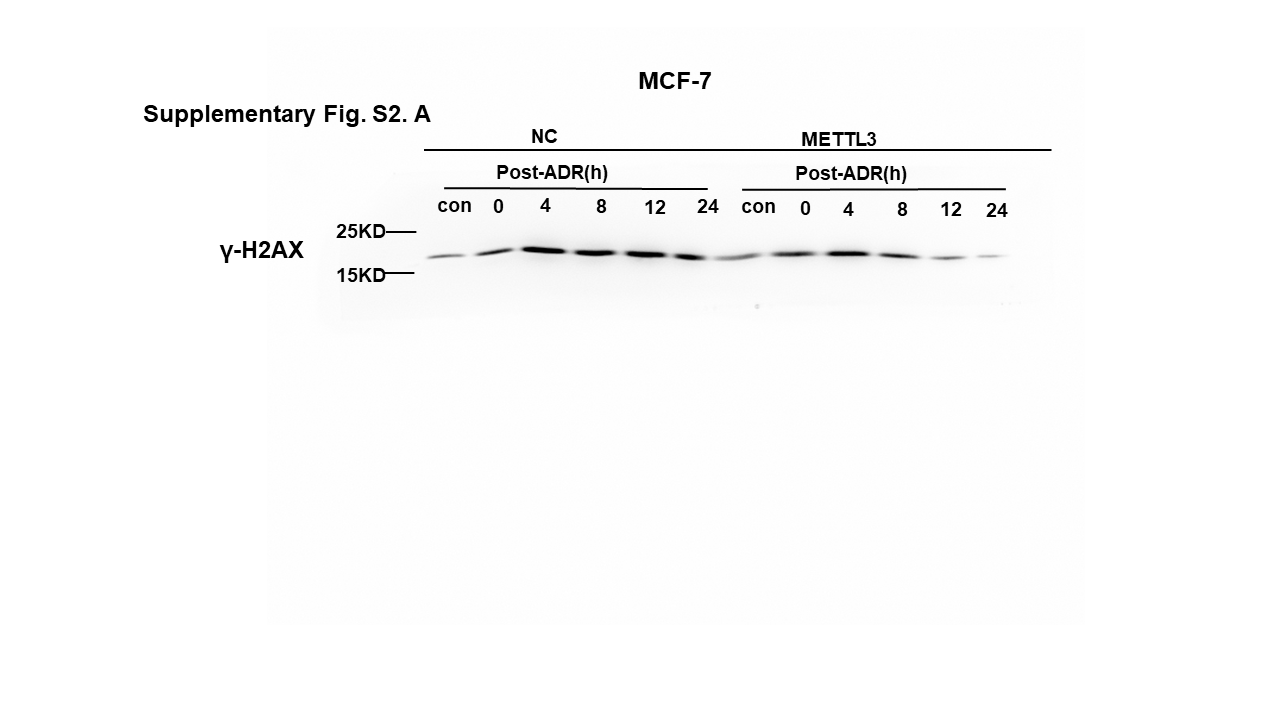

Supplement: Figure 2—figure supplement 1—source data 1. [file elife-75231-fig2-figsupp1-data1.zip › Figure S2A/Supplementary Fig.S2. A γ-H2AX.jpg.TIF]

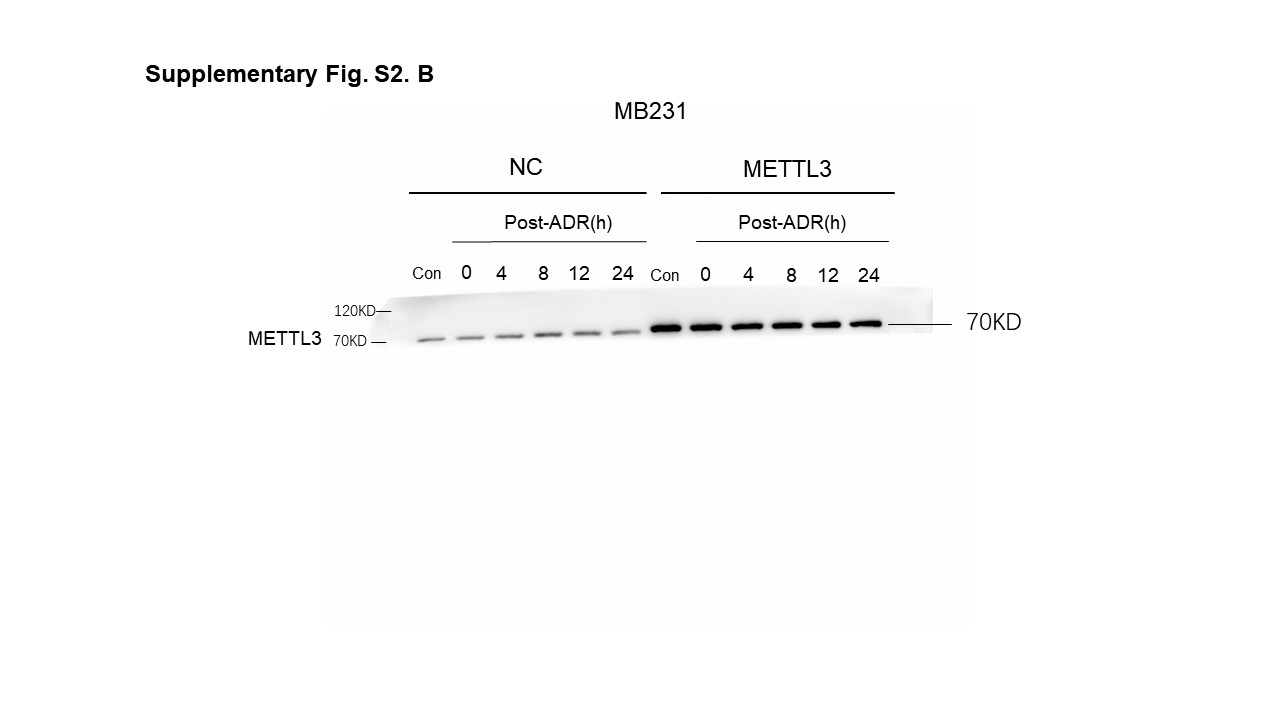

Supplement: Figure 2—figure supplement 1—source data 2. [file elife-75231-fig2-figsupp1-data2.zip › Figure S2B/Supplementary Fig. S2. B mettl3.JPG]

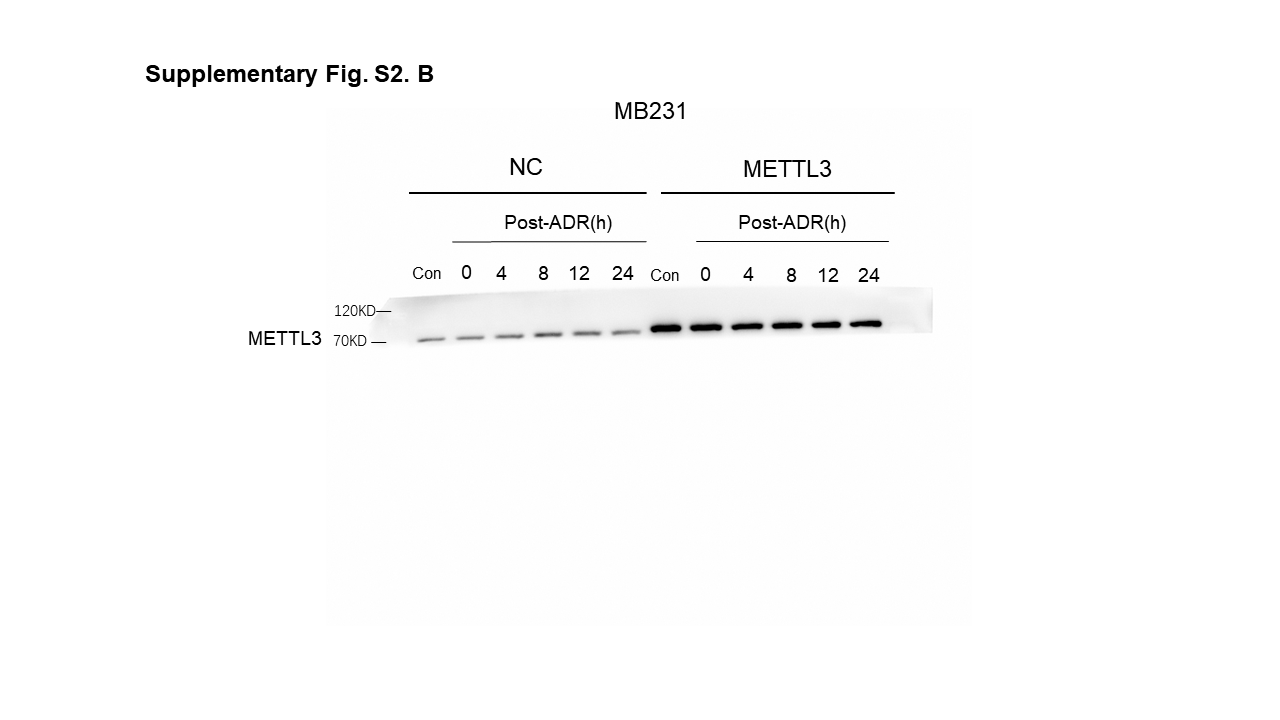

Supplement: Figure 2—figure supplement 1—source data 2. [file elife-75231-fig2-figsupp1-data2.zip › Figure S2B/Supplementary Fig. S2. B mettl3.TIF]

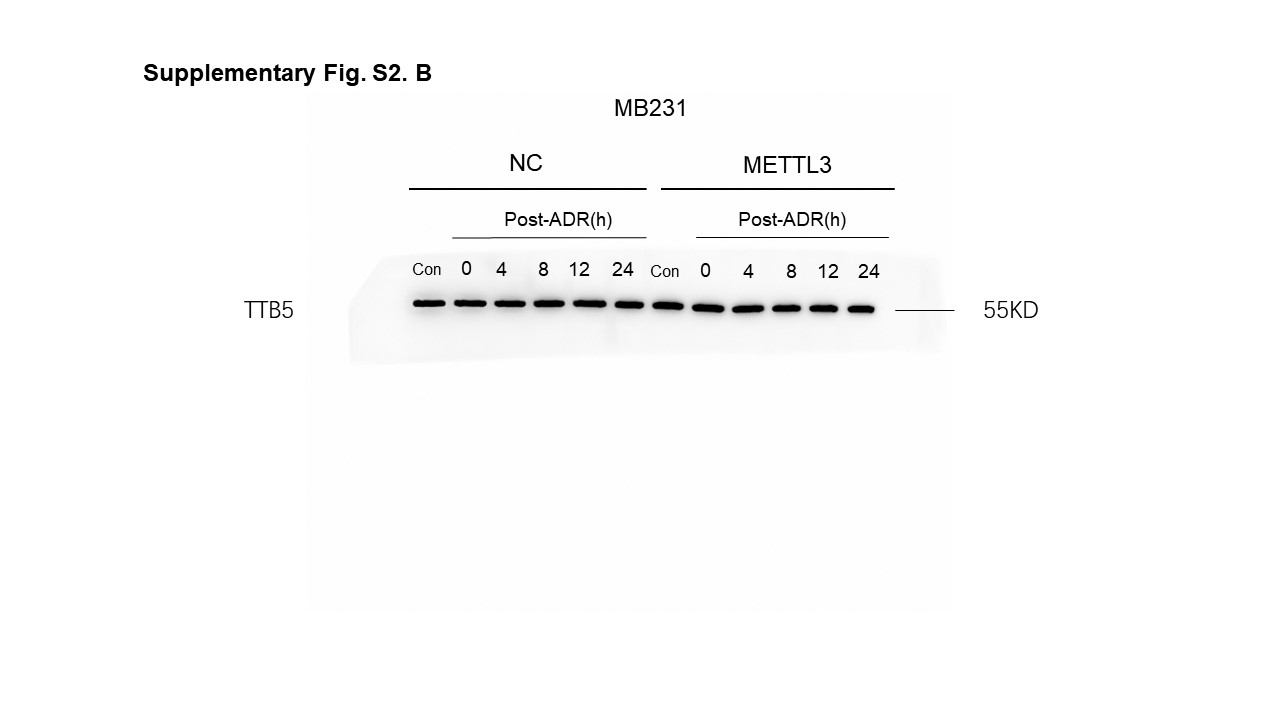

Supplement: Figure 2—figure supplement 1—source data 2. [file elife-75231-fig2-figsupp1-data2.zip › Figure S2B/Supplementary Fig. S2. B tbb5.JPG]

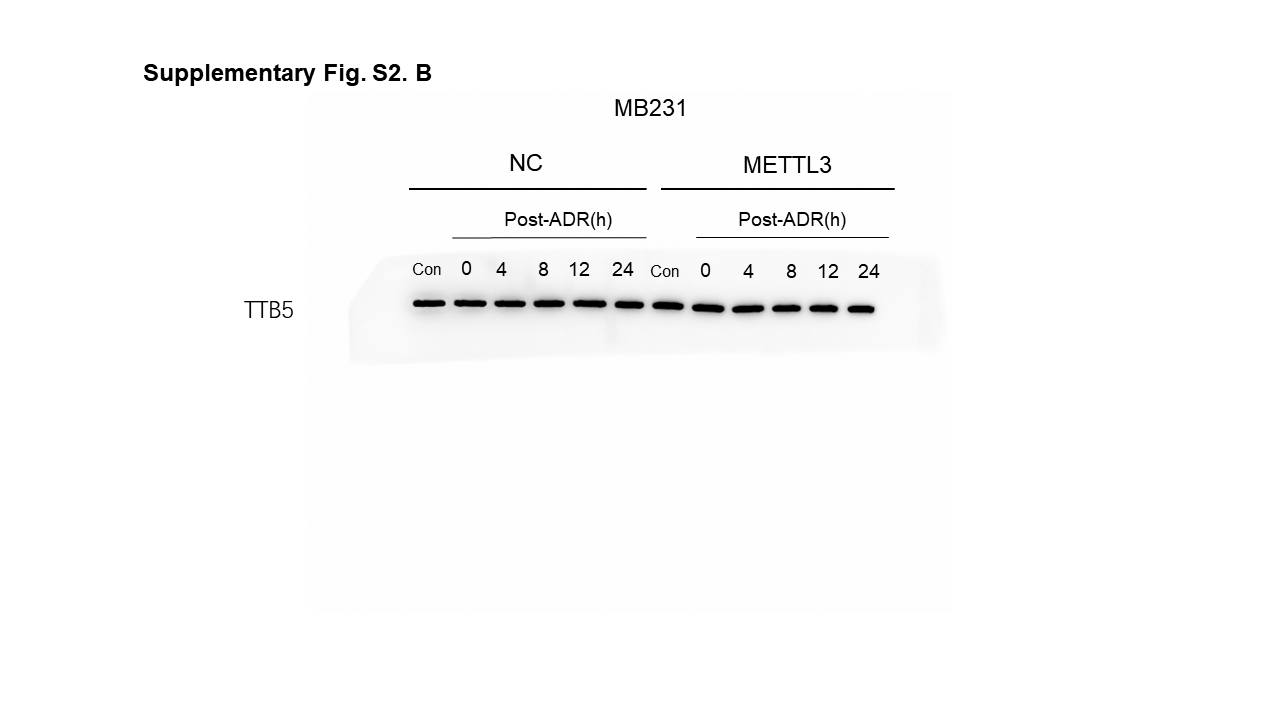

Supplement: Figure 2—figure supplement 1—source data 2. [file elife-75231-fig2-figsupp1-data2.zip › Figure S2B/Supplementary Fig. S2. B tbb5.TIF]

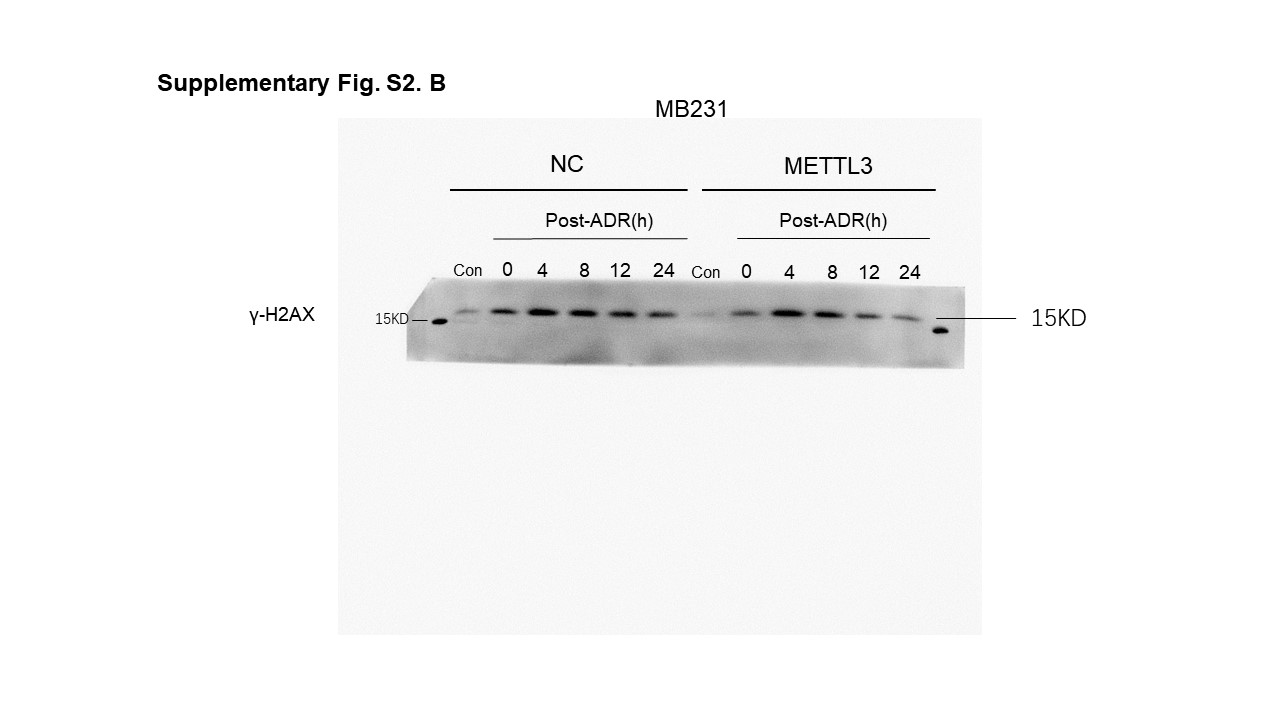

Supplement: Figure 2—figure supplement 1—source data 2. [file elife-75231-fig2-figsupp1-data2.zip › Figure S2B/Supplementary Fig. S2. B γh2ax.JPG]

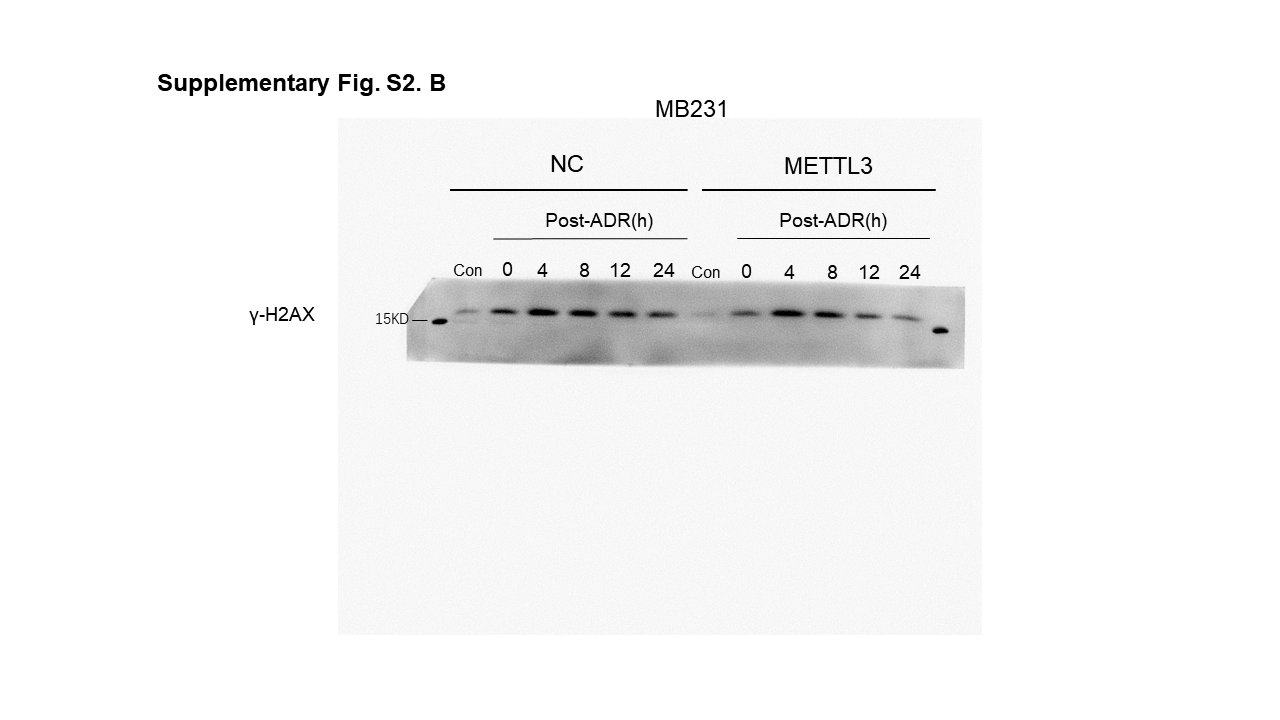

Supplement: Figure 2—figure supplement 1—source data 2. [file elife-75231-fig2-figsupp1-data2.zip › Figure S2B/Supplementary Fig. S2. B γh2ax.TIF]

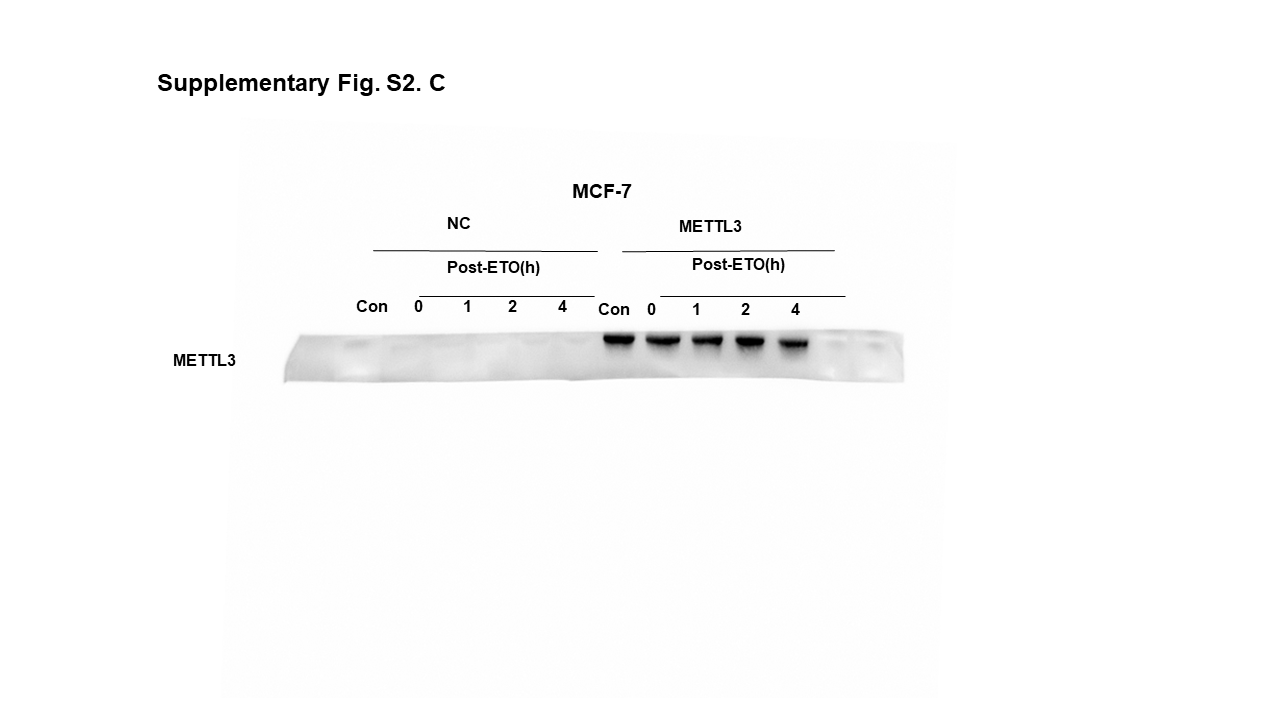

Supplement: Figure 2—figure supplement 1—source data 3. [file elife-75231-fig2-figsupp1-data3.zip › Figure S2C/Figure S2C METTL3.TIF]

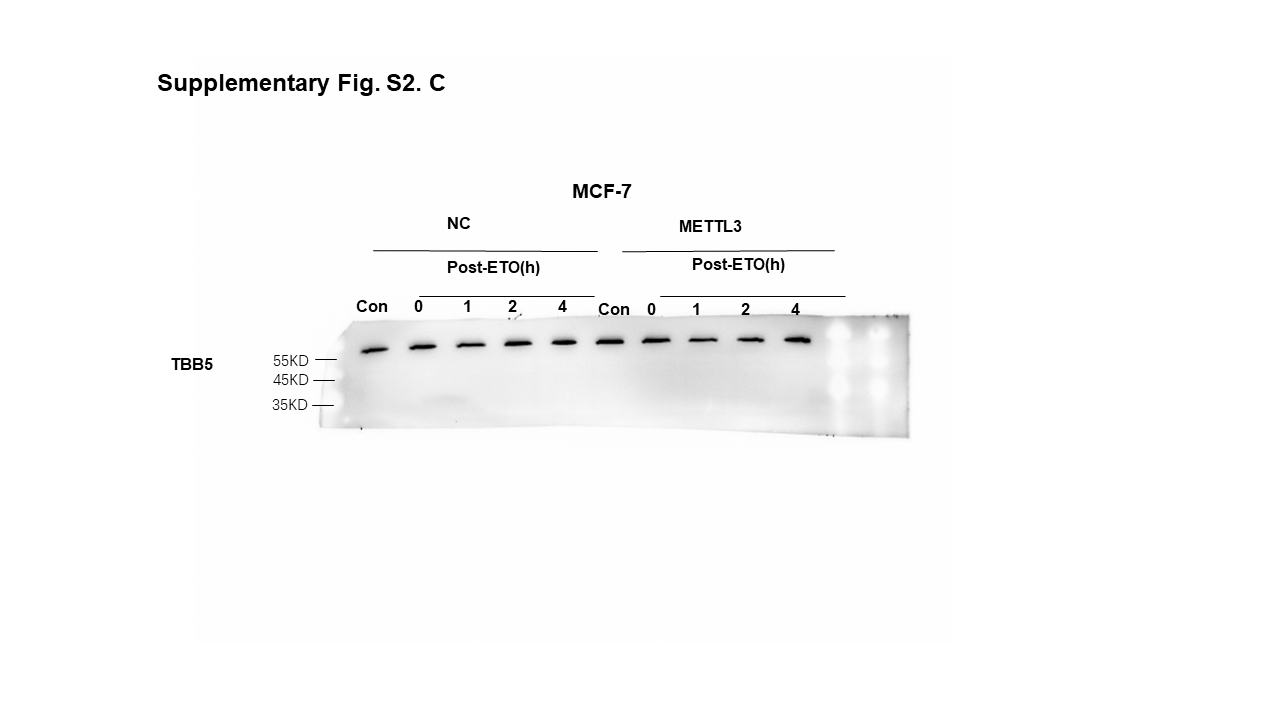

Supplement: Figure 2—figure supplement 1—source data 3. [file elife-75231-fig2-figsupp1-data3.zip › Figure S2C/Figure S2C TBB5.TIF]

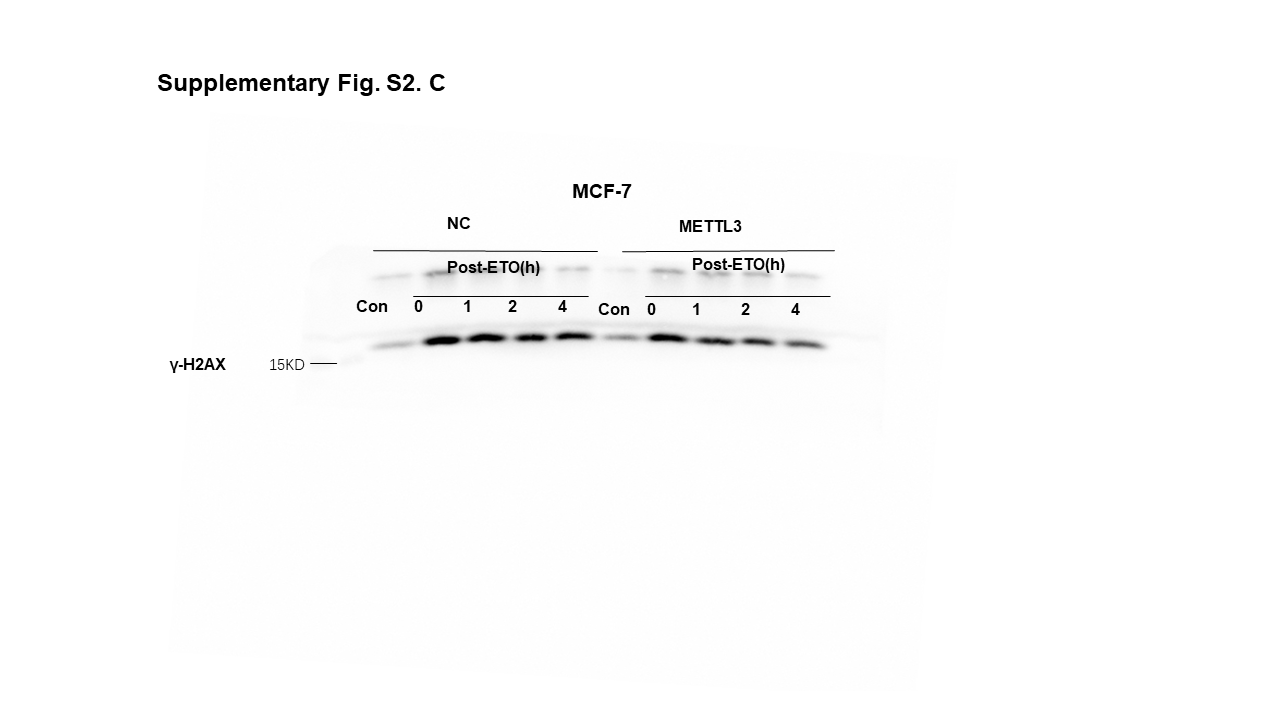

Supplement: Figure 2—figure supplement 1—source data 3. [file elife-75231-fig2-figsupp1-data3.zip › Figure S2C/Figure S2C a├-H2AX.TIF]

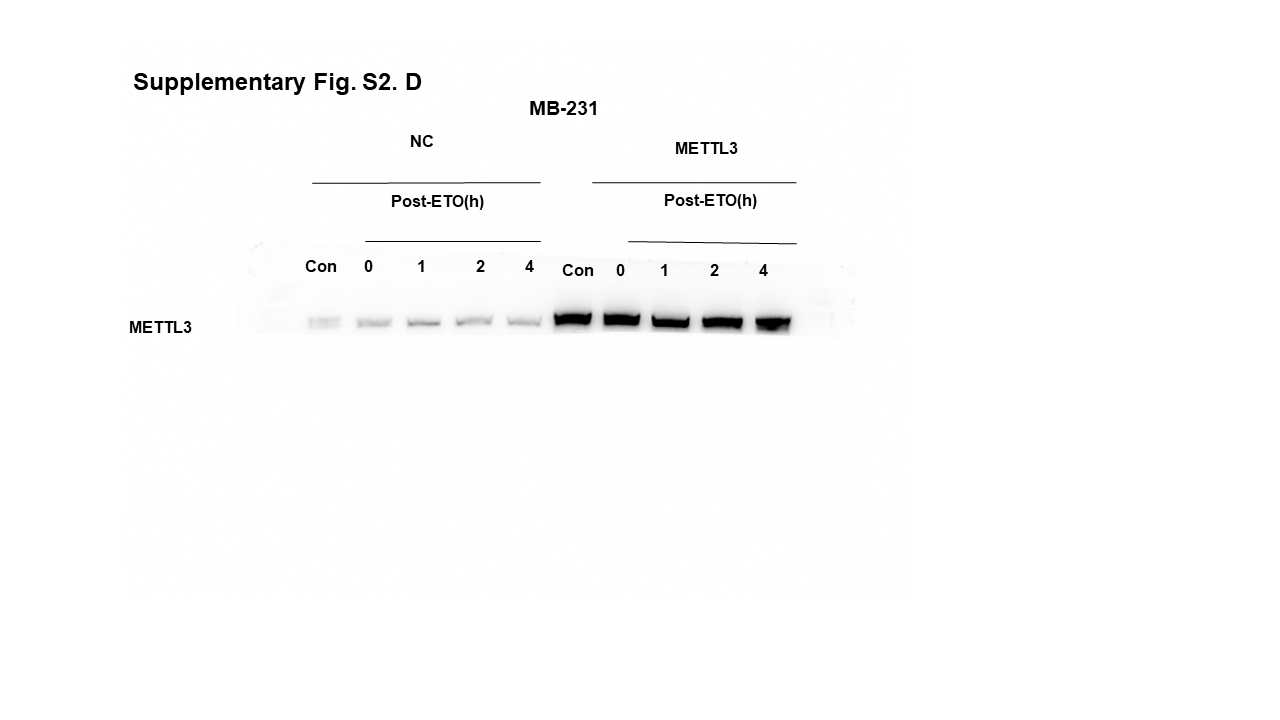

Supplement: Figure 2—figure supplement 1—source data 4. [file elife-75231-fig2-figsupp1-data4.zip › Figure S2D/Figure S2D METTL3.TIF]

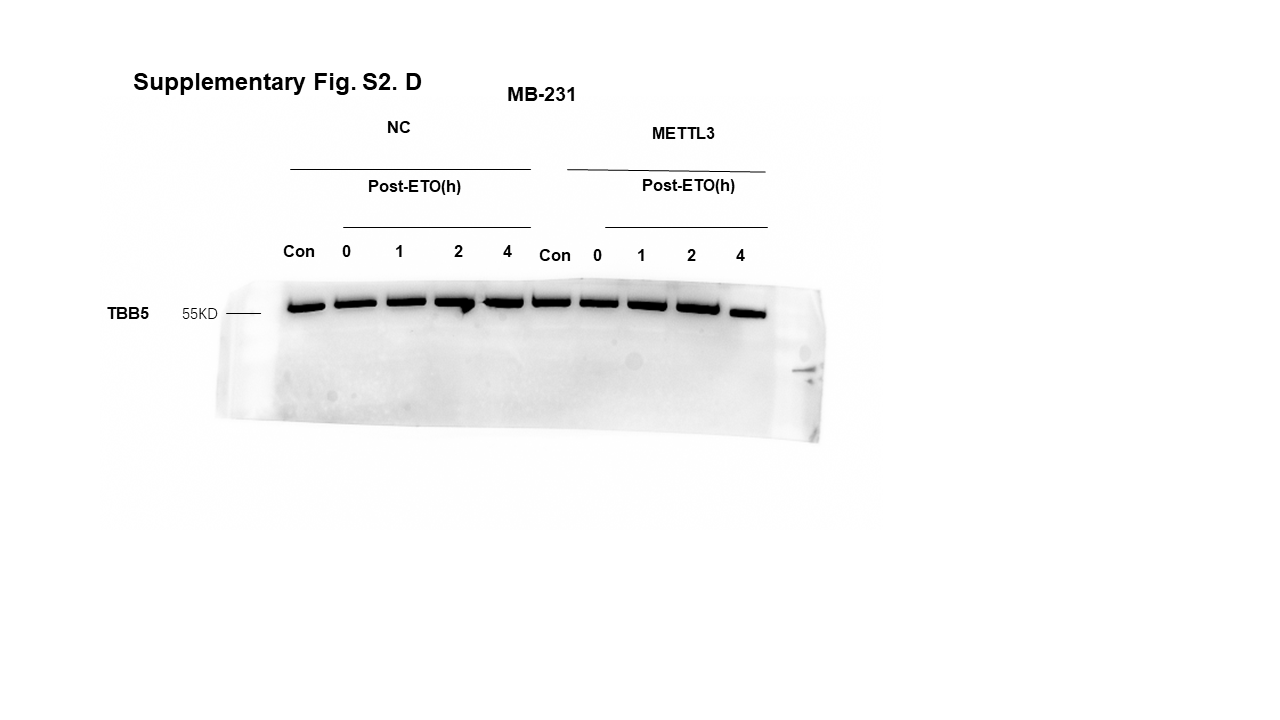

Supplement: Figure 2—figure supplement 1—source data 4. [file elife-75231-fig2-figsupp1-data4.zip › Figure S2D/Figure S2D TBB5.TIF]

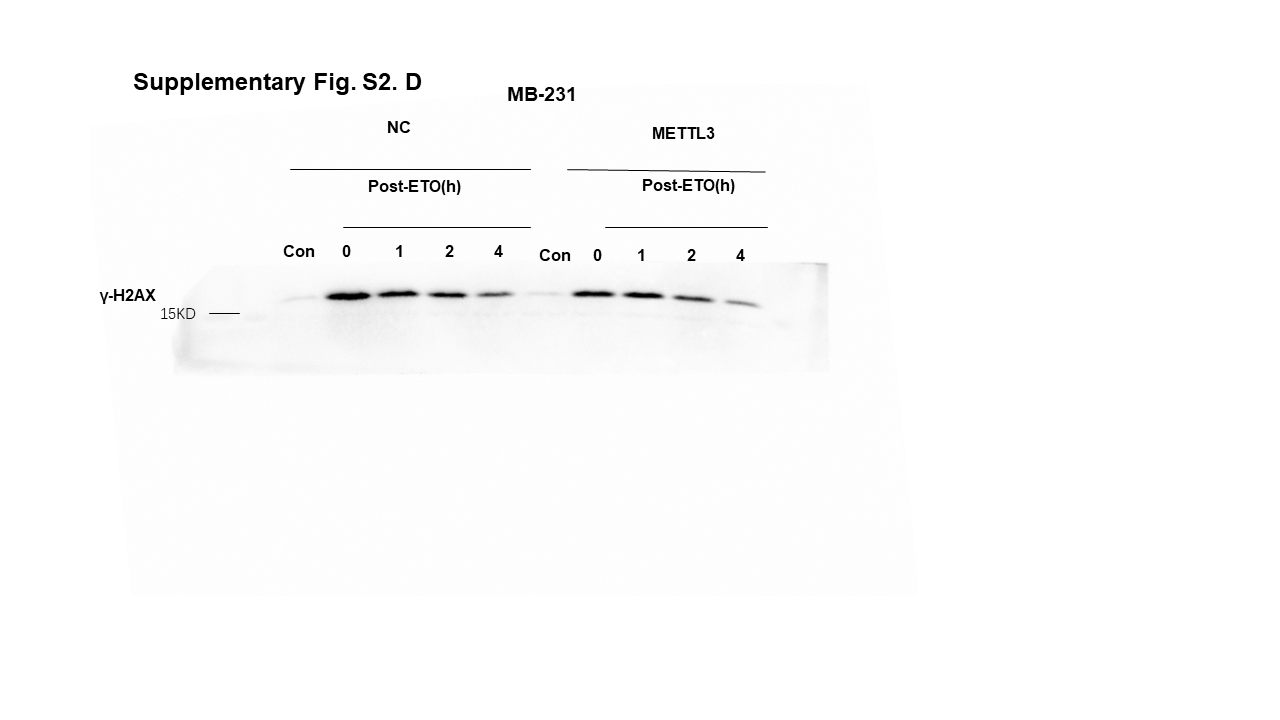

Supplement: Figure 2—figure supplement 1—source data 4. [file elife-75231-fig2-figsupp1-data4.zip › Figure S2D/Figure S2D a├-H2AX.tif]

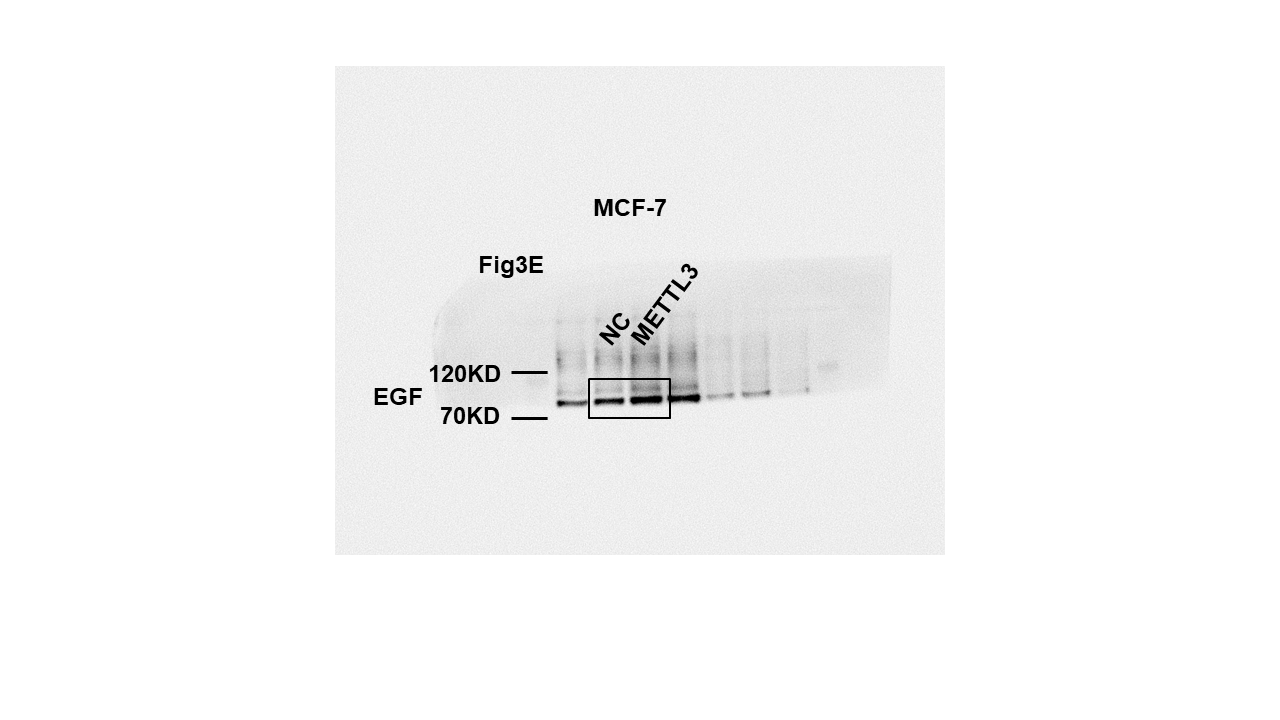

Supplement: Figure 3—source data 1. [file elife-75231-fig3-data1.zip › Figure 3e/Figure 3e EGF.TIF]

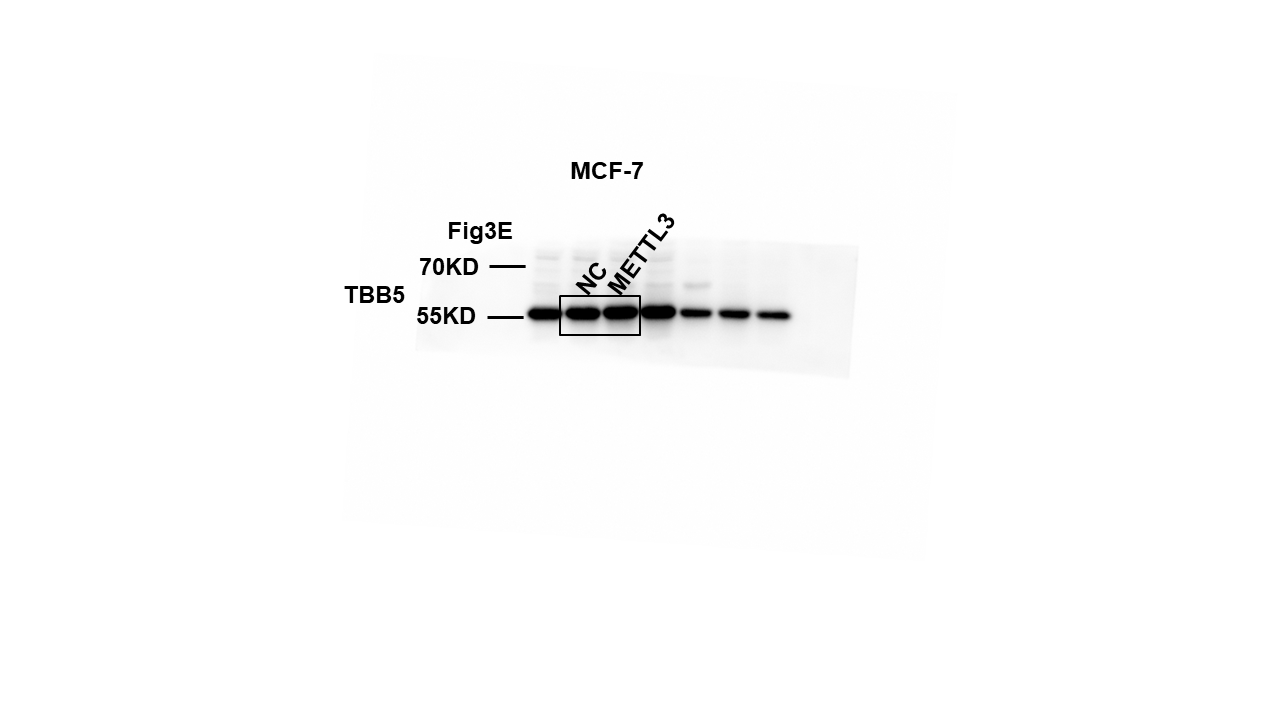

Supplement: Figure 3—source data 1. [file elife-75231-fig3-data1.zip › Figure 3e/Figure 3e TBB5.TIF]

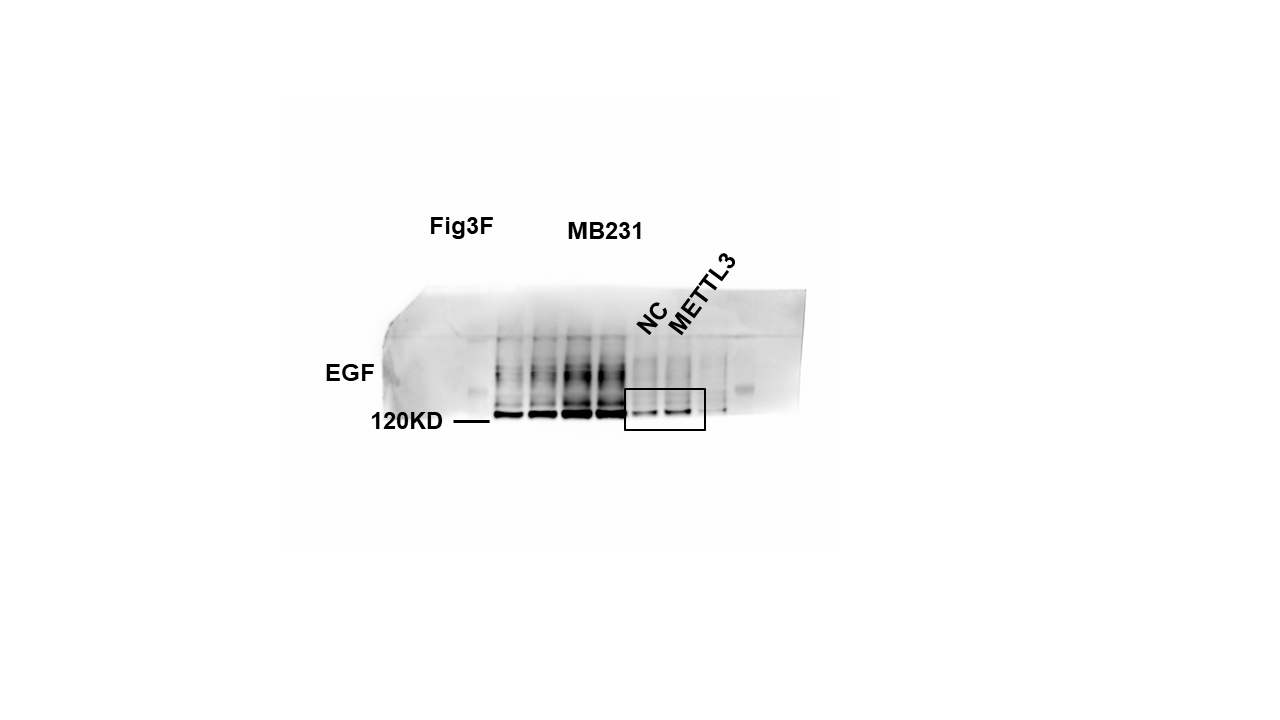

Supplement: Figure 3—source data 2. [file elife-75231-fig3-data2.zip › Figure 3f/Figure 3F EGF.TIF]

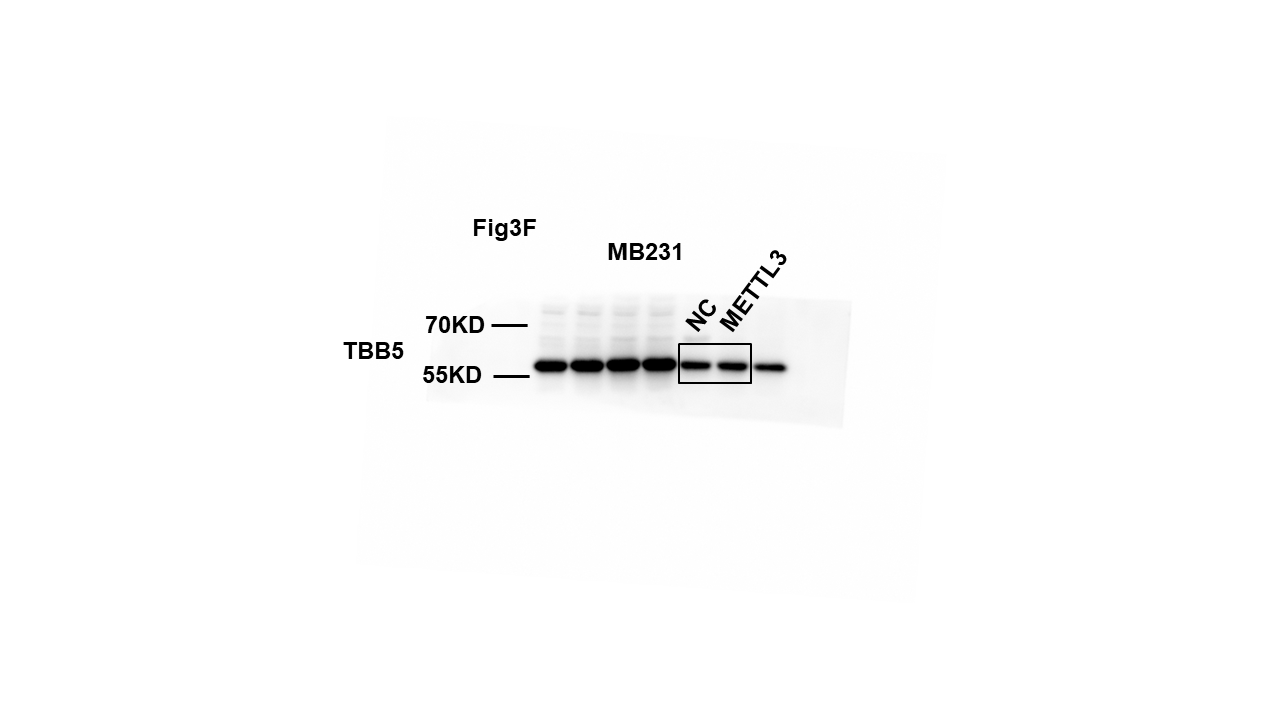

Supplement: Figure 3—source data 2. [file elife-75231-fig3-data2.zip › Figure 3f/Figure 3F TBB5.TIF]

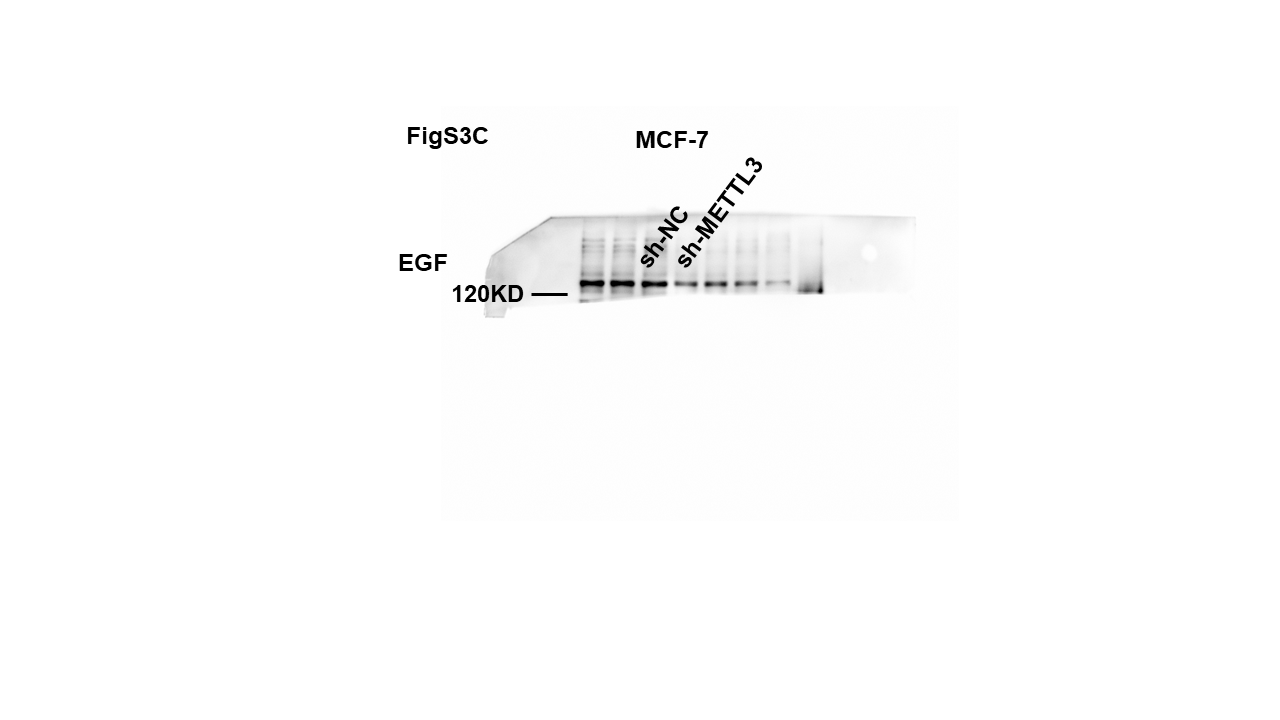

Supplement: Figure 3—figure supplement 1—source data 1. [file elife-75231-fig3-figsupp1-data1.zip › Figure S3C/Figure S3C EGF.TIF]

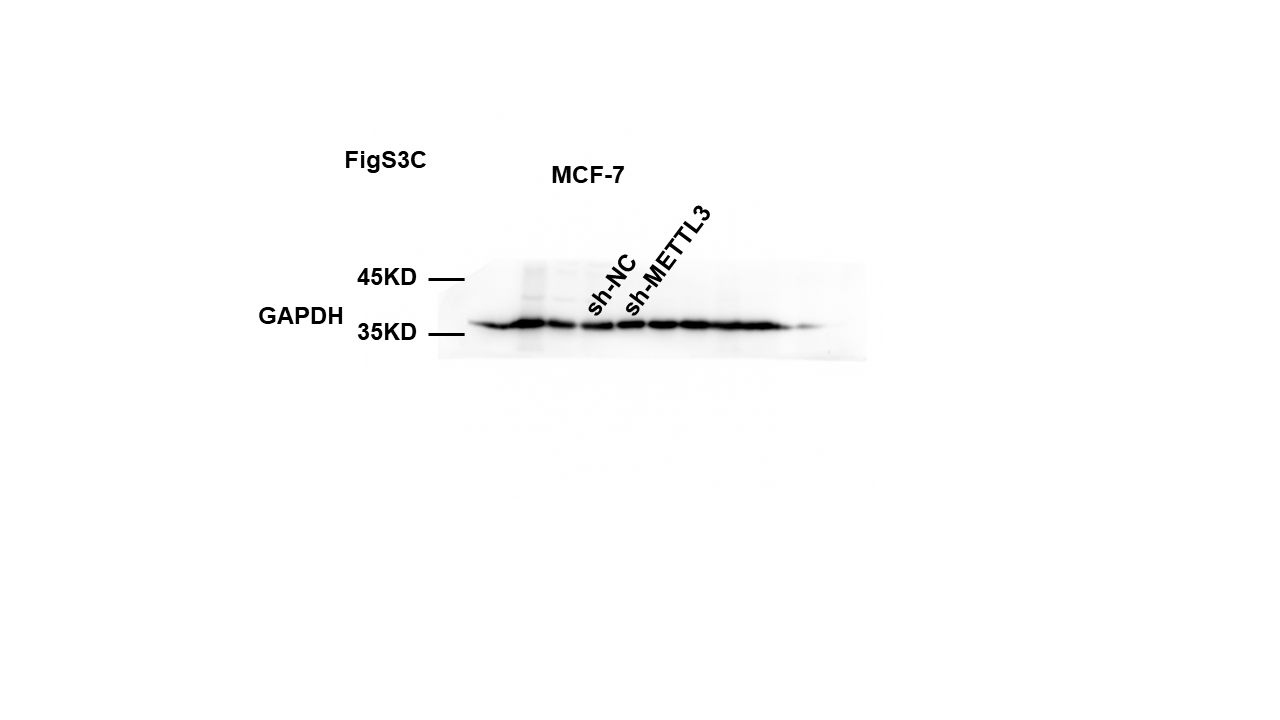

Supplement: Figure 3—figure supplement 1—source data 1. [file elife-75231-fig3-figsupp1-data1.zip › Figure S3C/Figure S3C GAPDH.TIF]

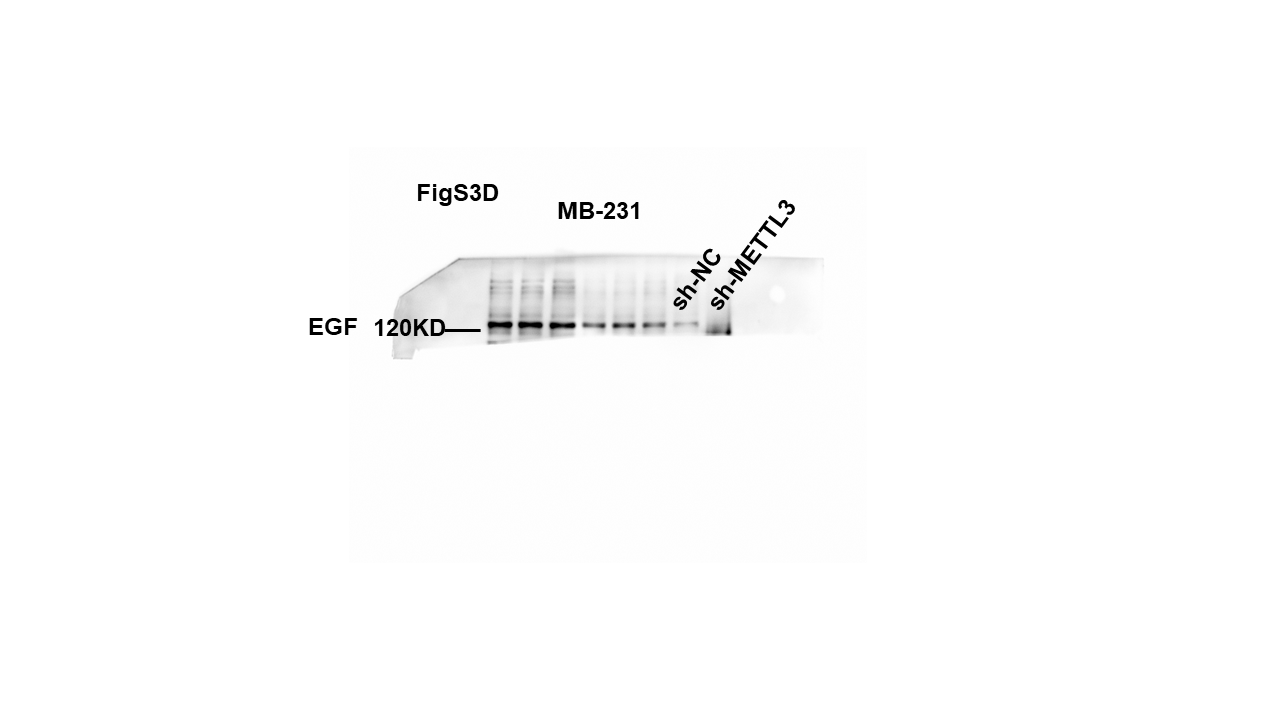

Supplement: Figure 3—figure supplement 1—source data 2. [file elife-75231-fig3-figsupp1-data2.zip › Figure S3D/Figure S3D EGF.TIF]

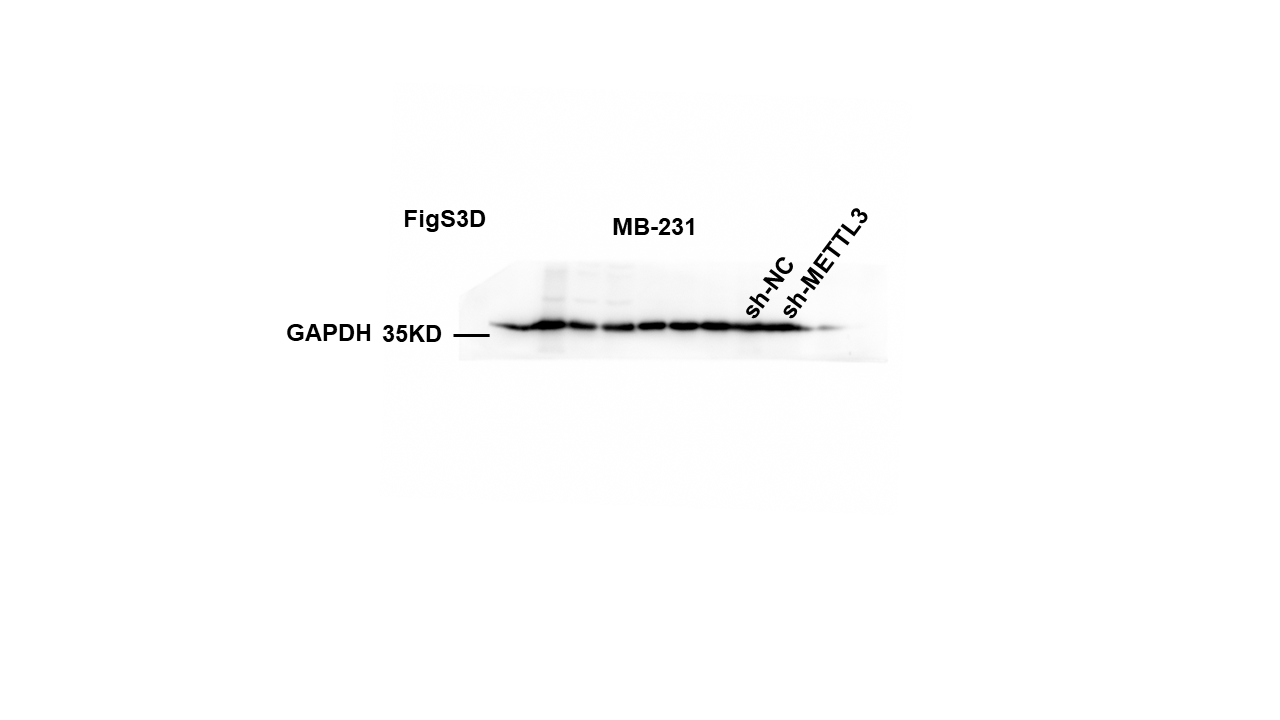

Supplement: Figure 3—figure supplement 1—source data 2. [file elife-75231-fig3-figsupp1-data2.zip › Figure S3D/Figure S3D GAPDH.TIF]

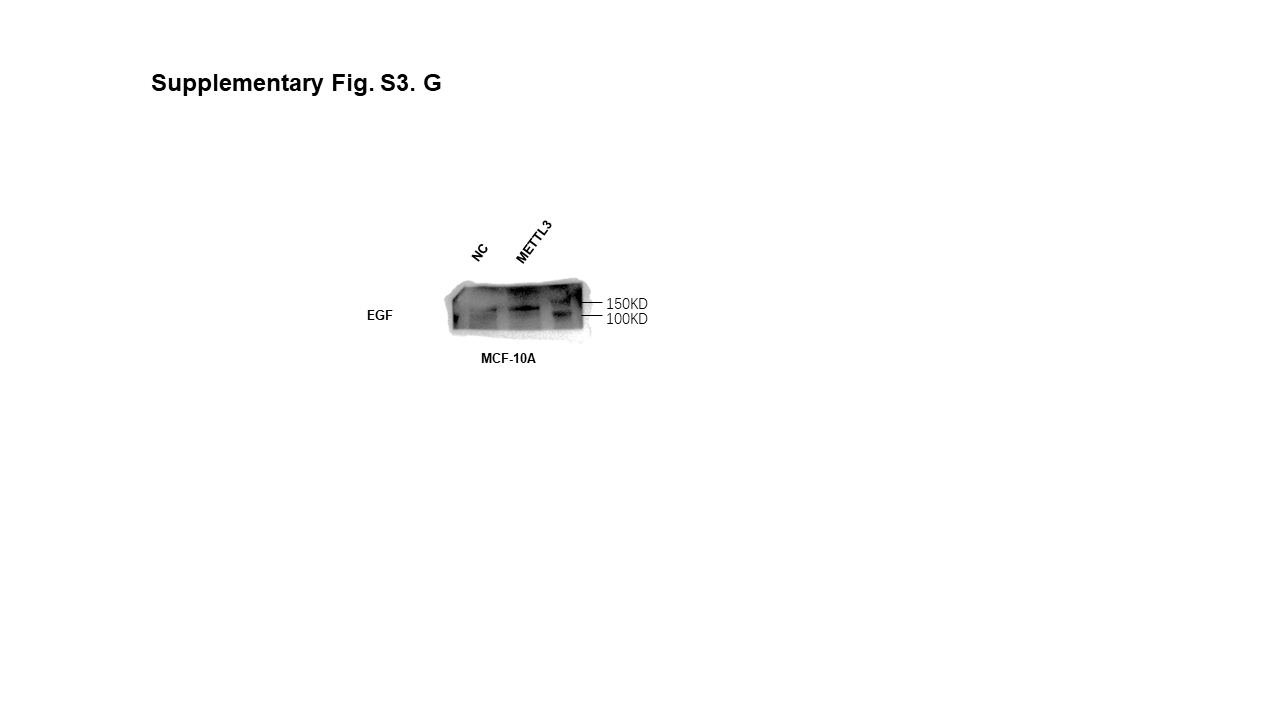

Supplement: Figure 3—figure supplement 1—source data 3. [file elife-75231-fig3-figsupp1-data3.zip › Figure S3G/Figure S3G EGF.TIF]

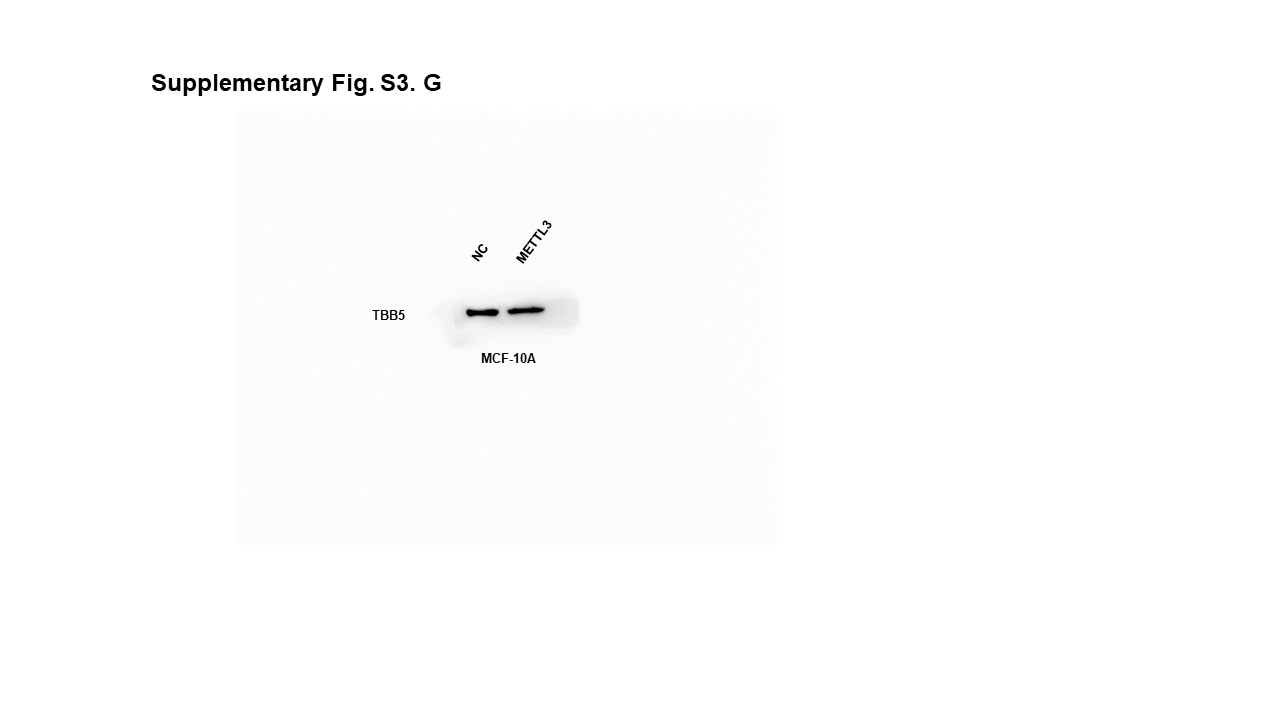

Supplement: Figure 3—figure supplement 1—source data 3. [file elife-75231-fig3-figsupp1-data3.zip › Figure S3G/Figure S3G TBB5.TIF]

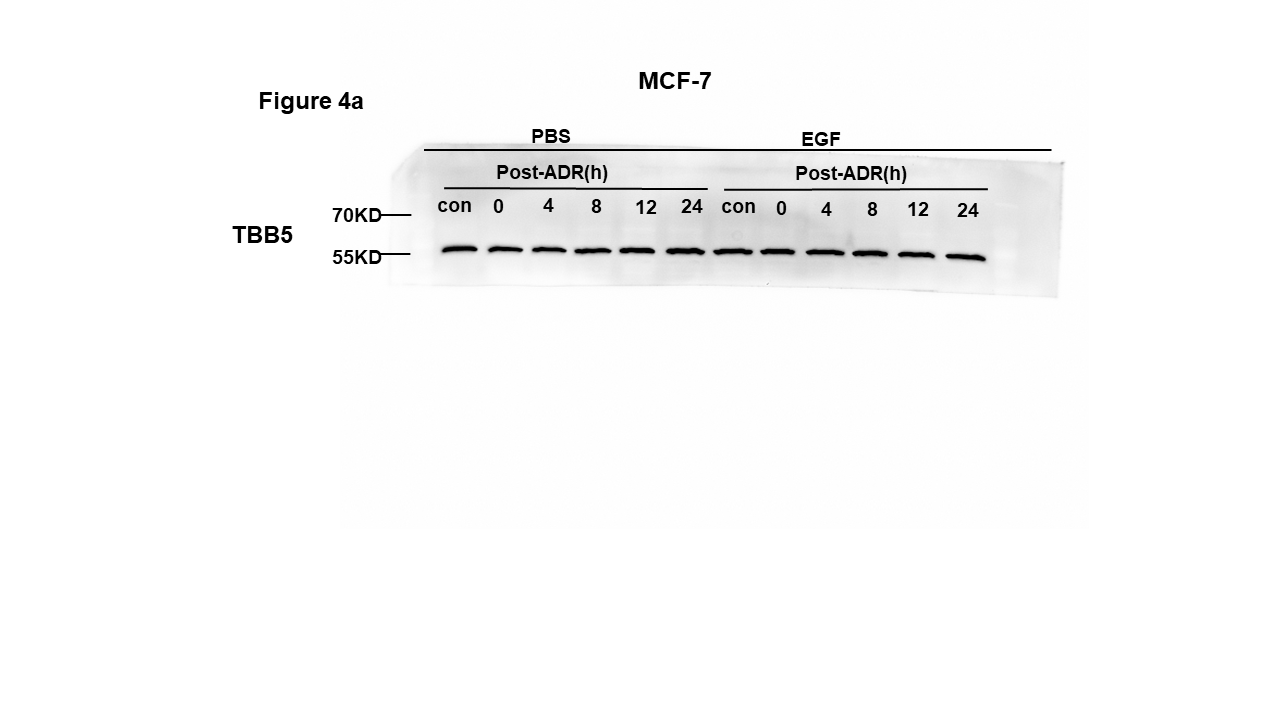

Supplement: Figure 4—source data 1. [file elife-75231-fig4-data1.zip › Figure 4a/Figure 4a TBB5.TIF]

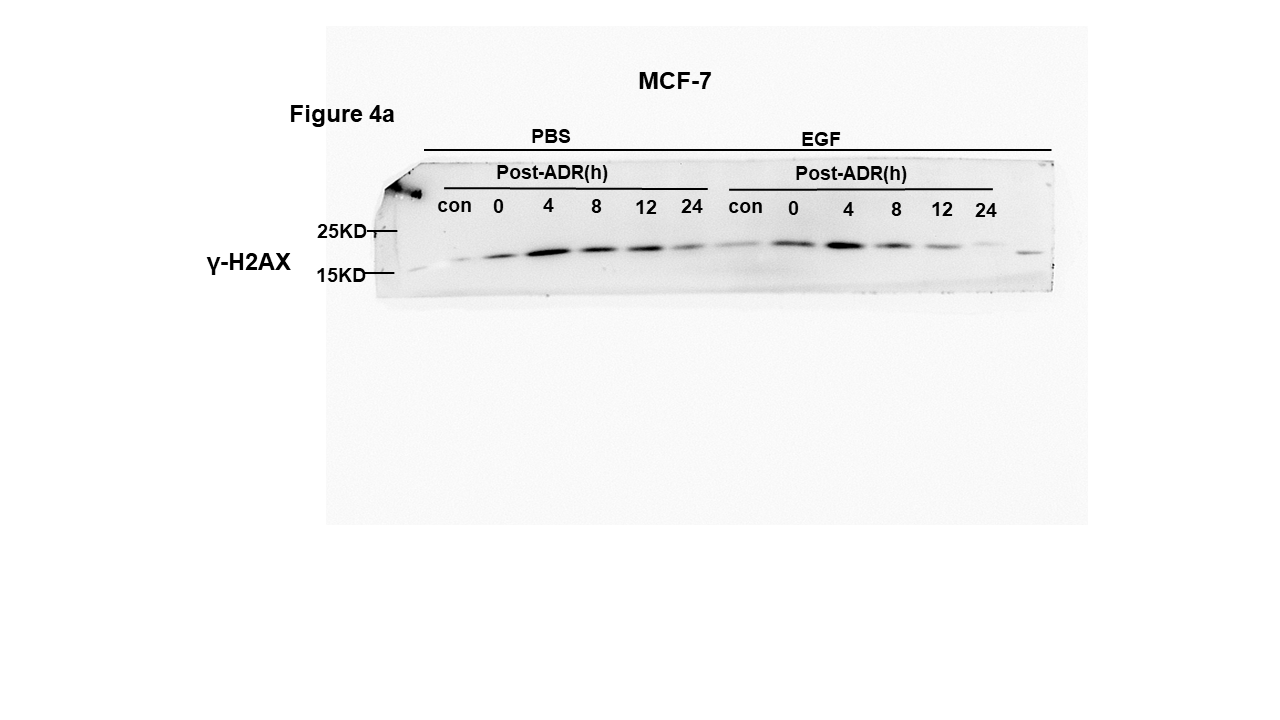

Supplement: Figure 4—source data 1. [file elife-75231-fig4-data1.zip › Figure 4a/Figure 4a γ-H2AX.TIF]

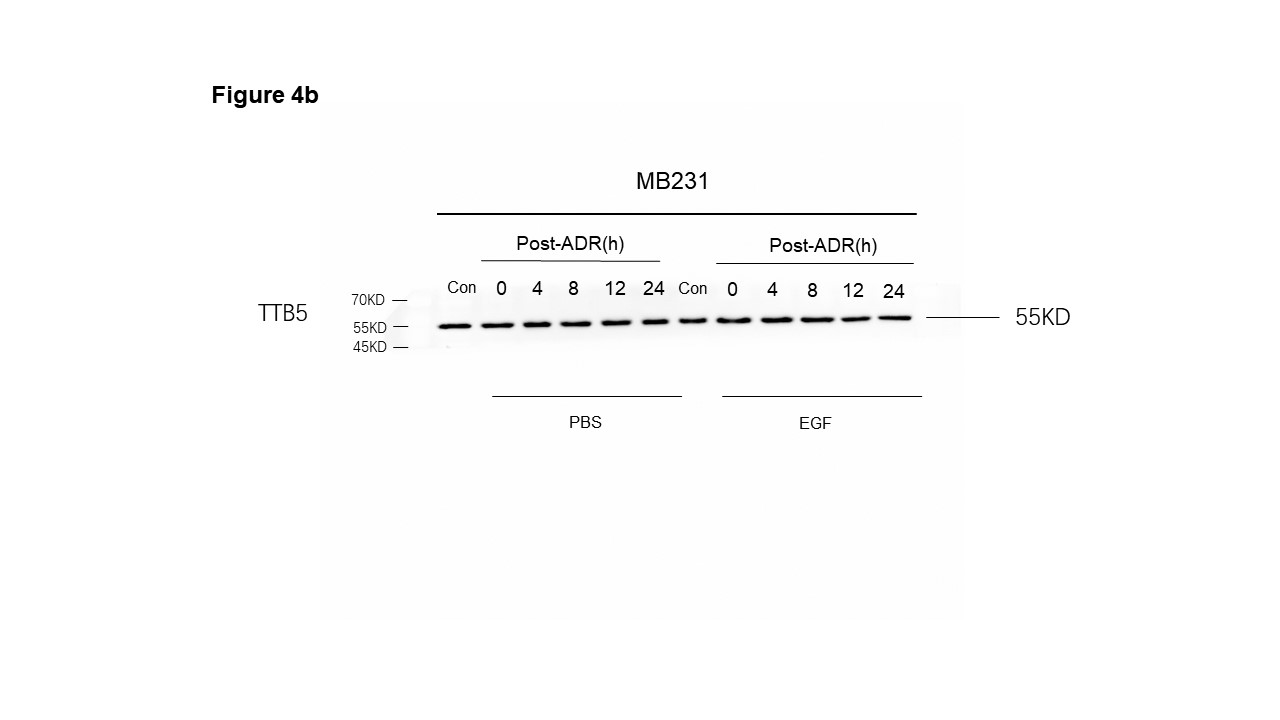

Supplement: Figure 4—source data 2. [file elife-75231-fig4-data2.zip › Figure 4b/Figure 4b tbb5.JPG]

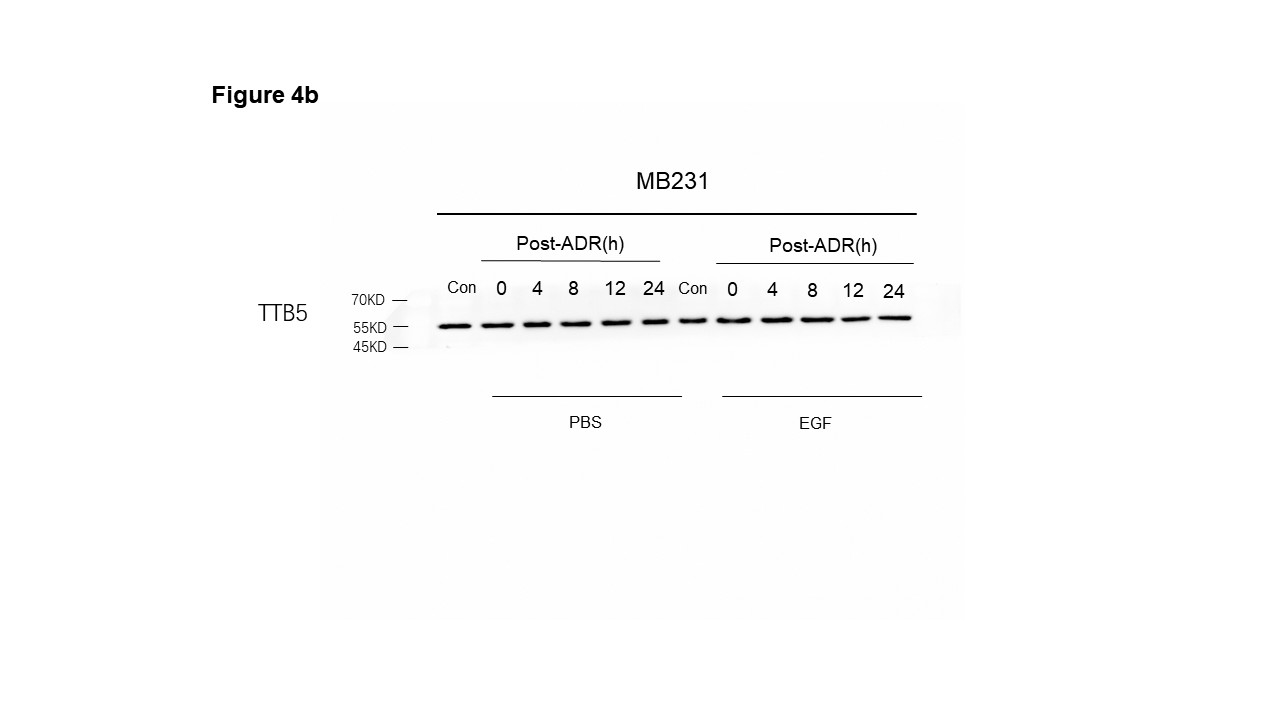

Supplement: Figure 4—source data 2. [file elife-75231-fig4-data2.zip › Figure 4b/Figure 4b tbb5.TIF]

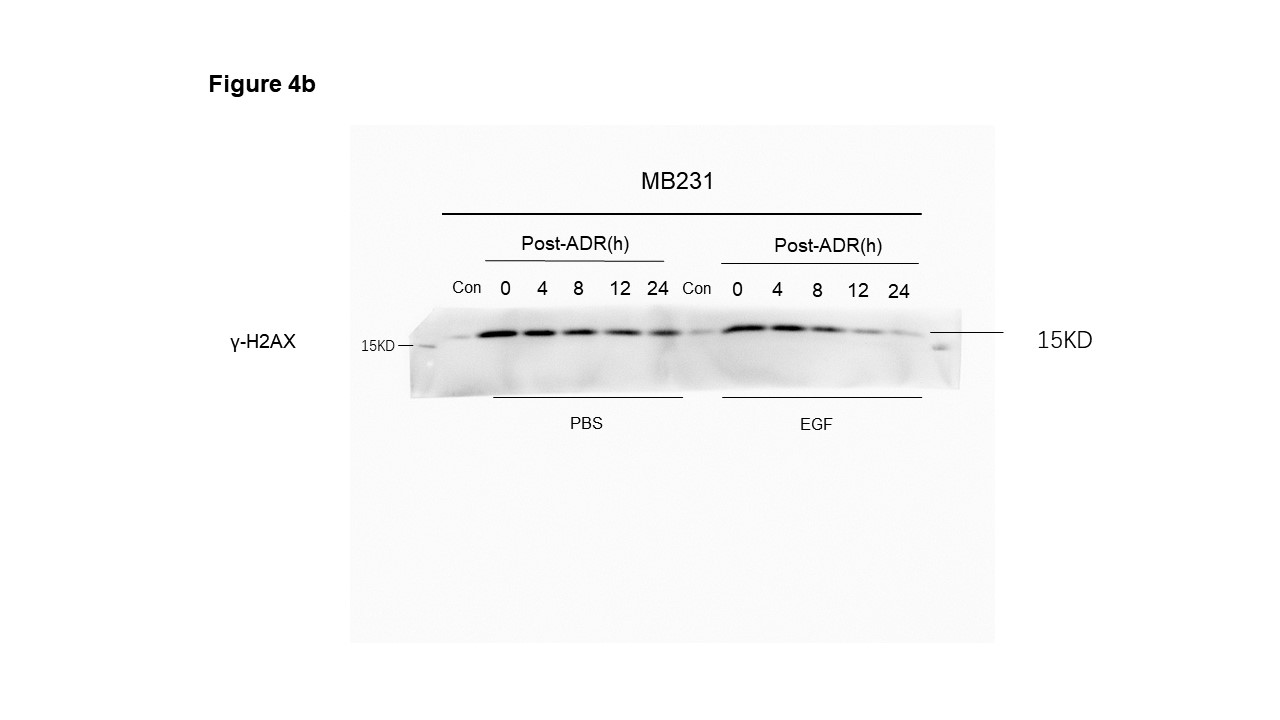

Supplement: Figure 4—source data 2. [file elife-75231-fig4-data2.zip › Figure 4b/Figure 4b γh2ax.JPG]

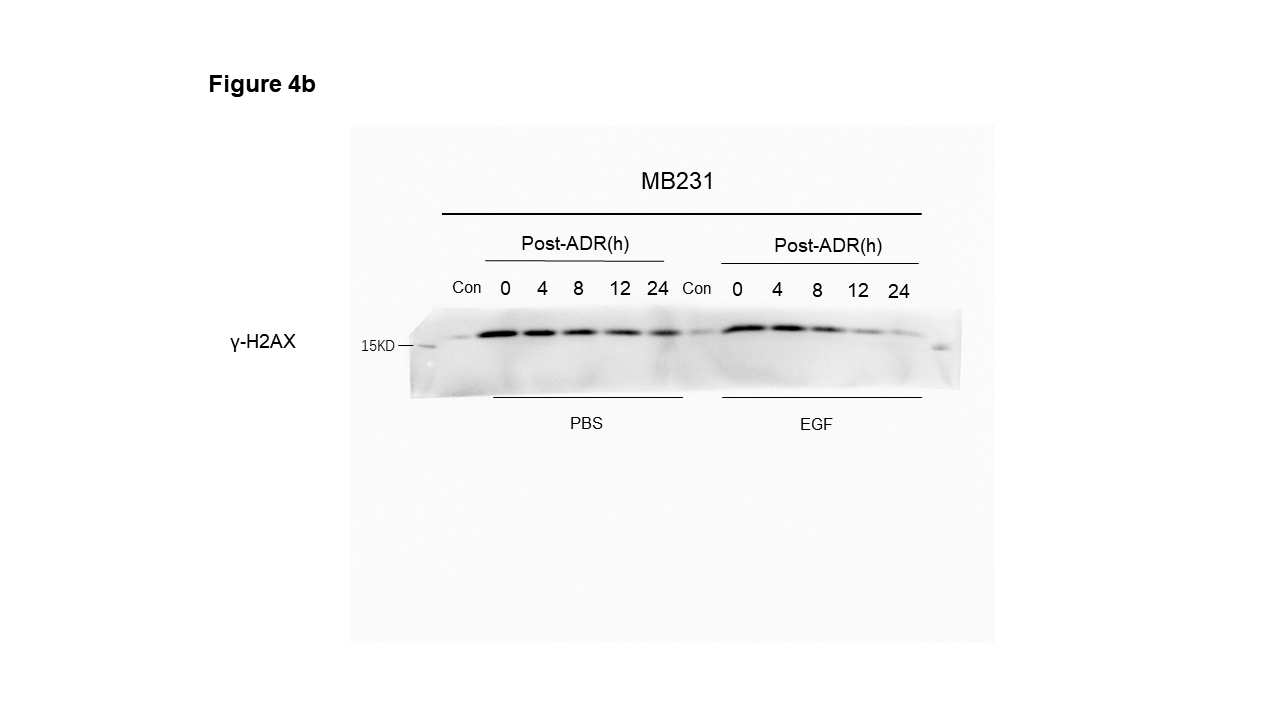

Supplement: Figure 4—source data 2. [file elife-75231-fig4-data2.zip › Figure 4b/Figure 4b γh2ax.TIF]

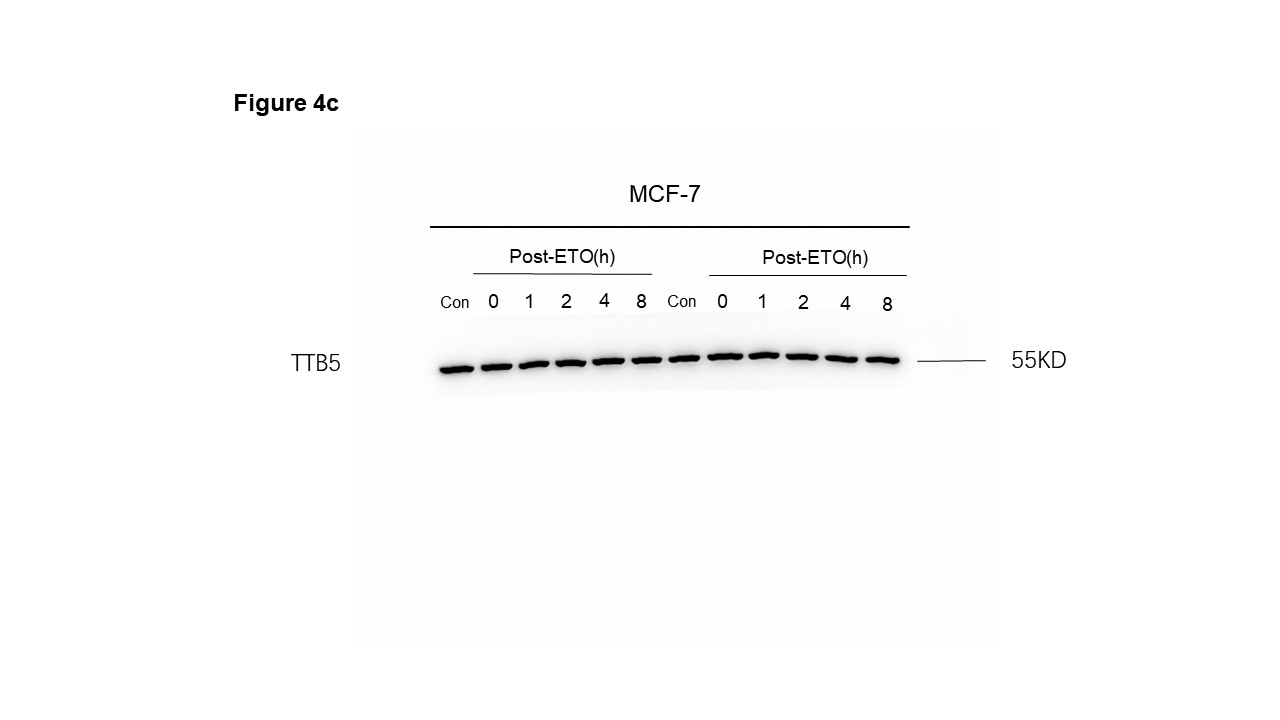

Supplement: Figure 4—source data 3. [file elife-75231-fig4-data3.zip › Figure 4c/Figure 4c tbb5.JPG]

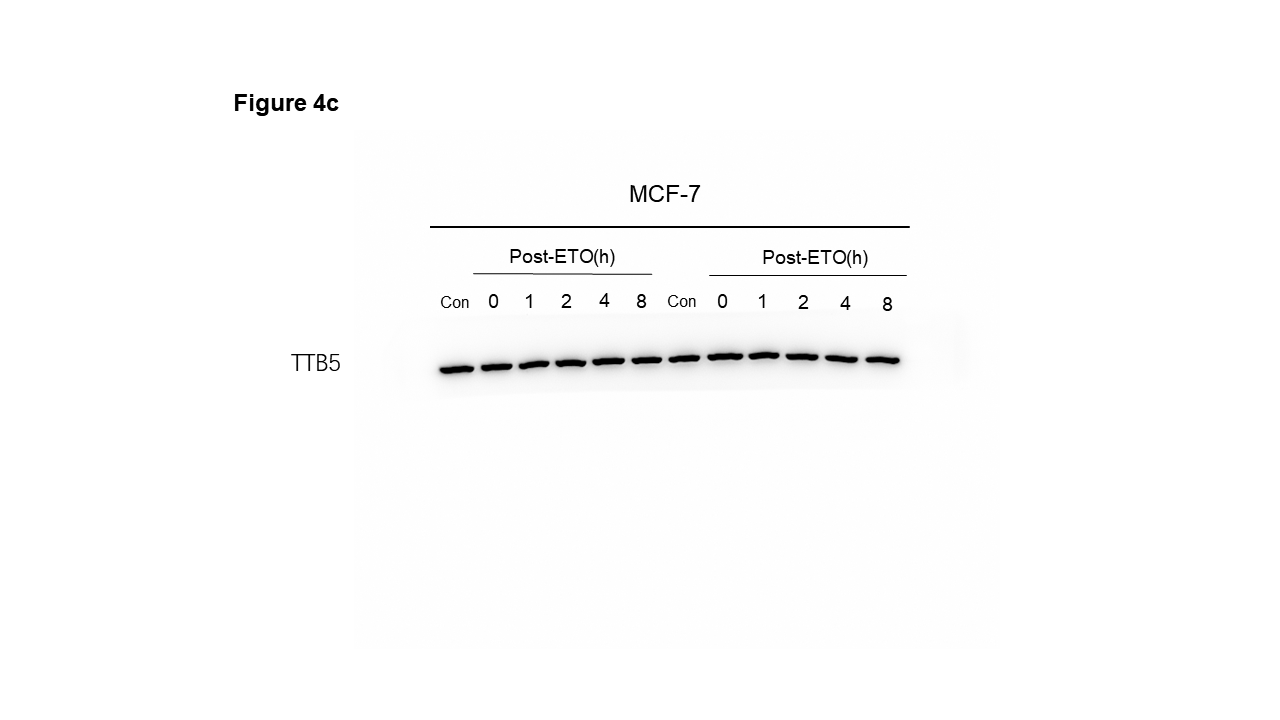

Supplement: Figure 4—source data 3. [file elife-75231-fig4-data3.zip › Figure 4c/Figure 4c tbb5.TIF]

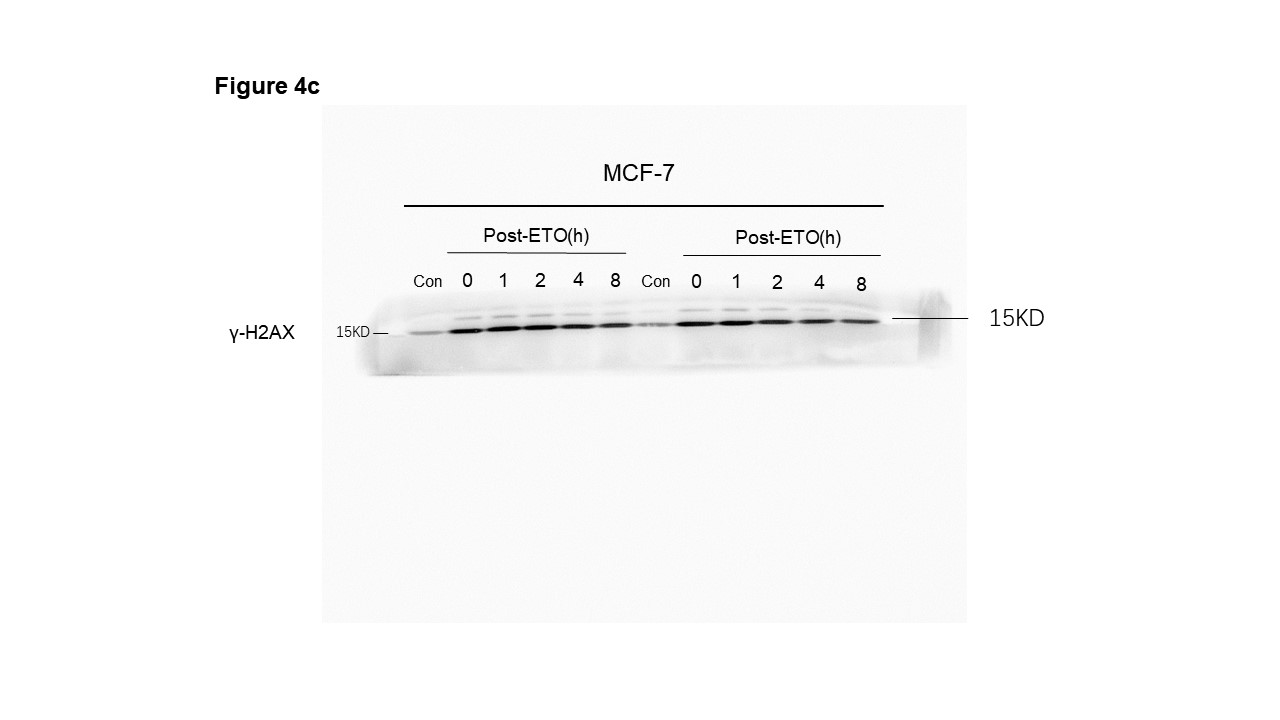

Supplement: Figure 4—source data 3. [file elife-75231-fig4-data3.zip › Figure 4c/Figure 4c γh2ax.JPG]

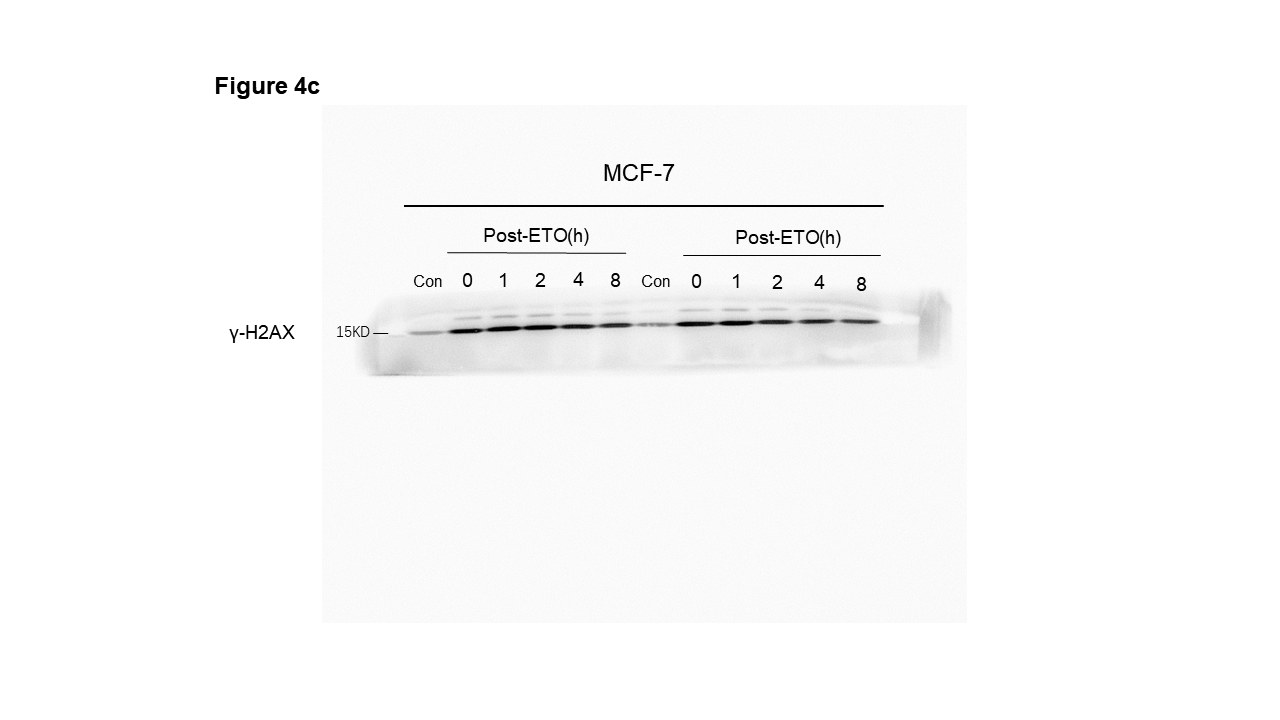

Supplement: Figure 4—source data 3. [file elife-75231-fig4-data3.zip › Figure 4c/Figure 4c γh2ax.TIF]

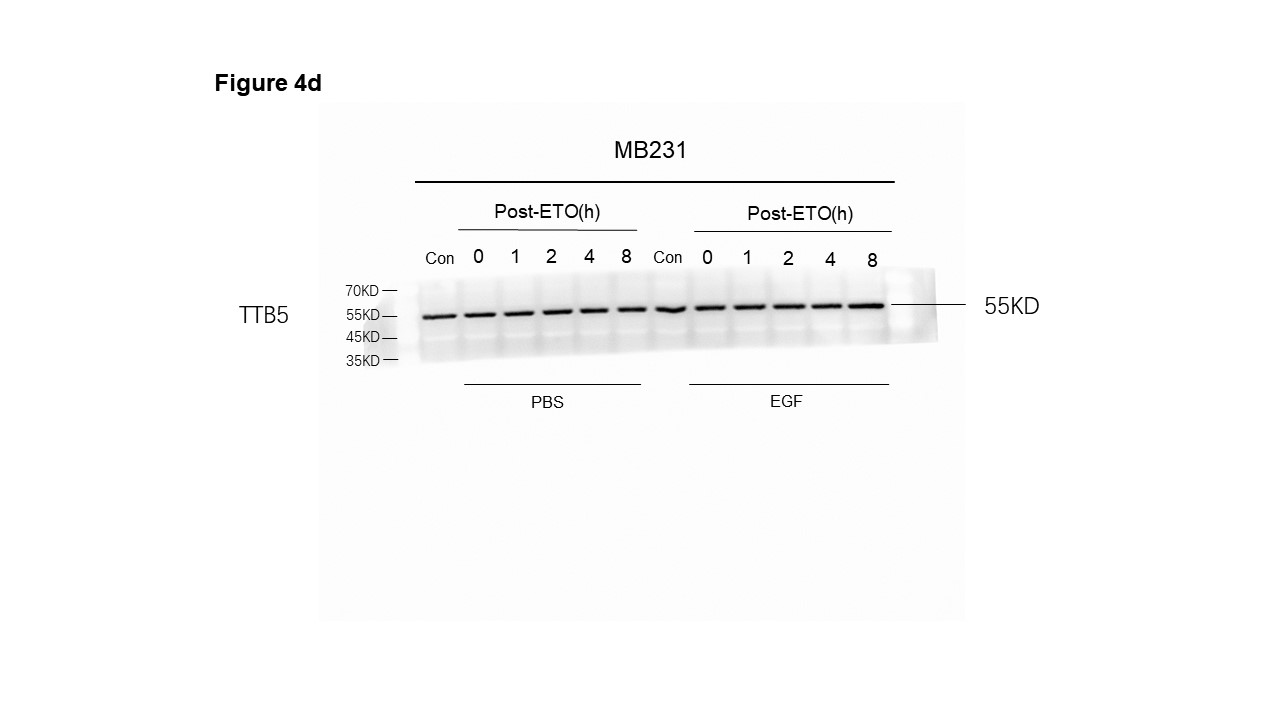

Supplement: Figure 4—source data 4. [file elife-75231-fig4-data4.zip › Figure 4d/Figure 4d tbb5.JPG]

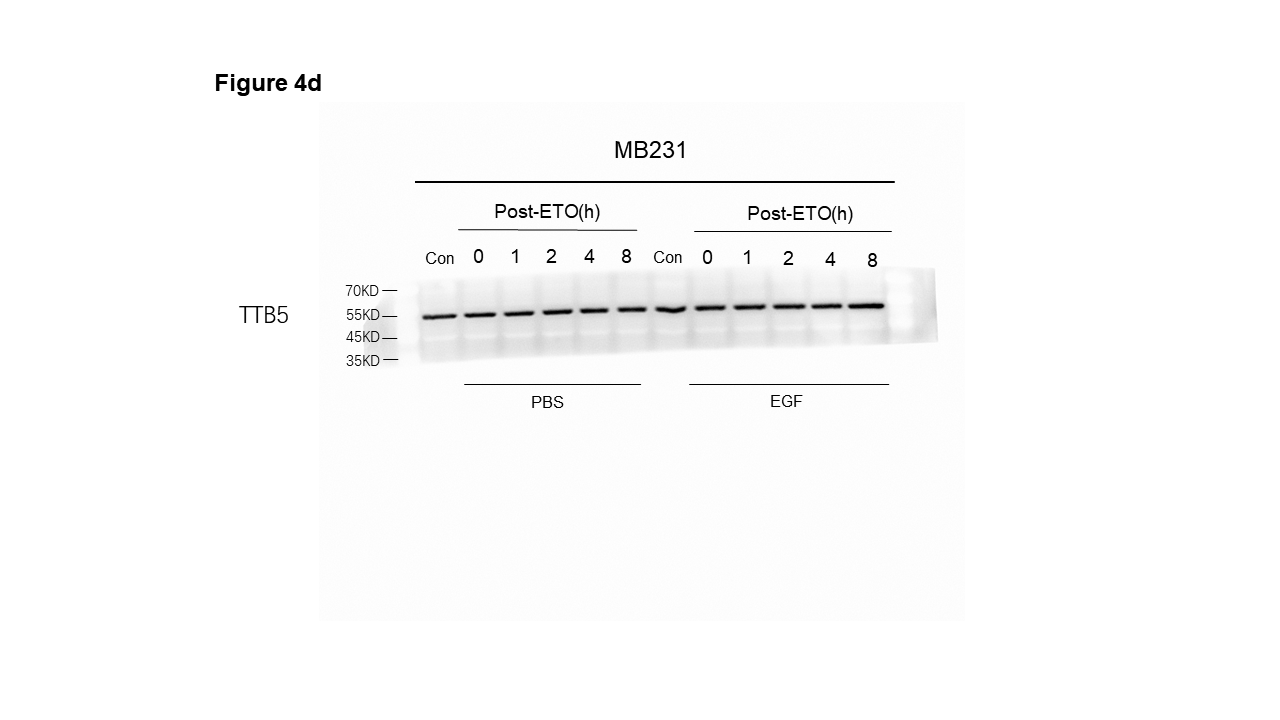

Supplement: Figure 4—source data 4. [file elife-75231-fig4-data4.zip › Figure 4d/Figure 4d tbb5.TIF]

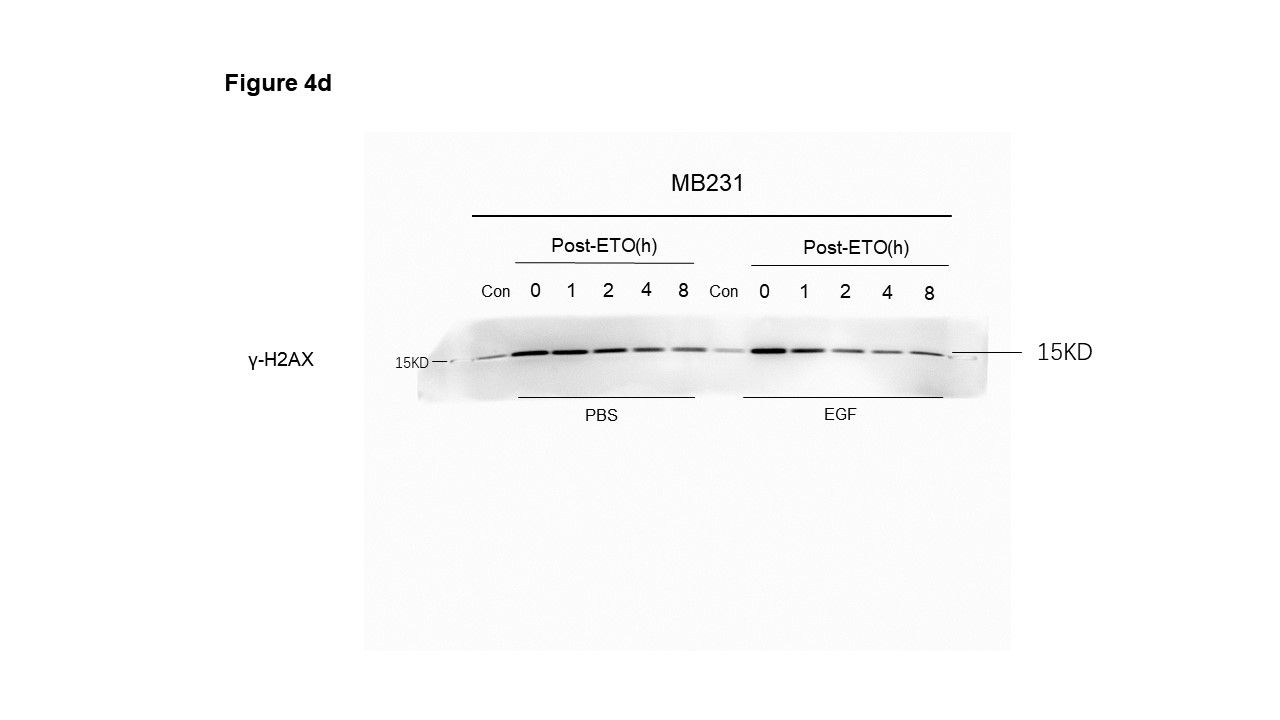

Supplement: Figure 4—source data 4. [file elife-75231-fig4-data4.zip › Figure 4d/Figure 4d γh2ax.JPG]

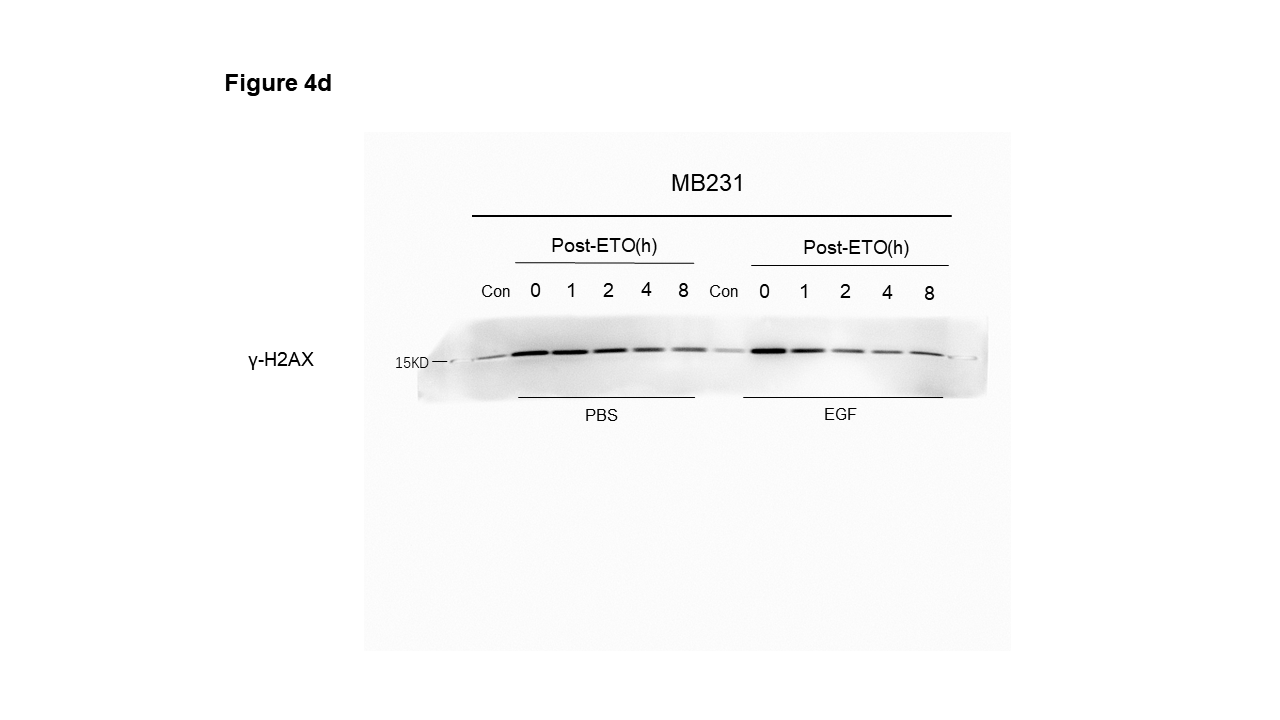

Supplement: Figure 4—source data 4. [file elife-75231-fig4-data4.zip › Figure 4d/Figure 4d γh2ax.TIF]

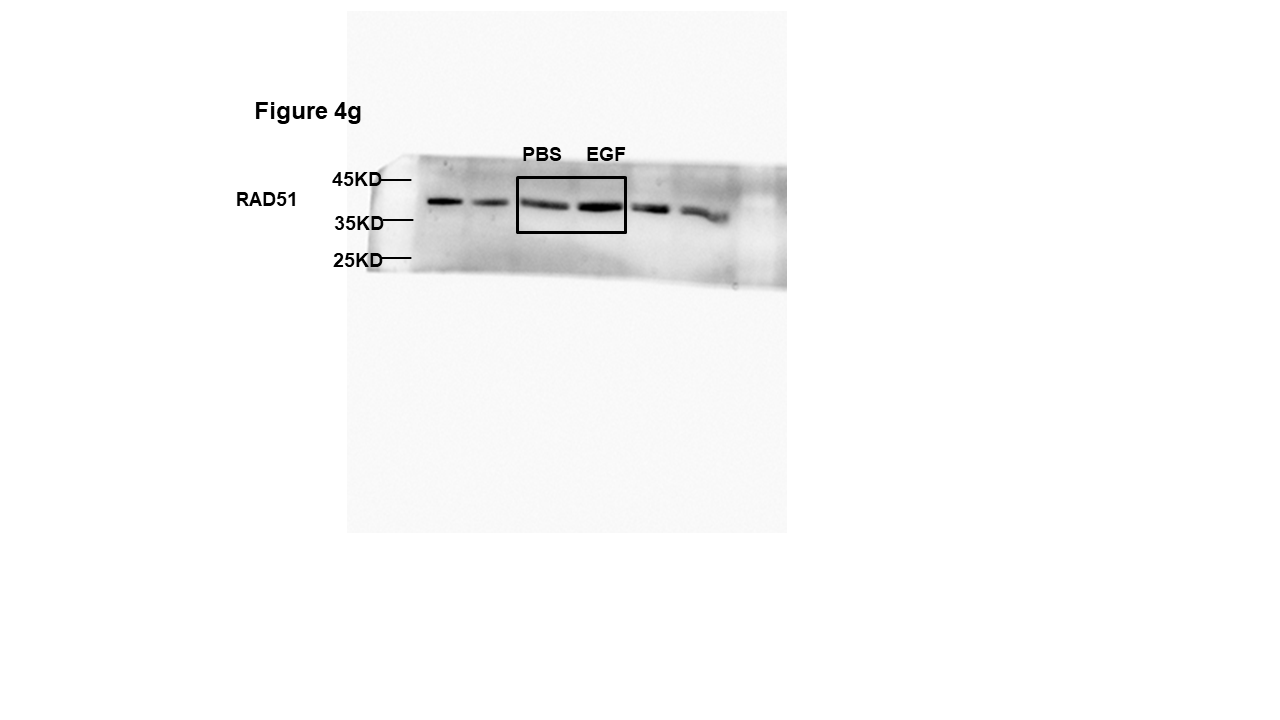

Supplement: Figure 4—source data 5. [file elife-75231-fig4-data5.zip › Figure 4g/Figure 4g RAD51.TIF]

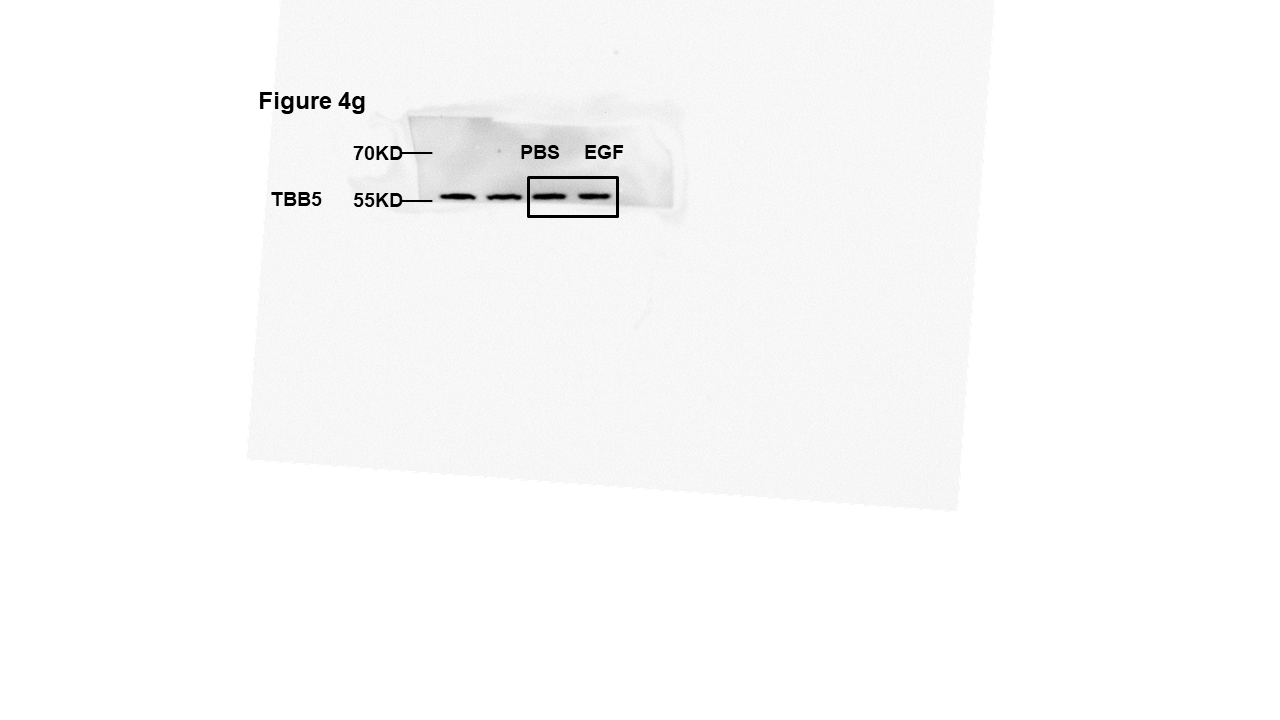

Supplement: Figure 4—source data 5. [file elife-75231-fig4-data5.zip › Figure 4g/Figure 4g TBB5.TIF]

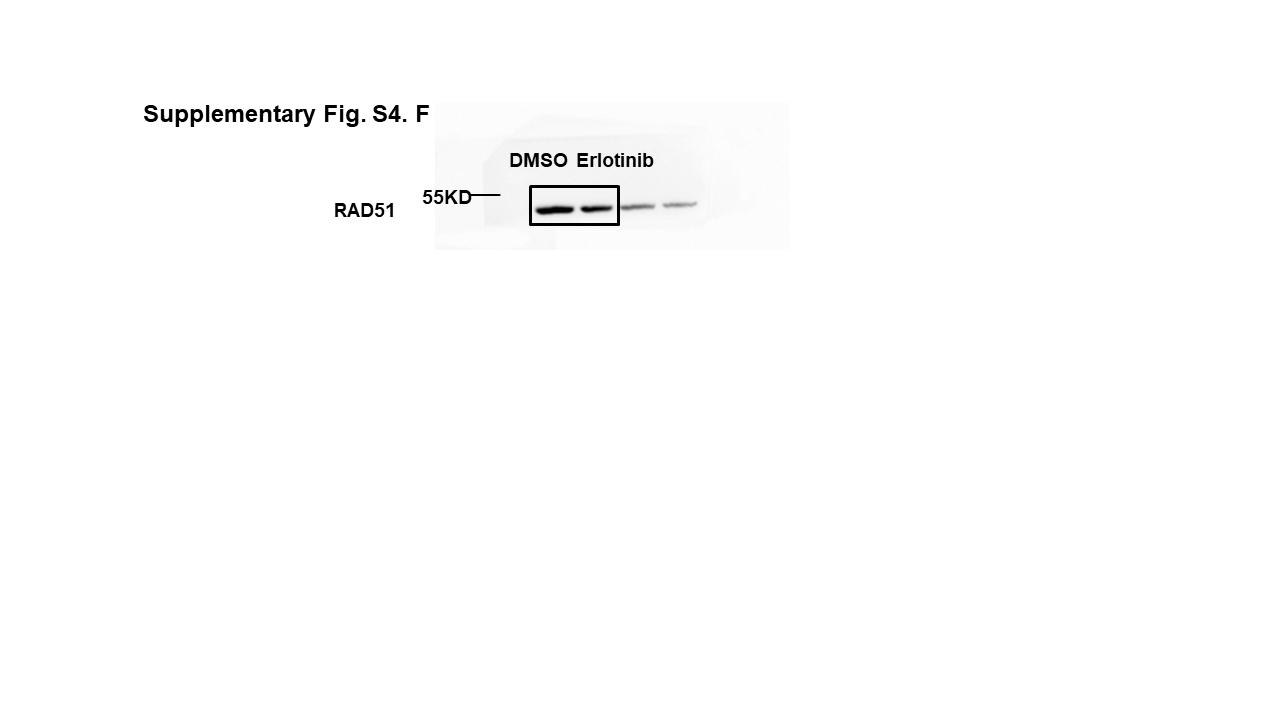

Supplement: Figure 4—figure supplement 1—source data 1. [file elife-75231-fig4-figsupp1-data1.zip › Figure S4F/Supplementary Fig.S4. F RAD51.png.TIF]

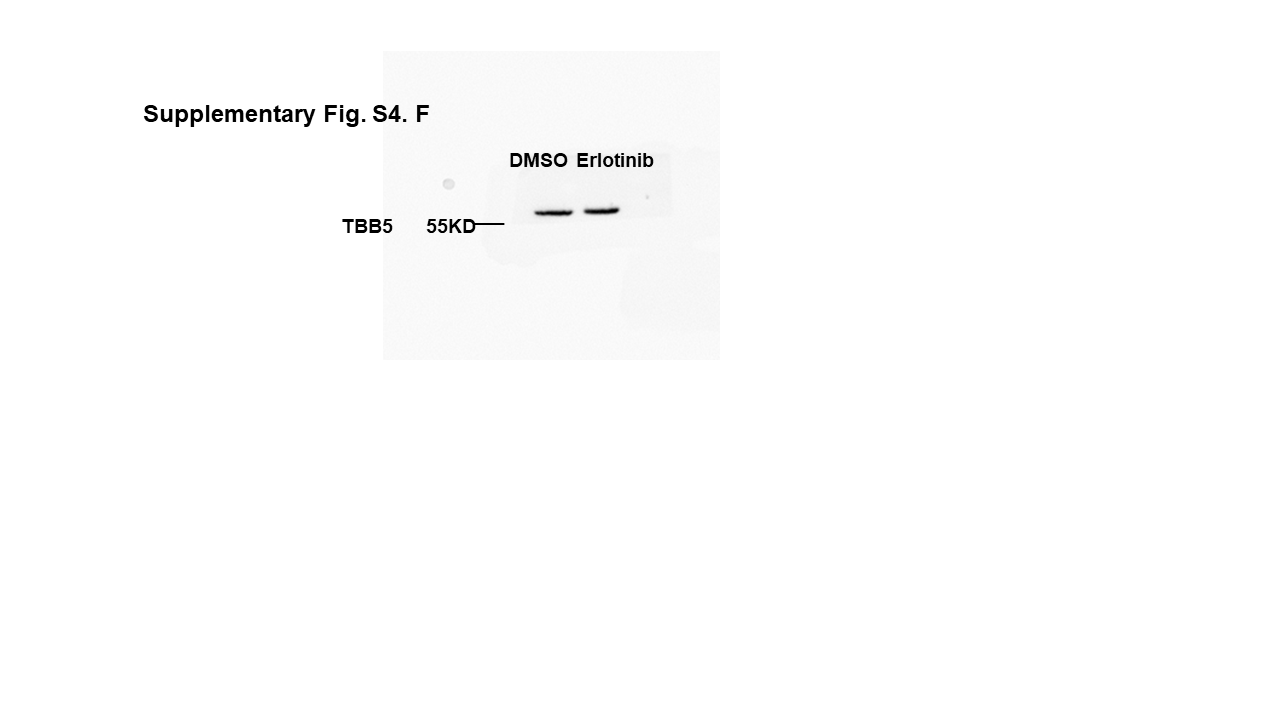

Supplement: Figure 4—figure supplement 1—source data 1. [file elife-75231-fig4-figsupp1-data1.zip › Figure S4F/Supplementary Fig.S4. F TBB5.png.TIF]

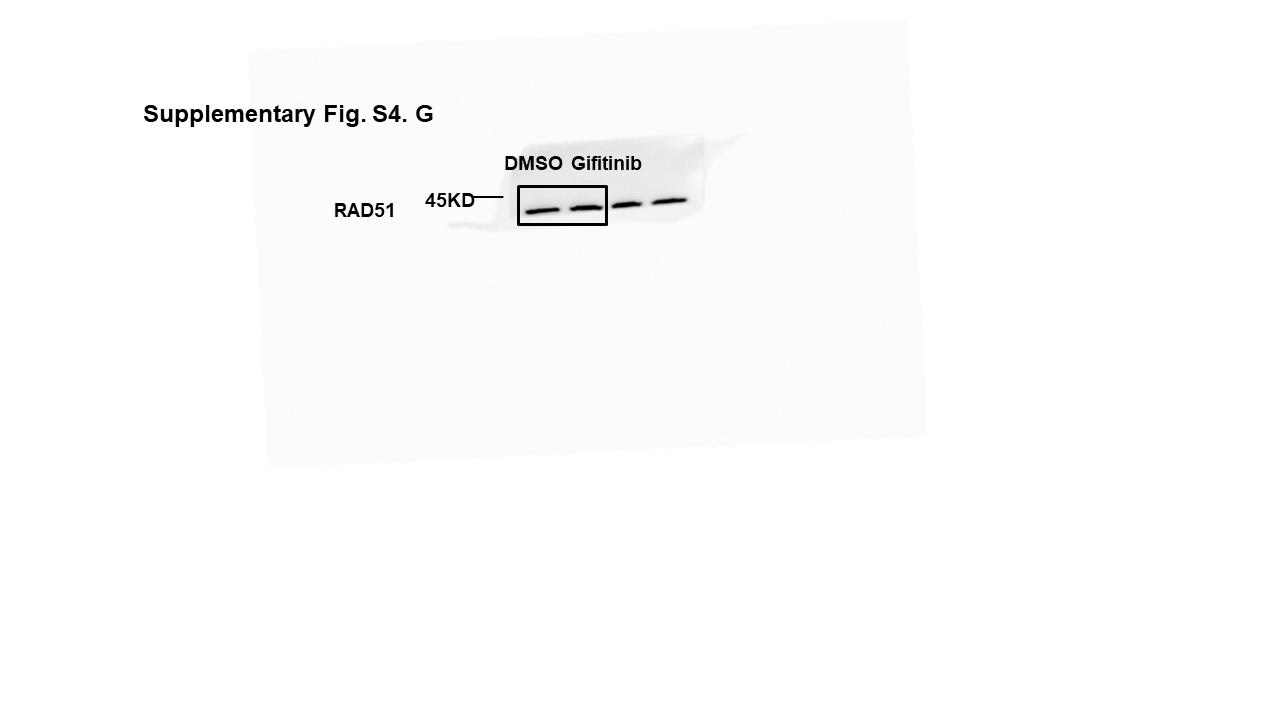

Supplement: Figure 4—figure supplement 1—source data 2. [file elife-75231-fig4-figsupp1-data2.zip › Figure S4G/Supplementary Fig.S4. G RAD51.png.TIF]

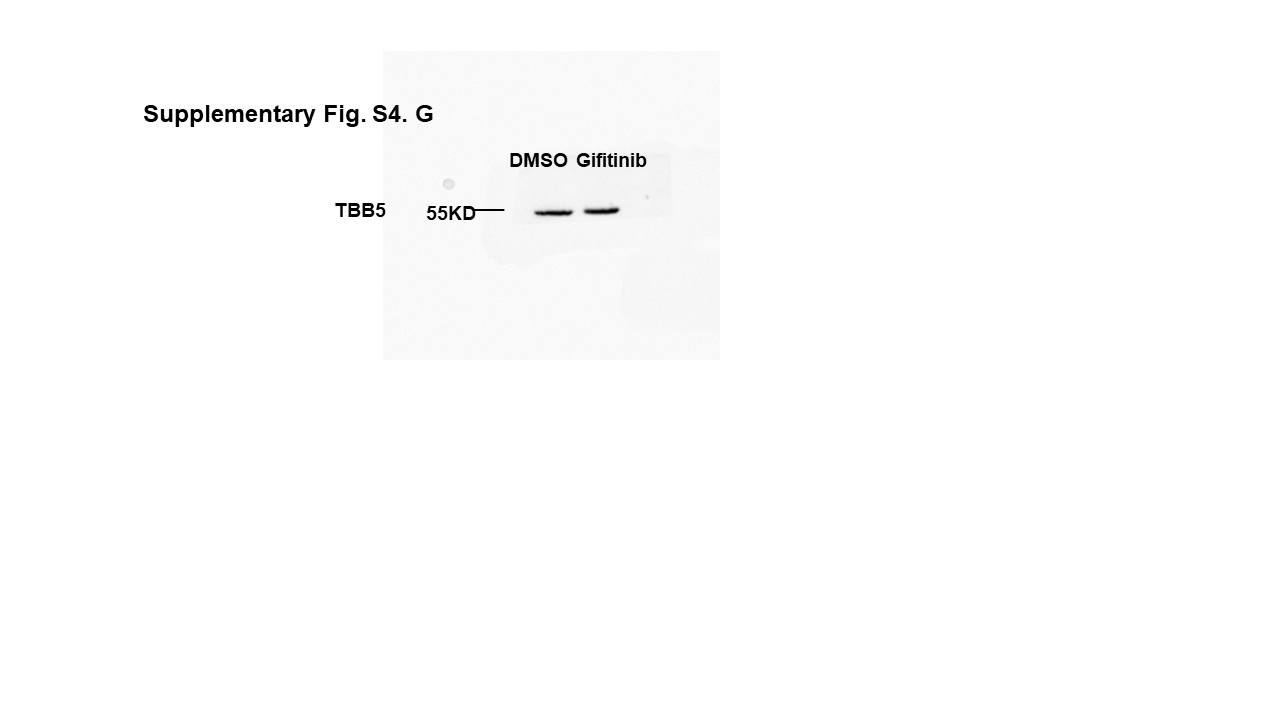

Supplement: Figure 4—figure supplement 1—source data 2. [file elife-75231-fig4-figsupp1-data2.zip › Figure S4G/Supplementary Fig.S4. G TBB5.png.TIF]

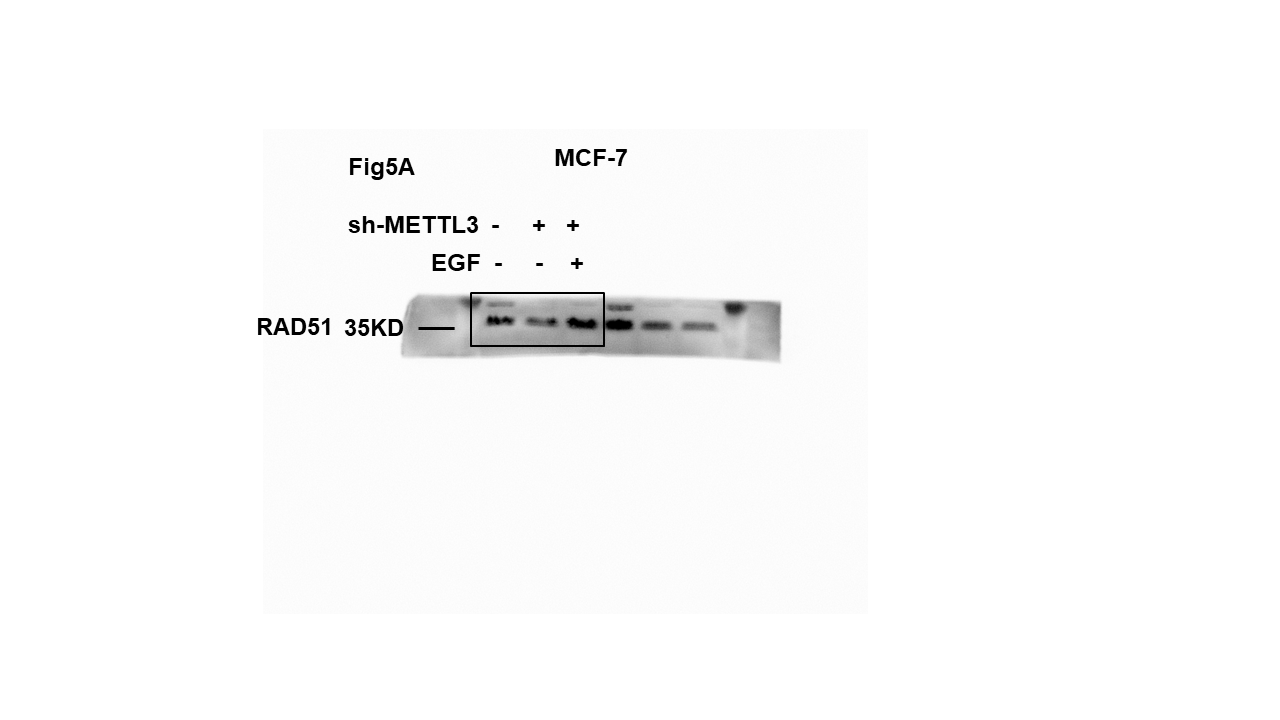

Supplement: Figure 5—source data 1. [file elife-75231-fig5-data1.zip › Figure 5a/Figure 5a RAD51.TIF]

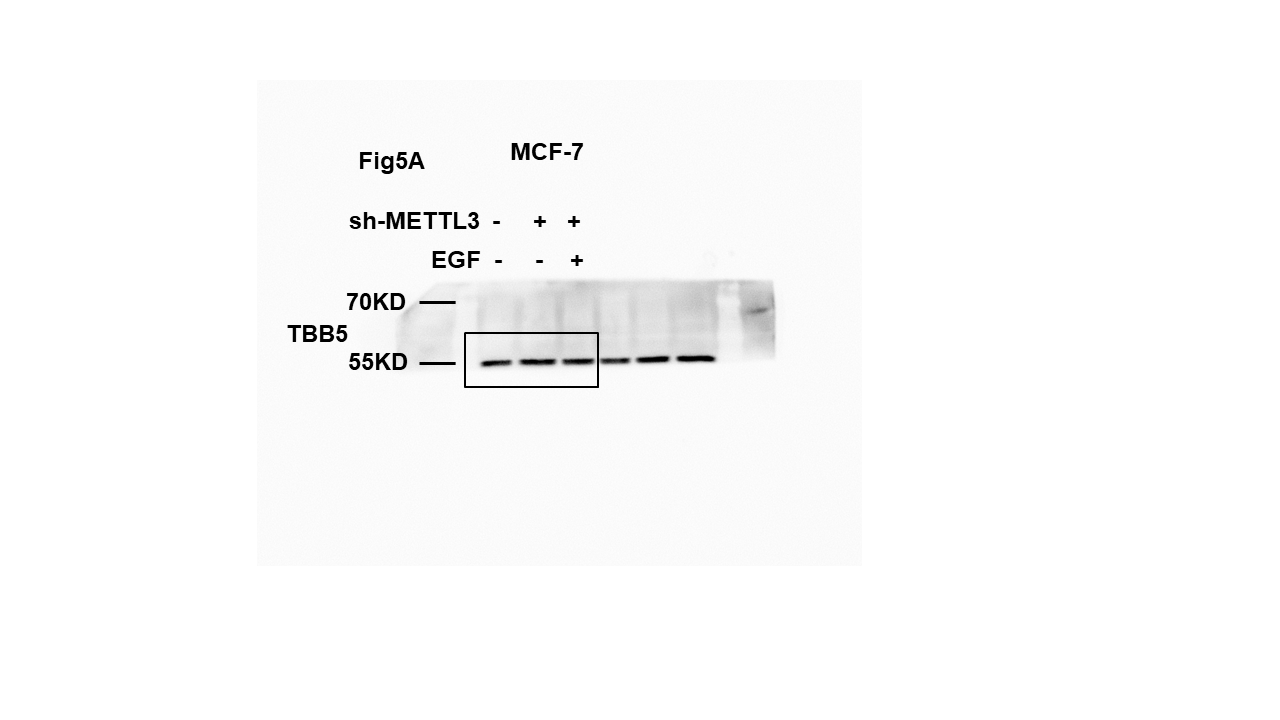

Supplement: Figure 5—source data 1. [file elife-75231-fig5-data1.zip › Figure 5a/Figure 5a TBB5.TIF]

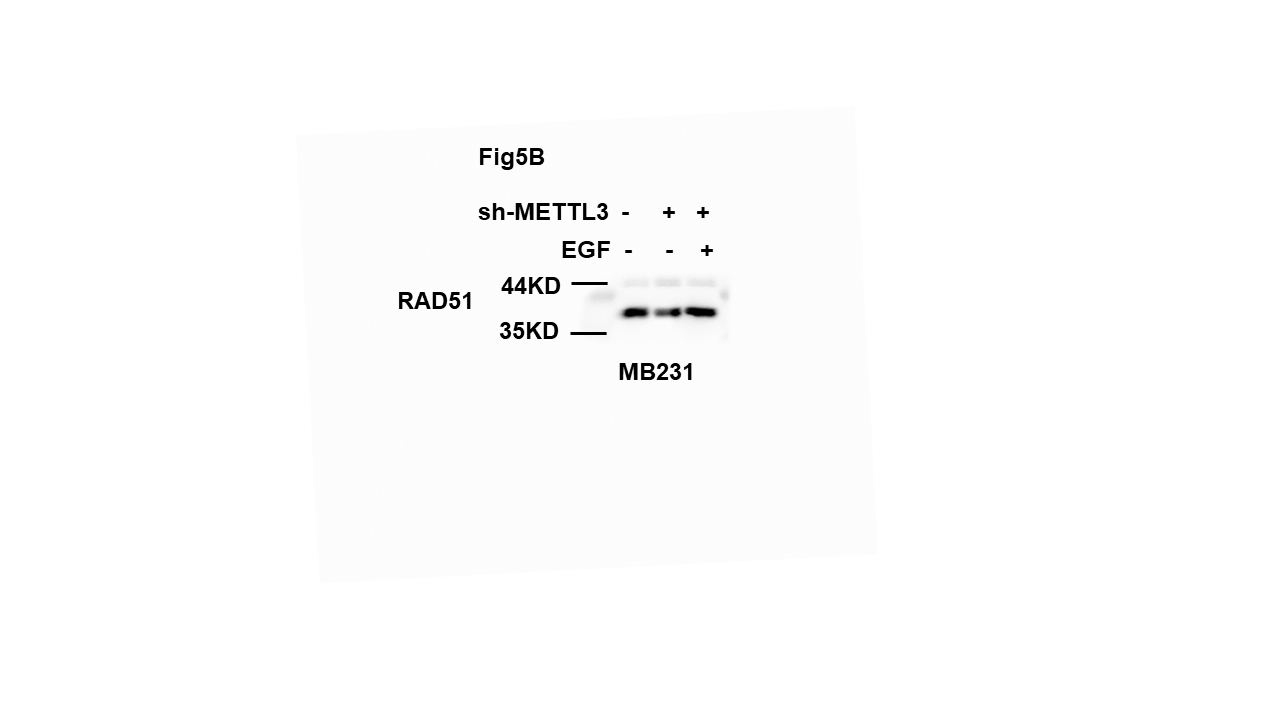

Supplement: Figure 5—source data 2. [file elife-75231-fig5-data2.zip › Figure 5b/Figure 5b RAD51.TIF]

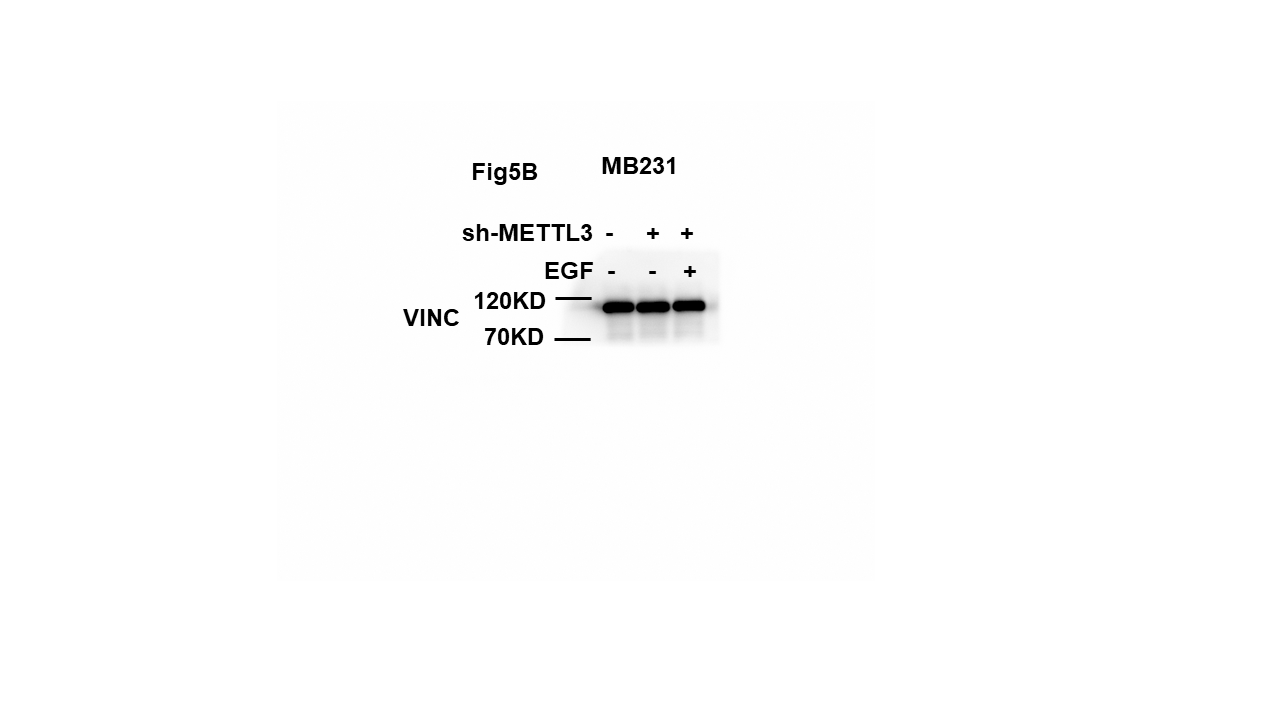

Supplement: Figure 5—source data 2. [file elife-75231-fig5-data2.zip › Figure 5b/Figure 5b VINC.TIF]

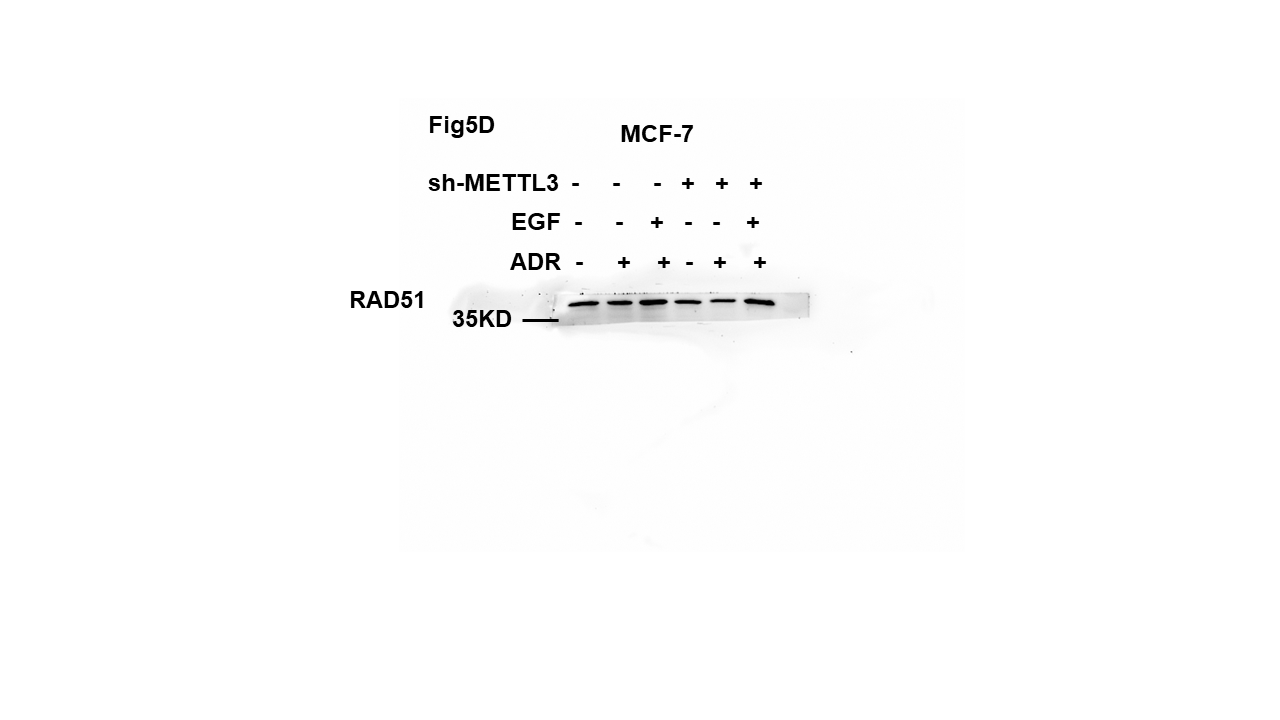

Supplement: Figure 5—source data 3. [file elife-75231-fig5-data3.zip › Figure 5d/Figure 5d RAD51.TIF]

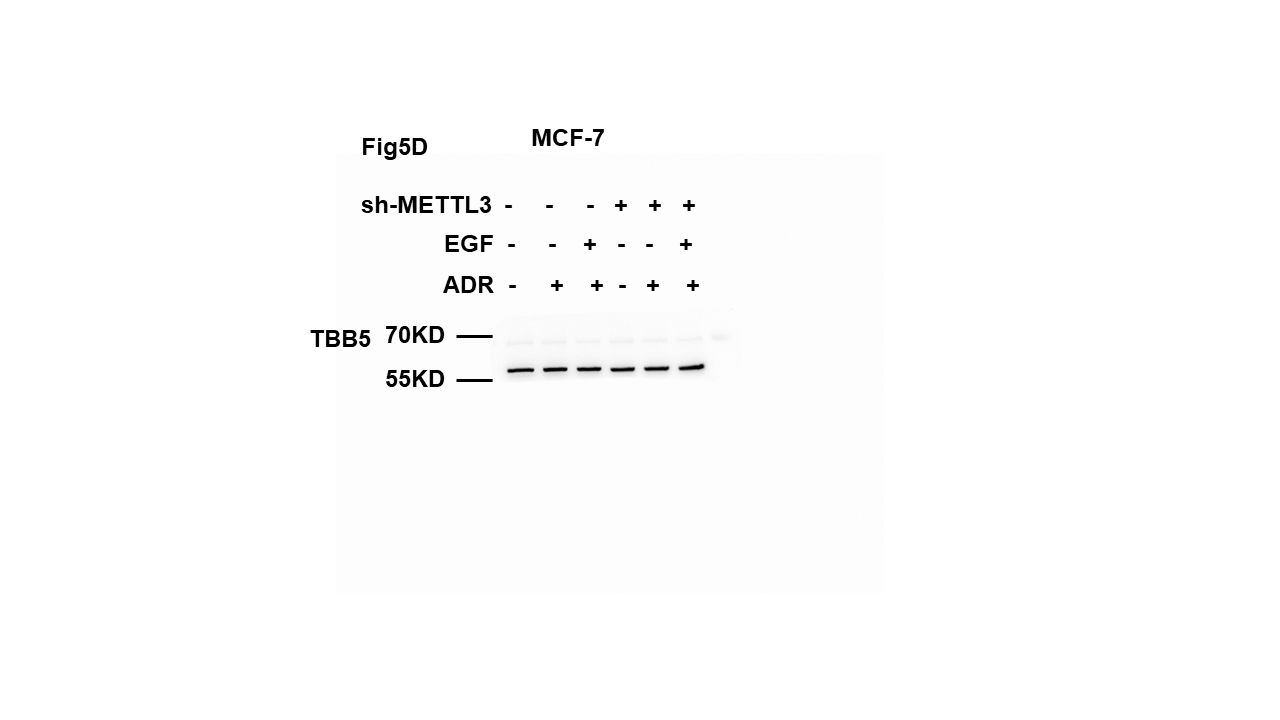

Supplement: Figure 5—source data 3. [file elife-75231-fig5-data3.zip › Figure 5d/Figure 5d TBB5.TIF]

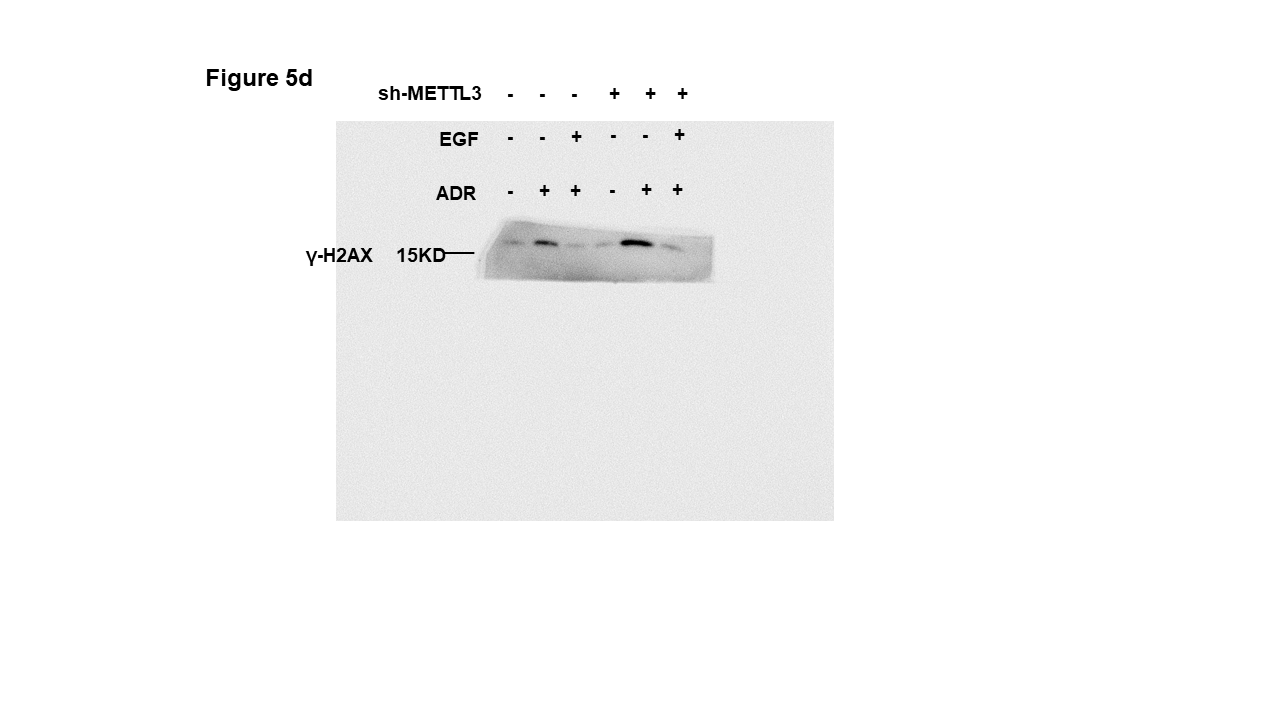

Supplement: Figure 5—source data 3. [file elife-75231-fig5-data3.zip › Figure 5d/Figure 5d γ-H2AX.TIF]

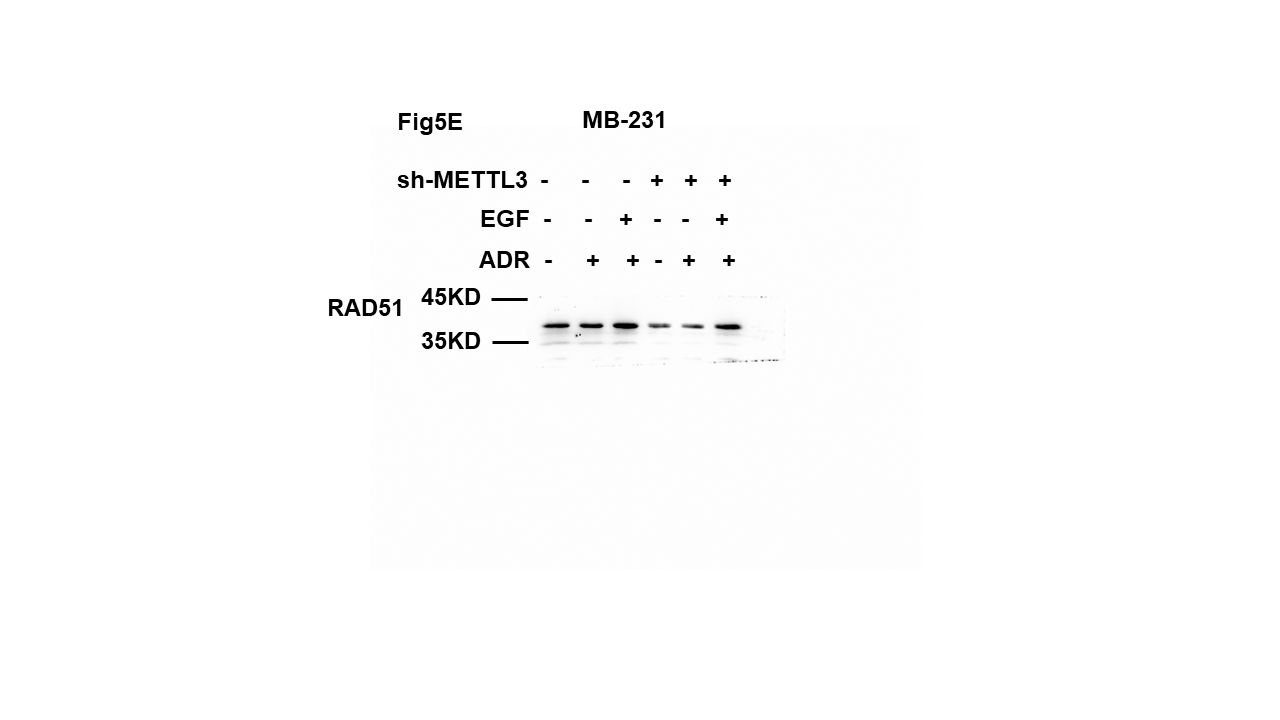

Supplement: Figure 5—source data 4. [file elife-75231-fig5-data4.zip › Figure 5e/Figure 5e RAD51.TIF]

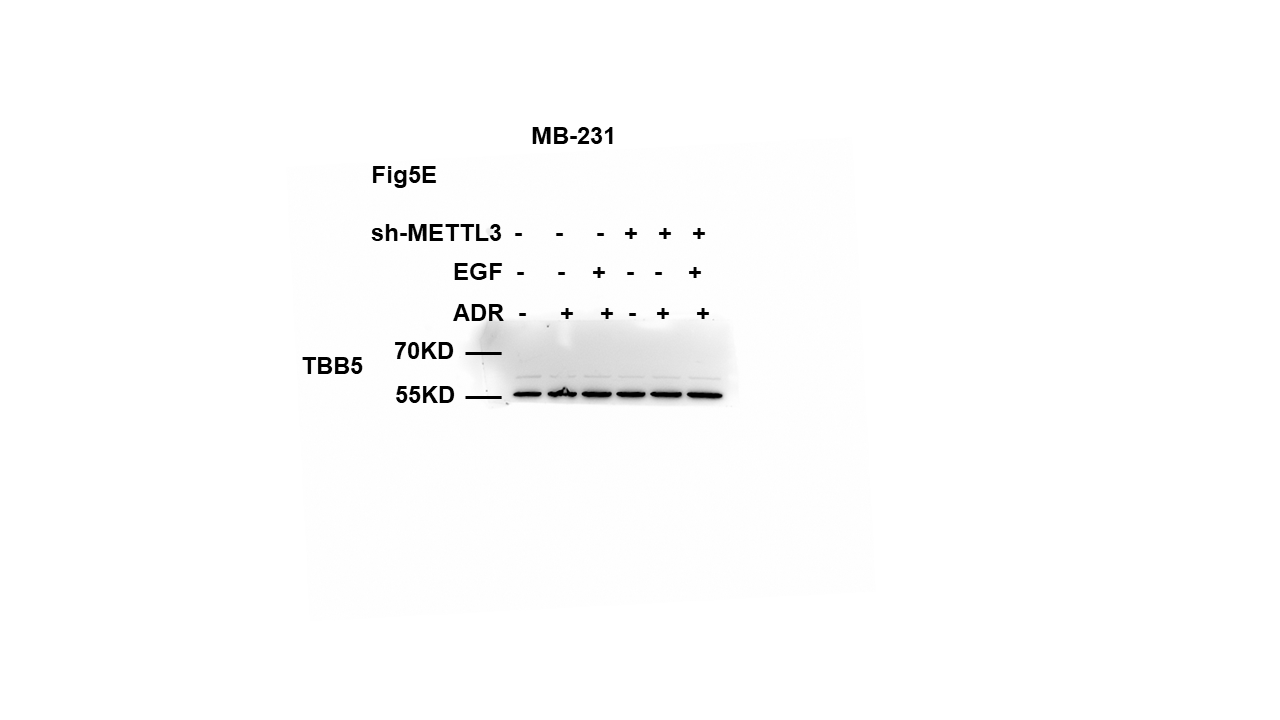

Supplement: Figure 5—source data 4. [file elife-75231-fig5-data4.zip › Figure 5e/Figure 5e TBB5.TIF]

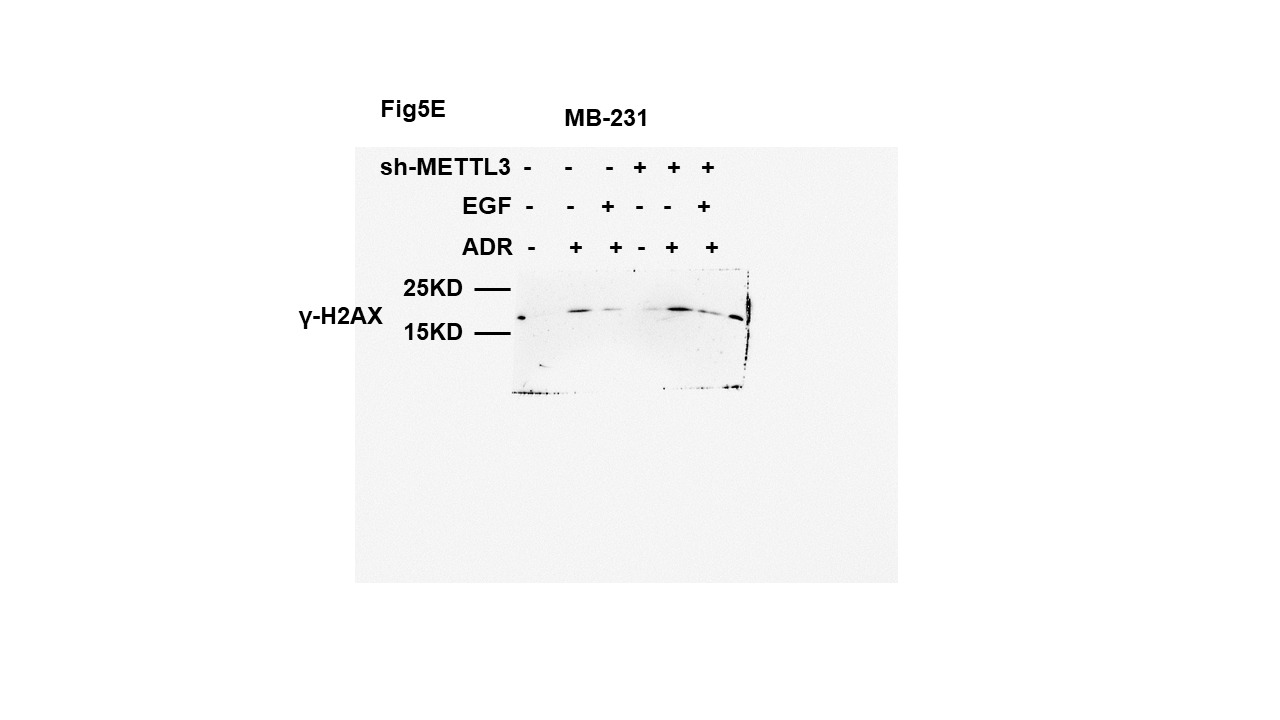

Supplement: Figure 5—source data 4. [file elife-75231-fig5-data4.zip › Figure 5e/Figure 5e γ-H2AX.TIF]

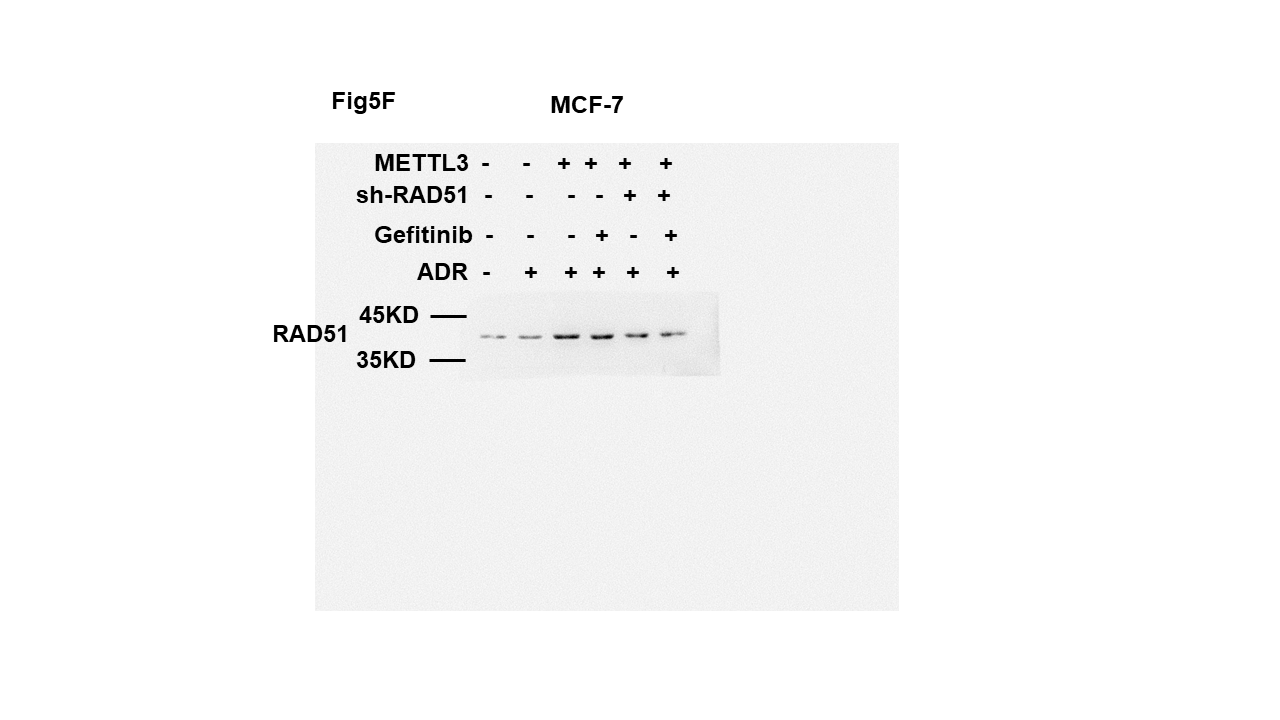

Supplement: Figure 5—source data 5. [file elife-75231-fig5-data5.zip › Figure 5f/Figure 5f RAD51.TIF]

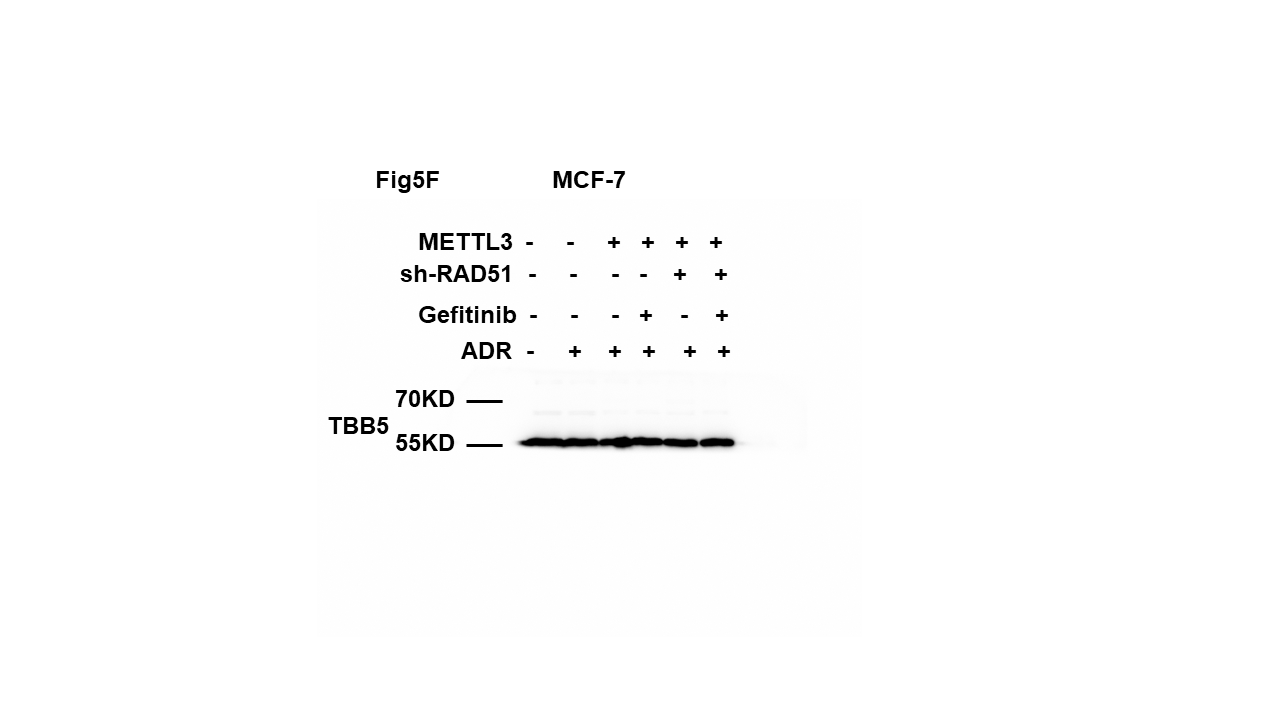

Supplement: Figure 5—source data 5. [file elife-75231-fig5-data5.zip › Figure 5f/Figure 5f TBB5.TIF]

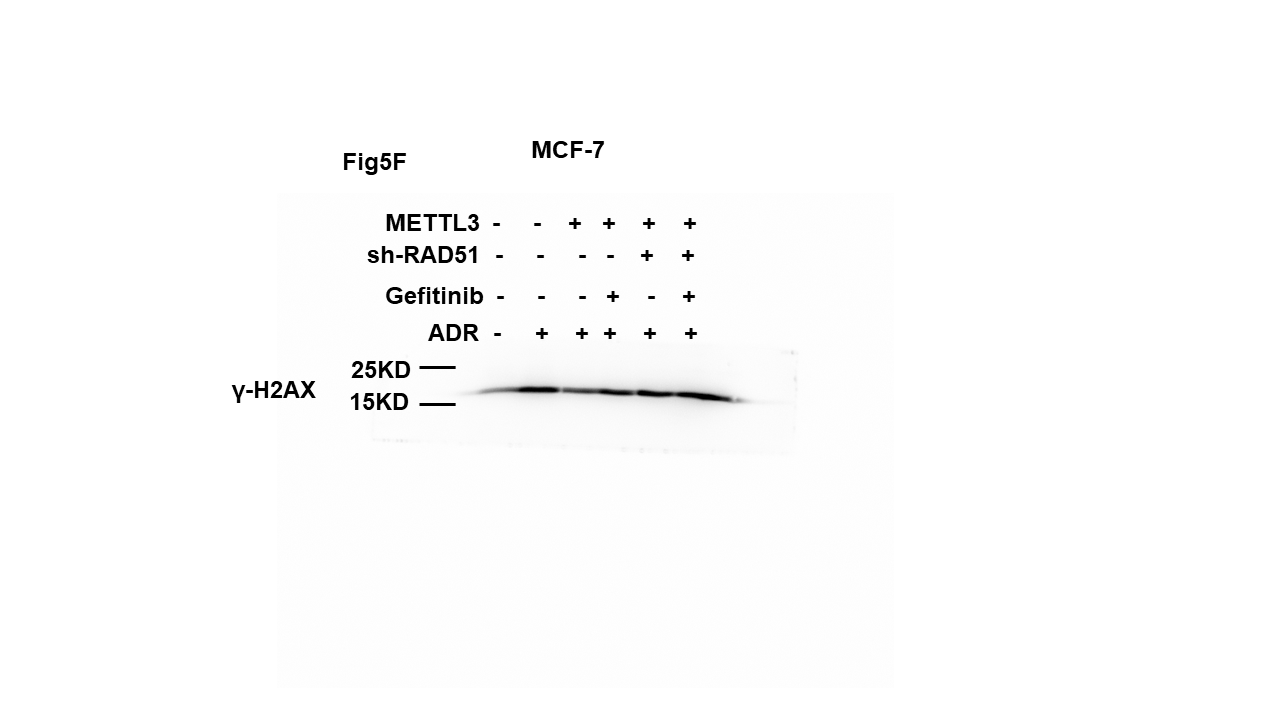

Supplement: Figure 5—source data 5. [file elife-75231-fig5-data5.zip › Figure 5f/Figure 5f γ-H2AX.TIF]

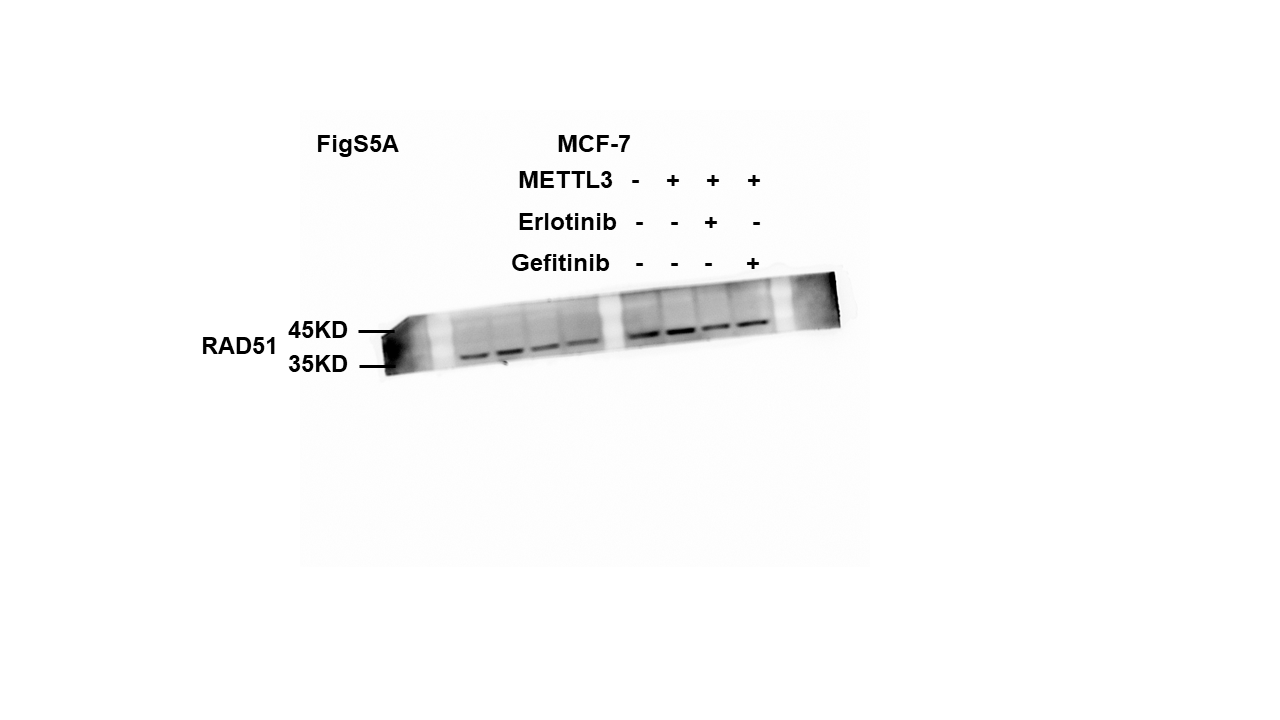

Supplement: Figure 5—figure supplement 1—source data 1. [file elife-75231-fig5-figsupp1-data1.zip › Figure S5A/Figure S5A RAD51.TIF]
