# Supplementary figures and images for: METTL3 promotes homologous recombination repair and modulates chemotherapeutic response in breast cancer by regulating the EGF/RAD51 axis (part 2 of 2)
Source: eLife. 2022 May 3;11:e75231. doi: 10.7554/eLife.75231 (PMC9094751; doi:10.7554/eLife.75231)

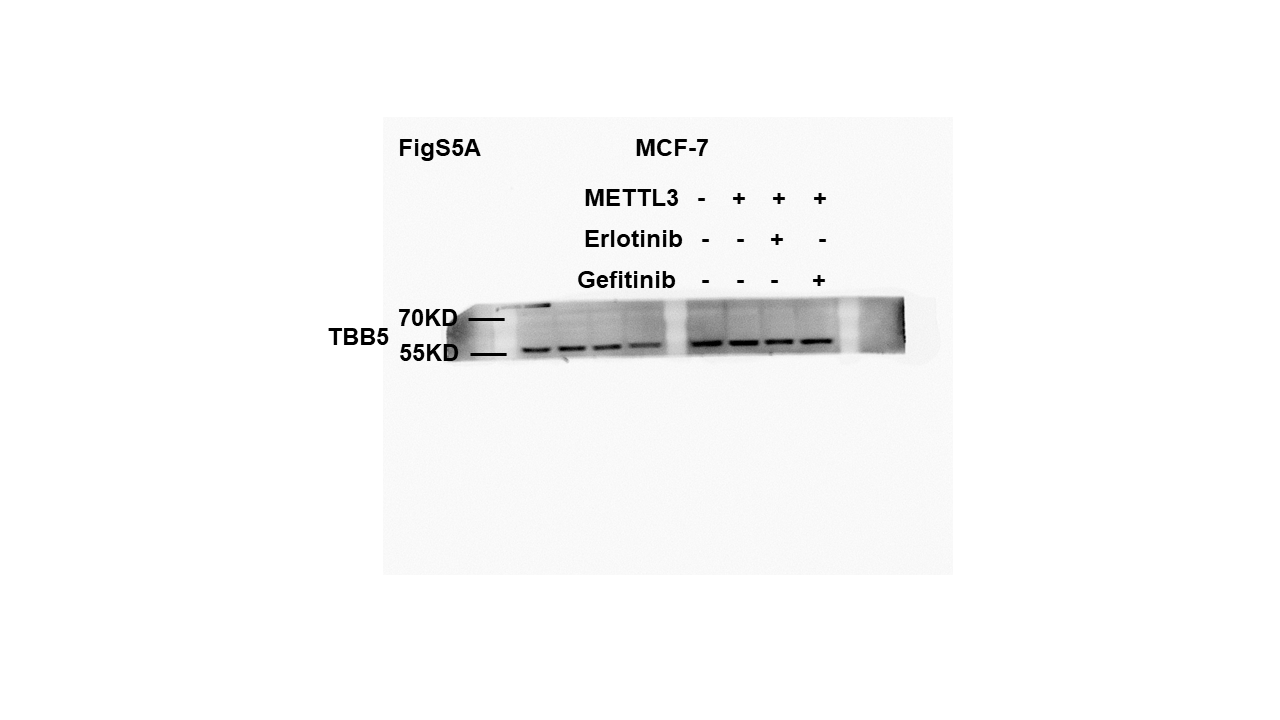

Supplement: Figure 5—figure supplement 1—source data 1. [file elife-75231-fig5-figsupp1-data1.zip › Figure S5A/Figure S5A TBB5.TIF]

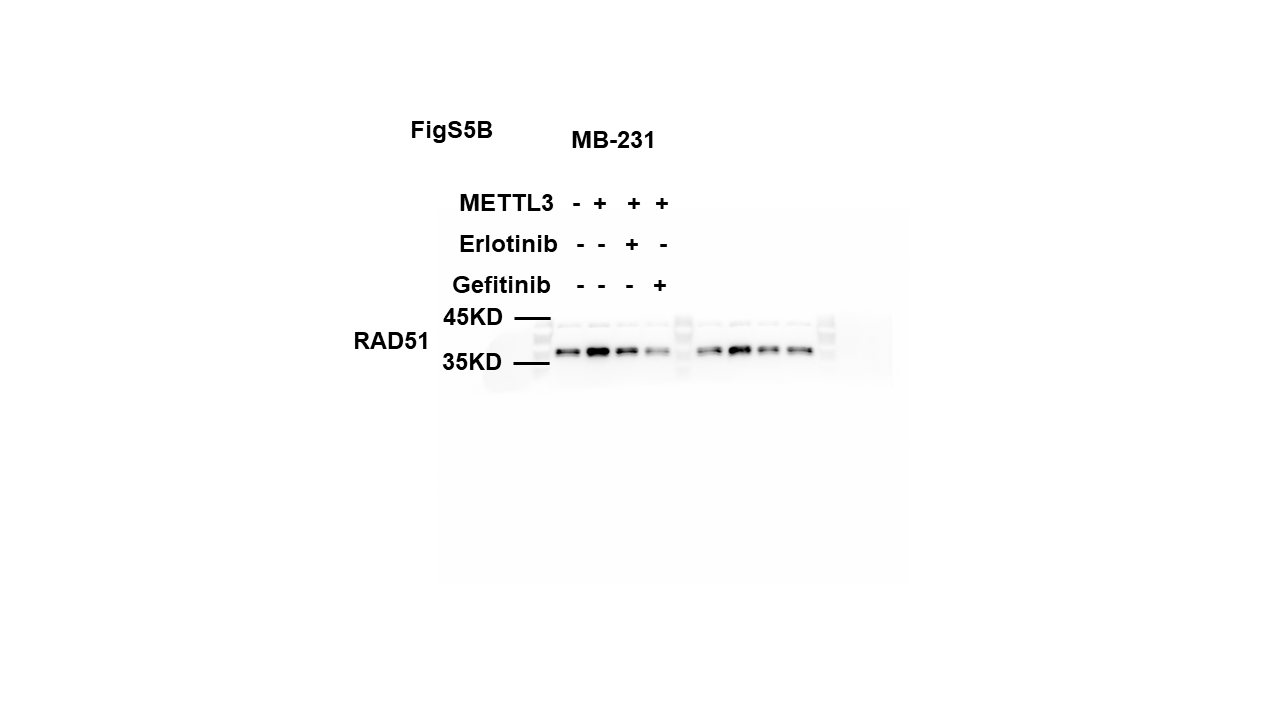

Supplement: Figure 5—figure supplement 1—source data 2. [file elife-75231-fig5-figsupp1-data2.zip › Figure S5B/Figure S5B RAD51.TIF]

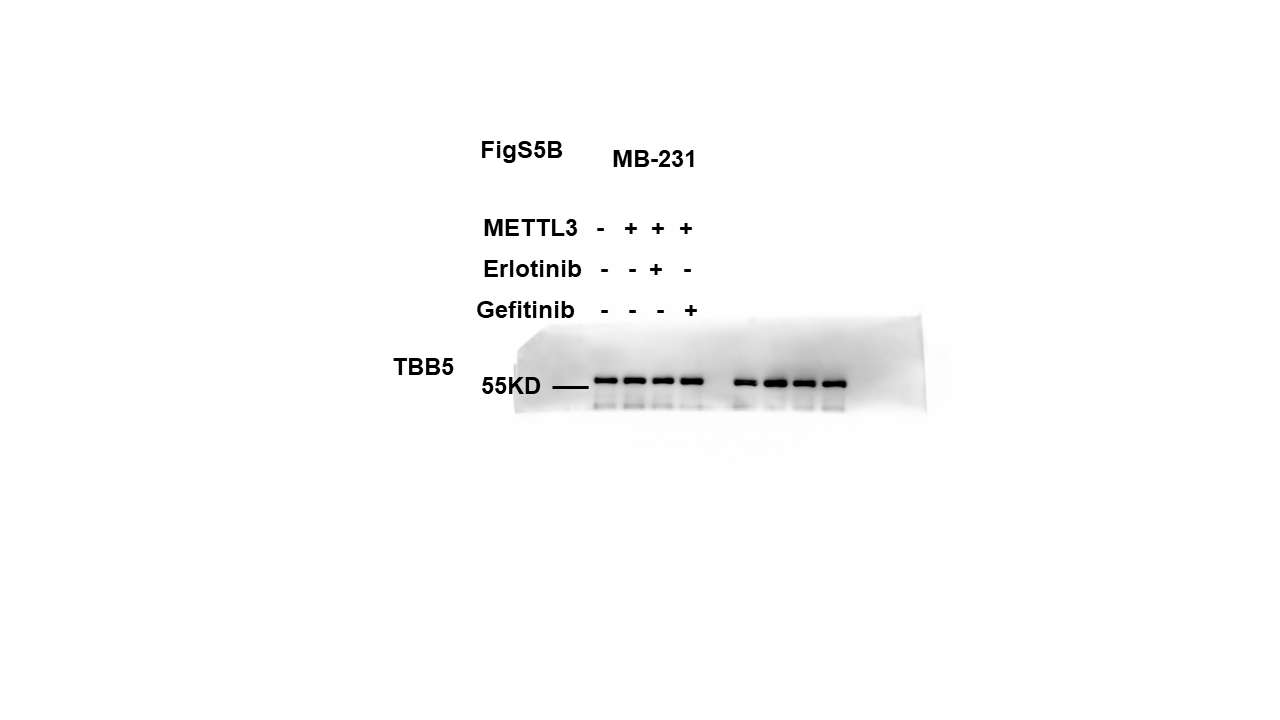

Supplement: Figure 5—figure supplement 1—source data 2. [file elife-75231-fig5-figsupp1-data2.zip › Figure S5B/Figure S5B TBB5.TIF]

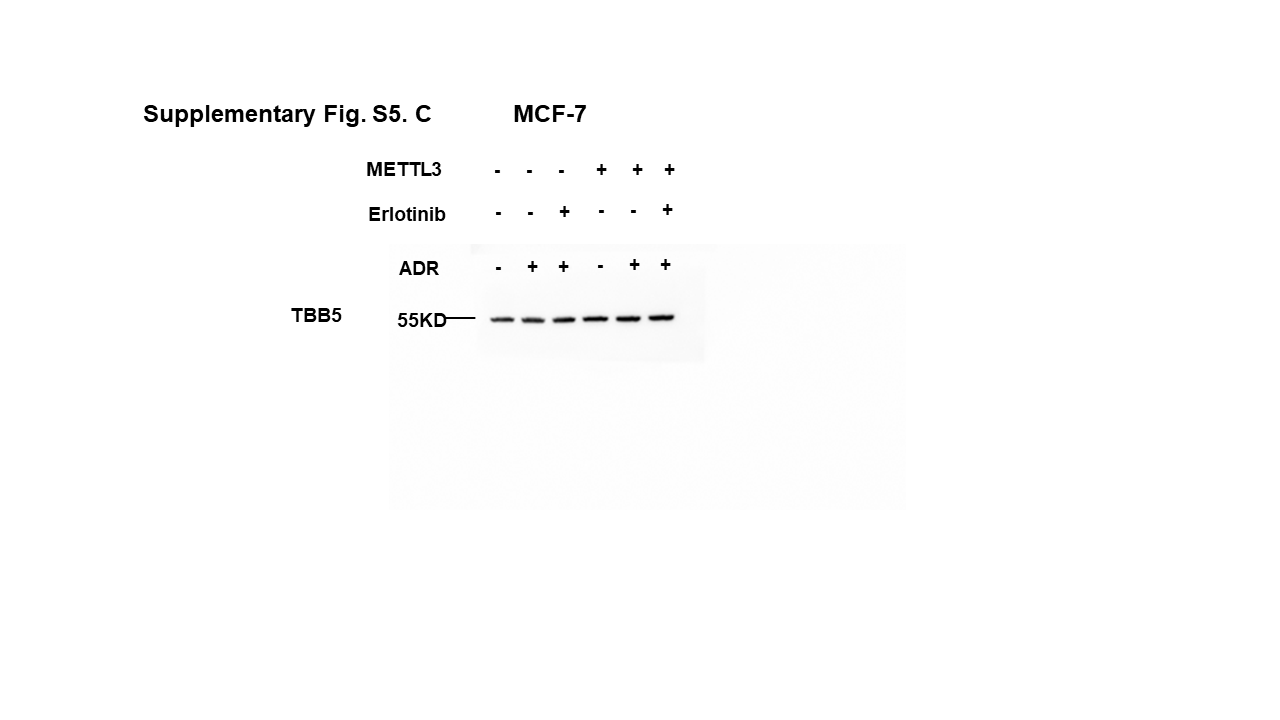

Supplement: Figure 5—figure supplement 1—source data 3. [file elife-75231-fig5-figsupp1-data3.zip › Figure S5C/Supplementary Fig.S5. C TBB5.png.TIF]

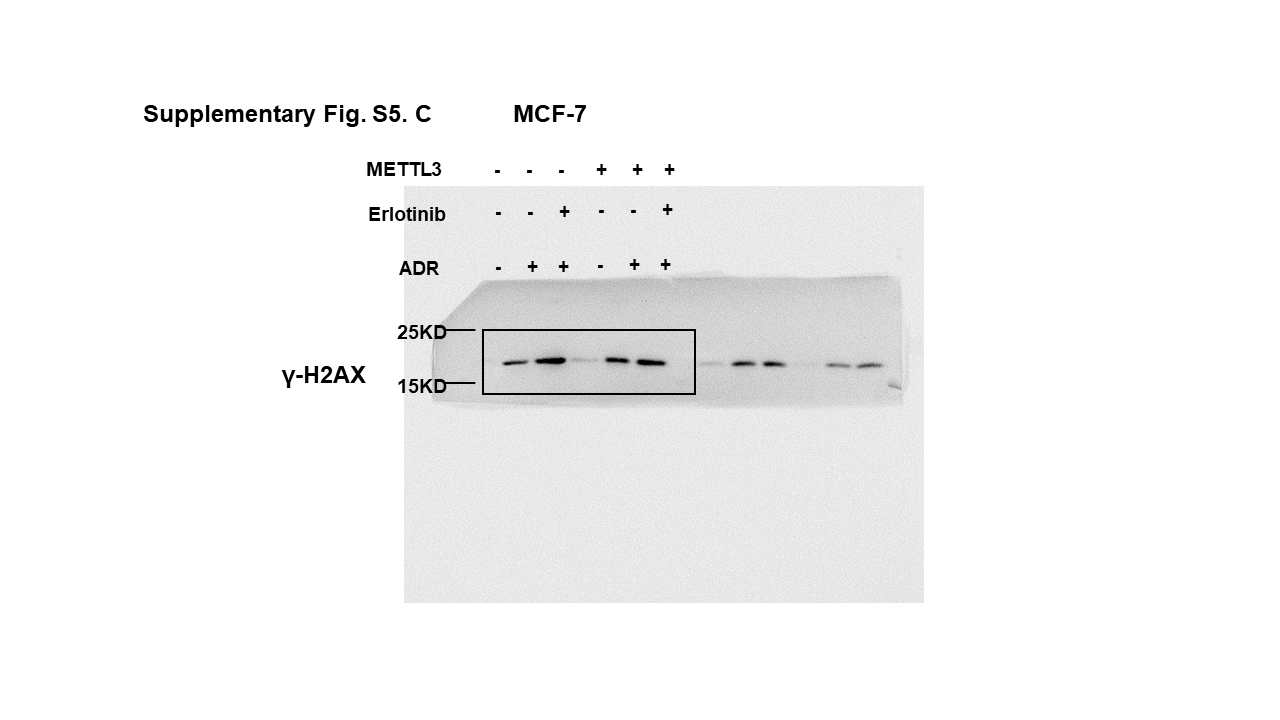

Supplement: Figure 5—figure supplement 1—source data 3. [file elife-75231-fig5-figsupp1-data3.zip › Figure S5C/Supplementary Fig.S5. C γ-H2AX.png.TIF]

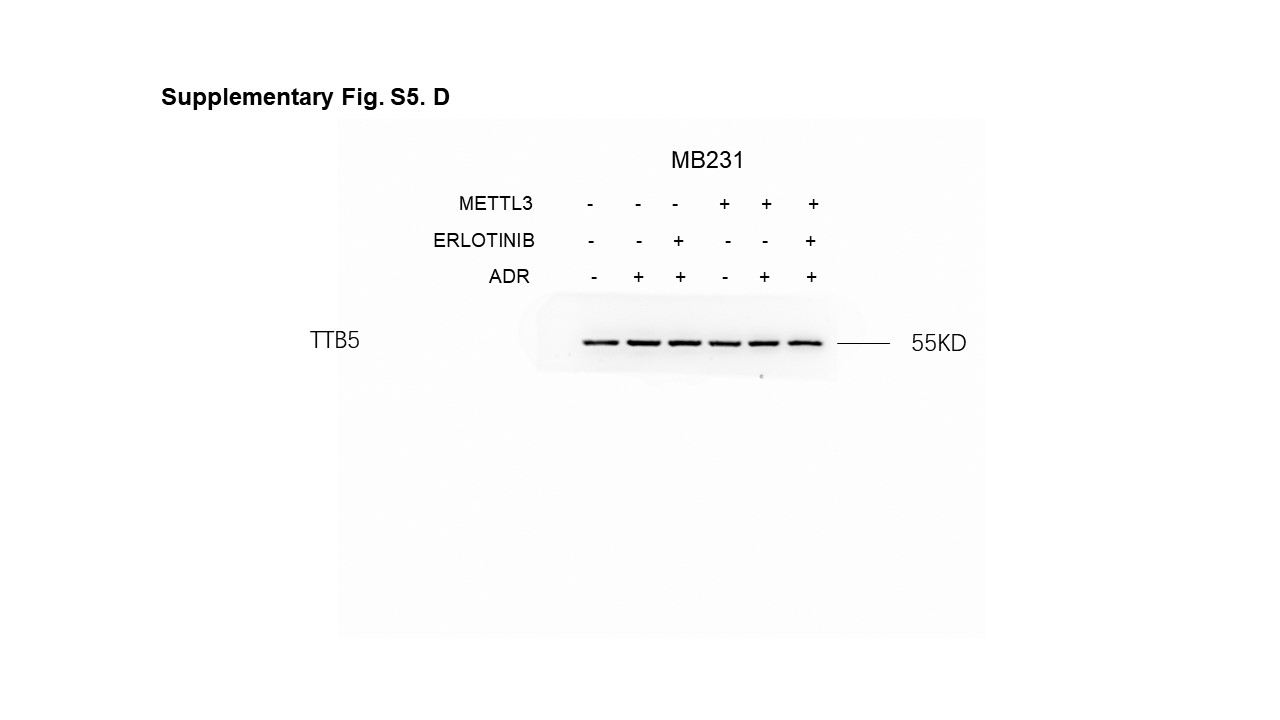

Supplement: Figure 5—figure supplement 1—source data 4. [file elife-75231-fig5-figsupp1-data4.zip › Figure S5D/Supplementary Fig. S5. D tbb5.JPG]

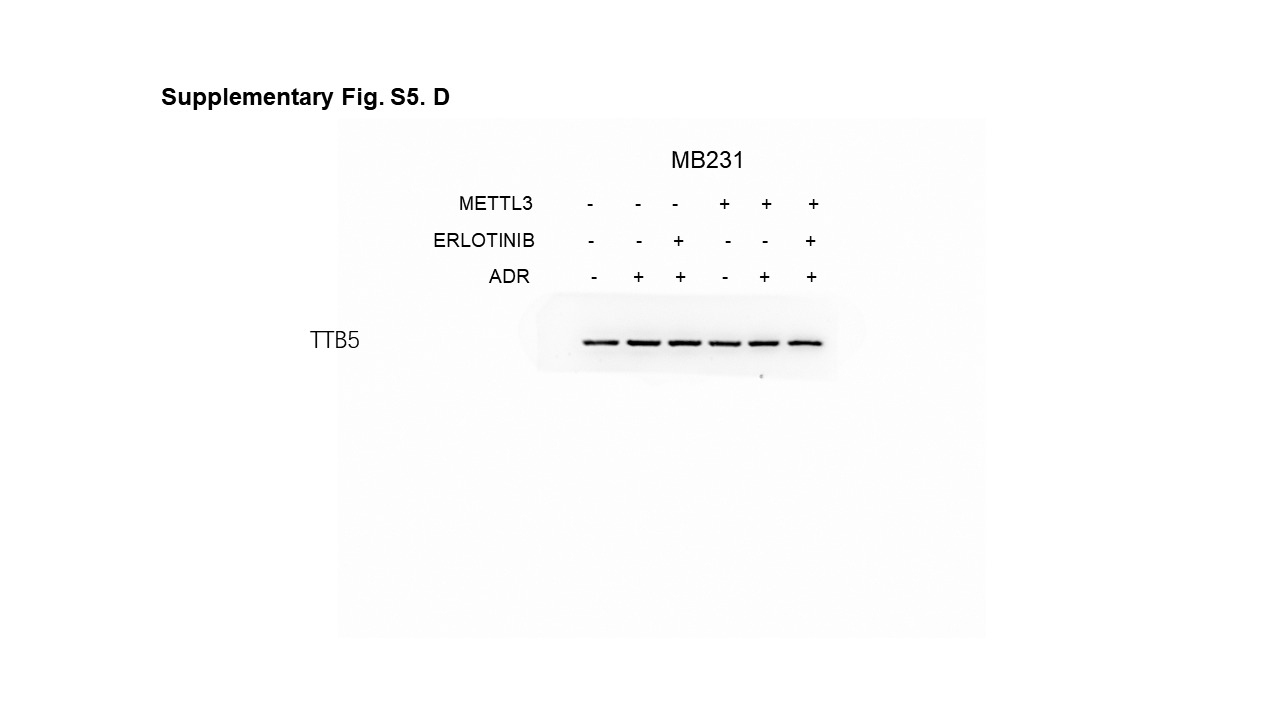

Supplement: Figure 5—figure supplement 1—source data 4. [file elife-75231-fig5-figsupp1-data4.zip › Figure S5D/Supplementary Fig. S5. D tbb5.TIF]

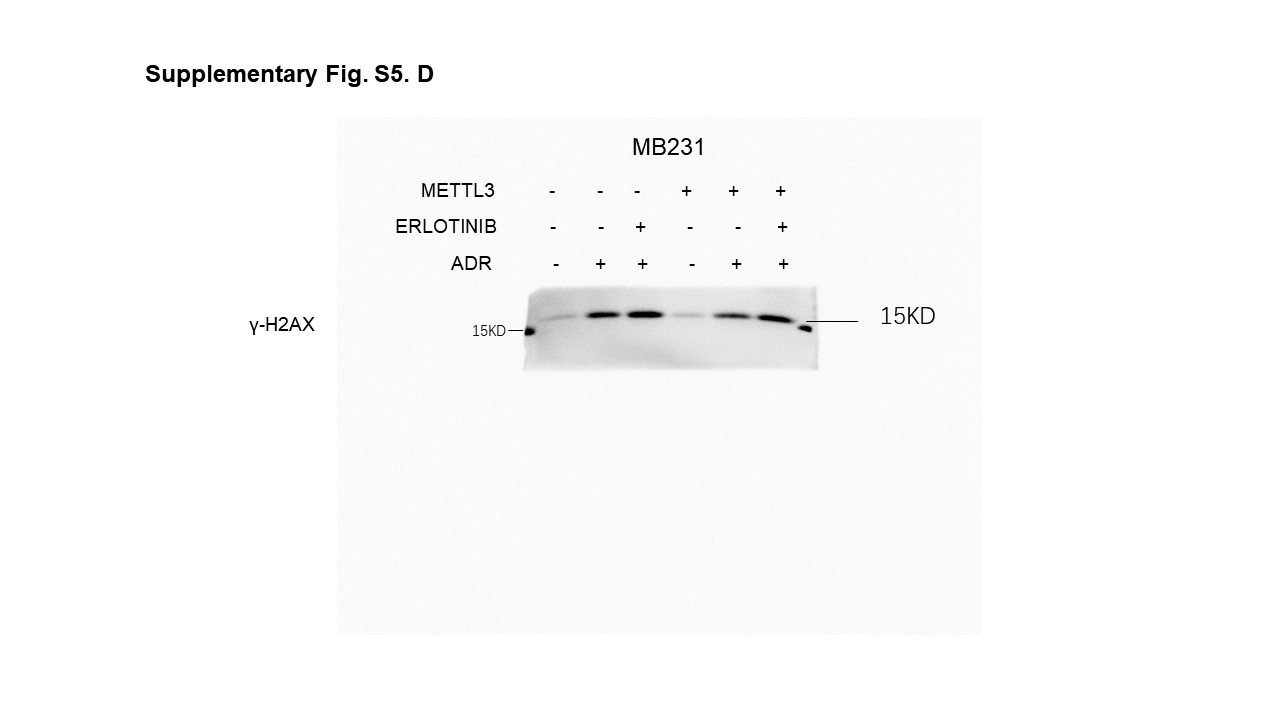

Supplement: Figure 5—figure supplement 1—source data 4. [file elife-75231-fig5-figsupp1-data4.zip › Figure S5D/Supplementary Fig. S5. D γh2ax.JPG]

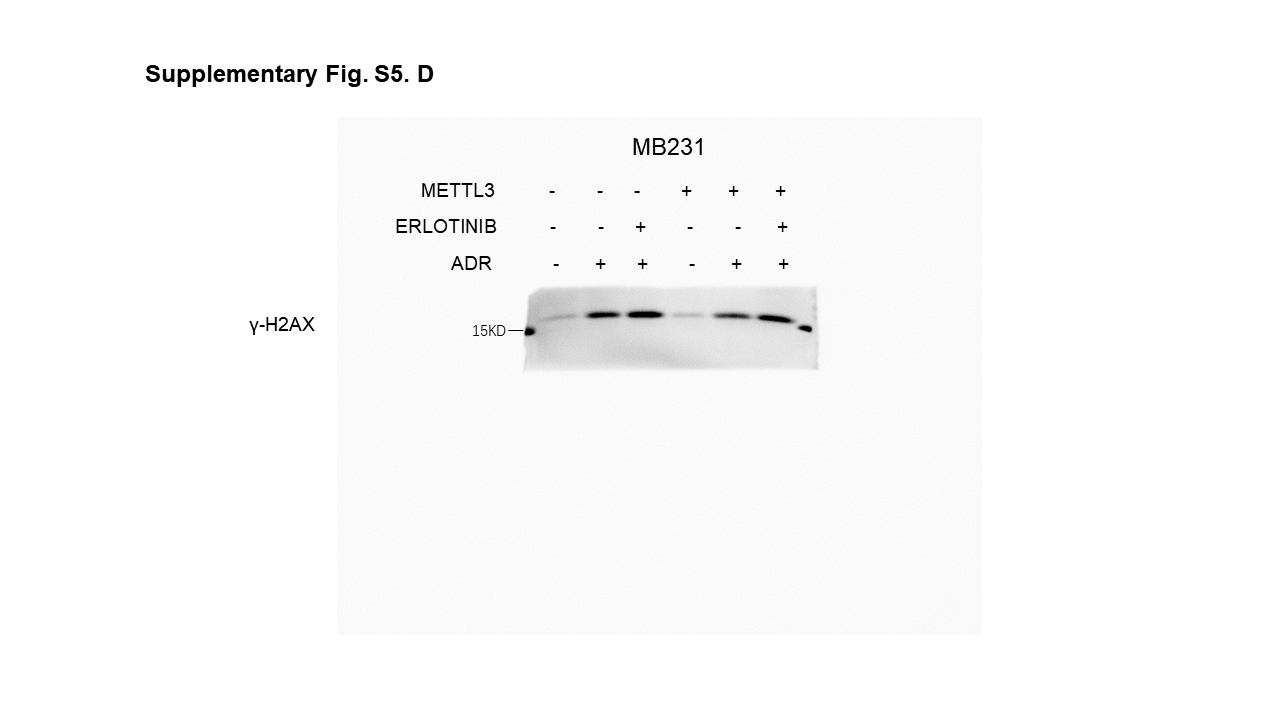

Supplement: Figure 5—figure supplement 1—source data 4. [file elife-75231-fig5-figsupp1-data4.zip › Figure S5D/Supplementary Fig. S5. D γh2ax.TIF]

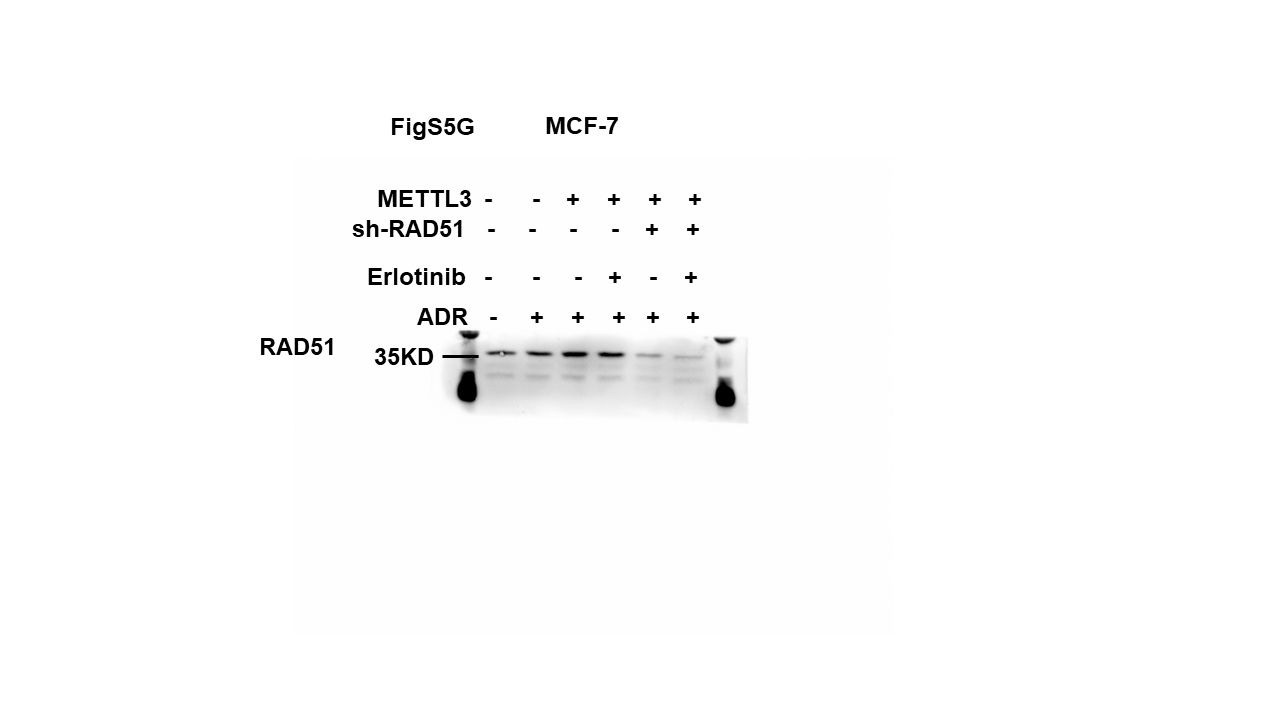

Supplement: Figure 5—figure supplement 1—source data 5. [file elife-75231-fig5-figsupp1-data5.zip › Figure S5G/Figure S5G RAD51.tif]

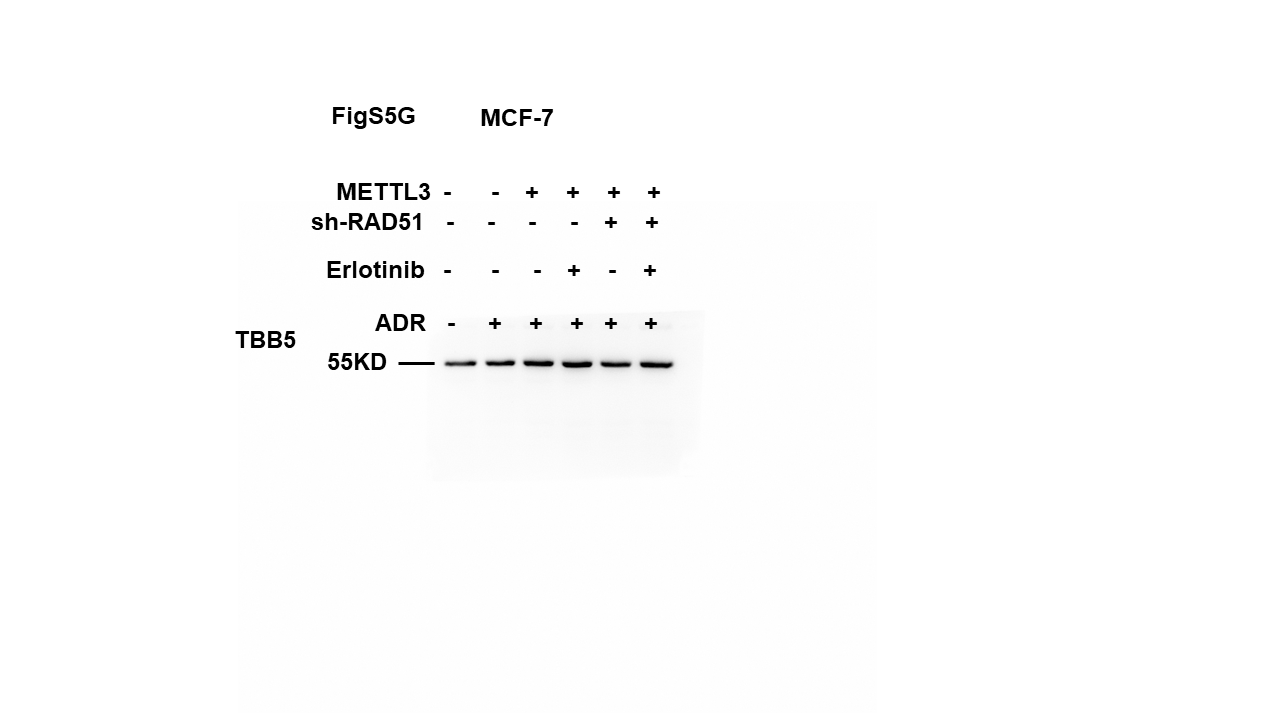

Supplement: Figure 5—figure supplement 1—source data 5. [file elife-75231-fig5-figsupp1-data5.zip › Figure S5G/Figure S5G TBB5.tif]

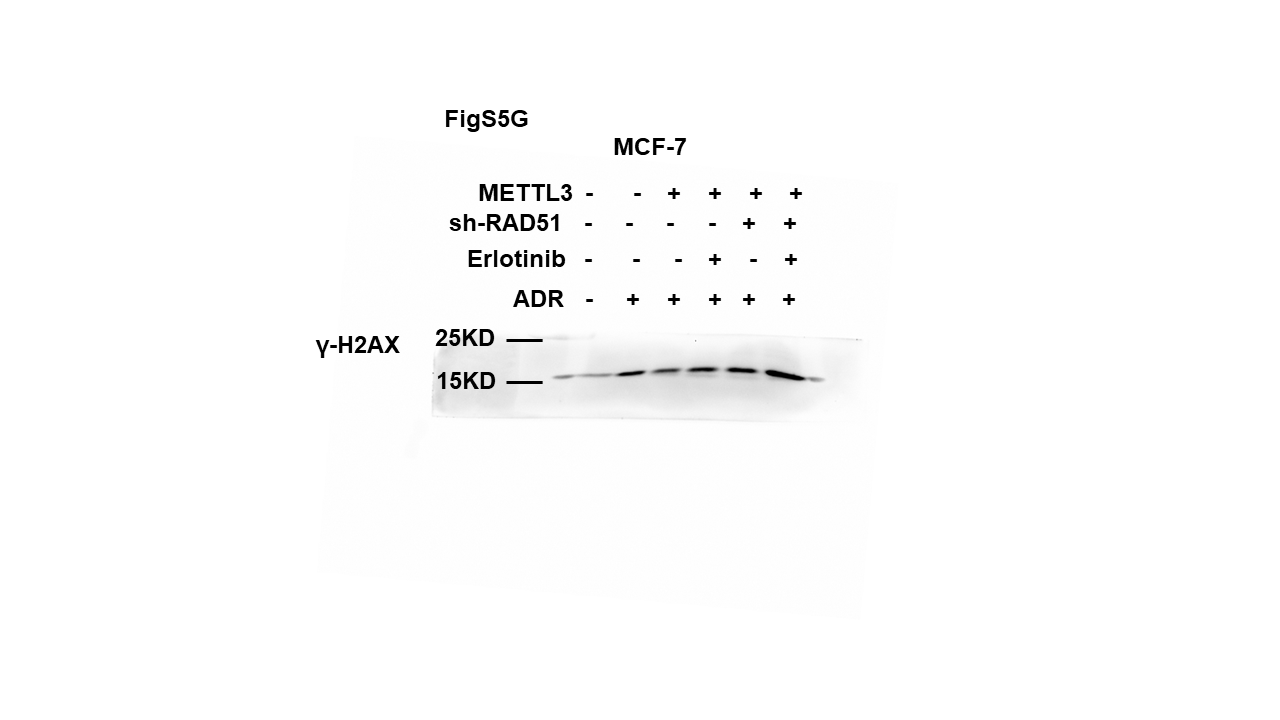

Supplement: Figure 5—figure supplement 1—source data 5. [file elife-75231-fig5-figsupp1-data5.zip › Figure S5G/Figure S5G a├-H2AX.tif]

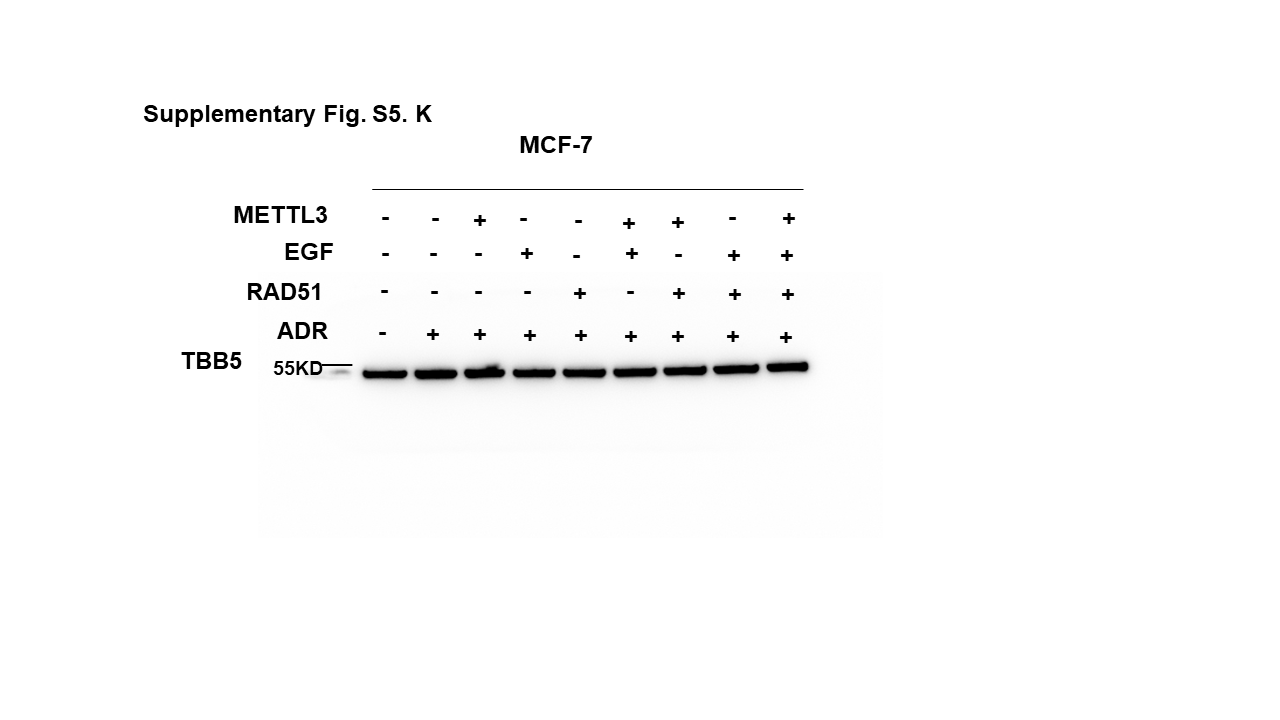

Supplement: Figure 5—figure supplement 1—source data 6. [file elife-75231-fig5-figsupp1-data6.zip › Figure S5K/Figure S5K-TBB5.tif]

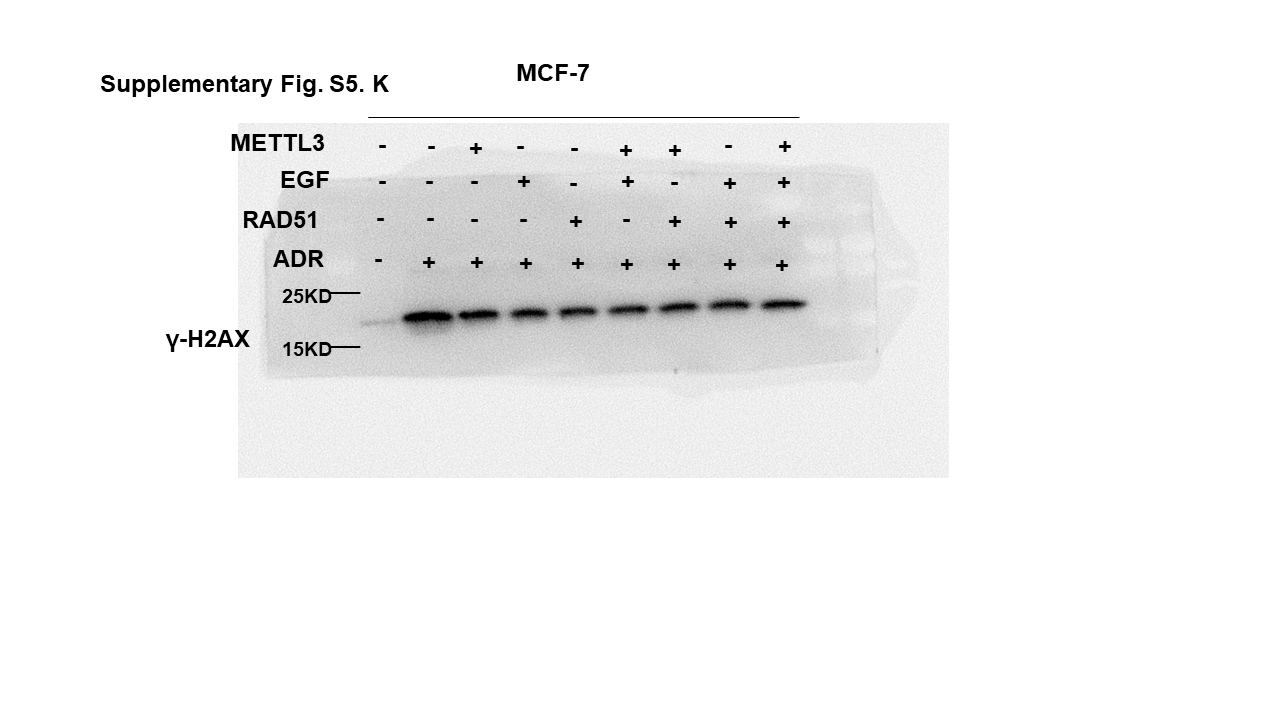

Supplement: Figure 5—figure supplement 1—source data 6. [file elife-75231-fig5-figsupp1-data6.zip › Figure S5K/Figure S5K-a├-H2AX.tif]

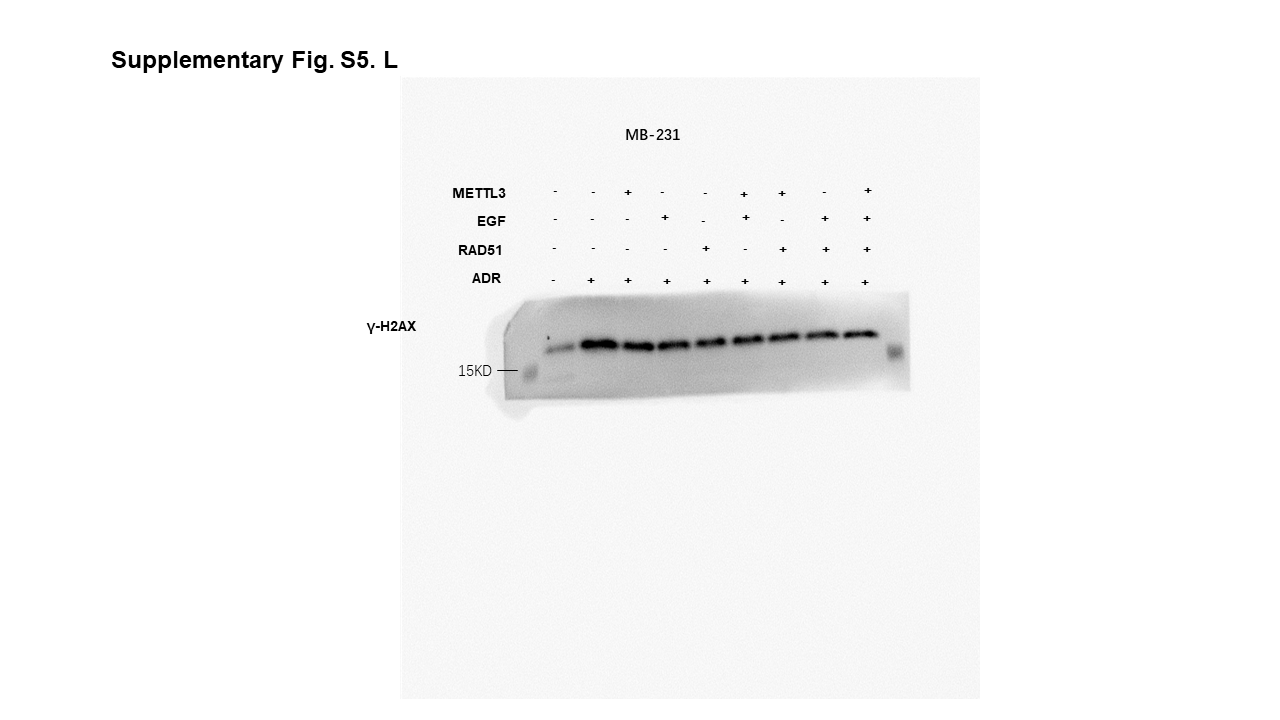

Supplement: Figure 5—figure supplement 1—source data 7. [file elife-75231-fig5-figsupp1-data7.zip › Figure S5L/Figure S5K a├-H2AX.tif]

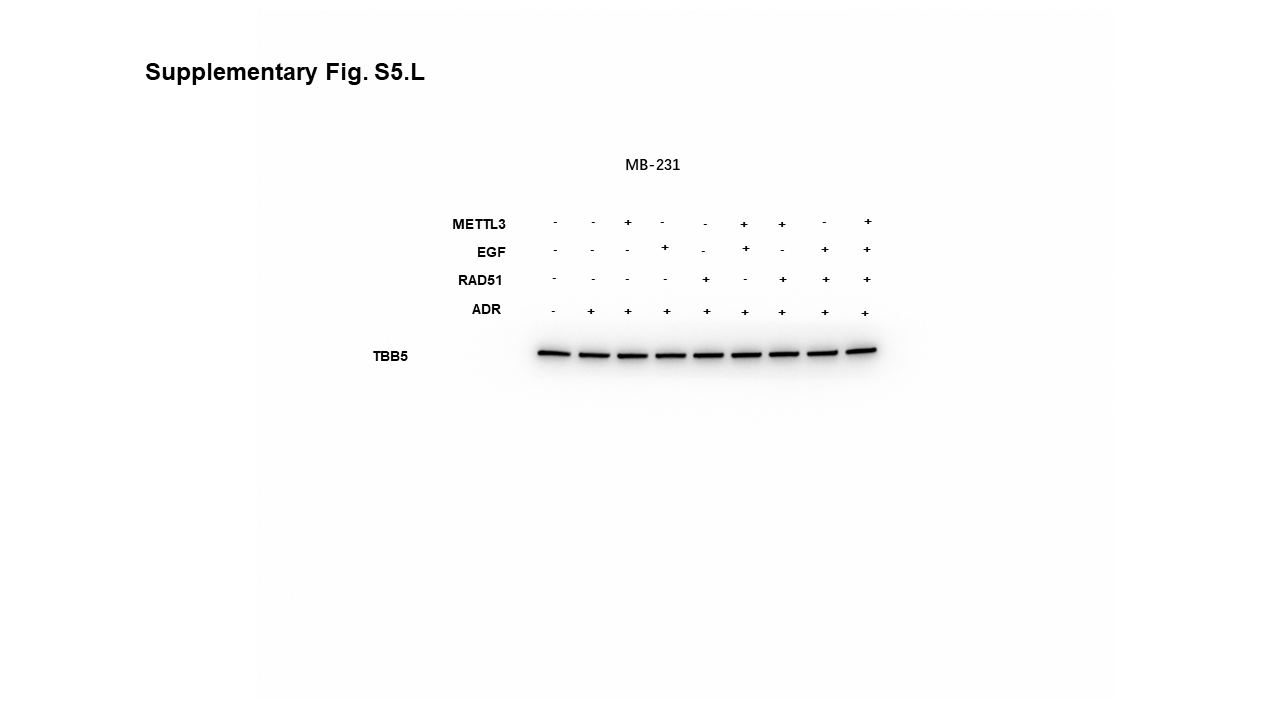

Supplement: Figure 5—figure supplement 1—source data 7. [file elife-75231-fig5-figsupp1-data7.zip › Figure S5L/figure S5L TBB5.tif]

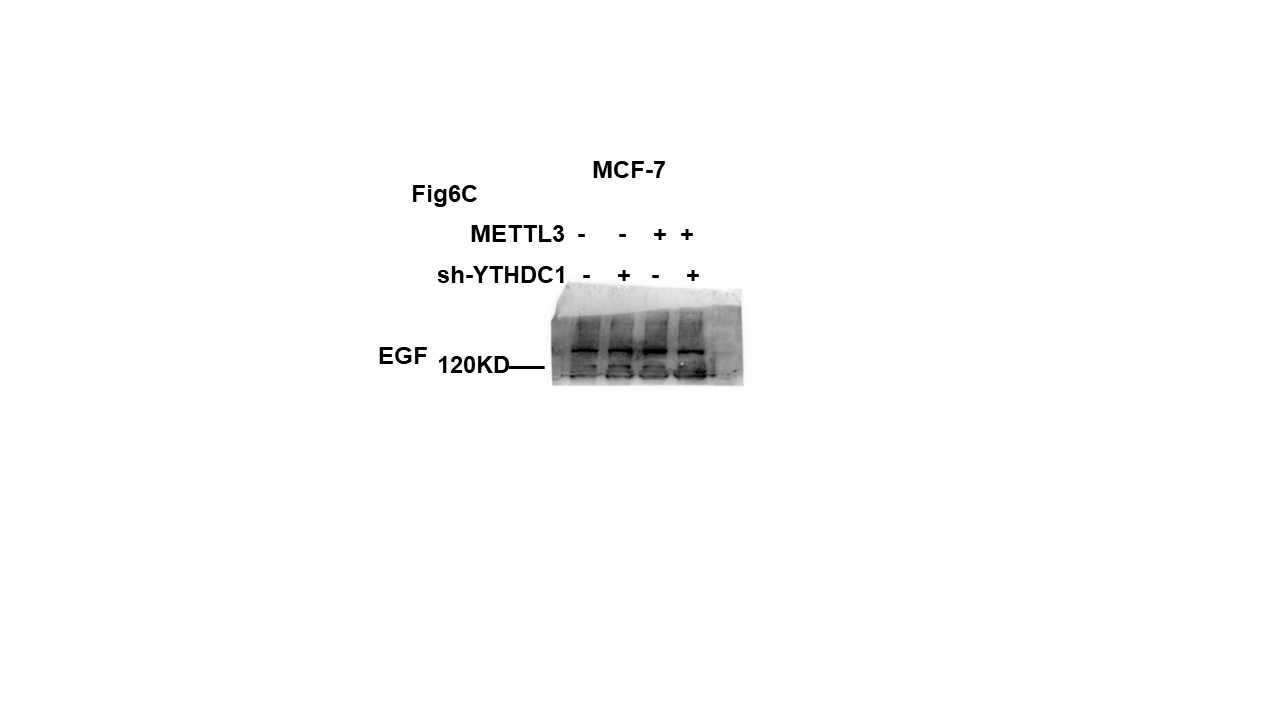

Supplement: Figure 6—source data 1. [file elife-75231-fig6-data1.zip › Figure 6c/Figure 6c EGF.TIF]

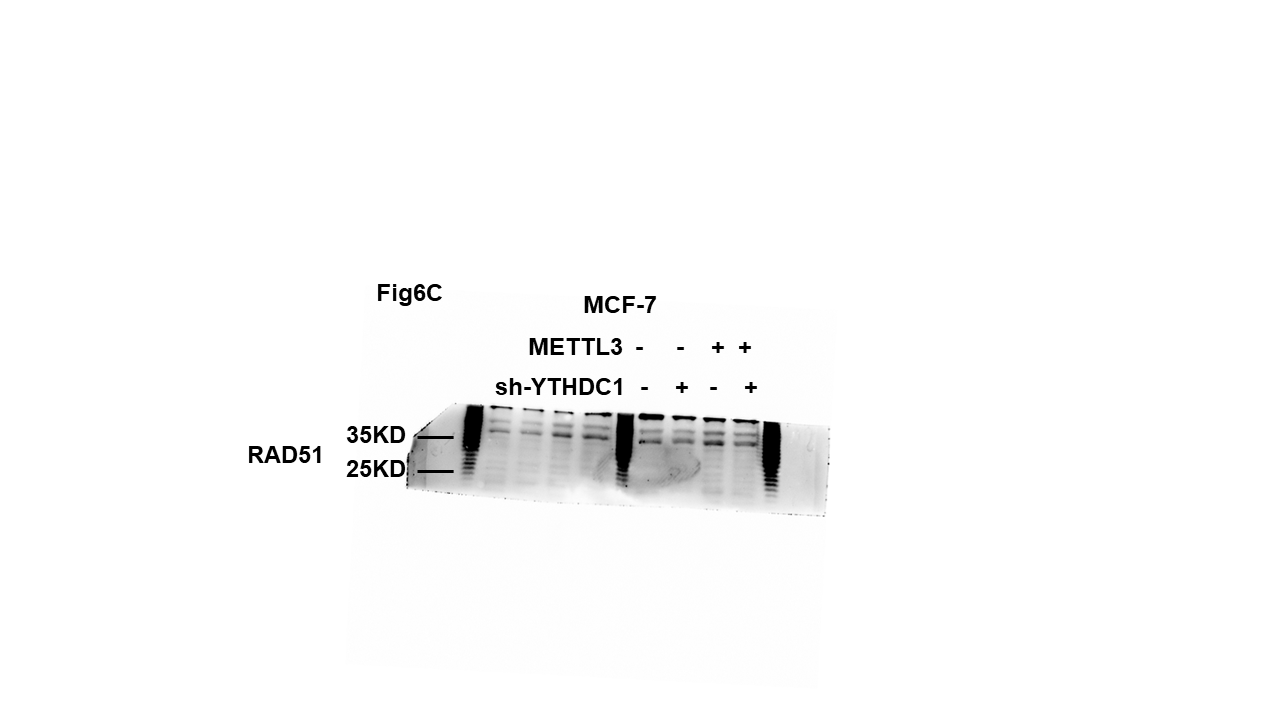

Supplement: Figure 6—source data 1. [file elife-75231-fig6-data1.zip › Figure 6c/Figure 6c RAD51 .TIF]

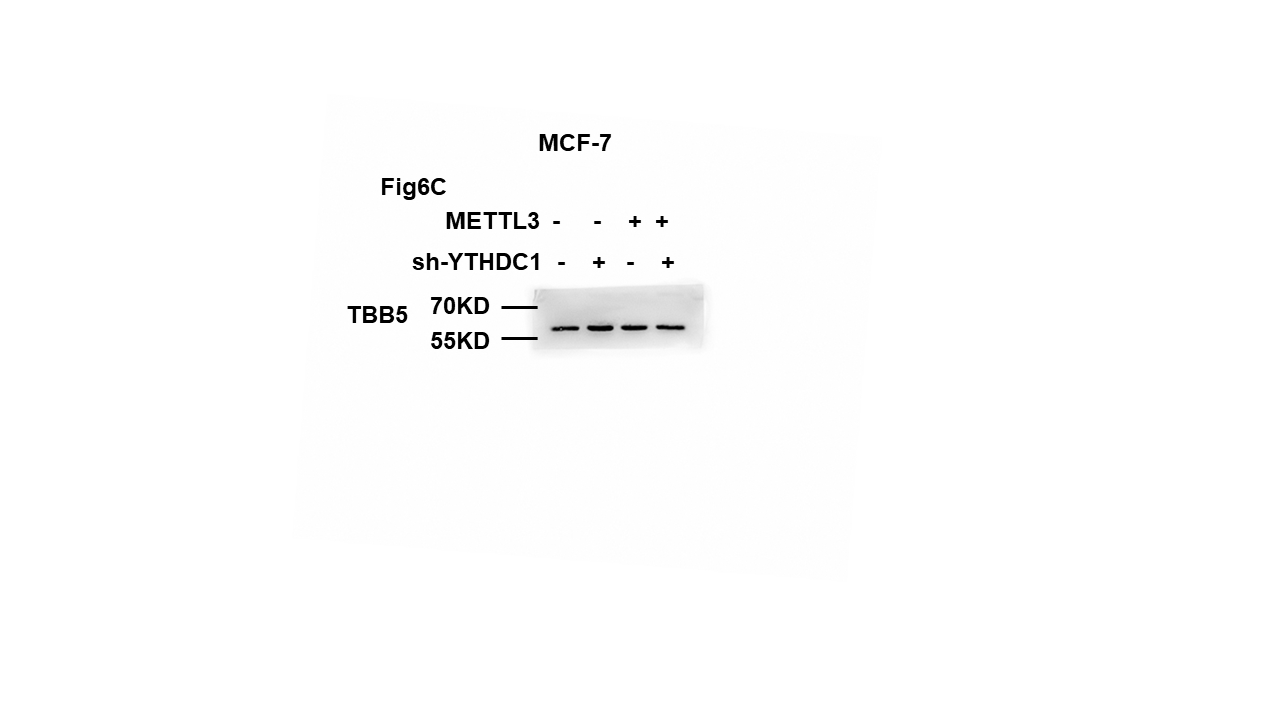

Supplement: Figure 6—source data 1. [file elife-75231-fig6-data1.zip › Figure 6c/Figure 6c TBB5.TIF]

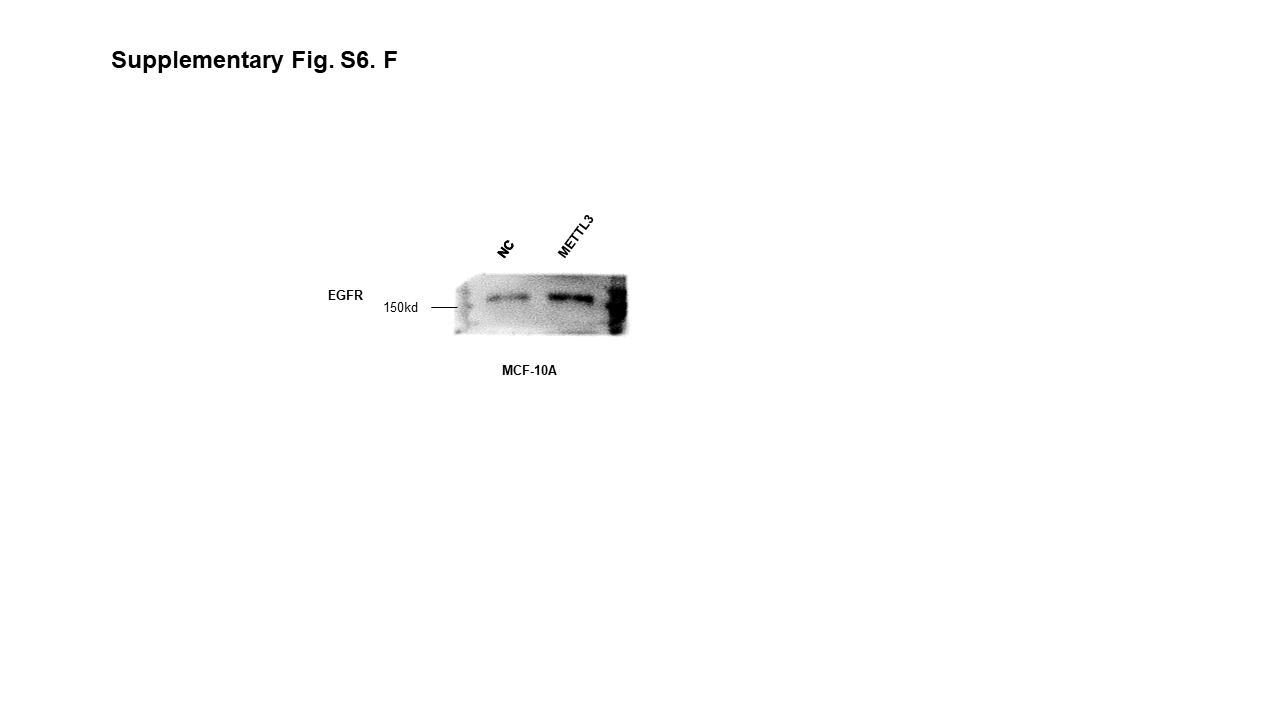

Supplement: Figure 6—figure supplement 1—source data 1. [file elife-75231-fig6-figsupp1-data1.zip › Figure S6F/Figure S6F EGFR.TIF]

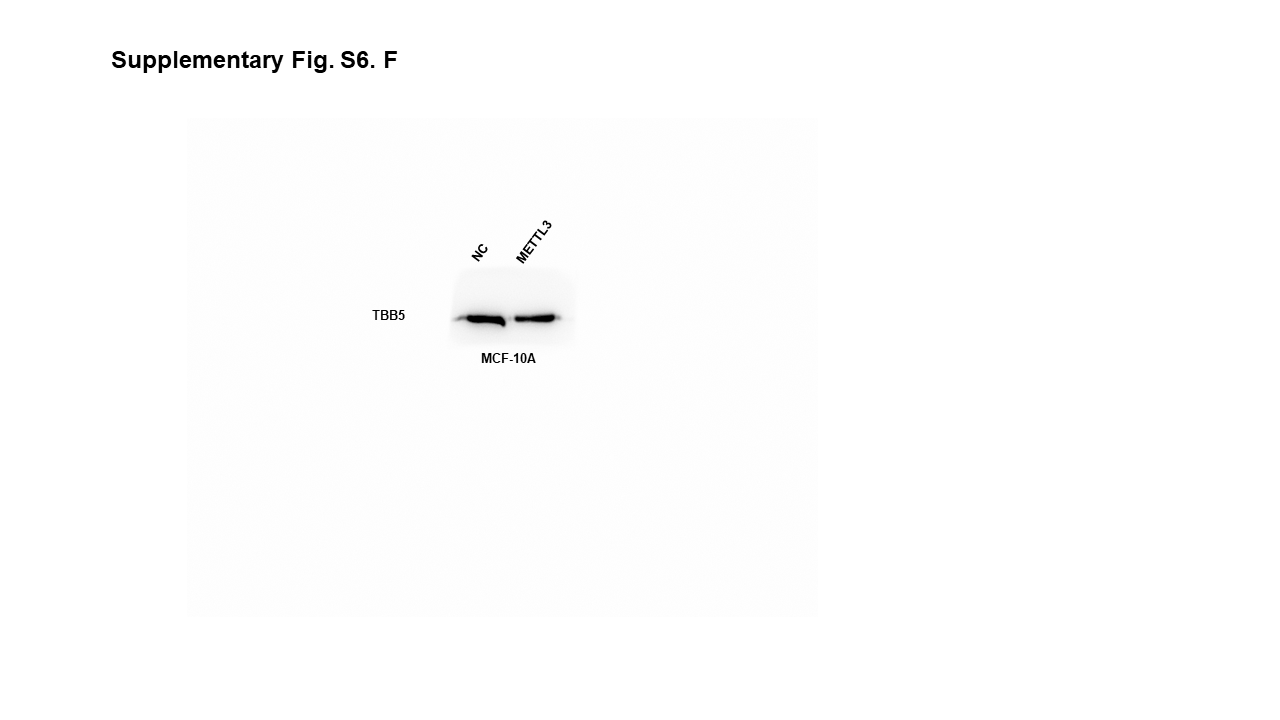

Supplement: Figure 6—figure supplement 1—source data 1. [file elife-75231-fig6-figsupp1-data1.zip › Figure S6F/Figure S6F TBB5.TIF]

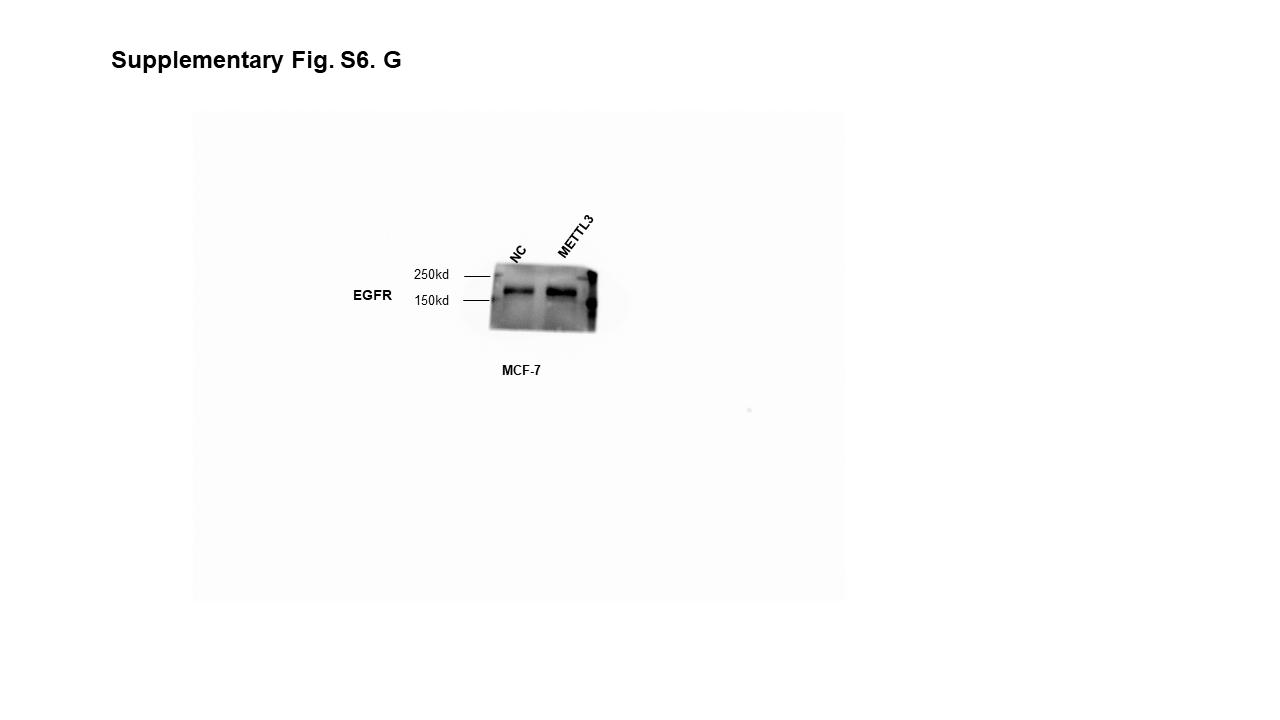

Supplement: Figure 6—figure supplement 1—source data 2. [file elife-75231-fig6-figsupp1-data2.zip › Figure S6G/Figure S6G EGFR.TIF]

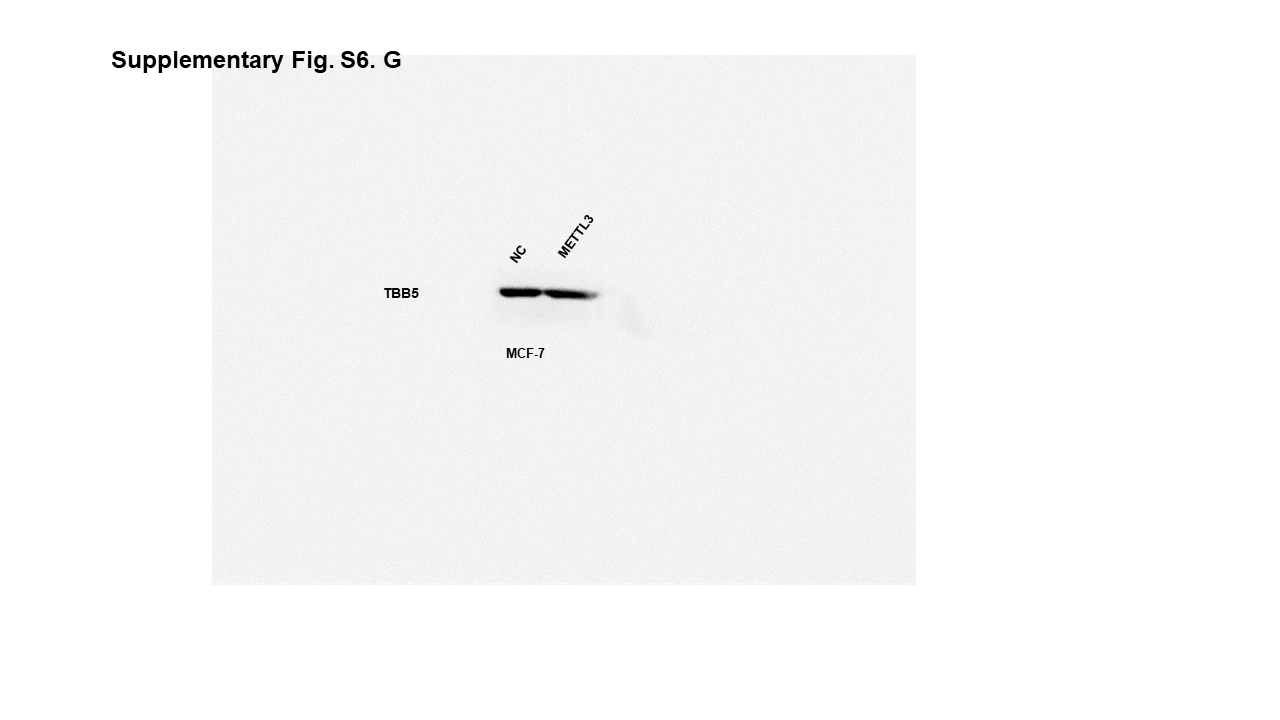

Supplement: Figure 6—figure supplement 1—source data 2. [file elife-75231-fig6-figsupp1-data2.zip › Figure S6G/Figure S6G TBB5.TIF]

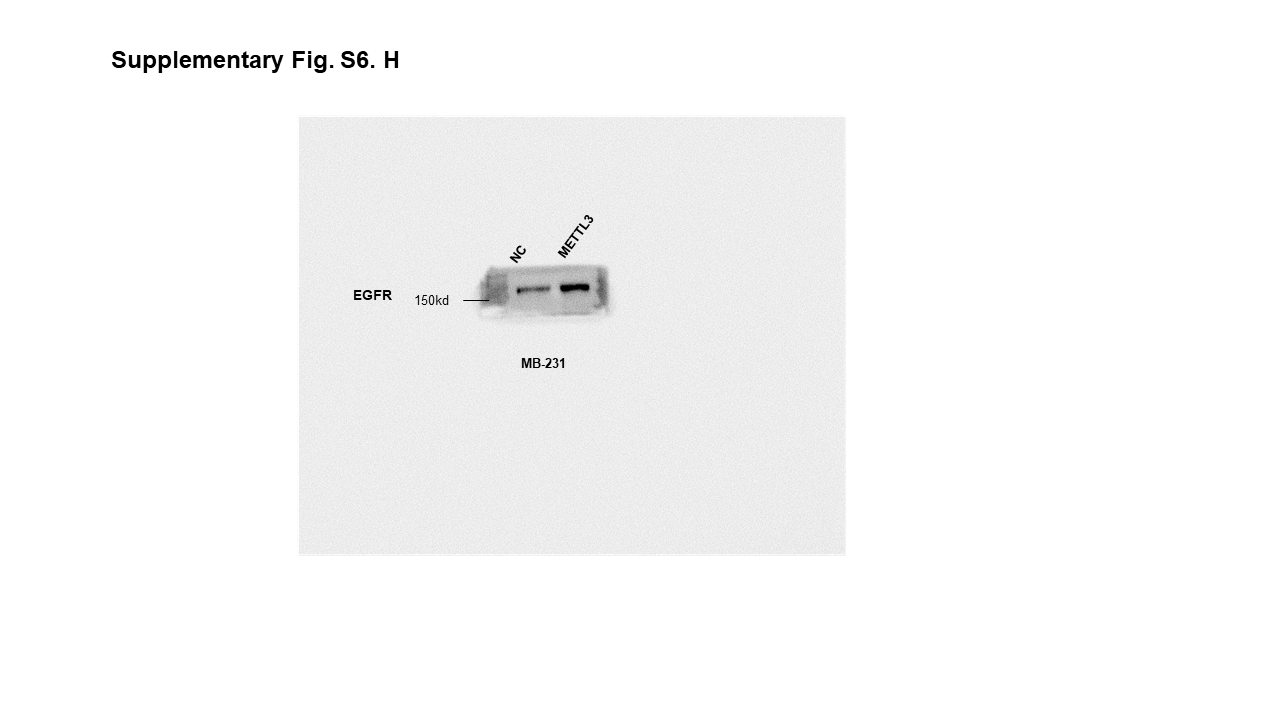

Supplement: Figure 6—figure supplement 1—source data 3. [file elife-75231-fig6-figsupp1-data3.zip › Figure S6H/Figure S6H EGFR.TIF]

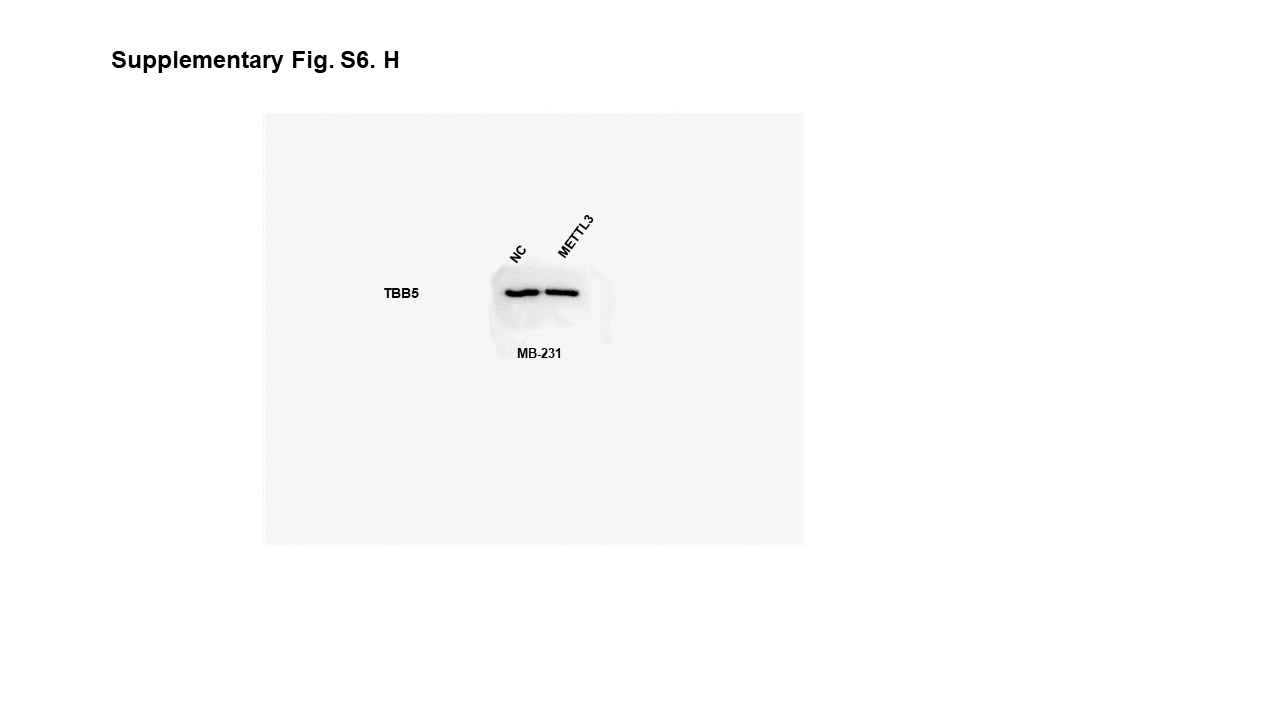

Supplement: Figure 6—figure supplement 1—source data 3. [file elife-75231-fig6-figsupp1-data3.zip › Figure S6H/Figure S6H TBB5.tif]
